# Genetic Mapping and Validation of Loci for Kernel-Related Traits in Wheat (*Triticum aestivum* L.)

**DOI:** 10.3389/fpls.2021.667493

**Published:** 2021-06-07

**Authors:** Xiangru Qu, Jiajun Liu, Xinlin Xie, Qiang Xu, Huaping Tang, Yang Mu, Zhien Pu, Yang Li, Jun Ma, Yutian Gao, Qiantao Jiang, Yaxi Liu, Guoyue Chen, Jirui Wang, Pengfei Qi, Ahsan Habib, Yuming Wei, Youliang Zheng, Xiujin Lan, Jian Ma

**Affiliations:** ^1^State Key Laboratory of Crop Gene Exploration and Utilization in Southwest China, Triticeae Research Institute, Sichuan Agricultural University, Chengdu, China; ^2^College of Agronomy, Sichuan Agricultural University, Chengdu, China; ^3^College of Agronomy and Biotechnology, China Agricultural University, Beijing, China; ^4^Biotechnology and Genetic Engineering Discipline, Khulna University, Khulna, Bangladesh

**Keywords:** common wheat, 55K SNP array, kernel traits, quantitative trait locus, validation

## Abstract

Kernel size (KS) and kernel weight play a key role in wheat yield. Phenotypic data from six environments and a Wheat55K single-nucleotide polymorphism array–based constructed genetic linkage map from a recombinant inbred line population derived from the cross between the wheat line 20828 and the line SY95-71 were used to identify quantitative trait locus (QTL) for kernel length (KL), kernel width (KW), kernel thickness (KT), thousand-kernel weight (TKW), kernel length–width ratio (LWR), KS, and factor form density (FFD). The results showed that 65 QTLs associated with kernel traits were detected, of which the major QTLs *QKL.sicau-2SY-1B*, *QKW.sicau-2SY-6D*, *QKT.sicau-2SY-2D*, and *QTKW.sicau-2SY-2D*, *QLWR.sicau-2SY-6D*, *QKS.sicau-2SY-1B*/*2D*/*6D*, and *QFFD.sicau-2SY-2D* controlling KL, KW, KT, TKW, LWR, KS, and FFD, and identified in multiple environments, respectively. They were located on chromosomes 1BL, 2DL, and 6DS and formed three QTL clusters. Comparison of genetic and physical interval suggested that only *QKL.sicau-2SY-1B* located on chromosome 1BL was likely a novel QTL. A Kompetitive Allele Specific Polymerase chain reaction (KASP) marker, *KASP-AX-109379070*, closely linked to this novel QTL was developed and used to successfully confirm its effect in two different genetic populations and three variety panels consisting of 272 Chinese wheat landraces, 300 Chinese wheat cultivars most from the Yellow and Huai River Valley wheat region, and 165 Sichuan wheat cultivars. The relationships between kernel traits and other agronomic traits were detected and discussed. A few predicted genes involved in regulation of kernel growth and development were identified in the intervals of these identified major QTL. Taken together, these stable and major QTLs provide valuable information for understanding the genetic composition of kernel yield and provide the basis for molecular marker–assisted breeding.

## Introduction

Common wheat (*Triticum aestivum* L. 2*n* = 6*x* = 42, AABBDD) is a widely grown cereal crop that provides energy and nutrition for human life. According to the statistics of the Food and Agriculture Organization, the annual yield of wheat must increase 1.6% to ensure the food demand of 9.1 billion people in the world by 2050 (Patil et al., 2013). With the increase of world population and the decrease of the planting area, increasing food production is of great strategic significance for solving future food supply and security issues.

The kernel-related traits can be divided into a number of components including thousand-kernel weight (TKW), kernel length (KL), kernel width (KW), kernel thickness (KT), kernel length–width ratio (LWR), kernel size (KS), and factor form density (FFD). Larger kernels have a positive influence on the vigor of wheat seedlings and yield increasing (Börner et al., 2002) and also give a beneficial effect on agronomic values and flour yield (Chastian et al., 1995). TKW was accompanied by a high heritability (Alexander et al., 1984), and KS usually influences TKW to promote yield. Therefore, improving KS and TKW is a prior breeding goal to enhance wheat yield and quality.

In recent years, kernel-related traits have received widespread attention in molecular genetics. Quantitative trait locus (QTL) mapping based on molecular markers has been widely used in the study of kernel traits of rice (Agrama et al., 2007; Huang et al., 2012), wheat (Sun et al., 2009; Ramya et al., 2010), maize (Yang et al., 2016; Lan et al., 2018), and other major crops. Genes controlling kernel traits were identified in diploid plant species such as *Arabidopsis thaliana* and rice (Hu et al., 2018; Xia et al., 2018; Ying et al., 2018). With the fast development of molecular biotechnology and comparative genomics, a large number of genes have been identified to adjust rice kernel shape in previous studies. For example, *qGL7* (Bai et al., 2010), *Gn1a* (Ashikari et al., 2005), *GS3* (Fan et al., 2006), *qSS7* (Qiu et al., 2012), and *GW2* (Song et al., 2007) regulate kernel development, positively or negatively. Several rice orthologs regulating KS and kernel weight in wheat were isolated and identified through homologous cloning technology. For example, *TaCwi-1A* was involved in the development of kernel size, which was a critical enzyme for sink tissue development and carbon allocation (Ma et al., 2012). *TaCKX6-D1* played a major role by controlling cytokinin levels, and its haplotype variants were determined to be significantly associated with TKW (Zhang et al., 2012). *GW8* (Yan et al., 2019) was a SQUAMOSA Promoter-Binding Protein-Like (SPL) family transcription factor whose function was similar to *GW2* and *GW5* genes and played a regulatory role of kernel traits and TKW in wheat (Song et al., 2007; Cheng et al., 2020).

Because of the complexity of wheat genome, genetic research of wheat yield–related traits and their components were mainly focused on QTL mapping and molecular marker development. Recently, numerous QTLs/genes for yield-related traits in wheat have been identified on almost 21 wheat chromosomes (Cui et al., 2014, 2016; Huang et al., 2015; Wu et al., 2015; Brinton et al., 2017; Cao et al., 2019; Sakuma et al., 2019; Chen et al., 2020; Cheng et al., 2020; Ren et al., 2021). However, the effects of QTL in hexaploid wheat were usually subtle than those identified in rice, and few environmental-stable QTLs have been identified and validated (Cristobal, 2017). Therefore, identification of major QTLs and development of effective markers will help accelerate molecular-assisted breeding and thus improve wheat breeding process.

In this study, we identified QTLs for seven kernel-related traits that were stably expressed in six environments. A new Kompetitive Allele Specific Polymerase chain reaction (KASP) marker was developed to further validate a novel QTL for KL (*QKL.sicau-2SY-1B*). Candidate genes were predicted for the major QTLs of four kernel-related traits. Additionally, we evaluated the correlation between kernel-related traits and other agronomic traits.

## Materials and Methods

### Plant Materials

**FIGURE 1:**
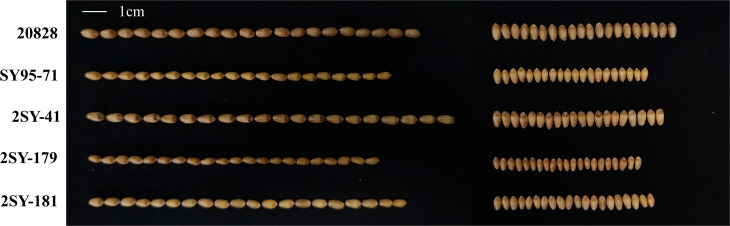
Kernel phenotypes of the parent “20828,” “SY95-71,” and partial RILs. The white line represents the scale = 1 cm.

A recombinant inbred line (RIL) mapping population developed from the cross between 20828 and SY95-71 (abbreviated as 2SY) containing 126 F_7_ lines plus parents was used for mapping QTL for kernel-related traits (Liu et al., 2020). The line 20828 (G214-5/3/Chuanyu19//Lang 9247/50788) shows longer kernels than SY95-71 (Figure 1) and has other excellent agronomic traits including multiple spikelets (Ma et al., 2019a) and high resistance to stripe rust (Ma et al., 2019b). SY95-71 (Eronga 83/Fan 6//Fan 6) is a stable line with well-developed root system (Zheng et al., 2019), more tillers (Liu et al., 2020), and better plant type (Tu et al., 2021). These excellent agronomic traits have enabled 20828 and SY95-71 to be widely used as a breeding parent in the past few years. Another two populations used for validating the identified QTL were developed with SY95-71 as a common parent, and they were derived from crosses S849-8/SY95-71 (SSY, 214 F_5_ lines) (Liu et al., 2020) and MTL4-5-3/SY95-71 (MTL4SY, F_3_ 200 lines) (Tu et al., 2021). Seeds of 20828 (ZM030677) and SY95-71 (ZM030678) were submitted to the Chinese Crop Germplasm Resources Information System hosted by National Germplasm Bank, China (NGBC). S849-8 and MTL4-5-3 were advanced wheat lines that have been used in wheat breeding. In addition, three variety panels were further used to evaluate the effect of the major QTLs, and they were (1) 272 Chinese wheat landraces (CWLs) genotyped by Wheat660K single-nucleotide polymorphism (SNP) array (Zhou et al., 2018); (2) 300 Chinese wheat cultivars (CWCs) collected from different wheat production regions of China, but most from the Yellow and Huai River Valley wheat region genotyped by Wheat55K SNP array (Jin et al., 2020); (3) 165 Sichuan wheat cultivars (SWCs) genotyped by Wheat55K SNP array (Ye et al., 2019). The information of these variety panels is listed in Supplementary Table 1.

### Phenotypic Evaluation

The RILs and parents were planted in six different environments including Chongzhou (103°38′E, 30°32′N) in 2017, 2018, and 2019 (2017CZ, 2018CZ and 2019CZ); Ya’an (103°0′E, 29°58′N) in 2017 and 2018 (2017YA and 2018YA); and Wenjiang (103°51’ E, 30°43′ N) in 2019 (2019WJ).

The RILs and parents in all the environments were designed by random blocks. Each line was planted in a single 1.5-m-length row with 0.3 m between rows. The sowing density was 15 seeds per row with 0.1 m between plants within a row. Field management followed conventional practices in wheat production. Five representative and well-pollinated spikes per line were selected and harvested for phenotypic measurement at the maturity stage. The seeds were fully cleaned and dried, and broken grains were removed before trait evaluations. Uniform and full seeds were selected for the measurement of kernel traits. Thirty kernels of each line were scanned by Epson Expression 10,000XL for measuring KL, KW, and KT and further calculated by WinSEEDLE (Regent Instruments Canada Inc.). TKW was calculated as 10-folds of the weight of 100 seeds with electronic balance, and three replicates for each line were set. The LWR was calculated by KL/KW, and the KS was expressed by the product of the KL, width, and thickness (Zhou et al., 2020); the FFD was calculated by TKW/[KL × KW], which describes the differences in kernel density (Prashant et al., 2012). Spikelet number per spike (SNS) was measured by counting the number of spikelets per the main spike; plant height (PH) was measured from the base of the plant to the top of the main spike (awns were not included); productive tiller number (PTN) was calculated as the number of branches that can produce a wheat spike; flag leaf length (FLL) and width (FLW) refer to the longest and widest distance of the first leaf under the wheat spike; spike length (SL) was measured from the base of the main spike to the top of the main spike (awns were not included); and spike density (SD) was calculated by dividing spikelet number by SL. Anthesis date (AD) was measured from the planting date to the date when half the plants in a given line bloom. Among TKW and other agronomic traits, TKW data in 2018 are newly added, and the other data of TKW, PTN, SNS, PH, AD (Liu et al., 2020), SL (Li et al., 2020), FLL, and FLW (Ma et al., 2020) were reported previously (Supplementary Table 2).

The 272 CWLs were planted in six different environments, including 2012YA, 2013-2015WJ, and 2014-2015CZ (Liu et al., 2017). Fifty kernels for each accession were measured by Win-Rhizo Pro 2008a image analysis system. The mean value of each accession in a single environment was used for further analysis (Liu et al., 2017).

The 300 CWCs were planted in three different environments, including Beijing in 2018 and 2019, and Baoding in 2019 (Gao et al., 2021). One hundred twenty seeds of each accession were planted in a single row of 2-m length with 25-cm spacing between the rows in each environment (Gao et al., 2021). The spikes were harvested at physiological maturity. The seeds were manually removed from the spikelet. For the 165 SWCs, 20 seeds of each accession were planted in rows 2 m in length with 30-cm spacing and individual plants spaced 10 cm apart in 2020CZ. The kernel-related traits of 300 Chinese common wheat and 165 SWCs were measured using a Crop Grain Appearance Quality Scanning Machine (SC-A, Hangzhou Wanshen Detection Technology Co., Ltd, Hangzhou, China).

### Map Construction and Statistical Analysis

The genetic linkage map utilized in this study was previously constructed by the Wheat55K SNP array. A total of 2,529 bin markers based on 2,517 wheat 55K SNPs and 12 polymerase chain reaction (PCR)–based markers were used to construct the genetic map containing 38 linkage groups in 2SY population. The total length of the genetic map is 4,761.34 cM, the average marker density is 1.88 cM, the minimum value is 0.70 cM, and the maximum value is 5.33 cM (Liu et al., 2020).

The phenotypic differences between parents, Pearson correlation between the seven kernel traits, frequency distributions obtained each year in each environment, and analysis of variance (ANOVA) for major and minor QTLs of each kernel trait with the best linear unbiased prediction (BLUP) value were tested using IBM SPSS Statistic 26 (SPSS, Chicago, IL, United States)^1^. SAS V8.0 (SAS Institute, Cary, NC, United States) was used for calculating BLUP and broad-sense heritability (*H*^2^) for all the investigated traits from different environments. The correlation coefficients of SNS, PH, PTN, FLL, FLW, SL, SD, and AD agronomic traits with kernel-related traits were analyzed using BLUP values of multienvironment phenotypes. The *H*^2^ was calculated as follows: *H*^2^ = *V*_G_/(*V*_G_ + *V*_GE_/*r* + *V*_E_), where *V*_G_ is genotypic variance; *V*_GE_, genotype × environment variance; *r*, the number of replicates; and *V*_E_, environmental variance (Smith et al., 1998).

Bip (QTL mapping in biparental populations) module of QTL IciMapping (version 4.1, based on ICIM)^2^ was used to detect the kernel traits QTL with values from six single environments and the BLUP dataset. Probability in stepwise regression (PIN) parameter value was 0.001, the step size chosen for all QTLs was 1 cM, and QTLs were claimed to be significantly above the 2.5 logarithm of odds (LOD) threshold. Combined analysis of kernel traits in the multienvironment by using Met (QTL mapping for multienvironmental trials) module of QTL IciMapping. The QTL interval on the genetic map was defined as the genetic distance between the two flanking markers of the QTL peak. Among the QTLs detected in more than three environments with the phenotypic variation, greater than 10% were considered as stable and major QTLs, whereas the rest were considered as secondary QTL.

### Nomenclature of QTLs

QTLs were named according to the rules of International Rules of Genetic Nomenclature^3^.

All QTLs were named as follows: “Q” stands for “QTL,” followed by the letter Q are the abbreviations of the corresponding kernel traits (KL, KW, KT, TKW, LWR, KS, and FFD represent kernel length, kernel width, kernel thickness, thousand-kernel weight, kernel length–width ratio, kernel size, and factor from density, respectively); “sicau” is the abbreviation of “Sichuan Agricultural University,” and “2SY” stands for the mapping populations name used in this study; the last is the wheat chromosome on which the corresponding QTL is distributed; if more than one QTL for a trait was distributed on the same chromosome, a serial number, viz.1, 2, 3, etc., was used to describe their order after the chromosome name, from the short arm to the long arm.

### Comparison of the Physical Intervals for Major QTLs Reported Here and Previously

The physical locations of the flanking markers associated with the major kernel trait QTLs were obtained as described previously (Liu et al., 2020). Previously reported flanking marker sequences of QTL for kernel-related genes were downloaded from NCBI^4^ and GrainGenes 3.0^5^, and further blasted against the *T. aestivum* cv. ‘‘Chinese Spring (CS)’’ (IWGSC RefSeq v1.0)^6^ (International Wheat Genome Sequencing Consortium, 2018) reference genome to obtain their physical locations.

### Marker Development and QTL Validation

According to QTL mapping results, we converted SNP marker *AX-109379070* closely linked to the new major QTLs of KL into a KASP marker as described previously (Liu et al., 2020). Ninety lines randomly selected from the two validation populations (SSY and MTL4SY), respectively, were used to do genotyping using this KASP marker. The reaction system included 0.75 μL DNA, 2.85 μL deionized water, 5 μL SsoFast EvaGreen mixture (Bio-Rad, Hercules, CA, United States), and 1.4 μL primers (*KASP-AX-109379070F*: GAAGGTGACCAAGTTCATGCTTGATTTCATGTGATAGCA CC, *AX-109379070H*: GAAGGTCGGAGTCAACGGATTTGAT TTCATGTGATAGCACT, *AX-109379070R*: ACCTCCCAAAA ATCGAGGTA). The whole process was carried out on real-time PCR (BioRad, CFX-96) system. The lines were divided into two categories based on the genotype of this KASP marker: (1) lines with homozygous alleles from either of S849-8 and MTL4-5-3 and (2) lines with homozygous alleles from SY95-71.

For three variety panels genotyped by Wheat55K or Wheat660K SNP array in which the flanking marker *AX-109379070* was included, the lines were also divided into two categories in each population based on the genotyping results. The BLUP values of KL data from all environments for CWLs and CWCs and average value for SWCs were used to analyze the significant differences between the two categories. Differences between the two categories were analyzed using Student *t*-test (*P* < 0.05).

## Results

### Phenotypic Evaluation

**FIGURE 2:**
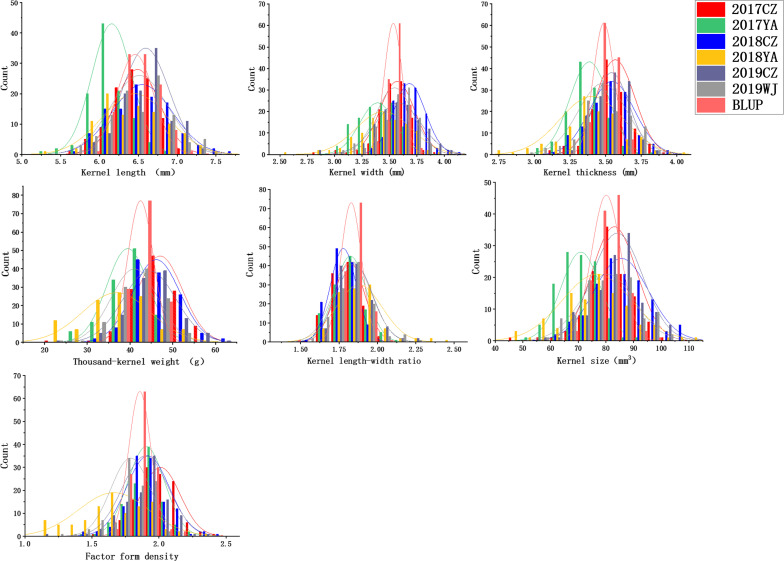
Frequency distributions for kernel length (KL), kernel width (KW), kernel thickness (KT), thousand-kernel weight (TKW), kernel length–width ratio (LWR), kernel size (KS), and factor form density (FFD) in different environments.

Descriptive statistics for KS and kernel weight in the 2SY population and two parents are presented in Table 1. The KL, KW, KT, TKW, LWR, KS, and FFD of 20828 were significantly higher than SY95-71, except for individual ecological locations (*P* < 0.05, Figure 1 and Table 1). Phenotypic data for the seven kernel traits in six environments and the BLUP dataset showed a continuous and normal distribution, indicating they were quantitatively inherited (Figure 2).

**TABLE 1:** Phenotype of the parents and RILs in this study.

Trait	Environment	Parents		RIL					
		20828	SY95-71	Min	Max	Mean	SD	CV	*H*^2^
KL (mm)	2017YA	6.10^b^	5.40	5.38	6.86	6.16	0.27	0.04	0.79
	2017CZ	6.20^b^	5.83	5.63	7.18	6.50	0.29	0.05	
	2018YA	6.14	6.29	5.22	7.58	6.47	0.42	0.07	
	2018CZ	6.52^b^	6.08	5.58	7.69	6.56	0.39	0.06	
	2019CZ	6.70^b^	6.25	5.72	7.39	6.61	0.31	0.05	
	2019WJ	6.36^b^	5.88	5.58	7.40	6.52	0.38	0.06	
	BLUP	6.35	6.01	5.80	7.11	6.47	0.24	0.04	
KW (mm)	2017YA	3.68^b^	3.27	3.06	3.69	3.39	0.16	0.05	0.54
	2017CZ	3.57	3.60	2.82	3.93	3.57	0.16	0.05	
	2018YA	3.77^b^	3.48	2.51	4.03	3.45	0.25	0.07	
	2018CZ	3.76^a^	3.69	3.31	4.10	3.68	0.15	0.04	
	2019CZ	3.70^b^	3.52	2.82	4.03	3.58	0.21	0.06	
	2019WJ	3.69^b^	3.51	3.05	3.87	3.55	0.17	0.05	
	BLUP	3.65	3.52	3.33	3.78	3.54	0.08	0.02	
KT (mm)	2017YA	3.61^a^	3.16	3.01	3.83	3.39	0.14	0.04	0.44
	2017CZ	3.48^b^	3.45	3.14	3.92	3.57	0.14	0.04	
	2018YA	3.61^b^	3.16	2.79	4.02	3.40	0.23	0.07	
	2018CZ	3.74^b^	3.47	3.15	3.99	3.54	0.16	0.05	
	2019CZ	3.61	3.66	3.21	3.87	3.54	0.13	0.04	
	2019WJ	3.67^b^	3.40	2.95	3.87	3.48	0.17	0.05	
	BLUP	3.59	3.46	3.33	3.67	3.49	0.07	0.02	
TKW (g)	2017YA	48.50^b^	31.00	25.33	49.00	39.47	4.92	0.13	0.60
	2017CZ	55.00^b^	43.29	24.00	57.33	47.04	5.26	0.11	
	2018YA	46.00^b^	40.00	20.00	56.00	36.46	8.37	0.23	
	2018CZ	58.00^a^	44.00	32.00	62.00	46.15	5.54	0.12	
	2019CZ	51.00^b^	37.33	23.33	60.00	44.56	6.19	0.14	
	2019WJ	48.67^b^	38	22.67	52.67	41.04	5.45	0.13	
	BLUP	49.04	39.81	36.04	49.98	42.46	2.99	0.07	
LWR	2017YA	1.66^b^	1.61	1.60	2.13	1.82	0.38	0.21	0.56
	2017CZ	1.74^a^	1.62	1.60	2.05	1.82	0.38	0.21	
	2018YA	1.63	1.70	1.62	2.49	1.88	0.34	0.18	
	2018CZ	1.74^a^	1.65	1.64	1.92	1.78	0.29	0.16	
	2019CZ	1.80^a^	1.77	1.63	2.28	1.85	0.31	0.17	
	2019WJ	1.72^b^	1.67	1.53	2.23	1.83	0.45	0.25	
	BLUP	1.74	1.72	1.48	2.05	1.83	0.07	0.04	
KS (mm^3^)	2017YA	80.98^a^	55.78	52.34	90.14	70.99	22.69	0.32	0.67
	2017CZ	76.87^b^	74.55	49.79	101.11	83.18	27.12	0.33	
	2018YA	85.63^a^	76.02	45.11	110.54	76.70	26.40	0.34	
	2018CZ	91.40^a^	77.80	61.83	109.40	85.57	19.36	0.23	
	2019CZ	89.60^a^	80.47	58.93	104.54	84.09	19.94	0.24	
	2019WJ	86.02^a^	70.00	55.89	102.00	80.64	26.27	0.33	
	BLUP	83.17	73.25	65.25	93.57	79.89	5.31	0.07	
FFD	2017CZ	2.49^a^	2.14	1.51	2.48	2.02	0.15	0.07	0.41
	2017YA	2.17^a^	1.76	1.50	2.26	1.89	0.14	0.07	
	2018CZ	2.38^a^	1.96	1.43	2.41	1.91	0.17	0.09	
	2018YA	2.03^a^	1.83	1.11	2.37	1.62	0.25	0.15	
	2019CZ	2.13^a^	1.67	1.17	2.32	1.88	0.18	0.10	
	2019WJ	2.09^a^	1.85	1.28	2.07	1.79	0.15	0.08	
	BLUP	2.12	1.88	1.62	2.14	1.85	0.08	0.04	

*KL, kernel length (mm); KW, kernel width (mm); KT, kernel thickness (mm); TKW, thousand-kernel weight (g); LWR, kernel length–width ratio; KS, kernel size; FFD, factor form density; RIL, recombinant inbred lines; SD, standard deviation; CV, variation coefficient; H^2^, the broad-sense heritability; BLUP, best linear unbiased prediction; WJ, Wenjiang; CZ, Chongzhou; YA, Ya’an. ^a^Difference is significant at the 0.05 level. ^b^ Difference is significant at the 0.01 level.*

In all the environments, KL ranges from 5.22 to 7.69 mm, KW from 2.51 to 4.10 mm, KT from 2.79 to 4.02 mm, TKW from 20.00 to 62.00 g, LWR from 1.48 to 2.49, KS from 45.11 to 110.54 mm^3^, and FFD from 1.11 to 2.48 (Table 1 and Figure 2). The *H*^2^ of KL, KW, KT, TKW, LWR, KS, and FFD were 0.79, 0.54, 0.44, 0.60, 0.56, 0.67, and 0.41, respectively, suggesting that KL was less affected by environmental factors than other kernel traits (Table 1). The variation coefficient of a given trait was similar in different environments, and the phenotypic data of all seven traits showed distinct bidirectional transgressive segregation in all the environments, indicating that favorable alleles may exist in both parents and reassociated in some offspring (Table 1).

### Correlation Analysis

Correlation analysis of kernel traits in different environments showed that only KL and TKW were correlated between all different environments (Supplementary Table 1). For KW, KT, KS, LWR, and FFD, the correlation was significant for most of the environments (Supplementary Table 3).

Positive correlations were detected among KL, KW, KT, and TKW, with correlation coefficients ranging from 0.354 to 0.756 (*P* < 0.01, Supplementary Table 4). LWR was negatively correlated with KW and FFD, but positively correlated with KL (*P* < 0.01). KS had a positive correlation with the other six kernel traits except for LWR (*P* < 0.01). FFD was positively correlated with KW, KT, TKW, and KS (*P* < 0.01, Supplementary Table 4).

The correlation analysis between the seven kernel traits and other agronomic traits measured earlier showed that KW, KT, TKW, KS, and FFD were positively related to PH (*P* < 0.01); KL and KW were negatively correlated with PTN (*P* < 0.05); KL and KS were positively correlated with FLL (*P* < 0.05); FLW was positively correlated with KW, TKW, and KS (*P* < 0.05); SL was positively correlated with all seven kernel traits (*P* < 0.05), and SD was negatively correlated with KL, KT, TKW, LWR, and KS (*P* < 0.05 Supplementary Table 5).

### QTL Mapping of Kernel Traits

**FIGURE 3:**
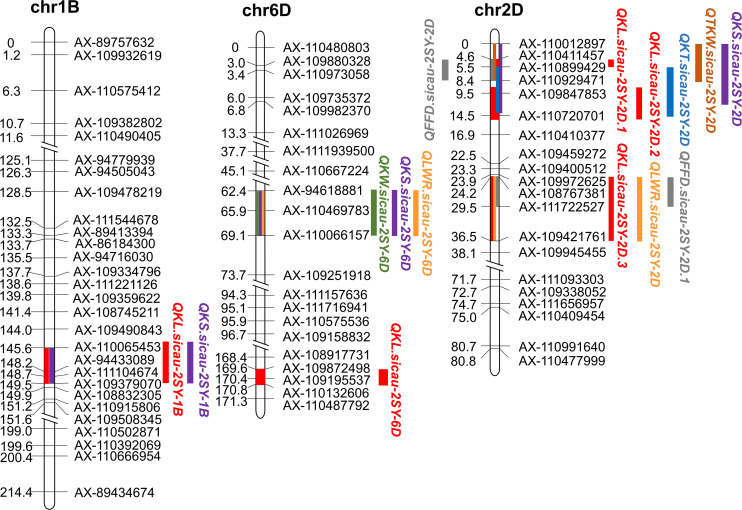
Fifteen major or minor QTLs for kernel traits in the genetic map. Red color represents QTL conferring kernel length (KL), green color represents QTL conferring kernel width (KW), blue color represents QTL conferring kernel thickness (KT), brown color represents QTL conferring thousand-kernel weight (TKW), orange color represents QTL conferring kernel length–width ratio (TKW), purple color represents QTL conferring kernel size (KS), and gray color represents QTL conferring factor form density (FFD).

QTL analysis results of seven kernel-related traits in the 2SY population are shown in Table 2. A total of 65 putative QTLs related to kernel traits were detected including 14 for KL, 4 for KW, 11 for KT, 11 for TKW, 9 for LWR, 12 for KS, and 4 for FFD (Supplementary Table 6). Among them, nine QTLs can be detected in at least three different environments in the 2SY population (Table 2). They were located on 1B (two QTLs), 2D (four QTLs), and 6D (three QTLs) chromosomes (Table 2 and Figure 3). *QKL.sicau-2SY-1B* is the major locus and can be detected in all environments and with BLUP dataset, explaining 9.48% to 25.23% of phenotypic variation. The positive allele at *QKL.sicau-2SY-1B* was derived from SY95-71 (Table 2). Other three QTLs for KL (*QKL.sicau-2SY-2D.1*, *QKL.sicau-2SY-2D.2*, and *QKL.sicau-2SY-2D.3*, respectively) were detected on 2D chromosome and explained the phenotypic variation of 13.36–23.24% (Supplementary Table 6). Although they also have a relatively high value of phenotypic variation, they were unstable and detected only in less than three environments. *QKL.sicau-2SY-6D*, a minor QTL for KL, explaining 5.33–7.07% of the phenotypic variation was detected, and the positive alleles of these four minor QTLs were all derived from 20828 (Supplementary Table 6).

**TABLE 2:** Quantitative trait loci (QTLs) for kernel traits and plant height (PH) and anthesis date (AD) detected in more than three environments in the “20828” × “SY95-71” population.

Trait	QTLs	Environment	Chromosome arm	Interval (cM)	Left marker	Right marker	LOD	PVE (%)	Add
KL	*QKL.sicau-2SY-1B*	2017CZ	1BL	148.5–149.5	*AX-111104674*	*AX-109379070*	6.02	13.67	−0.12
		2017YA	1BL	143.5–148.5	*AX-110065453*	*AX-94433089*	2.53	9.48	−0.08
		2018CZ	1BL	145.5–148.5	*AX-110065453*	*AX-94433089*	9.85	21.13	−0.20
		2018YA	1BL	143.5–148.5	*AX-110065453*	*AX-94433089*	6.97	10.52	−0.17
		2019CZ	1BL	144.5–147.5	*AX-110065453*	*AX-94433089*	8.38	14.77	−0.14
		2019WJ	1BL	148.5–149.5	*AX-111104674*	*AX-109379070*	16.88	25.23	−0.24
		BLUP	1BL	144.5–147.5	*AX-110065453*	*AX-94433089*	16.75	23.74	−0.14
KW	*QKW.sicau-2SY-6D*	2017CZ	6DS	64.5–68.5	*AX-94618881*	*AX-110469783*	3.07	11.28	0.05
		2018CZ	6DS	64.5–68.5	*AX-110469783*	*AX-110066157*	4.27	10.31	0.05
		2018YA	6DS	64.5–69.5	*AX-94618881*	*AX-110066157*	3.93	16.26	0.10
		2019CZ	6DS	65.5–67.5	*AX-110469783*	*AX-110066157*	14.55	39.50	0.13
		2019WJ	6DS	64.5–69.5	*AX-110469783*	*AX-110066157*	3.72	13.93	0.06
		BLUP	6DS	64.5–67.5	*AX-110469783*	*AX-110066157*	10.88	32.77	0.05
KT	*QKT.sicau-2SY-2D*	2017CZ	2DL	8.5–13.5	*AX-110929471*	*AX-109847853*	3.03	11.05	0.04
		2018CZ	2DL	5.5–9.5	*AX-110899429*	*AX-110929471*	13.23	31.72	0.09
		2018YA	2DL	8.5–12.5	*AX-109847853*	*AX-110720701*	8.71	29.62	0.12
		2019CZ	2DL	4.5–8.5	*AX-110899429*	*AX-110929471*	9.75	12.84	0.05
		2019WJ	2DL	6.5–9.5	*AX-110899429*	*AX-110929471*	11.52	29.29	0.09
		BLUP	2DL	8.5–12.5	*AX-109847853*	*AX-110720701*	14.71	41.26	0.05
TKW	*QTKW.sicau-2SY-2D*	2017YA	2DL	0–5.5	*AX-110012897*	*AX-110411457*	4.29	11.67	1.89
		2017CZ	2DL	4.5–7.5	*AX-110411457*	*AX-110899429*	4.05	11.50	1.72
		2018CZ	2DL	1.5–5.5	*AX-110012897*	*AX-110411457*	5.66	16.51	2.19
		2019CZ	2DL	1.5–5.5	*AX-110411457*	*AX-110899429*	8.32	22.98	3.20
		2019WJ	2DL	5.5–9.5	*AX-110899429*	*AX-110929471*	12.08	25.63	2.75
		BLUP	2DL	1.5–5.5	*AX-110012897*	*AX-110411457*	9.80	30.61	1.64
LWR	*QLWR.sicau-2SY-6D*	2017CZ	6DS	64.5–68.5	*AX-94618881*	*AX-110469783*	4.07	11.28	−0.05
		2018YA	6DS	64.5–68.5	*AX-110469783*	*AX-110066157*	2.54	11.63	−0.05
		2019CZ	6DS	64.5–66.5	*AX-94618881*	*AX-110469783*	14.25	22.76	−0.06
		BLUP	6DS	64.5–67.5	*AX-110469783*	*AX-110066157*	3.98	9.92	−0.02
KS	*QKS.sicau-2SY-1B*	2017CZ	1BL	144.5–148.5	*AX-110065453*	*AX-94433089*	3.17	7.95	−2.00
		2018CZ	1BL	145.5–148.5	*AX-110065453*	*AX-94433089*	5.22	11.28	−3.67
		2019WJ	1BL	148.5–149.5	*AX-111104674*	*AX-109379070*	4.11	9.01	−3.22
		BLUP	1BL	145.5–148.5	*AX-110065453*	*AX-94433089*	7.37	13.86	−1.98
	*QKS.sicau-2SY-2D*	2017CZ	2DL	8.5–13.5	*AX-109847853*	*AX-110720701*	6.00	3.92	2.82
		2018CZ	2DL	0–5.5	*AX-110012897*	*AX-110411457*	8.25	18.80	4.73
		2019CZ	2DL	0–5.5	*AX-110411457*	*AX-110899429*	7.35	19.14	3.91
		2019WJ	2DL	5.5–9.5	*AX-110899429*	*AX-110929471*	7.66	18.11	4.53
		BLUP	2DL	1.5–5.5	*AX-110411457*	*AX-110899429*	13.47	27.78	2.80
	*QKS.sicau-2SY-6D*	2018YA	6DS	64.5–65.5	*AX-110469783*	*AX-94618881*	2.68	6.78	3.45
		2019CZ	6DS	65.5–68.5	*AX-110469783*	*AX-110066157*	6.27	17.35	3.78
		2019WJ	6DS	64.5–69.5	*AX-110469783*	*AX-110066157*	4.03	8.88	3.25
		BLUP	6DS	64.5–68.5	*AX-110469783*	*AX-110066157*	5.31	9.39	1.64
FFD	*QFFD.sicau-2SY-2D*	2017YA	2DL	4.5–8.5	*AX-110411457*	*AX-110899429*	2.95	12.22	0.05
		2017CZ	2DL	4.5–8.5	*AX-110899429*	*AX-110929471*	2.59	10.11	0.05
		2018YA	2DL	4.5–8.5	*AX-110899429*	*AX-110899429*	3.73	15.76	0.11
		2019WJ	2DL	0–5.5	*AX-110411457*	*AX-110899429*	3.08	14.37	0.05
		BLUP	2DL	4.5–8.5	*AX-110899429*	*AX-110929471*	5.74	17.25	0.04
PH*	*PH.sicau-2SY-2D*	2017YA	2DS	10.5–29.5	*AX-86163393*	*AX-109785183*	2.88	8.61	5.05
		2017CZ	2DS	1.5–19.5	*AX-86163393*	*AX-109785183*	5.08	7.18	3.91
		2017WJ	2DS	9.5–24.5	*AX-109836946*	*AX-86163393*	3.26	6.23	3.92
		2018YA	2DS	2.5–13.5	*AX-86163393*	*AX-109785183*	2.75	11.03	3.80
		2018CZ	2DS	0–15.5	*AX-109836946*	*AX-86163393*	5.33	12.74	4.03
		2018WJ	2DS	2.5–20.5	*AX-86163393*	*AX-109785183*	6.82	13.48	5.08
		BLUP	2DS	2.5–13.5	*AX-109836946*	*AX-86163393*	6.81	13.38	3.95
	*PH.sicau-2SY-4B*	2017YA	4BS	1.5–5.5	*AX-111620391*	*AX-109110130*	4.98	12.86	−6.29
		2017CZ	4BS	3.5–5.5	*AX-111620391*	*AX-109110130*	18.97	40.58	−9.36
		2017WJ	4BS	2.5–5.5	*AX-111620391*	*AX-109110130*	15.33	34.09	−9.39
		2018WJ	4BS	2.5–5.5	*AX-111620391*	*AX-109110130*	5.83	12.60	−4.97
AD*	*AD.sicau-2SY-2D*	2017CZ	2DS	9.5–18.5	*AX-86163393*	*AX-109785183*	5.98	17.17	1.94
		2017WJ	2DS	6.5–20.5	*AX-86163393*	*AX-109785183*	7.62	21.23	2.99
		2018CZ	2DS	4.5–17.5	*AX-86163393*	*AX-109785183*	4.20	12.65	1.37
		2018WJ	2DS	9.5–19.5	*AX-86163393*	*AX-109785183*	8.79	19.14	1.64

LOD, logarithm of odds; PVE, phenotype variance explained; Add, additive effect of a QTL; positive values, alleles from 20828 are increasing the trait scores, negative values, alleles from SY95-71 are increasing the scores. KL, kernel length (cm); KW, kernel width (cm); KT, kernel thickness (cm); TKW, thousand-kernel weight (cm); LWR, kernel length–width ratio; KS, kernel size; FFD, factor form density; BLUP, best linear unbiased prediction; WJ, Wenjiang; CZ, Chongzhou; YA, Ya’an. *The data was from Liu et al. (2020). The QTL linkage group of PH and AD was located in the 2DS, and kernel-related traits were located in the 2DL.

Major QTLs for KW, KT, and TKW were detected on chromosomes 6D, 2D, and 2D, respectively. *QKW.sicau-2SY-6D* was detected in five different environments and with BLUP dataset, explaining the phenotypic variation of 9.36–39.50%. *QKT.sicau-2SY-2D* was detected in five different environments and with BLUP dataset, which explains 11.05–41.26% of the phenotypic variation. Meanwhile, *QTKW.sicau-2SY-2D*, the major QTL controlling TKW was also detected on 2D chromosome in five different environments and with BLUP dataset and accounted for 11.50–30.61% of the phenotypic variation. The positive alleles of these three major QTLs for KW, KT, and TKW were all contributed by 20828 (Table 2).

Major QTL *QLWR.sicau-2SY-6D* for LWR was detected in three different environments with BLUP dataset, explaining 9.92–22.76% of the phenotypic variance (Table 2). And the minor QTL *QLWR.sicau-2SY-2D* explained 8.24–9.70% of the phenotypic variance in a single environment (Supplementary Table 6). The positive alleles for these two QTL were contributed by SY95-71 and 20828, respectively (Supplementary Table 6).

Major QTLs for KS (*QKS.sicau-2SY-1B*, *QKS.sicau-2SY-2D*, and *QKS.sicau-2SY-6D*, respectively) were detected in four to five different environments and with BLUP dataset explaining 3.92–27.78% of the phenotypic variance (Table 2). The positive allele of the first one was derived from SY95-71 and the last two from 20828 (Table 2).

Major QTL *QFFD.sicau-2SY-2D* for FFD was detected in five different environments and with BLUP dataset, explaining 10.11–17.25% of the phenotypic variance (Table 2). The minor QTL *QFFD.sicau-2SY-2D.1* was detected in two different environments and explained 10.19–18.79% of the phenotypic variance (Supplementary Table 6). These two QTLs’ positive alleles were both contributed by 20828 (Supplementary Table 6).

**FIGURE 4:**
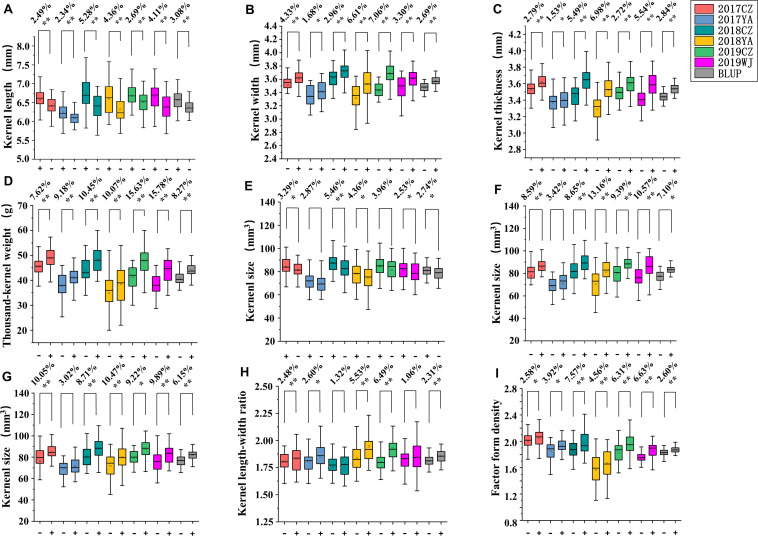
Genetic effects of *QKL.sicau-2SY-1B* **(A)**, *QKW.sicau-2SY-6D* **(B)**, *QKT.sicau-2SY-2D* **(C)**, *QTKW.sicau-2SY-2D* **(D)**, *QKS.sicau-2SY-1B* **(E)**, *QKS.sicau-2SY-2D* **(F)**, *QKS.sicau-2SY-6D* **(G)**, *QLWR.sicau-2SY-6D* **(H)**, and *QFFD.sicau-2SY-2D* **(I)** in 2SY population. Different colors represent different environments and BLUP. In **(A,E)** “+” and “-” represent homozygous lines carrying “SY95-71” and “20828” alleles, respectively; In **(B–I)** “+” and “-” represent homozygous lines carrying “20828” and “SY95-71” alleles, respectively. **Significance at the 0.01 probability level; *significance at the 0.05 probability level.

According to the genotypes of flanking markers for major QTLs of kernel-related traits, lines with homozygous alleles for a given QTL were obtained in 2SY population. For *QKL.sicau-2SY-1B* and *QKS.sicau-2SY-1B*, the phenotypic values of lines carrying SY95-71 alleles were significantly higher than those containing 20828 alleles in different environments and with BLUP dataset except for *QKS.sicau-2SY-1B* in 2017YA (*P* < 0.05, Figure 4). In major QTLs for KW, TKW, LWR, KS, and FFD (*QKW.sicau-2SY-6D*, *QTKW.sicau-2SY-2D*, *QLWR.sicau-2SY-6D*, *QKS.sicau-2SY-2D*, *QKS.sicau-2SY-6D*, and *QFFD.sicau-2SY-2D*, respectively), the phenotypic values of lines carrying 20828 alleles were significantly higher than those with SY95-71 alleles in different environments and BLUP dataset except for *QKS.sicau-2SY-2D* in 2017YA, *QKS.sicau-2SY-6D* in 2017YA, and *QLWR.sicau-2SY-6D* in 2018CZ (*P* < 0.05, Figure 4).

### Effects of *QKS.sicau-2SY-1B*, *QKS.sicau-2SY-2D*, and *QKS.sicau-2SY-6D* on KS in the 2SY Population

**FIGURE 5:**
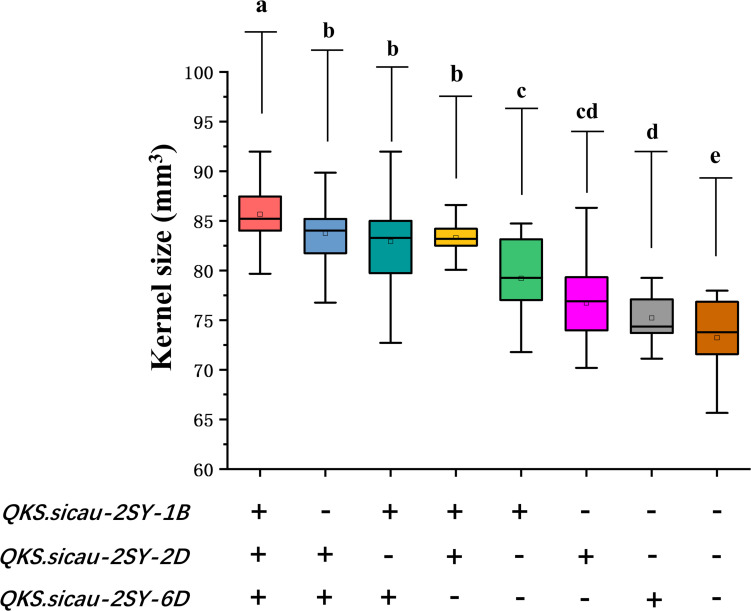
Pyramiding analysis of *QKS.sicau-2SY-1B*, *QKS.sicau-2SY-2D*, and *QKS.sicau-2SY-6D* for KS; “+” and “-” represent lines with and without the positive alleles of the target quantitative trait loci (QTLs) based on the flanking markers of the corresponding QTL, respectively. Letters a, b, c, d, and e represent significant difference.

The effects of the positive alleles at the three QTL for KS (*QKS.sicau-2SY-1B*, *QKS.sicau-2SY-2D*, and *QKS.sicau-2SY-6D*) were analyzed (Figure 5, *P* < 0.05). As expected, compared with those without alleles increasing KS, RILs carrying only one, two, and three alleles significantly increased KS, respectively. Lines with three alleles significantly increased KS compared with those with two and a single one, respectively. No significant differences were detected among three different combinations with any two alleles. Among the three alleles, *QKS.sicau-2SY-1B* has a larger effect on KS. Taken together, three alleles have the largest effect on increasing KS, followed by those with two, and those with a single one have the smallest effect (Figure 5, *P* < 0.05).

### The Factorial ANOVA for Major QTLs of Each Kernel Trait

We performed factorial analysis and the two-way interactions for the most significant markers based on the LOD values from the five QTLs on chromosomes 1B, 2D, and 6D of seven kernel-related traits to determine the contribution rate of a single marker to each trait (Supplementary Table 7).

The factorial ANOVA containing the major and minor QTLs and all possible two-way interactions explained a relative larger portion of the variation in KL (55.0%), KW (37.4%), KT (59.1%), TKW (44.0%), LWR (73.0%), KS (58.8%), and FFD (44.6%), respectively. Compared with QTLs on chromosome arm 6DL, the QTL on 1BL explained a larger proportion of variation for KL (23.6%), LWR (20.6%), KS (18.1%), and FFD (3.9%), but no variation was explained for other kernel traits (KW, KT, and TKW). The QTLs on chromosome arms 6DS and 2DL-1 explained the largest proportion of the variation for KW (13.9%), KT (18.4%), TKW (13.3%), and FFD (21.4%), respectively. Meanwhile, the 2DL-2 also explained a proportion variation for KL (6.7 %), LWR (11.3 %), and FFD (3.9%). Based on the QTL interaction analysis, we detected a total of seven pairs of QTL with interaction, and they explained the variation of less than 5% for kernel-related traits (KW, KT, TKW, LWR, and KS; *P* < 0.05). Additionally, no QTL interaction was detected for KL and FFD (Supplementary Table 7).

### The Multienvironment Analysis

A total of 609 QTLs related to kernel traits were detected in the multienvironment analysis (Supplementary Table 8). Among them, 51 were identical with those detected in individual environment QTL mapping. Some of these QTLs can be detected only in a single environment, indicating that they are greatly affected by the environments and cannot be stably expressed. Meanwhile, *QKL.sicau-2SY-1B*, *QKW.sicau-2SY-6D*, *QKT.sicau-2SY-2D, QTKW.sicau-2SY-2D*, *QLWR.sicau-2SY-6D*, *QKS.sicau-2SY-1B*, *QKS.sicau-2SY-2D*, *QKS.sicau-2SY-6D*, and *QFFD.sicau-2SY-2D* were all detected, further indicating that they were major and stable.

### Colocalized Regions and Physical Intervals for Mapped QTL

QTL mapping revealed that the major QTLs for KL and KS (*QKL.sicau-2SY-1B* and *QKS.sicau-2SY-1B*) were colocalized between makers *AX-110065453* and *AX-109379070* at 143.5-149.5 cM of the 1B chromosome (Table 2). Major QTLs for KW, LWR, and KS (*QKW.sicau-2SY-6D*, *QLWR.sicau-2SY-6D*, and *QKS.sicau-2SY-6D*, respectively) were colocalized between makers *AX-94618881* and *AX-110066157* at 64.5 to 69.5 cM of the 6D chromosome (Table 2). Major QTLs for KT, TKW, KS, and FFD (*QKT.sicau-2SY-2D*, *QTKW.sicau-2SY-2D*, *QKS.sicau-2SY-2D*, *QFFD.sicau-2SY-2D*, respectively), and the minor QTLs for KL (*QKL.sicau-2SY-2D.1* and *QKL.sicau-2SY-2D.2*) were colocalized between makers *AX-110012897* and *AX-110720701* at 0 to 15.5 cM of the 2D chromosome (Figure 3, and Table 2).

**FIGURE 6:**
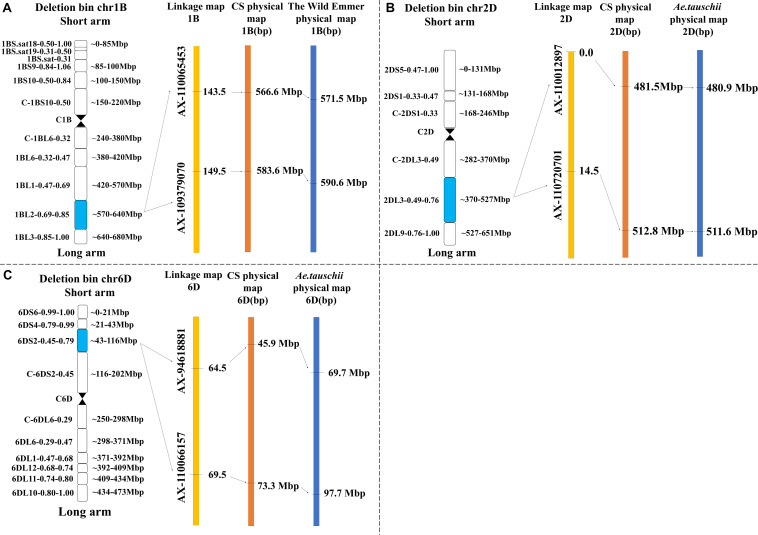
Genetic and physical maps of the four major QTLs in the target interval on Chinese spring, *T. turgidum* and *A. tauschii* reference genomes (“**A**” represents *QKL.sicau-2SY-1B* and *QKS.sicau-2SY-1B*; “**B**” represents *QKT.sicau-2SY-2D*, *QTKW.sicau-2SY-2D*, *QKS.sicau-2SY-2D*, and *QFFD.sicau-2SY-2D*; and “**C**” represents *QKW.sicau-2SY-6D*, *QLWR.sicau-2SY-6D*, and *QKS.sicau-2SY-6D*).

Besides, the major QTLs of seven kernel-related traits were located at 566.6 to 583.6 Mbp in the deletion bin 1BL2 0.69 to 0.85 on chromosome 1BL, 45.9 to 73.3 Mbp in the deletion bin 6DS2 0.45 to 0.79 on chromosome 6DS, and 481.5 to 512.8 Mbp in the deletion bin 2DL3 0.49 to 0.76 on chromosome 2DL of “Chinese Spring” (Figure 6). Meanwhile, we also identified the physical intervals of these major QTLs on wild emmer and *Aegilops tauschii* reference genomes using flanking markers’ sequences and predicted genes in these intervals (Supplementary Table 9).

### Validation of the Major and Novel KL QTL *QKL.sicau-2SY-1B*

**FIGURE 7:**
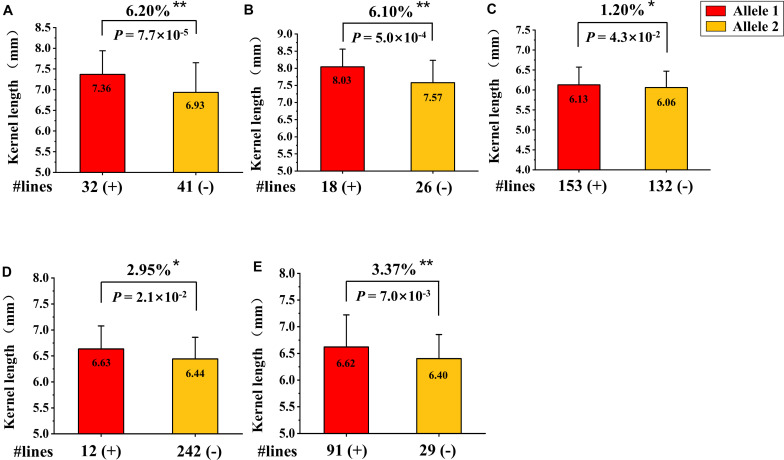
Validation of *QKL.sicau-2SY-1B* in two different genetic populations and three variety panels. **(A,B)** Effects of *QKL.sicau-2SY-1B* in two validation populations of SSY **(A)** and MTL4SY **(B)** by fluorescence PCR typing results of the Kompetitive Allele Specific PCR (KASP) marker *KASP- AX-109379070*, respectively. **(C–E)** Effects of *QKL.sicau-2SY-1B* based on the flanking marker *AX-109379070* in 300 Chinese wheat cultivars (CWC), 272 Chinese wheat landraces (CWL), and 165 Sichuan wheat cultivars (SWC), respectively. Allele1 (+) and Allele2 (-) represent lines with and without the positive alleles of the target quantitative trait loci (QTL) based on the flanking marker. **Significance at the 0.01 probability level; *significance at the 0.05 probability level.

Based on the QTL mapping results of KL, a new KASP marker (*KASP- AX-109379070*) closely linked to *QKL.sicau-2SY-1B* was developed. We detected the effect of *QKL.sicau-2SY-1B* in two verified populations with different genetic backgrounds (SSY and MTL4SY) using this marker. The marker *KASP- AX-109379070* was able to detect polymorphism between parent SY95-71 and the other two parents S849-8 and MTL4-5-3. The homozygous alleles were successfully divided using *KASP-AX-109379070* in SSY and MTL4SY populations (Figures 7A,B). Based on the genotyping results, 32 and 41 lines carrying SY95-71 and S849-8 alleles, respectively, were detected in SSY population, and 18 and 26 lines carrying SY95-71 alleles and MTL4-5-3 alleles, respectively, were detected in MTL4SY population. The Student *t*-test detected significant differences (*P* < 0.01) between two groups with different alleles for both two populations. The lines that carried the allele from SY95-71 significantly increased KL by 6.2 and 6.1%, respectively, compared with those without this allele in these two populations with different backgrounds (Figures 7A,B).

Additionally, based on the genotyping results of the flanking marker *AX-109379070* for KL, we validated the effect of *QKL.sicau-2SY-1B* in three variety panels. After excluding heterozygous lines, the lines in each panel were divided into two groups. The groups carrying alleles from SY95-71 significantly increased KL by 1.2, 2.95, and 3.37% in three variety panels, respectively (*P* < 0.05) (Figures 7C–E).

## Discussion

### Contribution of D Genome to Kernel-Related Traits

Three major QTLs for KW, LWR, and KS and a minor QTL for KL were identified on the 6D chromosome, whereas four major QTLs for KT, TKW, KS, and FFD and a minor QTL for KL, LWR, and FFD were identified on 2D chromosome. Zhao et al. (2017) constructed the first genome-based gene/QTL map for *A. tauschii* and observed that numerous genes or QTLs were detected on the 2D and 7D chromosomes. Many previous studies also detected kernel-related traits on 2D chromosome (Ammiraju et al., 2001; Huang et al., 2003, 2006, 2015; Cuthbert et al., 2008; Zhang et al., 2015). Our results together with those reported previously suggested that 2D and 6D chromosomes may have played a positive contribution to wheat kernel improvement. Thus, *A. tauschii* as the important genetic resource and donor of D genome should be further utilized through synthetic hexaploidy wheat given its important role in regulating kernel size (Okamoto et al., 2013; Arora et al., 2017).

### QTL Clusters on 1BL, 2DL, and 6DS Chromosomes

In wheat, QTLs for quantitative inheritance are usually located in gene-rich regions, and they are usually mapped to the same genomic region to form clusters (Mccartney et al., 2005; Li et al., 2007). In previous studies, some pleiotropic QTLs related to kernel traits were reported (Sun et al., 2009; Ramya et al., 2010; Cheng et al., 2015; Li et al., 2015; Zhang et al., 2015; Cabral et al., 2018; Kumari et al., 2018; Ma et al., 2019c). In this study, the major QTLs controlling KL and KS were located within 4 cM on the long arm of chromosome 1BL; the major QTLs controlling KT, TKW, KS, and FFD were located within 14 cM on the long arm of chromosome 2DL, and the major QTLs controlling KW, LWR, and KS were located within 5 cM on the short arm of chromosome 6DS. These overlapping QTL indicated that either a single QTL has a pleiotropic effect, or the genomic region associated with these QTL has a set of pertinent genes related to these traits. Therefore, the above three QTL clusters are of great value in marker-assisted breeding and should be paid more attention to in wheat genetics and improvement.

### Favorable Alleles at Major QTL Are Inherited From Low-Value Parents

Previous studies reported that additive effects of a few QTL can be detected from lower-value parents. For example, the KW and TKW of the parent Shannong483 were significantly higher than the parent Chuan35050, but the positive alleles at *QKw.sdau-6A* and *QTkw.sdau-6A* were detected from the latter (Sun et al., 2009). Another example is from the study reported by Mohler et al. (2016). The KL of the parent Pamier was larger, and the KW was smaller than the other parent Format. However, a major QTL controlling KL, the increased allele of which was inherited from the shorter kernel parent Format, was detected between makers *TaCwi-A1* and *BS00090569_51* on chromosome 2A, and a major QTL controlling KW, the positive allele of which was contributed by the narrow-kernel parent Pamier, was detected between makers *BS00010625_51* and *IWB7258* on chromosome 1B (Mohler et al., 2016).

Here, although the KL value of SY95-71 was significantly lower than 20828, we identified a major QTL *QKL.sicau-2SY-1B* for KL, the positive allele from which was contributed by the former. Furthermore, the effect of this favorable allele (i.e., SY95-71 allele) at *QKL.sicau-2SY-1B* was successfully verified in two different RIL populations and three variety panels. These results further suggested that genetic recombination between two genotypes provides a chance of producing an offspring carrying a single locus without other inhibiting loci that can exhibit an obvious corresponding phenotype. We should also know that some major QTLs, the positive allele of which is from higher-value parents, might not be detected because of the low coverage of SNP arrays distributed in the centromeres of chromosomes (Liu et al., 2020). Thus, in order to solve the defect of low enrichment of SNP in some regions of chromosome, scanning the whole genome using more mapped markers is necessary for accurate evaluation major QTLs for kernel-related traits.

### Comparison With Previous Studies

The comparison of physical regions showed that *QKT.sicau-2SY-2D*, *QTKW.sicau-2SY-2D*, *QKS.sicau-2SY-2D*, and *QFFD.sicau-2SY-2D* were colocated between 481.5 and 512.8 Mbp on chromosome 2DL of CS reference genome. These loci were overlapped with a QTL cluster for TKW, KW, and kernel area on 2D between 481.6 and 523.1 Mbp in previous research (Mohler et al., 2016), suggesting they were likely alleles (Supplementary Table 9). *QGwid.ccsu-6D.1* for KW and kernel surface area were located at 62.0 to 79.9 Mbp on 6DS chromosome (Tyagi et al., 2015), which was overlapped with the physical interval 45.9 to 73.3 Mbp of the cluster *QKW.sicau-2SY-6D*, *QLWR.sicau-2SY-6D*, and *QKS.sicau-2SY-6D* in this study (Supplementary Table 10). Therefore, there may be QTL clusters controlling kernel traits in the 45.9- to 79.9-Mbp region of 6DS chromosome. So far, there was no other QTL in the physical interval 566.6 to 583.6 Mbp on 1BL, indicating *QKL.sicau-2SY-1B* and *QKS.sicau-2SY-1B* are likely new QTLs (Supplementary Table 10). The physical location information of each major QTL is shown in Supplementary Table 11.

### Genes Located in the Intervals of the Three QTL Clusters

We attempted to predicate candidate genes for the identified QTLs conferring the kernel-related traits on chromosomes 1BL, 6DS, and 2DL based on the homology comparison results for CS with *Triticum turgidum* and *A. tauschii* reference genomes. Functional annotation results showed that the QTL cluster on 1BL between 566.6 to 583.6 Mbp on CS and 571.5 to 590.6 Mbp on *T. turgidum* physical regions contained 51 common predicated genes. *TraesCS1B01G351200* encodes proteins containing the VQ motif, and proteins with the same domain are reported to regulate endosperm growth and kernel size in model plant *A. thaliana* (Wang et al., 2010). *TraesCS1B01G338700* encodes a B3 domain–containing protein family and was reported to control endosperm development and kernel filling in maize *TraesCS1B01G338700* encodes a B3 domain–containing protein family, and the same proteins are found to control endosperm development and kernel filling in maize (Grimault et al., 2015). NHL domain–containing protein and carboxypeptidase encoded by *TraesCS1B01G349800* and *TraesCS1B01G345000* and functional proteins with these two domains regulate KS and kernel weight in rice and wheat (Chen et al., 2015; Ma et al., 2016). The QTL cluster on 6DS between 45.9 to 73.3 Mbp on CS and 69.7 to 97.7 Mbp on *A. tauschii* physical regions contained 186 common predicated genes. The BURP domain protein RD22 is encoded by *TraesCS6D01G081900* and *TraesCS6D01G082000*; these proteins with the same domain are related to the content of storage proteins and the composition of fatty acids in *A. thaliana* and ultimately affect the development of seed (Van Son et al., 2009). The cyclin-dependent kinase inhibitor is encoded by *TraesCS6D01G088500*; this kind of protein played an important role in the exit from the mitotic cell cycle during rice kernel formation (Barrôco et al., 2006). The QTL cluster on 2DL between 481.5 to 512.8 Mbp on CS and 480.9 to 511.6 Mbp on *A. tauschii* physical regions contained 147 common predicated genes. *TraesCS2D01G391600* encodes an expansin protein, a protein with this domain that interacts with E3 ubiquitin ligase and regulates kernel development after ubiquitin modification in rice (Choi et al., 2018). *TraesCS2D01G385000* encodes a kinesin-like protein; the gene encoding this kind of proteins has been reported to bind to the promoter of gibberellic acid (GA) biosynthesis gene and regulated cell elongation during panicle and seed development through GA biosynthesis in rice (Li et al., 2011). Interestingly, F-box family proteins were identified in all three QTL clusters. For some F-box proteins, it was involved in the nutrition and reproductive development of many plants, coding genes of which have a specific expression in flowering, spike, and seed stages and are important for the regulation of cellular protein degradation (Jain et al., 2007; van den Burg et al., 2008). These genes associated with cell expansion and plant reproductive development are the focus of our subsequent research.

### Relationship Between Kernel Traits and Other Agronomic Traits

In this study, positive correlations among the KL, KW, KT, TKW, and KS kernel traits were detected (Supplementary Table 4), and similar results have been previously reported (Sun et al., 2009; Gegas et al., 2010; Liu et al., 2010; Prashant et al., 2012; Zhang et al., 2015; Ma et al., 2019c; Xin et al., 2020), suggesting selection for higher TKW kernels might lead to direct selection for larger seeds in the breeding process (Ramya et al., 2010). In the analysis of other agronomic traits, KL and KW were negatively correlated with PTN (Supplementary Table 5). The analysis of QTL mapping interval in KW showed that there were indeed genes regulating tiller number. For example, *TraesCS6D01G082300* encodes a esterase/lipase/thioesterase-like protein, and the gene encoding this protein played a negative role in the regulation of PTN in rice (Liu et al., 2009; Supplementary Table 9). Therefore, the increase of tillers may be accompanied by a decline of the kernel-related traits. Zhang et al. (2015) and Ma et al. (2019c) also reached the same conclusion that PH was positively correlated with KW and TKW. We further genetically analyzed the relationship between the kernel-related traits and PH and AD. Reported QTLs for PH (32.7–46.8 Mbp) and AD (35.6–46.8 Mbp) on chromosome arm 2DS (Liu et al., 2020) were far away from the 2DL QTL cluster (Table 2). Similarly, the gene *early flowering 3* (*ELF3*), located at 685.6 Mbp on 1BL (Wang et al., 2016), was far away from the 1BL QTL cluster. These genes/QTLs that affect flowering and PH do not overlap with the *QKL.sicau-2SY-1B*. The longer SL may play a positive role in regulating development of kernel-related traits given that positive correlations were detected between SL and the seven kernel-related traits (Supplementary Table 5). Consistent with previous studies, SD was negatively correlated with KL and TKW (Wu et al., 2012; Liu et al., 2019). Therefore, increasing SL without changing SD may be a way to improve spike fertility and kernel yield (Sourdille et al., 2000). These correlations among these agronomic traits indicated that a reasonable control of the relationship between them can speed up the breeding process and increase the wheat yield.

## Conclusion

We identified nine stable and major QTLs for kernel-related traits including KL, KW, KT, TKW, LWR, KS, and FFD. These major QTLs formed three QTL clusters on 1BL, 2DL, and 6DS chromosomes. A novel QTL for KL was identified and validated in two RIL populations with different genetic backgrounds and three variety panels. Other major QTLs were previously detected and also identified in this study. We further analyzed and discussed the contribution and interaction of these kernel-related major QTLs. The predicted genes in the *QKL.sicau-2SY-1B* interval will be valuable for the subsequent fine mapping of candidate genes. The major QTLs for kernel traits identified and the developed KASP marker for *QKL.sicau-2SY-1B* may enhance the value of its use in wheat breeding.

## Data Availability Statement

The original contributions presented in the study are included in the article/Supplementary Material, further inquiries can be directed to the corresponding author/s.

## Author Contributions

XQ and JL performed the entire study and drafted this manuscript. XX did the phenotype measurement and data analysis. QX and HT did the field work and data analysis. YM, ZP, YL, JM, and YG collected and analyzed the data. QJ and YxL helped with the data analysis. GC, JW, and PQ did the QTL analysis and manuscript revision. AH and YW revised the manuscript. YZ discussed the results and revised the manuscript. XL guided the study and revised the manuscript. JM designed the experiments, guided the entire study, participated in data analysis, wrote and extensively revised this manuscript. All authors participated in the research and approved the final manuscript.

## Conflict of Interest

The authors declare that the research was conducted in the absence of any commercial or financial relationships that could be construed as a potential conflict of interest.

## Footnotes

1 http://en.wikipedia.org/wiki/SPSS

2 http://www.isbreeding.net/

3 http://wheat.pw.usda.gov/ggpages/wgc/98/Intro.htm

4 https://www.ncbi.nlm.nih.gov/

5 http://wheat.pw.usda.gov/GG3/index.shtml

6 https://urgi.versailles.inra.fr/download/iwgsc/

## Supplementary Material

Supplementary Table 1 Information of three variety panels assessed in this study (S1.1–1.3).

Supplementary Table 2 Ecological locations for measuring kernel length (KL), kernel width (KW), kernel thickness (KT), thousand-kernel weight (TKW), kernel length–width ratio (LWR), kernel size (KS), factor form density (FFD), spikelet number per spike (SNS), plant height (PH), productive tiller number (PTN), flag leaf length (FLL), flag leaf length width (FLW), spike length (SL), and spike density (SD).

Supplementary Table 3 Correlation coefficients for kernel traits in different environments.

Supplementary Table 4 Correlation coefficients among different kernel traits.

Supplementary Table 5 Correlation coefficients for kernel traits with spikelet number per spike (SNS), plant height (PH), productive tiller number (PTN), flag leaf length (FLL), flag leaf width (FLW), spike length (SL), and spike density (SD) in 2SY population.

Supplementary Table 6 All quantitative trait loci for kernel traits identified in the “20828” × “SY95-71” population evaluated in different environments.

Supplementary Table 7 Analyses of variance for kernel-related traits.

Supplementary Table 8 Quantitative trait loci (QTL) detected in the QTL × environment (QE) interaction module for kernel-related traits in 2SY population.

Supplementary Table 9 Predicted genes in the interval of the major QTL (S9.1–9.3).

Supplementary Table 10 Comparison of the major quantitative trait loci (QTL) for kernel-related traits identified in this study with previous studies.

Supplementary Table 11 The physical location information of the detected major QTL.
