# Supplementary material for: Genetic Mapping and Validation of Loci for Kernel-Related Traits in Wheat (Triticum aestivum L.)
Source: Front Plant Sci. 2021 Jun 7;12:667493. doi: 10.3389/fpls.2021.667493 (PMC8215603; doi:10.3389/fpls.2021.667493)
Supplement: Supplementary Table 1 — Information of three variety panels assessed in this study (S1.1–1.3). [file Table_1.DOCX]

**Table S1** Information of three variety panels assessed in this study (Table S1.1-S1.3).

**Table S1.1** Details of 272 landraces wheat in China

| **Count** | **Material name** | **Origin** | **Chinese wheat plant zones** |  | **Count** | **Material name** | **Origin** | **Chinese wheat plant zones** |
| --- | --- | --- | --- | --- | --- | --- | --- | --- |
| 1 | Chinese Spring | sichuan | SWAS |  | 137 | zaohuangmai | sichuan | SWAS |
| 2 | baixiaomai | beijing | NW |  | 138 | hongtiaomai | sichuan | SWAS |
| 3 | hongmangbai | tianjin | NW |  | 139 | yupi | sichuan | SWAS |
| 4 | baitumai | shangdong | YH |  | 140 | dahongmang | hebei | YH |
| 5 | hongduomai | shangdong | YH |  | 141 | hongxumai | sichuan | SWAS |
| 6 | yulincao | shangdong | YH |  | 142 | lushanmai | sichuan | SWAS |
| 7 | baihulutou | tianjin | NW |  | 143 | tuotuomai | sichuan | SWAS |
| 8 | baimangyouzi | henan | YH |  | 144 | baikebai | hebei | YH |
| 9 | baigedatou | henan | YH |  | 145 | yuweimai | sichuan | SWAS |
| 10 | dakoumai | henan | YH |  | 146 | hechuanmai | sichuan | SWAS |
| 11 | cantiaomai | henan | YH |  | 147 | huayangxiaomai | sichuan | SWAS |
| 12 | xiaozibai | henan | YH |  | 148 | huanghuaxiaomai | sichuan | SWAS |
| 13 | kumai | henan | YH |  | 149 | guangguangtou | sichuan | SWAS |
| 14 | xiaobaimang | hebei | YH |  | 150 | baikejiang | sichuan | SWAS |
| 15 | pushanbamai | henan | YH |  | 151 | baihulutou | hebei | YH |
| 16 | baitiaoyu | henan | YH |  | 152 | hongmangmaizi | sichuan | QT |
| 17 | wuhuatou | henan | YH |  | 153 | zaodongmai | sichuan | SWAS |
| 18 | youzimai | hebei | YH |  | 154 | yicuomao | sichuan | SWAS |
| 19 | tutoumai | henan | YH |  | 155 | baimaier | sichuan | SWAS |
| 20 | laochushanbao | henan | YH |  | 156 | baikexuxusanyuehuang | sichuan | SWAS |
| 21 | lingbao925 | henan | YH |  | 157 | yuqiumai | guizhou | SWAS |
| 22 | youmangcao | henan | YH |  | 158 | caoxiepian | guizhou | SWAS |
| 23 | dalihong | henan | YH |  | 159 | dabaimai | shanxi | NW |
| 24 | zihuatou | henan | YH |  | 160 | huimai | guizhou | SWAS |
| 25 | benmai | henan | YH |  | 161 | guangtoumai | guizhou | SWAS |
| 26 | qumangmai | henan | YH |  | 162 | hongmangmai | guizhou | SWAS |
| 27 | xiaobaimai | henan | YTS |  | 163 | liulengmai | guizhou | SWAS |
| 28 | liangganbai | henan | YTS |  | 164 | tiekemai | yunnan | SWAS |
| 29 | baipibai | hebei | NW |  | 165 | zimai | yunnan | SWAS |
| 30 | baiheshangtou | henan | YH |  | 166 | yangmaizi | yunnan | SWAS |
| 31 | yerenmao | henan | YH |  | 167 | erdaomei | inner mongolia | NS |
| 32 | xiaozihong | henan | YH |  | 168 | xiaobaipi | inner mongolia | NS |
| 33 | youmangcao | henan | YTS |  | 169 | xiaomangmai | shanxi | NW |
| 34 | huomai | henan | YTS |  | 170 | baiyuhua | jiangsu | YTS |
| 35 | baihuomai | henan | YH |  | 171 | nanjinghuang | jiangsu | YTS |
| 36 | changmangbaike | shaanxi | NW |  | 172 | zhenjiang3-96 | jiangsu | YTS |
| 37 | xiaomaimang | hebei | YH |  | 173 | heshangmai | fujian | SAS |
| 38 | shihuiyaohuomai | shaanxi | NW |  | 174 | guangtoumai | jiangxi | YTS |
| 39 | siqiangxiaomai | shaanxi | NW |  | 175 | baisuizi | shangdong | YH |
| 40 | nv'erhong | shaanxi | NW |  | 176 | yejihong | shangdong | YH |
| 41 | daqimai | shaanxi | YH |  | 177 | changshamai | hubei | YTS |
| 42 | gouweibabaimai | shaanxi | YH |  | 178 | baimai | hubei | YTS |
| 43 | baimai39F | hebei | NW |  | 179 | zhuganqing | shanxi | NW |
| 44 | yu'ermai | shaanxi | YH |  | 180 | zhuganqing | hubei | YTS |
| 45 | laobaitiaozi | shaanxi | NW |  | 181 | xiaoganmai | hubei | YTS |
| 46 | yangmai | shaanxi | NW |  | 182 | caoxieban | hubei | YTS |
| 47 | gongxianmai | hebei | YH |  | 183 | yangmai | hubei | YTS |
| 48 | xiaohongmang | heilongjiang | NES |  | 184 | baimanghong | shanxi | NW |
| 49 | xuemai | jilin | NES |  | 185 | tiezimai | hubei | YTS |
| 50 | xinjinyoumang | liaoning | NW |  | 186 | quanmangxiaomai | hubei | YTS |
| 51 | xiaozhihongmai | liaoning | NW |  | 187 | xishuibagutao | hubei | YTS |
| 52 | mangmai | inner mongolia | NS |  | 188 | zimai | hubei | YTS |
| 53 | baimangmai | beijing | NW |  | 189 | wugongmai | hubei | YTS |
| 54 | xiaobaisui | inner mongolia | NS |  | 190 | wugongxu | hubei | YTS |
| 55 | baichunmai | gansu | NWS |  | 191 | baikeheshangmai | hunan | YTS |
| 56 | laohongmai | gansu | NWS |  | 192 | baiyuxiaomai | sichuan | QT |
| 57 | baike | hebei | NW |  | 193 | gaoshanzaoshuxiaomai | sichuan | QT |
| 58 | xiaomai | gansu | NWS |  | 194 | zhaonixiaomai | sichuan | SWAS |
| 59 | dabaimai | hebei | YH |  | 195 | ranriwumangmai | sichuan | QT |
| 60 | baidatou | gansu | NWS |  | 196 | qianqianmai | guizhou | SWAS |
| 61 | shanxihong | gansu | NWS |  | 197 | guangtoubaikemai | yunnan | SWAS |
| 62 | baiqimai | gansu | NW |  | 198 | huakemai | yunnan | SWAS |
| 63 | xiaoqingmang | gansu | NW |  | 199 | xiaohongmai | shaanxi | NW |
| 64 | dabailing | hebei | YH |  | 200 | shanyangxue | shaanxi | NW |
| 65 | kuchebaidongmai | xinjiang | XJ |  | 201 | baixianmang | shanxi | NW |
| 66 | qingxinlanmai | xinjiang | XJ |  | 202 | bailanmai | shaanxi | YH |
| 67 | zijingbai | hebei | NW |  | 203 | baiwugongcao | shaanxi | YH |
| 68 | jianzituan | jiangsu | YTS |  | 204 | baijiantiao | shaanxi | NW |
| 69 | xiaobaipi | hebei | YH |  | 205 | youmangshangeda | shanxi | NW |
| 70 | huanghuazhu | jiangsu | YTS |  | 206 | cantiaomai | shaanxi | NW |
| 71 | yejihong | jiangsu | YTS |  | 207 | pushanba | shaanxi | YTS |
| 72 | liulengmai | hebei | YH |  | 208 | deguolan | shaanxi | YH |
| 73 | huoshaotian | jiangsu | YTS |  | 209 | hongmaomai | gansu | NWS |
| 74 | guangdezao | jiangsu | YTS |  | 210 | xiaohongmai(babao) | qinghai | QT |
| 75 | erguangtou | hebei | NW |  | 211 | xiaohongmai | qinghai | QT |
| 76 | nuopangtou | jiangsu | YTS |  | 212 | galaohan | qinghai | QT |
| 77 | daoshuibai | jiangsu | YTS |  | 213 | lanmai | qinghai | QT |
| 78 | baizhaoyu | jiangsu | YTS |  | 214 | baishanmai | shanxi | NW |
| 79 | qingmangzi | jiangsu | YTS |  | 215 | wangkamai | tibet | QT |
| 80 | huaqixiaomai | jiangsu | YTS |  | 216 | wumangbaimai | tibet | QT |
| 81 | manghuazi | jiangsu | YTS |  | 217 | zharenmabu | tibet | QT |
| 82 | baimangzi | jiangsu | YTS |  | 218 | zharenzhuoma | tibet | QT |
| 83 | dahongpi | jiangsu | YTS |  | 219 | zhaguogouqumai | tibet | QT |
| 84 | daqitou | hebei | NW |  | 220 | zhana | tibet | QT |
| 85 | zhuantoubaike | jiangsu | YH |  | 221 | louguding | shangdong | YH |
| 86 | hongmai | jiangsu | YH |  | 222 | maoyinmai | tibet | QT |
| 87 | xiaolihong | jiangsu | YH |  | 223 | changmangmaoyinmai | tibet | QT |
| 88 | baisuihong | jiangsu | YTS |  | 224 | changmangpeizhuo | tibet | QT |
| 89 | tutoubai | hebei | NW |  | 225 | renbuchun | tibet | QT |
| 90 | shunshuihong | jiangsu | YTS |  | 226 | rendachangguangmai | tibet | QT |
| 91 | xiaoyuhua | jiangsu | YTS |  | 227 | qimai | shangdong | YH |
| 92 | putaomai | shanghai | YTS |  | 228 | jiachazharenzhuoma | tibet | QT |
| 93 | baijiaomai | shanghai | YTS |  | 229 | jiachashumai | tibet | QT |
| 94 | huxumai | anhui | YTS |  | 230 | jidingwumangchun | tibet | QT |
| 95 | wugongmai | anhui | YTS |  | 231 | jinbaoyu | shangdong | YH |
| 96 | jiangxizao | anhui | YTS |  | 232 | quxiajizhuo | tibet | QT |
| 97 | wugongbian | anhui | YTS |  | 233 | suzhuozhuoma | tibet | QT |
| 98 | dabaimang | hebei | NW |  | 234 | banmangmai | shandong | YH |
| 99 | dujiaqiaoxiaomai | zhejiang | YTS |  | 235 | bomizamai-5 | tibet | QT |
| 100 | baixu | zhejiang | YTS |  | 236 | zedangzaxiaomai | tibet | QT |
| 101 | baikeguangtou | zhejiang | YTS |  | 237 | dingrichangmang | tibet | QT |
| 102 | chunqiumaizi | hebei | NW |  | 238 | wumangxiaomai | tibet | QT |
| 103 | hongkewugong | zhejiang | YTS |  | 239 | bo’erqing | shandong | YH |
| 104 | xiaohongmang | hebei | NW |  | 240 | xiaohongmang | jiangsu | YH |
| 105 | tiezhuantou | zhejiang | YTS |  | 241 | molengmai | yunnan | SWAS |
| 106 | xianjumai | zhejiang | YTS |  | 242 | shenggexiaomai | tibet | QT |
| 107 | qingtianmai | zhejiang | YTS |  | 243 | zhaxigangzhuo | tibet | QT |
| 108 | hongtoumai | zhejiang | YTS |  | 244 | jigupizhuo | tibet | QT |
| 109 | xiyangmai | zhejiang | YTS |  | 245 | dadangzhuo | tibet | QT |
| 110 | hongmangbai | hebei | YH |  | 246 | henanbai | beijing | NW |
| 111 | wugongmai | zhejiang | YTS |  | 247 | yigongzhuo | tibet | QT |
| 112 | tiegengqing | zhejiang | YTS |  | 248 | bomiduanqu | tibet | QT |
| 113 | zaoxiaomai | zhejiang | YTS |  | 249 | tajizhuo | tibet | QT |
| 114 | qiyanghongke | hunan | YTS |  | 250 | pumanghongmai | tibet | QT |
| 115 | baimangmai | hebei | YH |  | 251 | tarongzhuo | tibet | QT |
| 116 | heshangmai | hunan | YTS |  | 252 | hongmangxiaomai | shangdong | YH |
| 117 | baikeyoumang | fujian | SAS |  | 253 | fanshenzhuo | tibet | QT |
| 118 | youmai | guangdong | SAS |  | 254 | laobaimai | gansu | NWS |
| 119 | datianquxiaomai | guangdong | SAS |  | 255 | youmangyangmai | gansu | SWAS |
| 120 | qingyuanxiaomai | guangdong | SAS |  | 256 | hongtuzi | ningxia | NWS |
| 121 | huoshaomai | guangdong | SAS |  | 257 | xiaogongxian | shandong | YH |
| 122 | guchengxiaomai | hebei | YH |  | 258 | baishamai | shandong | YH |
| 123 | baikexiaomai | guangdong | SAS |  | 259 | huangshuibai | zhejiang | YTS |
| 124 | zaoxiaomai | guangdong | SAS |  | 260 | xiaobiansui | shandong | YH |
| 125 | puningxiaomai | guangdong | SAS |  | 261 | baitutou | shangdong | YH |
| 126 | zhangmuxiaomai | guangxi | SAS |  | 262 | banjiemangdabai | shandong | YH |
| 127 | guangtoumai | guangxi | SAS |  | 263 | mazhatoumai | shangdong | YH |
| 128 | sanyuehuang | sichuan | SWAS |  | 264 | hongmanghongmai | shangdong | YH |
| 129 | tumai | beijing | NW |  | 265 | baikumai | shangdong | YH |
| 130 | gaochan1hao | hebei | YH |  | 266 | daqingmang | tianjin | NW |
| 131 | wuyangmai | sichuan | SWAS |  | 267 | baikangyangmai | shangdong | YH |
| 132 | huangmaizi | sichuan | SWAS |  | 268 | hongheshang | shandong | YH |
| 133 | yuzuiweimai | sichuan | SWAS |  | 269 | yejiling | shangdong | YH |
| 134 | youtiaomai | sichuan | SWAS |  | 270 | shidaosankemang | shangdong | YH |
| 135 | dahonghua | sichuan | SWAS |  | 271 | xiaohongmangliangmai | shandong | YH |
| 136 | honghulubaimai | hebei | YH |  | 272 | piaoxiao | shangdong | YH |

Geographic distribution of the tested landrace accessions. NW- Northern winter wheat zone, YH- Yellow and Huai River valleys facultative wheat zone, YTS- Middle and low Yangtze valleys autumnsown spring wheat zone, SAS- Southern autumn-sown spring wheat zone, SWAS- Southwestern autumn-sown spring wheat zone, NES- Northeastern spring wheat zone, NS-Northern spring wheat zone, NWS- Northwestern spring wheat zone, QT- Qinghai–Tibetan plateau spring-winter wheat zone) and XJ (xinjiang winter–spring wheat zone).

**Table S1.2** Details of 300 Chinese common wheats

| **Count** | **Material name** | **Count** | **Material name** | **Count** | **Material name** | **Count** | **Material name** |
| --- | --- | --- | --- | --- | --- | --- | --- |
| 1 | Sheng 172 | 76 | Xingmai 6 | 151 | Bainong 160 | 226 | Shannong 12 |
| 2 | Jimai 403 | 77 | Shannongda 7-9-3 | 152 | BN12 | 227 | Shixin 616 |
| 3 | Jimai 585 | 78 | Jinan 17 | 153 | Heng 6632 | 228 | Jinan 13 |
| 4 | Gaoyou 9618 | 79 | W110095 | 154 | Huaimei 28 | 229 | Xinmai 19 |
| 5 | Bao 5168 | 80 | Shannongda 5-6-6 | 155 | Jimai 21 | 230 | Xiaoyan 22 |
| 6 | Shannong 01-35 | 81 | 4BL-2R | 156 | Yong 4896 | 231 | Xu 10054 |
| 7 | Heng 05-6607 | 82 | Shannong 33 | 157 | Demai 1201 | 232 | Baofeng 2018 |
| 8 | Luomai 9 | 83 | Yunhan 2130 | 158 | Jinmai 47 | 233 | Xuzhou 24 |
| 9 | W11031 | 84 | Baomai 3 | 159 | Luohan 7 | 234 | Jund2016 |
| 10 | Bao 5067 | 85 | Lesson 15 | 160 | Keyi 10-6140 | 235 | Jinmai 30 |
| 11 | Daman 77 | 86 | Hemai 17 | 161 | Shiluan 02 | 236 | Jinmai 33 |
| 12 | Xumai 29 | 87 | Yanmai 98 | 162 | Shannongda 6-7-8 | 237 | Yannong 25 |
| 13 | LS6109 | 88 | Shannong 055849 | 163 | Nongda 179 | 238 | Xinong 889 |
| 14 | Hengguan 35 | 89 | Shannong 17 | 164 | Henong 7069 | 239 | Zhongmai 175 |
| 15 | Xu 11108 | 90 | Huaimei 29 | 165 | Chang 6878 | 240 | Baomai 12-6 |
| 16 | Henong 6119 | 91 | Shannongda 6-8-4 | 166 | Bodan 30 | 241 | Heng 95 Guan 26 |
| 17 | Yangao 1 | 92 | Aikang 58 | 167 | Jimai 24 | 242 | Tanmai 98 |
| 18 | Changhan 58 | 93 | Hanmai 13 | 168 | Ke 16 | 243 | Tainong 9236 |
| 19 | RS804 | 94 | Fengshou 60 | 169 | Yunong 201 | 244 | Shannong 55843 |
| 20 | Xu 9169 | 95 | Tainong 2987 | 170 | Yangao 21 | 245 | Nongda 189 |
| 21 | Xinmai 2119 | 96 | 10H4-14 | 171 | Ruiquanmai 168 | 246 | Shiluan 02-1 |
| 22 | Shannong 65 | 97 | Huaimei 17 | 172 | Bao 5108 | 247 | D45 |
| 23 | Baomai 2 | 98 | Sheng 126 | 173 | Tianhe 3 | 248 | Guomai 301 |
| 24 | Shannong 19 | 99 | Lifu 05 | 174 | Huaimei 30 | 249 | Luomai 24 |
| 25 | Zhengmai 7698 | 100 | D209 | 175 | Shixin 733 | 250 | Jimai 325 |
| 26 | D08-6 | 101 | W120134 | 176 | Jingmai 989 | 251 | Linzi 217 |
| 27 | Nongda 3432 | 102 | Fengdecun 1 | 177 | Sheng 232 | 252 | Zhongmai 12 |
| 28 | Shannongda 6-8-2 | 103 | Yumai 52 | 178 | Jinan 8 | 253 | Lumai 22 |
| 29 | Lankaoaozao 8 | 104 | Lunxuan 061 | 179 | Jinghe 91-P39 | 254 | Jinmai 31 |
| 30 | Handan 05-093 | 105 | Yannong 19 | 180 | Ke 0801 | 255 | Shaan 253 |
| 31 | Henong 05 (97) | 106 | Sheng 217 | 181 | Han 06-5170 | 256 | Yunhan 20410 |
| 32 | Zhongxin 5199 | 107 | Shimai 22 | 182 | Shannong 20-1 | 257 | Zhongxin 78 |
| 33 | 10X21 | 108 | Yunong 9901 | 183 | Lumai 1 | 258 | Wan 50 |
| 34 | Xinmai 21 | 109 | Xiaoyan 597 | 184 | Henong 4198 | 259 | Zhoumai 16 |
| 35 | Zhongyuanzhixing | 110 | Hengfu 9103 | 185 | Zhongmai 349 | 260 | TAM107 |
| 36 | Ningmaizi 28 | 111 | Henong 822 | 186 | Shengtian 69 | 261 | Jinmai 54 |
| 37 | Hengshui 4 | 112 | H6756 | 187 | Henong 6331 | 262 | JInboshi 731 |
| 38 | Sankecun | 113 | Hanzao 1 | 188 | Lumai 23 | 263 | Shi03-5285 |
| 39 | Xuke 718 | 114 | Sheng 119 | 189 | Chang 4640 | 264 | Gaoyou 9908 |
| 40 | Shannong 11 | 115 | Hansheng 923 | 190 | Wen 0418 | 265 | Jinmai 70 |
| 41 | Huaimei 22 | 116 | Jingdong 17 | 191 | Lumai 16 | 266 | Huaimei 33 |
| 42 | Lv Han 1608 | 117 | Zhengmai 101 | 192 | Wunong 6 | 267 | Luoma 05095 |
| 43 | Fumai 5 | 118 | Jimai 30 | 193 | Xumai 30 | 268 | Le 639 |
| 44 | Bainong 207 | 119 | Changwu 134 | 194 | Taishan 5 | 269 | Yan 2070 |
| 45 | Yunhan 618 | 120 | Luomai 21 | 195 | Baomaipin 8 | 270 | Xinmai 3 |
| 46 | Linyou 145 | 121 | Nongda 3492 | 196 | Jimai 2 | 271 | Bao 5113 |
| 47 | Zhongmai 9 | 122 | Baofeng 0601 | 197 | Henong 583 | 272 | Shi05-7388 |
| 48 | Henong 05 (9) | 123 | Shiluan 08-2 | 198 | Jimai 4 | 273 | Chang 4738 |
| 49 | Hannong 1412 | 124 | Sanshumai | 199 | Heng 71-3 | 274 | Cangmai 6005 |
| 50 | Yikemai 5 | 125 | Tainong 9862 | 200 | Keyi 11-6072 | 275 | Huapei 1 |
| 51 | Shannong 981 | 126 | Zhongmai 875 | 201 | RS53 | 276 | Afu |
| 52 | Taishan 4606 | 127 | Zhongjian 49 | 202 | Shi 4185 | 277 | Shannongjian 14-20 |
| 53 | Xinong 529 | 128 | Xin 9535 | 203 | Henghe 1 | 278 | Zhou Mai 28 |
| 54 | Xuzhou 25 | 129 | Bao 5036 | 204 | Jinuo 200 | 279 | Heng 05-4444 |
| 55 | Kaimai 21 | 130 | Linfeng 3 | 205 | Henong 06 | 280 | Keyu 16 |
| 56 | Baofeng 1082 | 131 | Heng 4399 | 206 | Lian 0809 | 281 | Jimai 19 |
| 57 | Shannongjian 25-229 | 132 | Zhongyu 01095 | 207 | Luo 23 | 282 | Chang 6135 |
| 58 | Liangxing 99 | 133 | Shannong 23 | 208 | Jinhe 8431 | 283 | Shunmai 1718 |
| 59 | Nongda 5181 | 134 | Shi 06-6136 | 209 | Sheng 219 | 284 | Tianmin 198 |
| 60 | Henong 7106 | 135 | Ligao 6 | 210 | Shannong 21 | 285 | Yujiao 5 |
| 61 | Yuanda 1 | 136 | Xinmai 23 | 211 | Ji 729 | 286 | Lian 0756 |
| 62 | Han 00-7050 | 137 | Shannongda 5-6-2 | 212 | Jinmai 63 | 287 | Xinmai 18 |
| 63 | Shi00-7221 | 138 | Zhongyu 01089 | 213 | Ping'an 9 | 288 | Zhoumai 19 |
| 64 | Ji 7369 | 139 | Kenong 3106 | 214 | Yujiao 6 | 289 | Zhengmai103 |
| 65 | Shannong 22 | 140 | Shannongjian 6E-1-17 | 215 | Wanmai 53 | 290 | Xinong 1376 |
| 66 | Bonong 6 | 141 | 09CA034 | 216 | Lunxuan 24 | 291 | Yannong 999 |
| 67 | Ruihua 1101 | 142 | D180 | 217 | Weilai 0818 | 292 | Ping'an 7 |
| 68 | Zhongmai 1 | 143 | Lumai 12 | 218 | Henong 825 | 293 | Henong 06 (159) |
| 69 | Gaoyou 2018 | 144 | Ji 5265 | 219 | Shannongjian 38-498 | 294 | Hanmai 9 |
| 70 | Tainong 18 | 145 | Taishan 21 | 220 | Huaimei 18 | 295 | Heng 7228 |
| 71 | Lumai 15 | 146 | Huaihe 0615 | 221 | 11CA26 | 296 | Jinmai 24 |
| 72 | Lunzao 3 | 147 | Sheng 125 | 222 | Xuke 316 | 297 | Shannongjian 41-560 |
| 73 | Shannong 20 | 148 | Sheng 183 | 223 | Baomai 8 | 298 | Jimai 22 |
| 74 | Shannong 10-2 | 149 | Handan 6172 | 224 | Shaanmai 159 | 299 | Keyu 11 |
| 75 | Shi05-6678 | 150 | Xinong 979 | 225 | Mingtian 0402 | 300 | Nongda 399 |

**Table S1.3** Details of 165 Sichuan wheat cultivars

| **Count** | **Material name** | **Origin** | **Count** | **Material name** | **Origin** |
| --- | --- | --- | --- | --- | --- |
| 1 | Chuanmai 28 | Sichuan Academy of Agricultural Sciences | 84 | Changmai 28 | Xichang Academy of Agricultural Sciences |
| 2 | Chuanmai 29 | Sichuan Academy of Agricultural Sciences | 85 | Chuanmai 50 | Sichuan Academy of Agricultural Sciences |
| 3 | Mianyang 27 | Mianyang Academy of Agricultural Sciences | 86 | Chuanmai 51 | Sichuan Academy of Agricultural Sciences |
| 4 | Mianyang 28 | Mianyang Academy of Agricultural Sciences | 87 | Chuanmai 52 | Sichuan Academy of Agricultural Sciences |
| 5 | Chuanmai 30 | Sichuan Academy of Agricultural Sciences | 88 | Chuanyu 23 | Chengdu institute of biology, Chinese Academy of Sciences |
| 6 | Chuanyu 14 | Chengdu institute of biology, Chinese Academy of Sciences | 89 | Mianmai 185 | Mianyang Academy of Agricultural Sciences |
| 7 | Mianyang 26 | Mianyang Academy of Agricultural Sciences | 90 | Mianmai 46 | Mianyang Academy of Agricultural Sciences |
| 8 | Panmai 6 | Panzhihua Academy of Agricultural Sciences | 91 | Rongmai 757 | Chengdu Xingfeng Agricultural Technology Co., Ltd. |
| 9 | Mianyang 29 | Mianyang Academy of Agricultural Sciences | 92 | Shumai 482 | Sichuan Agricultural University |
| 10 | Panmai 7 | Panzhihua Academy of Agricultural Sciences | 93 | Xikemai 5 | SouthWest University of Science and Technology |
| 11 | Chuanmai 107 | Sichuan Academy of Agricultural Sciences | 94 | Xikemai 6 | SouthWest University of Science and Technology |
| 12 | Chuannong 7 | Sichuan Agricultural University | 95 | Bomai 1 | Zizhong Ruibo Crop Seed Research Institute |
| 13 | Changmai 32 | Xichang Academy of Agricultural Sciences | 96 | Chuanmai 53 | Sichuan Academy of Agricultural Sciences |
| 14 | Chuanmai 32 | Sichuan Academy of Agricultural Sciences | 97 | Chuanmai 54 | Sichuan Academy of Agricultural Sciences |
| 15 | Chuannong 11 | Sichuan Agricultural University | 98 | Chuanmai 55 | Sichuan Academy of Agricultural Sciences |
| 16 | Miannong 7 | SouthWest University of Science and Technology | 99 | Chuanmai 56 | Sichuan Academy of Agricultural Sciences |
| 17 | Chuanfu 5 | Sichuan Academy of Agricultural Sciences | 100 | Chuannong 26 | Chengdu Zhenglong Plant Breeding Co., Ltd.& Sichuan Agricultural University |
| 18 | Chuanmai 33 | Sichuan Academy of Agricultural Sciences | 101 | Chuannong 27 | Sichuan Agricultural University |
| 19 | Chuanmai 35 | Sichuan Academy of Agricultural Sciences | 102 | Chuanyu 24 | Chengdu institute of biology, Chinese Academy of Sciences |
| 20 | Chuanmai 36 | Sichuan Academy of Agricultural Sciences | 103 | Jinkemai 33 | Puchuanyong and Puchunlei |
| 21 | Chuannong 12 | Sichuan Agricultural University | 104 | Kechengmai 2 | Chengdu institute of biology, Chinese Academy of Sciences |
| 22 | Chuannong 16 | Sichuan Agricultural University | 105 | Mianmai 43 | Mianyang Academy of Agricultural Sciences |
| 23 | Chuannong 17 | Sichuan Agricultural University | 106 | Mianmai 48 | Mianyang Academy of Agricultural Sciences |
| 24 | Chuanyu 16 | Chengdu institute of biology, Chinese Academy of Sciences | 107 | Xianmai 99 | Sichuan Queliang Seeds Industry Co., Ltd. |
| 25 | Mianyang 31 | Mianyang Academy of Agricultural Sciences | 108 | Yumai 1 | Sichuan Shenglong Tianxiang Technology Co., Ltd. |
| 26 | Chuanmai 37 | Sichuan Academy of Agricultural Sciences | 109 | Chuanchongzu 125 | Sichuan Academy of Agricultural Sciences |
| 27 | Chuanmai 38 | Sichuan Academy of Agricultural Sciences | 110 | Chuanmai 58 | Sichuan Academy of Agricultural Sciences |
| 28 | Chuanmai 39 | Sichuan Academy of Agricultural Sciences | 111 | Neimai 3416 | Neijiang Academy of Agricultural Sciences |
| 29 | Chuanmai 41 | Sichuan Academy of Agricultural Sciences | 112 | Chuanmai 60 | Sichuan Academy of Agricultural Sciences |
| 30 | Chuanmai 42 | Sichuan Academy of Agricultural Sciences | 113 | Guohaomai 15 | Sichuan Guohao Seeds Industry Co., Ltd. |
| 31 | Chuannong 10 | Sichuan Agricultural University | 114 | Mianmai 228 | Mianyang Academy of Agricultural Sciences |
| 32 | Chuannong 18 | Sichuan Agricultural University | 115 | Chuanmai 104 | Sichuan Academy of Agricultural Sciences |
| 33 | Chuannong 19 | Sichuan Agricultural University | 116 | Chuanmai 61 | Sichuan Academy of Agricultural Sciences |
| 34 | Chuanyu 18 | Chengdu institute of biology, Chinese Academy of Sciences | 117 | Chuanmai 62 | Sichuan Academy of Agricultural Sciences |
| 35 | Chuanyu 19 | Chengdu institute of biology, Chinese Academy of Sciences | 118 | Mianmai 51 | Mianyang Academy of Agricultural Sciences |
| 36 | Mianyang 30 | Mianyang Academy of Agricultural Sciences | 119 | Nanmai 302 | Nanchong Academy of Agricultural Sciences |
| 37 | Mianyang 33 | Mianyang Academy of Agricultural Sciences | 120 | Xikemai 7 | SouthWest University of Science and Technology |
| 38 | Mianyang 35 | Mianyang Academy of Agricultural Sciences | 121 | Y11-1741 | Sichuan Agricultural University |
| 39 | Neimai 8 | Neijiang Academy of Agricultural Sciences | 122 | Chuanmai 63 | Sichuan Academy of Agricultural Sciences |
| 40 | Rongmai 2 | Chengdu Academy of Agricultural and Forestry Sciences | 123 | Chuanmai 64 | Sichuan Academy of Agricultural Sciences |
| 41 | Xichang 18 | Xichang Academy of Agricultural Sciences | 124 | Chuanmai 65 | Sichuan Academy of Agricultural Sciences |
| 42 | Xifu 12 | Xichang Academy of Agricultural Sciences | 125 | Mianmai 1618 | Mianyang Academy of Agricultural Sciences |
| 43 | Xikemai 1 | SouthWest University of Science and Technology | 126 | Nanmai 618 | Nanchong Academy of Agricultural Sciences |
| 44 | Chuanmai 43 | Sichuan Academy of Agricultural Sciences | 127 | Shumai 51 | Sichuan Agricultural University |
| 45 | Chuanmai 44 | Sichuan Academy of Agricultural Sciences | 128 | Shumai 969 | Sichuan Agricultural University |
| 46 | Chuannong 21 | Sichuan Agricultural University | 129 | Teyanmainan 88 | Nanchong Academy of Agricultural Sciences |
| 47 | Liangmai 2 | Sichuan Agricultural University | 130 | Xikemai 8 | SouthWest University of Science and Technology |
| 48 | Mianmai 37 | Mianyang Academy of Agricultural Sciences | 131 | Chuanmai 1131 | Sichuan Academy of Agricultural Sciences |
| 49 | Mianmai 38 | Mianyang Academy of Agricultural Sciences | 132 | Chuanmai 1145 | Sichuan Academy of Agricultural Sciences |
| 50 | Neimai 9 | Neijiang Academy of Agricultural Sciences | 133 | Chuanmai 1247 | Sichuan Academy of Agricultural Sciences |
| 51 | Xichang 19 | Xichang Academy of Agricultural Sciences | 134 | Chuanmai 66 | Sichuan Academy of Agricultural Sciences |
| 52 | Xifu 13 | Xichang Academy of Agricultural Sciences | 135 | Chuanmai 67 | Sichuan Academy of Agricultural Sciences |
| 53 | Xingmai 2 | Neijiang Academy of Agricultural Sciences | 136 | Chuanmai 80 | Sichuan Academy of Agricultural Sciences |
| 54 | Chuanmai 45 | Sichuan Academy of Agricultural Sciences | 137 | Chuanmai 90 | Sichuan Academy of Agricultural Sciences |
| 55 | Chuanmai 46 | Sichuan Academy of Agricultural Sciences | 138 | Chuanmai 91 | Sichuan Academy of Agricultural Sciences |
| 56 | Chuanmai 47 | Sichuan Academy of Agricultural Sciences | 139 | Rongchuannanmai 1 | Nanchong Academy of Agricultural Sciences & Sichuan Rongchun Seed Industry Co., Ltd. |
| 57 | Chuannong 22 | Sichuan Agricultural University | 140 | Xikemai 9 | SouthWest University of Science and Technology |
| 58 | Chuannong 23 | Sichuan Agricultural University | 141 | Yimai 9 | Yibin Academy of Agricultural Sciences |
| 59 | Kechengmai 1 | Chengdu institute of biology, Chinese Academy of Sciences | 142 | Zhongkemai 138 | Chengdu institute of biology, Chinese Academy of Sciences |
| 60 | Liangmai 3 | Sichuan Agricultural University | 143 | Zhongkemai 47 | Chengdu institute of biology, Chinese Academy of Sciences |
| 61 | Mianmai 39 | Mianyang Academy of Agricultural Sciences | 144 | Chuanfu 7 | Sichuan Academy of Agricultural Sciences |
| 62 | Mianmai 40 | Mianyang Academy of Agricultural Sciences | 145 | Chuanfu 8 | Sichuan Academy of Agricultural Sciences |
| 63 | Xikemai 2 | SouthWest University of Science and Technology | 146 | Chuanmai 68 | Sichuan Academy of Agricultural Sciences |
| 64 | Changmai 26 | Xichang Academy of Agricultural Sciences | 147 | Chuanmai 81 | Sichuan Academy of Agricultural Sciences |
| 65 | Chuanmai 48 | Sichuan Academy of Agricultural Sciences | 148 | Chuanmai 92 | Sichuan Academy of Agricultural Sciences |
| 66 | Chuanmai 49 | Sichuan Academy of Agricultural Sciences | 149 | Chuannong 29 | Sichuan Agricultural University |
| 67 | Chuanyu 20 | Chengdu institute of biology, Chinese Academy of Sciences | 150 | Chuanyu 25 | Chengdu institute of biology, Chinese Academy of Sciences |
| 68 | Mianmai 41 | Mianyang Academy of Agricultural Sciences | 151 | Kechengmai 4 | Chengdu institute of biology, Chinese Academy of Sciences |
| 69 | Mianmai 42 | Mianyang Academy of Agricultural Sciences | 152 | Mianmai 112 | Mianyang Academy of Agricultural Sciences |
| 70 | Xifu 14 | Xichang Academy of Agricultural Sciences | 153 | Nanmai 991 | Nanchong Academy of Agricultural Sciences |
| 71 | Zimai 1 | Sichuan Wanfa Seeds Industry Co., Ltd. | 154 | Neimai 366 | Neijiang Academy of Agricultural Sciences |
| 72 | Chengdianmai 1 | University of Electronic Science and Techonology of China | 155 | Xikemai 10 | SouthWest University of Science and Technology |
| 73 | Chuannong 24 | Sichuan Agricultural University | 156 | Changmai 33 | Xichang Academy of Agricultural Sciences |
| 74 | Chuannong 25 | Chengdu Zhenglong Plant Breeding Co., Ltd.& Sichuan Agricultural University | 157 | Chuanmai 601 | Sichuan Academy of Agricultural Sciences |
| 75 | Chuanyu 21 | Chengdu institute of biology, Chinese Academy of Sciences | 158 | Chuannong 30 | Sichuan Agricultural University & Sichuan Yicheng Modern Agricultural Technology Co., Ltd. |
| 76 | Liangmai 4 | Sichuan Agricultural University | 159 | Chuanyu 26 | Chengdu institute of biology, Chinese Academy of Sciences |
| 77 | Mianmai 1403 | Mianyang Academy of Agricultural Sciences | 160 | Guohaomai 3 | Sichuan Guohao Seeds Industry Co., Ltd. |
| 78 | Mianmai 45 | Mianyang Academy of Agricultural Sciences | 161 | Kechengmai 5 | Chengdu institute of biology, Chinese Academy of Sciences |
| 79 | Neimai 11 | Neijiang Academy of Agricultural Sciences | 162 | Mianmai 285 | Mianyang Academy of Agricultural Sciences |
| 80 | Rongmai 4 | Chengdu Academy of Agricultural and Forestry Sciences | 163 | Shumai 126 | Sichuan Agricultural University |
| 81 | Shumai 375 | Sichuan Agricultural University | 164 | Shumai 921 | Sichuan Agricultural University |
| 82 | Xikemai 3 | SouthWest University of Science and Technology | 165 | Xikemai 18 | SouthWest University of Science and Technology |
| 83 | Xikemai 4 | SouthWest University of Science and Technology |  |  |  |
