# Supplementary material for: Genetic Mapping and Validation of Loci for Kernel-Related Traits in Wheat (Triticum aestivum L.)
Source: Front Plant Sci. 2021 Jun 7;12:667493. doi: 10.3389/fpls.2021.667493 (PMC8215603; doi:10.3389/fpls.2021.667493)
Supplement: Supplementary Table 2 — Ecological locations for measuring kernel length (KL), kernel width (KW), kernel thickness (KT), thousand-kernel weight (TKW), kernel length–width ratio (LWR), kernel size (KS), factor form density (FFD), spikelet number per spike (SNS), plant height (PH), productive tiller number (PTN), flag leaf length (FLL), flag leaf length width (FLW), spike length (SL), and spike density (SD). [file Table_2.DOCX]

**Table S2** Ecological location for measuring kernel length (KL), kernel width (KW), kernel thickness (KT), thousand-kernel weight (TKW), kernel length-width ratio (LWR), kernel size (KS), factor form density (FFD), spikelet number per spike (SNS), plant height (PH), productive tiller number (PTN), Anthesis date (AD), flag leaf length (FLL), flag leaf length width (FLW), spike length (SL), and spike density (SD).

| **Population** | **Traits** | **Environments** | **References** |
| --- | --- | --- | --- |
| 2SY | KL | 2017CZ 2017YA 2018CZ 2018YA 2019CZ 2019WJ | this study |
|  | KW | 2017CZ 2017YA 2018CZ 2018YA 2019CZ 2019WJ | this study |
|  | KT | 2017CZ 2017YA 2018CZ 2018YA 2019CZ 2019WJ | this study |
|  | TKW | 2017CZ 2017YA 2019CZ 2019WJ | [1] |
|  | TKW | 2018CZ 2018YA | this study |
|  | LWR | 2017CZ 2017YA 2018CZ 2018YA 2019CZ 2019WJ | this study |
|  | KS | 2017CZ 2017YA 2018CZ 2018YA 2019CZ 2019WJ | this study |
|  | FFD | 2017CZ 2017YA 2018CZ 2018YA 2019CZ 2019WJ | this study |
|  | SNS | 2017CZ 2017WJ 2017YA 2018WJ 2018CZ 2018YA | [1] |
|  | PH | 2017CZ 2017WJ 2017YA 2018WJ 2018CZ 2018YA | [1] |
|  | PTN | 2017CZ 2017WJ 2017YA 2019CZ 2019WJ 2019YA 2019KB | [1] |
|  | AD | 2017WJ 2017CZ 2018WJ 2018CZ | [1] |
|  | FLL | 2017CZ 2017WJ 2017YA 2018WJ 2018CZ 2018YA 2019WJ 2019CZ 2019YA | [2] |
|  | FLW | 2017CZ 2017WJ 2017YA 2018WJ 2018CZ 2018YA 2019WJ 2019CZ 2019YA | [2] |
|  | SL | 2017CZ 2017WJ 2017YA 2018CZ 2018WJ 2018YA 2018KB | [3] |
|  | SD | 2017CZ 2017WJ 2017YA 2018CZ 2018WJ 2018YA 2018KB | this study |
| SSY | KL | 2020CZ | this study |
| MTL4SY | KL | 2020CZ | this study |

**Note:**

Population: 2SY: 20828 / SY95-71; SSY: S849-8 / SY95-71; MTL4SY: MTL4 / SY95-71

[1] Liu J, Tang H, Qu X, Liu H, LI C, Tu Y, Li S, Habib A, Mu Y, Dai S, Deng M, Jiang Q, Liu Y, Chen G, Wang J, Chen G, Li W, Jiang Y, Wei Y, Lan X, Zheng Y, Ma J (2020) A novel, major, and validated QTL for the effective tiller number located on chromosome arm 1BL in bread wheat. Plant Mol Biol 104:173-185.

[2] Ma J, Tu Y, Zhu J, Luo W, Liu H, Li C, Li S, Liu J, Ding P, Habib A (2020) Flag leaf size and posture of bread wheat: genetic dissection, QTL validation and their relationships with yield-related traits. Theor Appl Genet 133:297-315.

[3] Li C, Tang H, Luo W, Zhang X, Mu Y, Deng M, Liu Y, Jiang Q, Chen G, Wang J, Qi P, Pu Z, Jiang Y, Wei Y, Zheng Y, Lan X, Ma J (2020) A novel, validated, and plant height-independent QTL for spike extension length is associated with yield-related traits in wheat. Theor Appl Genet 133:3381–3393.
