# Supplementary material for: Genetic Mapping and Validation of Loci for Kernel-Related Traits in Wheat (Triticum aestivum L.)
Source: Front Plant Sci. 2021 Jun 7;12:667493. doi: 10.3389/fpls.2021.667493 (PMC8215603; doi:10.3389/fpls.2021.667493)
Supplement: Supplementary Table 3 — Correlation coefficients for kernel traits in different environments. [file Table_3.DOCX]

**Table S3** Correlation coefficients for kernel traits in different environments

kernel length

|  | 2017CZ | 2017YA | 2018CZ | 2018YA | 2019CZ | 2019WJ | BLUP |
| --- | --- | --- | --- | --- | --- | --- | --- |
| 2017CZ | 1 |  |  |  |  |  |  |
| 2017YA | 0.61** | 1 |  |  |  |  |  |
| 2018CZ | 0.67** | 0.45** | 1 |  |  |  |  |
| 2018YA | 0.63** | 0.38** | 0.60** | 1 |  |  |  |
| 2019CZ | 0.61** | 0.37** | 0.65** | 0.54** | 1 |  |  |
| 2019WJ | 0.66** | 0.39** | 0.74** | 0.63** | 0.69** | 1 |  |
| BLUP | 0.85** | 0.62** | 0.86** | 0.82** | 0.80** | 0.86** | 1 |

kernel width

|  | 2017CZ | 2017YA | 2018CZ | 2018YA | 2019CZ | 2019WJ | BLUP |
| --- | --- | --- | --- | --- | --- | --- | --- |
| 2017CZ | 1 |  |  |  |  |  |  |
| 2017YA | 0.31** | 1 |  |  |  |  |  |
| 2018CZ | 0.26** | 0.31** | 1 |  |  |  |  |
| 2018YA | 0.17 | 0.25* | 0.45** | 1 |  |  |  |
| 2019CZ | 0.46** | 0.32** | 0.43** | 0.41** | 1 |  |  |
| 2019WJ | 0.24* | 0.30** | 0.14 | 0.12 | 0.35** | 1 |  |
| BLUP | 0.61** | 0.60** | 0.62** | 0.69** | 0.77** | 0.56** | 1 |

kernel thickness

|  | 2017CZ | 2017YA | 2018CZ | 2018YA | 2019CZ | 2019WJ | BLUP |
| --- | --- | --- | --- | --- | --- | --- | --- |
| 2017CZ | 1 |  |  |  |  |  |  |
| 2017YA | 0.11 | 1 |  |  |  |  |  |
| 2018CZ | 0.43** | 0.20* | 1 |  |  |  |  |
| 2018YA | 0.32** | 0.19* | 0.52** | 1 |  |  |  |
| 2019CZ | 0.41** | 0.08 | 0.35** | 0.37** | 1 |  |  |
| 2019WJ | 0.35** | 0.08 | 0.39** | 0.30** | 0.33** | 1 |  |
| BLUP | 0.64** | 0.39** | 0.77** | 0.77** | 0.60** | 0.64** | 1 |

thousand kernel weight

|  | 2017CZ | 2017YA | 2018CZ | 2018YA | 2019CZ | 2019WJ | BLUP |
| --- | --- | --- | --- | --- | --- | --- | --- |
| 2017CZ | 1 |  |  |  |  |  |  |
| 2017YA | 0.41** | 1 |  |  |  |  |  |
| 2018CZ | 0.44** | 0.41** | 1 |  |  |  |  |
| 2018YA | 0.33** | 0.31** | 0.39** | 1 |  |  |  |
| 2019CZ | 0.35** | 0.25** | 0.48** | 0.25* | 1 |  |  |
| 2019WJ | 0.38** | 0.43** | 0.51** | 0.25* | 0.42** | 1 |  |
| BLUP | 0.69** | 0.61** | 0.77** | 0.68** | 0.67** | 0.72** | 1 |

kernel length/kernel width

|  | 2017CZ | 2017YA | 2018CZ | 2018YA | 2019CZ | 2019WJ | BLUP |
| --- | --- | --- | --- | --- | --- | --- | --- |
| 2017CZ | 1 |  |  |  |  |  |  |
| 2017YA | 0.23* | 1 |  |  |  |  |  |
| 2018CZ | 0.03 | 0.55** | 1 |  |  |  |  |
| 2018YA | 0.42** | 0.10 | 0.03 | 1 |  |  |  |
| 2019CZ | 0.28** | 0.05 | 0.36** | 0.31** | 1 |  |  |
| 2019WJ | 0.03 | 0.45** | 0.67** | 0.01 | 0.19* | 1 |  |
| BLUP | 0.14 | 0.25** | 0.50** | 0.34** | 0.28* | 0.33** | 1 |

kernel size

|  | 2017CZ | 2017YA | 2018CZ | 2018YA | 2019CZ | 2019WJ | BLUP |
| --- | --- | --- | --- | --- | --- | --- | --- |
| 2017CZ | 1 |  |  |  |  |  |  |
| 2017YA | 0.08 | 1 |  |  |  |  |  |
| 2018CZ | -0.01 | 0.32* | 1 |  |  |  |  |
| 2018YA | 0.19* | 0.07 | 0.05 | 1 |  |  |  |
| 2019CZ | 0.17 | 0.12 | 0.32** | 0.28** | 1 |  |  |
| 2019WJ | 0.02 | 0.24** | 0.31** | 0.04 | 0.26** | 1 |  |
| BLUP | 0.23 | 0.22* | 0.41** | 0.28** | 0.50** | 0.15 | 1 |

factor form density

|  | 2017CZ | 2017YA | 2018CZ | 2018YA | 2019CZ | 2019WJ | BLUP |
| --- | --- | --- | --- | --- | --- | --- | --- |
| 2017CZ | 1 |  |  |  |  |  |  |
| 2017YA | 0.31** | 1 |  |  |  |  |  |
| 2018CZ | 0.22* | 0.35** | 1 |  |  |  |  |
| 2018YA | 0.29** | 0.01 | 0.25* | 1 |  |  |  |
| 2019CZ | 0.14 | 0.12 | 0.19* | 0.13 | 1 |  |  |
| 2019WJ | 0.26* | 0.39** | 0.49** | 0.21 | 0.18 | 1 |  |
| BLUP | 0.56** | 0.50** | 0.65** | 0.59** | 0.64** | 0.66** | 1 |

* Correlation is significant at the 0.05 level, ** Correlation is significant at the 0.01 level.
