# Supplementary material for: Genetic Mapping and Validation of Loci for Kernel-Related Traits in Wheat (Triticum aestivum L.)
Source: Front Plant Sci. 2021 Jun 7;12:667493. doi: 10.3389/fpls.2021.667493 (PMC8215603; doi:10.3389/fpls.2021.667493)
Supplement: Supplementary Table 4 — Correlation coefficients among different kernel traits. [file Table_4.DOCX]

**Table S4** Correlation coefficients among different kernel traits

|  | KL | KW | KT | TKW | LWR | KS | FFD |
| --- | --- | --- | --- | --- | --- | --- | --- |
| KL | 1 |  |  |  |  |  |  |
| KW | 0.35** | 1 |  |  |  |  |  |
| KT | 0.41** | 0.57** | 1 |  |  |  |  |
| TKW | 0.58** | 0.73** | 0.76** | 1 |  |  |  |
| LWR | 0.61** | -0.38** | -0.05 | -0.06 | 1 |  |  |
| KS | 0.78** | 0.79** | 0.77** | 0.84** | 0.16 | 1 |  |
| FFD | 0.12 | 0.45** | 0.51** | 0.71** | -0.22** | 0.42** | 1 |

** Correlation is significant at the 0.01 level.
