# Supplementary material for: Genetic Mapping and Validation of Loci for Kernel-Related Traits in Wheat (Triticum aestivum L.)
Source: Front Plant Sci. 2021 Jun 7;12:667493. doi: 10.3389/fpls.2021.667493 (PMC8215603; doi:10.3389/fpls.2021.667493)
Supplement: Supplementary Table 5 — Correlation coefficients for kernel traits with spikelet number per spike (SNS), plant height (PH), productive tiller number (PTN), flag leaf length (FLL), flag leaf width (FLW), spike length (SL), and spike density (SD) in 2SY population. [file Table_5.DOCX]

**Table S5** Correlation coefficients for kernel traits with spikelet number per spike (SNS), plant height (PH), productive tiller number (PTN), flag leaf length (FLL), flag leaf width (FLW), spike length (SL), spike density (SD) and heading date (AD) in 2SY population

|  | SNS | PH | PTN | FLL | FLW | SL | SD | AD |
| --- | --- | --- | --- | --- | --- | --- | --- | --- |
| KL | -0.02 | 0.11 | -0.20* | 0.21* | 0.15 | 0.40** | -0.41** | -0.03 |
| KW | 0.11 | 0.28** | -0.18* | 0.09 | 0.19* | 0.21* | -0.12 | 0.06 |
| KT | 0.12 | 0.23** | -0.12 | 0.14 | 0.16 | 0.31** | -0.22* | 0.13 |
| TKW | -0.03 | 0.32** | -0.16 | 0.12 | 0.19* | 0.36** | -0.36** | 0.01 |
| LWR | 0.02 | -0.04 | 0.04 | 0.06 | 0.02 | 0.20* | -0.19* | -0.08 |
| KS | 0.12 | 0.25** | 0.14 | 0.19* | 0.19* | 0.38** | -0.30** | 0.04 |
| FFD | 0.05 | 0.22** | -0.04 | -0.03 | 0.07 | 0.18* | -0.12 | 0.03 |

* Correlation is significant at the 0.05 level, ** Correlation is significant at the 0.01 level.
