# Supplementary material for: Genetic Mapping and Validation of Loci for Kernel-Related Traits in Wheat (Triticum aestivum L.)
Source: Front Plant Sci. 2021 Jun 7;12:667493. doi: 10.3389/fpls.2021.667493 (PMC8215603; doi:10.3389/fpls.2021.667493)
Supplement: Supplementary Table 6 — All quantitative trait loci for kernel traits identified in the “20828” × “SY95-71” population evaluated in different environments. [file Table_6.DOCX]

**Table S6** All quantitative trait loci for kernel traits identified in the ‘20828’ × ‘SY95-71’ population evaluated in different environments

| Trait | QTL | Environment | Chromosome | Interval | Left Marker | Right Marker | LOD | PVE (%) | Add |
| --- | --- | --- | --- | --- | --- | --- | --- | --- | --- |
| **KL** | ***QKL.sicau-2SY-1B*** | 2017CZ | 1B | 148.5-149.5 | *AX-111104674* | *AX-109379070* | 6.02 | 13.67 | -0.12 |
|  |  | 2017YA | 1B | 143.5-148.5 | *AX-110065453* | *AX-94433089* | 2.53 | 9.48 | -0.08 |
|  |  | 2018CZ | 1B | 145.5-148.5 | *AX-110065453* | *AX-94433089* | 9.85 | 21.13 | -0.20 |
|  |  | 2018YA | 1B | 143.5-148.5 | *AX-110065453* | *AX-94433089* | 6.97 | 10.52 | -0.17 |
|  |  | 2019CZ | 1B | 144.5-147.5 | *AX-110065453* | *AX-94433089* | 8.38 | 14.77 | -0.14 |
|  |  | 2019WJ | 1B | 148.5-149.5 | *AX-111104674* | *AX-109379070* | 16.88 | 25.23 | -0.24 |
|  |  | BLUP | 1B | 144.5-147.5 | *AX-110065453* | *AX-94433089* | 16.75 | 23.74 | -0.14 |
|  | *QKL.sicau-2SY-1B.1* | 2019WJ | 1B | 70.5-71.5 | *AX-94786026* | *AX-110581259* | 4.96 | 5.85 | 0.12 |
|  | *QKL.sicau-2SY-2B* | BLUP | 2B | 145.5-147.5 | *AX-94441014* | *AX-109521609* | 3.69 | 4.02 | -0.06 |
|  | ***QKL.sicau-2SY-2D.1*** | 2019CZ | 2D | 0-5.5 | *AX-110411457* | *AX-110899429* | 12.44 | 23.24 | 0.17 |
|  |  | BLUP | 2D | 0-5.5 | *AX-110012897* | *AX-110411457* | 14.08 | 18.97 | 0.12 |
|  | ***QKL.sicau-2SY-2D.2*** | 2018CZ | 2D | 8.5-13.5 | *AX-109847853* | *AX-110720701* | 8.06 | 17.50 | 0.18 |
|  |  | 2019WJ | 2D | 11.5-15.5 | *AX-109847853* | *AX-110720701* | 13.03 | 18.30 | 0.20 |
|  | ***QKL.sicau-2SY-2D.3*** | 2017CZ | 2D | 31.5-37.5 | *AX-111722527* | *AX-109421761* | 6.35 | 16.45 | 0.13 |
|  |  | 2018YA | 2D | 24.5-28.5 | *AX-108767381* | *AX-111722527* | 8.50 | 13.36 | 0.19 |
|  | *QKL.sicau-2SY-3A.1* | 2018CZ | 3A | 120.5-121.5 | *AX-110479868* | *AX-109342110* | 3.57 | 6.82 | 0.11 |
|  |  | 2019WJ | 3A | 82.5-87.5 | *AX-109594342* | *AX-86178328* | 3.19 | 3.64 | 0.09 |
|  | *QKL.sicau-2SY-3A.3* | BLUP | 3A | 71.5-74.5 | *AX-108940763* | *AX-111552673* | 4.68 | 5.23 | 0.06 |
|  | *QKL.sicau-2SY-4A* | BLUP | 4A | 146.5-148.5 | *AX-111061531* | *AX-109471718* | 4.11 | 4.62 | 0.06 |
|  | *QKL.sicau-2SY-5A* | 2017CZ | 5A | 86.5-89.5 | *AX-110979994* | *AX-111212447* | 2.83 | 6.06 | 0.08 |
|  | *QKL.sicau-2SY-5A.1* | 2018YA | 5A | 103.5-106.5 | *AX-109434576* | *AX-108889203* | 5.43 | 8.47 | 0.15 |
|  | *QKL.sicau-2SY-5A.2* | 2018YA | 5A | 108.5-110.5 | *AX-108848765* | *AX-111673092* | 9.87 | 15.83 | -0.21 |
|  | *QKL.sicau-2SY-6B* | 2017CZ | 6B | 107.5-110.5 | *AX-94831360* | *AX-110404003* | 3.68 | 7.96 | -0.09 |
|  | ***QKL.sicau-2SY-6D*** | 2018YA | 6D | 170.5-171 | *AX-110378380* | *AX-111487792* | 3.82 | 5.33 | 0.12 |
|  |  | 2019WJ | 6D | 169.5-171 | *AX-110412658* | *AX-109195537* | 5.78 | 7.07 | 0.13 |
| **KW** | *QKW.sicau-2SY-1B* | 2018CZ | 1B | 126.5-128.5 | *AX-89635557* | *AX-109478219* | 4.76 | 11.87 | -0.05 |
|  | *QKW.sicau-2SY-3D* | 2017YA | 3D | 0-5.5 | *AX-109271722* | *AX-110941549* | 3.32 | 12.32 | 0.06 |
|  | *QKW.sicau-2SY-6B* | 2019CZ | 6B | 46.5-53.5 | *AX-109461489* | *AX-111547303* | 2.60 | 5.58 | -0.05 |
|  | ***QKW.sicau-2SY-6D*** | 2017CZ | 6D | 64.5-68.5 | *AX-94618881* | *AX-110469783* | 3.07 | 11.28 | 0.05 |
|  |  | 2018CZ | 6D | 64.5-68.5 | *AX-110469783* | *AX-110066157* | 4.27 | 10.31 | 0.05 |
|  |  | 2018YA | 6D | 64.5-69.5 | *AX-94618881* | *AX-110066157* | 3.93 | 16.26 | 0.10 |
|  |  | 2019CZ | 6D | 65.5-67.5 | *AX-110469783* | *AX-110066157* | 14.55 | 39.50 | 0.13 |
|  |  | 2019WJ | 6D | 64.5-69.5 | *AX-110469783* | *AX-110066157* | 3.72 | 13.93 | 0.06 |
|  |  | BLUP | 6D | 64.5-67.5 | *AX-110469783* | *AX-110066157* | 10.88 | 32.77 | 0.05 |
| KT | *QKT.sicau-2SY-1B* | 2019WJ | 1B | 101.5-102.5 | *AX-108891924* | *AX-109887385* | 3.32 | 7.14 | 0.05 |
|  | *QKT.sicau-2SY-2B.1* | 2019CZ | 2B | 31.5-34.5 | *AX-108806563* | *AX-109835884* | 5.39 | 6.52 | -0.04 |
|  | *QKT.sicau-2SY-2B.2* | 2019WJ | 2B | 126.5-131.5 | *AX-109915619* | *AX-110934424* | 3.13 | 7.23 | -0.05 |
|  | *QKT.sicau-2SY-2B.3* | 2019CZ | 2B | 158.5-160.5 | *AX-109447372* | *AX-108826010* | 5.45 | 6.65 | -0.04 |
|  | ***QKT.sicau-2SY-2D*** | 2017CZ | 2D | 8.5-13.5 | *AX-110929471* | *AX-109847853* | 3.03 | 11.05 | 0.04 |
|  |  | 2018CZ | 2D | 5.5-9.5 | *AX-110899429* | *AX-110929471* | 13.23 | 31.72 | 0.09 |
|  |  | 2018YA | 2D | 8.5-12.5 | *AX-109847853* | *AX-110720701* | 8.71 | 29.62 | 0.12 |
|  |  | 2019CZ | 2D | 4.5-8.5 | *AX-110899429* | *AX-110929471* | 9.75 | 12.84 | 0.05 |
|  |  | 2019WJ | 2D | 6.5-9.5 | *AX-110899429* | *AX-110929471* | 11.52 | 29.29 | 0.09 |
|  |  | BLUP | 2D | 8.5-12.5 | *AX-109847853* | *AX-110720701* | 14.71 | 41.26 | 0.05 |
|  | *QKT.sicau-2SY-4A* | 2019CZ | 4A | 30.5-32.5 | *AX-111645255* | *AX-111543816* | 5.48 | 6.81 | 0.04 |
|  | *QKT.sicau-2SY-5A* | 2019CZ | 5A | 15.5-17.5 | *AX-109512380* | *AX-111572088* | 3.88 | 4.57 | 0.03 |
|  | *QKT.sicau-2SY-5D* | 2018CZ | 5D | 14.5-32.5 | *AX-89489968* | *AX-89633041* | 3.46 | 10.43 | -0.05 |
|  | *QKT.sicau-2SY-6A* | 2018CZ | 6A | 27.5-36.5 | *AX-110423063* | *AX-109420246* | 3.24 | 6.40 | 0.04 |
|  | *QKT.sicau-2SY-6B* | 2019CZ | 6B | 46.5-51.5 | *AX-111547303* | *AX-111472663* | 6.59 | 8.44 | -0.04 |
|  | *QKT.sicau-2SY-7B* | 2019CZ | 7B | 9.5-12.5 | *AX-111765415* | *AX-94537587* | 6.98 | 9.13 | 0.04 |
| **TKW** | *QTKW.sicau-2SY-1B.1* | 2019WJ | 1B | 31.5-32.5 | *AX-94872837* | *AX-89506553* | 4.89 | 8.81 | 1.61 |
|  | *QTKW.sicau-2SY-1B.2* | 2019WJ | 1B | 118.5-121.5 | *AX-108990221* | *AX-109457059* | 3.08 | 5.58 | 1.30 |
|  | *QTKW.sicau-2SY-1B.3* | 2018CZ | 1B | 157.5-168.5 | *AX-111042449* | *AX-111688280* | 2.83 | 8.04 | -1.53 |
|  | *QTKW.sicau-2SY-1D* | 2017CZ | 1D | 4.5-9.5 | *AX-109304203* | *AX-108764560* | 3.33 | 9.50 | 1.56 |
|  | *QTKW.sicau-2SY-2A* | 2019CZ | 2A | 94.5-95.5 | *AX-109311920* | *AX-111734494* | 2.70 | 9.94 | 2.12 |
|  | *QTKW.sicau-2SY-2B* | 2019WJ | 2B | 131.5-132.5 | *AX-110571313* | *AX-109578675* | 2.60 | 4.53 | -1.15 |
|  | ***QTKW.sicau-2SY-2D*** | 2017YA | 2D | 0-5.5 | *AX-110012897* | *AX-110411457* | 4.29 | 11.67 | 1.89 |
|  |  | 2017CZ | 2D | 4.5-7.5 | *AX-110411457* | *AX-110899429* | 4.05 | 11.50 | 1.72 |
|  |  | 2018CZ | 2D | 1.5-5.5 | *AX-110012897* | *AX-110411457* | 5.66 | 16.51 | 2.19 |
|  |  | 2019CZ | 2D | 1.5-5.5 | *AX-110411457* | *AX-110899429* | 8.32 | 22.98 | 3.20 |
|  |  | 2019WJ | 2D | 5.5-9.5 | *AX-110899429* | *AX-110929471* | 12.08 | 25.63 | 2.75 |
|  |  | BLUP | 2D | 1.5-5.5 | *AX-110012897* | *AX-110411457* | 9.80 | 30.61 | 1.64 |
|  | *QTKW.sicau-2SY-4B* | 2017CZ | 4B | 29.5-31.5 | *AX-110574019* | *AX-109330857* | 3.53 | 10.06 | -1.64 |
|  | *QTKW.sicau-2SY-4D* | 2019WJ | 4D | 61.5-69.5 | *AX-110572006* | *AX-109230716* | 4.20 | 7.78 | 1.51 |
|  | *QTKW.sicau-2SY-6D* | 2018CZ | 6D | 64.5-67.5 | *AX-110469783* | *AX-110066157* | 3.71 | 10.26 | 1.74 |
|  | *QTKW.sicau-2SY-7A* | 2019WJ | 7A | 95.5-69.5 | *AX-111730218* | *AX-108837168* | 5.53 | 10.95 | -1.81 |
| LWR | *QLWR.sicau-2SY-1B* | BLUP | 1B | 156.5-167.5 | *AX-111042449* | *AX-111688280* | 4.62 | 13.43 | 0.03 |
|  | *QLWR.sicau-2SY-1D* | 2019CZ | 1D | 58.5-67.5 | *AX-111515122* | *AX-109320713* | 2.74 | 3.59 | 0.02 |
|  | *QLWR.sicau-2SY-2B* | 2019CZ | 2B | 44.5-45.5 | *AX-111710268* | *AX-108766426* | 7.35 | 10.35 | 0.04 |
|  | ***QLWR.sicau-2SY-2D*** | 2019WJ | 2D | 24.5-33.5 | *AX-111722527* | *AX-109421761* | 3.96 | 9.70 | 0.04 |
|  |  | BLUP | 2D | 23.5-29.5 | *AX-108767381* | *AX-111722527* | 3.32 | 8.24 | 0.02 |
|  | *QLWR.sicau-2SY-3A* | 2019WJ | 3A | 71.5-77.5 | *AX-111552673* | *AX-111780961* | 3.99 | 9.72 | 0.04 |
|  | *QLWR.sicau-2SY-4A* | 2019WJ | 4A | 26.5-30.5 | *AX-109426161* | *AX-110626940* | 3.85 | 9.01 | -0.04 |
|  | *QLWR.sicau-2SY-6B* | 2019CZ | 6B | 46.5- 50.5 | *AX-109461489* | *AX-111547303* | 7.21 | 9.91 | 0.04 |
|  | ***QLWR.sicau-2SY-6D*** | 2017CZ | 6D | 64.5-68.5 | *AX-94618881* | *AX-110469783* | 4.07 | 11.28 | 0.05 |
|  |  | 2018YA | 6D | 64.5-68.5 | *AX-110469783* | *AX-110066157* | 2.54 | 11.63 | -0.05 |
|  |  | 2019CZ | 6D | 64.5-66.5 | *AX-94618881* | *AX-110469783* | 14.25 | 22.76 | -0.06 |
|  |  | BLUP | 6D | 64.5-67.5 | *AX-110469783* | *AX-110066157* | 3.98 | 9.92 | -0.02 |
|  | *QLWR.sicau-2SY-7B* | 2019WJ | 7B | 95.5- 101.5 | *AX-108795893* | *AX-109920134* | 5.48 | 13.02 | -0.05 |
| KS | ***QKS.sicau-2SY-1B*** | 2017CZ | 1B | 144.5- 148.5 | *AX-110065453* | *AX-94433089* | 3.17 | 7.95 | -2.00 |
|  |  | 2018CZ | 1B | 145.5- 148.5 | *AX-110065453* | *AX-94433089* | 5.22 | 11.28 | -3.67 |
|  |  | 2019WJ | 1B | 148.5- 149.5 | *AX-111104674* | *AX-109379070* | 4.11 | 9.01 | -3.22 |
|  |  | BLUP | 1B | 145.5- 148.5 | *AX-110065453* | *AX-94433089* | 7.37 | 13.86 | -1.98 |
|  | *QKS.sicau-2SY-1B.1* | 2017CZ | 1B | 27.5- 30.5 | *AX-108800083* | *AX-108960675* | 7.96 | 5.37 | 3.30 |
|  | *QKS.sicau-2SY-1B.2* | 2019CZ | 1B | 126.5- 128.5 | *AX-89635557* | *AX-109478219* | 2.86 | 7.18 | -2.42 |
|  | *QKS.sicau-2SY-1B.3* | 2018YA | 1B | 137.5-139.5 | *AX-111221126* | *AX-109359622* | 3.73 | 9.72 | -4.15 |
|  | ***QKS.sicau-2SY-2D*** | 2017CZ | 2D | 8.5- 13.5 | *AX-109847853* | *AX-110720701* | 6.00 | 3.92 | 2.82 |
|  |  | 2018CZ | 2D | 0-5.5 | *AX-110012897* | *AX-110411457* | 8.25 | 18.80 | 4.73 |
|  |  | 2019CZ | 2D | 0-5.5 | *AX-110411457* | *AX-110899429* | 7.35 | 19.14 | 3.91 |
|  |  | 2019WJ | 2D | 5.5- 9.5 | *AX-110899429* | *AX-110929471* | 7.66 | 18.11 | 4.53 |
|  |  | BLUP | 2D | 1.5- 5.5 | *AX-110411457* | *AX-110899429* | 13.47 | 27.78 | 2.80 |
|  | *QKS.sicau-2SY-2D.1* | 2017CZ | 2D | 7.5- 9.5 | *AX-110604633* | *AX-111526465* | 3.56 | 2.19 | 2.13 |
|  | *QKS.sicau-2SY-2D.1* | 2018YA | 2D | 24.5- 26.5 | *AX-108767381* | *AX-111722527* | 8.92 | 26.61 | 6.85 |
|  | *QKS.sicau-2SY-3A* | 2017CZ | 3A | 93.5- 96.5 | *AX-109295444* | *AX-86173791* | 28.21 | 30.99 | -7.93 |
|  | *QKS.sicau-2SY-3D* | 2017YA | 3D | 0- 4.5 | *AX-109271722* | *AX-110941549* | 2.69 | 11.79 | 2.32 |
|  | *QKS.sicau-2SY-5A* | 2019WJ | 5A | 107.5- 109.5 | *AX-110383644* | *AX-108848765* | 2.74 | 5.91 | 2.59 |
|  | *QKS.sicau-2SY-6A* | 2018CZ | 6A | 25.5-36.5 | *AX-109333168* | *AX-110423063* | 3.39 | 8.54 | 3.19 |
|  | ***QKS.sicau-2SY-6D*** | 2018YA | 6D | 56.5-65.5 | *AX-110667224* | *AX-94618881* | 2.68 | 6.78 | 3.45 |
|  |  | 2019CZ | 6D | 65.5-68.5 | *AX-110469783* | *AX-110066157* | 6.27 | 17.35 | 3.78 |
|  |  | 2019WJ | 6D | 64.5-69.5 | *AX-110469783* | *AX-110066157* | 4.03 | 8.88 | 3.25 |
|  |  | BLUP | 6D | 64.5-68.5 | *AX-110469783* | *AX-110066157* | 5.31 | 9.39 | 1.64 |
| FFD | ***QFFD.sicau-2SY-2D*** | 2017YA | 2D | 4.5- 8.5 | *AX-110411457* | *AX-110899429* | 2.95 | 12.22 | 0.05 |
|  |  | 2017CZ | 2D | 4.5-8.5 | AX-110899429 | AX-110929471 | 2.59 | 10.11 | 0.05 |
|  |  | 2018YA | 2D | 4.5-8.5 | AX-110899429 | AX-110899429 | 3.73 | 15.76 | 0.11 |
|  |  | 2019WJ | 2D | 0-5.5 | *AX-110411457* | *AX-110899429* | 3.08 | 14.37 | 0.05 |
|  |  | BLUP | 2D | 4.5-8.5 | *AX-110899429* | *AX-110929471* | 5.74 | 17.25 | 0.04 |
|  | ***QFFD.sicau-2SY-2D.1*** | 2018CZ | 2D | 23.5- 27.5 | *AX-108767381* | *AX-111722527* | 6.27 | 18.79 | 0.07 |
|  |  | 2019CZ | 2D | 24.5- 29.5 | *AX-108767381* | *AX-111722527* | 3.29 | 10.19 | 0.10 |
|  | *QFFD.sicau-2SY-3D* | BLUP | 3D | 81.5- 85.5 | *AX-111807575* | *AX-110987465* | 2.58 | 8.86 | 0.03 |
|  | *QFFD.sicau-2SY-7D* | 2018CZ | 7D | 78.5- 80.5 | *AX-109488029* | *AX-108739865* | 4.30 | 12.41 | 0.06 |

Note: WJ: Wenjiang

CZ: Chongzhou

YA: Ya'an

LOD: logarithm of odds

PVE: phenotype variance explained

Add: additive effect of a QTL

Repeatedly detected QTL was shown in bold
