# Supplementary material for: Genetic Mapping and Validation of Loci for Kernel-Related Traits in Wheat (Triticum aestivum L.)
Source: Front Plant Sci. 2021 Jun 7;12:667493. doi: 10.3389/fpls.2021.667493 (PMC8215603; doi:10.3389/fpls.2021.667493)
Supplement: Supplementary Table 7 — Analyses of variance for kernel-related traits. [file Table_7.DOCX]

**Table S7** Analyses of variance for kernel-related traits

| Variance component | KL | | KW | | KT | | TKW | | LWR | | KS | | FFD | |
| --- | --- | --- | --- | --- | --- | --- | --- | --- | --- | --- | --- | --- | --- | --- |
|  | Variation (%) | P | Variation (%) | P | Variation (%) | P | Variation (%) | P | Variation (%) | P | Variation (%) | P | Variation (%) | P |
| QTL |  |  |  |  |  |  |  |  |  |  |  |  |  |  |
| 1BL (*AX-109379070*) | 23.6 | ** | NS | NS | NS | NS | NS | NS | 20.6 | ** | 18.1 | ** | 3.9 | * |
| 6DS (*AX-110066157*) | NS | NS | 13.9 | ** | NS | NS | NS | NS | 8.8 | ** | 9.7 | ** | 5.3 | ** |
| 6DL (*AX-110412658*) | 15.4 | ** | NS | NS | NS | NS | NS | NS | 15.5 | ** | 9.6 | ** | 2.2 | * |
| 2DL-1 (*AX-110929471*) | 7.9 | ** | NS | NS | 18.4 | ** | 13.3 | ** | NS | NS | 10.1 | ** | 21.4 | ** |
| 2DL-2 (*AX-111722527*) | 6.7 | * | NS | NS | NS | NS | NS | NS | 11.3 | ** | NS | NS | 3.9 | NS |
| Interactions |  |  |  |  |  |  |  |  |  |  |  |  |  |  |
| 1BL * 2DL-1 | NS | NS | NS | NS | NS | NS | NS | NS | 2.3 | * | NS | NS | NS | NS |
| 1BL * 2DL-2 | NS | NS | NS | NS | 2.7 | * | NS | NS | 3.5 | * | NS | NS | NS | NS |
| 1BL * 6DS | NS | NS | NS | NS | NS | NS | NS | NS | NS | NS | 2.1 | * | NS | NS |
| 6DS * 2DL-1 | NS | NS | 3.4 | * | NS | NS | 3.3 | * | 2.1 | * | NS | NS | NS | NS |
| 6DS * 2DL-2 | NS | NS | 2.5 | * | NS | NS | NS | NS | 4.1 | * | NS | NS | NS | NS |
| 6DL * 2DL-1 | NS | NS | NS | NS | 4.5 | * | NS | NS | NS | NS | NS | NS | NS | NS |
| 6DL * 2DL-2 | NS | NS | NS | NS | 2.2 | * | 3.2 | * | NS | NS | 3.9 | ** | NS | NS |
| Total explained variance | 55.0 |  | 37.4 |  | 59.1 |  | 44.0 |  | 73.0 |  | 58.8 |  | 44.6 |  |

The ANOVA model included all major and minor QTL, and all possible two way interactions. For each trait the first column indicates the percent of variation contributed by the factor and the second column the significance of the difference. (NS P > 0.05; * P < 0.05; ** P < 0.01), Only the factors and interactions that were significant for at least one trait are shown. 1BL (*AX-109379070*): KL and KS major QTL; 6DS (*AX-110066157*): KW, KS and LWR major QTL; 6DL (*AX-110412658*): KL minor QTL; 2DL-1 (*AX-110929471*): KT, TKW, KS and FFD major QTL, and two KL minor QTL; 2DL-2 (*AX-111722527*): KL, LWR and FFD minor QTL.
