# Supplementary material for: Genetic Mapping and Validation of Loci for Kernel-Related Traits in Wheat (Triticum aestivum L.)
Source: Front Plant Sci. 2021 Jun 7;12:667493. doi: 10.3389/fpls.2021.667493 (PMC8215603; doi:10.3389/fpls.2021.667493)
Supplement: Supplementary Table 8 — Quantitative trait loci (QTL) detected in the QTL × environment (QE) interaction module for kernel-related traits in 2SY population. [file Table_8.DOCX]

**Table S8** Quantitative trait loci (QTL) detected in the QTL × environment (QE) interaction module for kernel-related traits in 2SY population

| **trait** | **Chromosome** | **Position (cM)** | **Left Marker** | **Right Marker** | **LOD (A)** | **LOD (A by E)** | **PVE (A)** | **PVE (A by E)** | **Add_E1** | **Add_E2** | **Add_E3** | **Add_E_4_** | **Add_E_5_** | **Add_E_6_** | **Detected QTL** |
| --- | --- | --- | --- | --- | --- | --- | --- | --- | --- | --- | --- | --- | --- | --- | --- |
| **KL** | 1A | 5 | *AX-110507135* | *AX-109998250* | 1.49 | 1.38 | 0.32 | 0.22 | 0.01 | 0.03 | 0.00 | 0.01 | -0.02 | -0.03 |  |
|  | 1A | 16 | *AX-110549046* | *AX-108770405* | 1.36 | 1.35 | 0.29 | 0.22 | 0.01 | 0.01 | 0.00 | 0.02 | 0.00 | -0.04 |  |
|  | 1A | 37 | *AX-109469190* | *AX-111484839* | 0.94 | 1.68 | 0.20 | 0.37 | 0.02 | 0.03 | -0.02 | 0.03 | -0.03 | -0.02 |  |
|  | 1A | 28 | *AX-94991487* | *AX-110559766* | 3.40 | 0.81 | 0.71 | 0.03 | 0.01 | 0.01 | -0.01 | 0.00 | 0.00 | -0.01 |  |
|  | 1B | 46 | *AX-95150128* | *AX-94518238* | 1.03 | 3.23 | 0.21 | 0.46 | 0.06 | 0.00 | -0.02 | -0.02 | 0.00 | -0.02 |  |
|  | 1B | 71 | *AX-94786026* | *AX-110581259* | 1.84 | 2.84 | 0.39 | 0.56 | -0.03 | -0.01 | -0.01 | -0.02 | -0.01 | 0.07 | *QKL.sicau-2SY-1B.1* |
|  | 1B | 146 | *AX-110065453* | *AX-94433089* | 40.57 | 11.09 | 9.77 | 2.17 | -0.01 | 0.04 | -0.08 | -0.03 | -0.02 | 0.11 | ***QKL.sicau-2SY-1B*** |
|  | 1B | 214 | *AX-110666954* | *AX-110415999* | 1.20 | 1.31 | 0.25 | 0.21 | -0.02 | -0.01 | -0.01 | 0.00 | 0.02 | 0.03 |  |
|  | 1B | 15 | *AX-109509313* | *AX-108726041* | 1.74 | 1.02 | 0.37 | 0.12 | 0.02 | 0.00 | -0.02 | -0.01 | -0.01 | 0.02 |  |
|  | 1B | 44 | *AX-109618461* | *AX-110000934* | 1.22 | 1.51 | 0.26 | 0.22 | 0.03 | 0.00 | -0.02 | -0.01 | 0.00 | 0.02 |  |
|  | 2A | 40 | *AX-108892553* | *AX-108750376* | 1.62 | 1.11 | 0.34 | 0.41 | 0.03 | -0.02 | -0.01 | -0.04 | 0.03 | 0.00 |  |
|  | 2A | 44 | *AX-109074831* | *AX-86179766* | 1.57 | 1.87 | 0.32 | 0.57 | 0.03 | -0.02 | 0.00 | -0.05 | 0.04 | -0.01 |  |
|  | 2A | 54 | *AX-111736141* | *AX-109512780* | 3.26 | 0.44 | 0.69 | 0.07 | 0.02 | 0.00 | -0.01 | 0.00 | 0.00 | -0.01 |  |
|  | 2A | 60 | *AX-109990976* | *AX-110407291* | 4.49 | 1.01 | 0.92 | 0.16 | 0.03 | 0.01 | -0.01 | -0.01 | 0.00 | -0.03 |  |
|  | 2A | 72 | *AX-109913197* | *AX-111715216* | 0.59 | 2.26 | 0.12 | 0.41 | 0.02 | 0.01 | -0.01 | -0.05 | 0.03 | 0.00 |  |
|  | 2A | 79 | *AX-109842387* | *AX-108918456* | 4.85 | 0.99 | 1.00 | 0.14 | 0.03 | 0.00 | -0.01 | 0.00 | -0.01 | -0.02 |  |
|  | 2A | 86 | *AX-108917836* | *AX-109950638* | 4.44 | 0.52 | 0.86 | 0.09 | 0.02 | 0.00 | -0.01 | 0.00 | 0.00 | -0.01 |  |
|  | 2A | 93 | *AX-110990867* | *AX-109311920* | 3.22 | 1.25 | 0.63 | 0.30 | 0.03 | 0.01 | -0.01 | -0.03 | 0.02 | -0.03 |  |
|  | 2A | 102 | *AX-109417714* | *AX-110949248* | 3.61 | 1.12 | 0.67 | 0.25 | 0.04 | 0.00 | 0.00 | -0.03 | 0.01 | -0.02 |  |
|  | 2A | 105 | *AX-109438714* | *AX-110946129* | 2.69 | 1.03 | 0.48 | 0.25 | 0.03 | 0.00 | -0.01 | -0.03 | 0.02 | -0.01 |  |
|  | 2A | 107 | *AX-108865385* | *AX-108833449* | 6.63 | 1.25 | 1.32 | 0.09 | -0.02 | 0.01 | 0.00 | -0.01 | 0.02 | -0.01 |  |
|  | 2B | 45 | *AX-111710268* | *AX-108766426* | 0.93 | 1.81 | 0.20 | 0.25 | 0.00 | -0.01 | -0.02 | -0.02 | 0.04 | 0.00 |  |
|  | 2B | 54 | *AX-110435861* | *AX-109428627* | 0.48 | 2.28 | 0.10 | 0.37 | 0.01 | 0.01 | -0.02 | -0.02 | 0.04 | -0.02 |  |
|  | 2B | 72 | *AX-110426897* | *AX-111597275* | 1.72 | 1.68 | 0.36 | 0.48 | 0.02 | -0.02 | -0.03 | -0.02 | 0.05 | -0.02 |  |
|  | 2B | 9 | *AX-111640258* | *AX-108972533* | 2.90 | 0.39 | 0.61 | 0.03 | 0.01 | 0.00 | 0.00 | 0.01 | 0.00 | -0.01 |  |
|  | 2B | 109 | *AX-111137915* | *AX-109377141* | 2.34 | 1.04 | 0.49 | 0.24 | -0.02 | 0.04 | 0.01 | 0.00 | -0.02 | 0.01 |  |
|  | 2B | 120 | *AX-109921595* | *AX-108980566* | 2.06 | 0.89 | 0.44 | 0.11 | 0.00 | 0.02 | 0.00 | -0.02 | 0.01 | 0.00 |  |
|  | 2B | 137 | *AX-110396567* | *AX-108752545* | 1.96 | 1.10 | 0.41 | 0.12 | 0.01 | 0.02 | -0.02 | 0.00 | 0.01 | -0.01 |  |
|  | 2B | 141 | *AX-111019809* | *AX-109451490* | 1.84 | 1.51 | 0.39 | 0.17 | 0.01 | 0.03 | -0.02 | 0.01 | -0.01 | -0.01 |  |
|  | 2B | 149 | *AX-109521609* | *AX-111457622* | 3.27 | 2.09 | 0.68 | 0.13 | 0.01 | 0.02 | -0.01 | 0.01 | -0.01 | -0.01 |  |
|  | 2B | 190 | *AX-110462784* | *AX-109817336* | 1.31 | 1.25 | 0.28 | 0.25 | 0.01 | 0.03 | 0.02 | -0.01 | -0.02 | -0.01 |  |
|  | 2D | 49 | *AX-109417243* | *AX-109294613* | 2.47 | 0.51 | 0.52 | 0.10 | -0.01 | 0.00 | 0.01 | 0.02 | -0.02 | -0.01 |  |
|  | 2D | 4 | *AX-110012897* | *AX-110411457* | 13.29 | 13.52 | 2.91 | 1.83 | -0.03 | -0.02 | -0.03 | -0.06 | 0.10 | -0.02 | ***QKL.sicau-2SY-2D.1*** |
|  | 2D | 25 | *AX-108767381* | *AX-111722527* | 18.70 | 5.09 | 4.17 | 3.18 | -0.06 | -0.06 | 0.05 | 0.10 | -0.05 | 0.08 | ***QKL.sicau-2SY-2D.3*** |
|  | 2D | 36 | *AX-111722527* | *AX-109421761* | 3.35 | 4.59 | 0.71 | 0.54 | 0.06 | 0.01 | -0.03 | -0.01 | 0.00 | -0.03 |  |
|  | 2D | 80 | *AX-110409454* | *AX-110991640* | 2.57 | 0.43 | 0.53 | 0.25 | 0.02 | -0.01 | 0.00 | -0.04 | 0.02 | 0.00 |  |
|  | 3A | 31 | *AX-109393734* | *AX-109848595* | 1.44 | 1.30 | 0.30 | 0.19 | 0.01 | 0.02 | 0.00 | -0.02 | 0.02 | -0.02 |  |
|  | 3A | 62 | *AX-111799835* | *AX-110407102* | 3.88 | 1.65 | 0.81 | 0.30 | 0.01 | 0.01 | 0.00 | 0.02 | 0.02 | -0.04 |  |
|  | 3A | 72 | *AX-108940763* | *AX-111552673* | 10.88 | 2.91 | 2.32 | 0.37 | -0.02 | -0.02 | -0.03 | 0.01 | 0.01 | 0.04 | *QKL.sicau-2SY-3A.3* |
|  | 3A | 107 | *AX-111087163* | *AX-108854601* | 1.74 | 1.26 | 0.37 | 0.18 | 0.01 | 0.00 | -0.02 | 0.00 | 0.03 | -0.01 |  |
|  | 3A | 51 | *AX-111541089* | *AX-108819775* | 3.00 | 0.97 | 0.63 | 0.37 | 0.01 | 0.00 | -0.04 | -0.03 | 0.03 | 0.02 |  |
|  | 3B | 11 | *AX-111045342* | *AX-110928522* | 1.34 | 1.85 | 0.28 | 0.33 | 0.00 | 0.00 | 0.02 | -0.05 | 0.02 | 0.02 |  |
|  | 3B | 35 | *AX-110561047* | *AX-110432985* | 1.30 | 1.28 | 0.27 | 0.34 | -0.02 | -0.02 | 0.02 | -0.04 | 0.02 | 0.01 |  |
|  | 3B | 46 | *AX-110599784* | *AX-111135059* | 0.03 | 2.73 | 0.01 | 0.60 | 0.01 | -0.03 | 0.01 | -0.05 | 0.05 | 0.00 |  |
|  | 3B | 50 | *AX-110042579* | *AX-110625472* | 0.03 | 2.48 | 0.01 | 0.48 | 0.01 | -0.02 | 0.02 | -0.05 | 0.04 | 0.00 |  |
|  | 3B | 64 | *AX-94820269* | *AX-110001564* | 0.40 | 2.16 | 0.09 | 0.45 | 0.03 | -0.01 | 0.01 | -0.05 | 0.02 | 0.01 |  |
|  | 3B | 77 | *AX-108868950* | *AX-108914541* | 0.19 | 2.34 | 0.04 | 0.62 | 0.03 | -0.02 | 0.03 | -0.06 | 0.03 | 0.00 |  |
|  | 3B | 94 | *AX-109391294* | *AX-110453138* | 2.67 | 1.09 | 0.53 | 0.22 | 0.01 | -0.02 | -0.01 | -0.02 | 0.04 | 0.00 |  |
|  | 3D | 84 | *AX-110987465* | *AX-111109273* | 2.24 | 0.50 | 0.45 | 0.19 | 0.02 | 0.00 | -0.02 | -0.02 | 0.02 | 0.00 |  |
|  | 3D | 0 | *AX-109271722* | *AX-110941549* | 1.70 | 0.91 | 0.36 | 0.11 | 0.00 | 0.02 | -0.01 | 0.01 | -0.03 | 0.00 |  |
|  | 4A | 20 | *AX-108771516* | *AX-111099057* | 3.45 | 0.50 | 0.73 | 0.01 | 0.00 | 0.01 | -0.01 | 0.00 | 0.00 | -0.01 |  |
|  | 4A | 26 | *AX-109867908* | *AX-110580622* | 6.48 | 0.93 | 1.36 | 0.07 | 0.01 | -0.01 | -0.01 | 0.00 | 0.01 | -0.01 |  |
|  | 4A | 63 | *AX-110320735* | *AX-108772504* | 4.39 | 0.50 | 0.90 | 0.13 | 0.02 | 0.01 | -0.01 | -0.02 | 0.01 | -0.01 |  |
|  | 4A | 76 | *AX-109533701* | *AX-109391536* | 0.98 | 1.55 | 0.20 | 0.39 | 0.01 | 0.03 | 0.00 | -0.05 | 0.01 | 0.02 |  |
|  | 4A | 126 | *AX-109415651* | *AX-111479307* | 3.59 | 0.87 | 0.76 | 0.09 | -0.01 | -0.01 | 0.02 | -0.01 | 0.00 | 0.01 |  |
|  | 4A | 148 | *AX-111061531* | *AX-109471718* | 3.92 | 2.16 | 0.82 | 0.23 | -0.04 | 0.00 | 0.01 | -0.01 | 0.01 | 0.02 | *QKL.sicau-2SY-4A* |
|  | 4A | 168 | *AX-109926421* | *AX-109384787* | 2.94 | 2.42 | 0.58 | 0.24 | -0.03 | 0.02 | 0.02 | -0.02 | -0.01 | 0.01 |  |
|  | 4A | 174 | *AX-110913699* | *AX-110481242* | 1.90 | 1.92 | 0.40 | 0.26 | -0.02 | 0.02 | 0.02 | -0.03 | -0.01 | 0.01 |  |
|  | 4B | 51 | *AX-110428871* | *AX-110488730* | 2.42 | 1.01 | 0.51 | 0.16 | -0.01 | -0.01 | -0.02 | 0.01 | 0.03 | 0.00 |  |
|  | 4B | 52 | *AX-109637078* | *AX-109861624* | 4.73 | 0.93 | 0.98 | 0.38 | 0.03 | -0.02 | -0.01 | -0.04 | 0.03 | -0.01 |  |
|  | 4B | 59 | *AX-111190784* | *AX-109324513* | 3.82 | 0.88 | 0.80 | 0.28 | 0.01 | -0.03 | 0.00 | -0.03 | 0.03 | 0.00 |  |
|  | 4D | 36 | *AX-110937266* | *AX-110005953* | 1.61 | 1.12 | 0.34 | 0.15 | 0.00 | 0.00 | 0.03 | -0.02 | 0.00 | -0.02 |  |
|  | 5A | 31 | *AX-110006805* | *AX-111616054* | 2.01 | 2.09 | 0.42 | 0.29 | 0.03 | 0.03 | 0.00 | -0.03 | 0.00 | -0.02 |  |
|  | 5A | 44 | *AX-111103432* | *AX-108734535* | 3.40 | 1.63 | 0.72 | 0.14 | 0.02 | 0.01 | 0.00 | 0.00 | 0.01 | -0.03 |  |
|  | 5A | 57 | *AX-109362376* | *AX-108878364* | 3.44 | 1.01 | 0.73 | 0.08 | 0.01 | 0.00 | 0.01 | -0.02 | 0.01 | 0.00 |  |
|  | 5A | 71 | *AX-110972140* | *AX-109907697* | 2.88 | 0.86 | 0.61 | 0.13 | 0.01 | 0.02 | 0.02 | -0.01 | -0.02 | 0.00 |  |
|  | 5A | 87 | *AX-110979994* | *AX-111212447* | 2.67 | 0.88 | 0.57 | 0.10 | 0.01 | 0.00 | 0.01 | -0.02 | 0.00 | 0.01 | *QKL.sicau-2SY-5A* |
|  | 5A | 110 | *AX-108848765* | *AX-111673092* | 0.32 | 2.85 | 0.06 | 0.80 | 0.01 | -0.03 | 0.00 | -0.07 | 0.04 | 0.04 | *QKL.sicau-2SY-5A.2* |
|  | 5A | 125 | *AX-110739984* | *AX-109622137* | 1.86 | 1.16 | 0.39 | 0.35 | 0.01 | -0.03 | -0.01 | -0.03 | 0.03 | 0.02 |  |
|  | 5A | 156 | *AX-108728615* | *AX-110951051* | 0.34 | 3.07 | 0.07 | 0.80 | -0.01 | -0.01 | 0.03 | -0.07 | 0.03 | 0.04 |  |
|  | 5B | 0 | *AX-108791526* | *AX-111490382* | 4.18 | 0.97 | 0.88 | 0.28 | 0.03 | 0.01 | -0.04 | -0.02 | 0.02 | 0.00 |  |
|  | 5B | 24 | *AX-110384815* | *AX-94755266* | 3.09 | 1.19 | 0.62 | 0.20 | 0.04 | 0.00 | -0.02 | 0.00 | 0.00 | -0.01 |  |
|  | 5B | 29 | *AX-109370636* | *AX-110001926* | 0.98 | 1.55 | 0.21 | 0.31 | 0.04 | 0.00 | -0.03 | 0.01 | 0.00 | -0.02 |  |
|  | 5B | 94 | *AX-109648386* | *AX-111627154* | 4.19 | 0.44 | 0.84 | 0.09 | 0.02 | -0.01 | -0.01 | 0.00 | 0.01 | -0.01 |  |
|  | 5B | 89 | *AX-110439607* | *AX-109855795* | 2.70 | 2.23 | 0.57 | 0.37 | -0.01 | 0.04 | -0.04 | 0.02 | -0.02 | 0.01 |  |
|  | 5B | 94 | *AX-108776572* | *AX-111711490* | 2.90 | 2.59 | 0.61 | 0.42 | -0.01 | 0.03 | -0.04 | 0.03 | -0.02 | 0.01 |  |
|  | 5D | 33 | *AX-89489968* | *AX-89633041* | 2.79 | 1.01 | 0.58 | 0.21 | 0.02 | 0.01 | -0.04 | 0.01 | 0.00 | -0.01 |  |
|  | 5D | 64 | *AX-108822269* | *AX-111388087* | 1.05 | 1.87 | 0.22 | 0.38 | 0.00 | 0.03 | -0.03 | 0.02 | 0.01 | -0.04 |  |
|  | 5D | 114 | *AX-110824131* | *AX-109034925* | 2.82 | 1.00 | 0.59 | 0.34 | 0.01 | 0.01 | 0.02 | -0.04 | 0.02 | -0.03 |  |
|  | 6A | 0 | *AX-108834300* | *AX-109026744* | 1.87 | 0.94 | 0.40 | 0.10 | -0.01 | -0.02 | 0.02 | -0.01 | 0.00 | 0.00 |  |
|  | 6A | 56 | *AX-89321181* | *AX-110947561* | 2.18 | 2.15 | 0.45 | 0.63 | -0.03 | 0.00 | 0.07 | 0.01 | -0.03 | -0.01 |  |
|  | 6B | 109 | *AX-94831360* | *AX-110404003* | 3.10 | 0.67 | 0.65 | 0.07 | 0.00 | -0.02 | 0.00 | -0.01 | 0.01 | 0.01 | *QKL.sicau-2SY-6B* |
|  | 6B | 118 | *AX-108816205* | *AX-111513561* | 3.22 | 0.75 | 0.67 | 0.02 | 0.00 | 0.00 | 0.01 | 0.00 | -0.01 | 0.01 |  |
|  | 6B | 133 | *AX-108926385* | *AX-110427037* | 1.01 | 1.76 | 0.21 | 0.28 | -0.01 | -0.02 | 0.00 | 0.03 | -0.03 | 0.02 |  |
|  | 6B | 35 | *AX-111112860* | *AX-111756688* | 1.00 | 2.11 | 0.21 | 0.45 | -0.01 | -0.03 | -0.03 | 0.00 | 0.05 | 0.02 |  |
|  | 6B | 80 | *AX-109876626* | *AX-110515622* | 2.18 | 2.02 | 0.46 | 0.31 | 0.00 | -0.03 | -0.01 | 0.04 | 0.02 | -0.01 |  |
|  | 6D | 37 | *AX-111026969* | *AX-109353709* | 0.23 | 2.80 | 0.05 | 0.58 | 0.00 | -0.05 | 0.04 | -0.03 | 0.04 | 0.00 |  |
|  | 6D | 94 | *AX-109400957* | *AX-111157636* | 3.76 | 0.44 | 0.79 | 0.08 | -0.01 | -0.01 | 0.02 | 0.00 | -0.01 | 0.01 |  |
|  | 6D | 170 | *AX-110412658* | *AX-109195537* | 2.19 | 3.45 | 0.46 | 0.93 | -0.03 | 0.00 | 0.03 | -0.02 | -0.03 | 0.08 | ***QKL.sicau-2SY-6D*** |
|  | 7A | 18 | *AX-111576650* | *AX-108812645* | 3.21 | 0.76 | 0.68 | 0.20 | 0.01 | 0.00 | -0.01 | -0.03 | 0.02 | 0.01 |  |
|  | 7A | 24 | *AX-111040783* | *AX-109431738* | 4.01 | 0.73 | 0.84 | 0.18 | 0.01 | 0.01 | -0.01 | -0.04 | 0.02 | 0.01 |  |
|  | 7A | 31 | *AX-110590540* | *AX-111644847* | 4.11 | 0.52 | 0.87 | 0.16 | 0.00 | 0.01 | 0.00 | -0.04 | 0.01 | 0.01 |  |
|  | 7A | 60 | *AX-109306246* | *AX-109492030* | 1.79 | 1.48 | 0.37 | 0.38 | -0.01 | 0.00 | 0.02 | -0.05 | 0.01 | 0.03 |  |
|  | 7A | 18 | *AX-110515851* | *AX-111203913* | 3.11 | 0.68 | 0.64 | 0.23 | 0.04 | -0.01 | -0.02 | -0.03 | 0.00 | 0.00 |  |
|  | 7A | 23 | *AX-109598170* | *AX-109465295* | 3.93 | 0.55 | 0.81 | 0.18 | 0.03 | -0.01 | -0.02 | -0.01 | 0.01 | 0.00 |  |
|  | 7A | 26 | *AX-110418031* | *AX-109417084* | 3.34 | 0.52 | 0.68 | 0.14 | 0.02 | 0.00 | -0.03 | 0.00 | 0.00 | 0.00 |  |
|  | 7A | 31 | *AX-111518897* | *AX-111215580* | 2.04 | 0.64 | 0.43 | 0.14 | 0.03 | -0.01 | -0.02 | 0.00 | -0.01 | 0.00 |  |
|  | 7A | 37 | *AX-110677006* | *AX-109824443* | 2.09 | 0.54 | 0.44 | 0.14 | 0.03 | -0.01 | -0.02 | 0.00 | -0.01 | -0.01 |  |
|  | 7A | 51 | *AX-110458473* | *AX-111253793* | 2.33 | 0.19 | 0.48 | 0.08 | 0.02 | -0.01 | -0.01 | -0.01 | 0.01 | 0.00 |  |
|  | 7A | 196 | *AX-110463075* | *AX-109078499* | 1.92 | 1.03 | 0.41 | 0.12 | 0.02 | -0.01 | 0.01 | 0.00 | 0.00 | -0.02 |  |
|  | 7B | 146 | *AX-109325469* | *AX-110390771* | 2.42 | 1.53 | 0.51 | 0.13 | -0.01 | 0.02 | -0.01 | -0.01 | 0.02 | -0.02 |  |
|  | 7B | 1 | *AX-110935680* | *AX-108794246* | 3.16 | 1.32 | 0.66 | 0.10 | -0.01 | -0.01 | 0.01 | -0.02 | 0.01 | 0.01 |  |
|  | 7B | 25 | *AX-109926256* | *AX-89540697* | 2.25 | 0.95 | 0.48 | 0.12 | 0.00 | 0.00 | 0.02 | -0.03 | 0.00 | 0.01 |  |
|  | 7D | 41 | *AX-111902534* | *AX-110443275* | 2.70 | 1.16 | 0.56 | 0.20 | 0.01 | 0.04 | -0.02 | -0.01 | -0.01 | -0.01 |  |
|  | 7D | 53 | *AX-108814055* | *AX-108956953* | 0.67 | 1.91 | 0.14 | 0.46 | 0.03 | 0.04 | -0.01 | -0.04 | -0.02 | 0.01 |  |
|  | 7D | 60 | *AX-109436921* | *AX-108739145* | 2.04 | 1.72 | 0.42 | 0.36 | 0.04 | 0.03 | -0.01 | -0.03 | -0.02 | 0.00 |  |
|  | 7D | 76 | *AX-109276388* | *AX-109953422* | 2.39 | 1.19 | 0.51 | 0.19 | 0.02 | 0.02 | 0.00 | -0.02 | -0.02 | 0.00 |  |
|  | 7D | 81 | *AX-108912162* | *AX-108797663* | 2.38 | 1.29 | 0.51 | 0.19 | 0.02 | 0.02 | 0.01 | -0.02 | -0.03 | 0.00 |  |
|  | 7D | 104 | *AX-108777441* | *AX-111793251* | 2.14 | 0.44 | 0.45 | 0.10 | 0.01 | 0.02 | 0.00 | -0.01 | -0.01 | -0.01 |  |
| **KW** | 1A | 4 | *AX-108848169* | *AX-111071514* | 0.50 | 2.73 | 0.11 | 0.50 | 0.01 | 0.03 | 0.01 | -0.01 | -0.01 | -0.03 |  |
|  | 1A | 30 | *AX-109383322* | *AX-108873053* | 2.61 | 1.45 | 0.57 | 0.24 | 0.01 | 0.00 | 0.00 | 0.01 | 0.01 | -0.02 |  |
|  | 1A | 41 | *AX-110998966* | *AX-109006135* | 2.95 | 1.09 | 0.65 | 0.12 | 0.01 | 0.00 | -0.01 | 0.01 | 0.00 | -0.01 |  |
|  | 1A | 48 | *AX-111173698* | *AX-109823197* | 2.15 | 1.15 | 0.47 | 0.06 | 0.00 | 0.00 | 0.01 | 0.00 | 0.00 | -0.01 |  |
|  | 1A | 14 | *AX-109836978* | *AX-110488387* | 1.38 | 1.49 | 0.30 | 0.22 | 0.01 | 0.02 | 0.00 | 0.00 | -0.02 | 0.00 |  |
|  | 1B | 22 | *AX-111626947* | *AX-108973027* | 0.87 | 3.39 | 0.18 | 0.90 | 0.01 | -0.02 | 0.00 | -0.03 | 0.00 | 0.04 |  |
|  | 1B | 47 | *AX-94645635* | *AX-109849211* | 1.47 | 2.28 | 0.32 | 0.50 | 0.00 | -0.02 | 0.01 | -0.02 | 0.00 | 0.03 |  |
|  | 1B | 55 | *AX-89352854* | *Xgwm11* | 2.74 | 2.59 | 0.53 | 0.49 | 0.01 | -0.01 | 0.00 | -0.02 | 0.00 | 0.03 |  |
|  | 1B | 60 | *AX-86176488* | *AX-94711657* | 3.64 | 3.43 | 0.73 | 0.63 | 0.00 | -0.02 | 0.01 | -0.02 | 0.00 | 0.03 |  |
|  | 1B | 72 | *AX-110570180* | *AX-109605973* | 2.16 | 2.82 | 0.47 | 0.48 | -0.01 | -0.02 | 0.01 | -0.01 | -0.01 | 0.03 |  |
|  | 1B | 99 | *AX-109308955* | *AX-110970287* | 2.95 | 2.58 | 0.65 | 0.41 | 0.00 | -0.01 | 0.01 | -0.02 | -0.01 | 0.03 |  |
|  | 1B | 128 | *AX-89635557* | *AX-109478219* | 4.86 | 3.86 | 1.07 | 0.37 | 0.01 | 0.01 | -0.03 | -0.01 | -0.01 | 0.02 | *QKW.sicau-2SY-1B* |
|  | 1B | 138 | *AX-109334796* | *AX-111221126* | 2.16 | 1.38 | 0.47 | 0.21 | 0.00 | 0.00 | 0.02 | -0.02 | 0.00 | 0.01 |  |
|  | 1B | 146 | *AX-110065453* | *AX-94433089* | 1.49 | 1.06 | 0.33 | 0.09 | -0.01 | 0.00 | 0.01 | -0.01 | 0.01 | 0.00 |  |
|  | 1B | 200 | *AX-110414986* | *AX-110666954* | 3.29 | 1.23 | 0.72 | 0.12 | 0.00 | 0.01 | -0.02 | 0.00 | 0.00 | 0.01 |  |
|  | 1B | 26 | *AX-109335890* | *AX-94871279* | 2.73 | 1.31 | 0.60 | 0.06 | 0.00 | 0.00 | 0.01 | 0.01 | 0.00 | -0.01 |  |
|  | 1B | 40 | *AX-109618461* | *AX-110000934* | 2.67 | 1.16 | 0.59 | 0.08 | 0.01 | 0.00 | 0.00 | 0.00 | -0.01 | -0.01 |  |
|  | 1D | 99 | *AX-108797722* | *AX-111504007* | 1.38 | 2.80 | 0.30 | 0.54 | 0.00 | 0.00 | -0.02 | -0.02 | 0.02 | 0.03 |  |
|  | 2A | 0 | *AX-109977789* | *AX-110944608* | 0.68 | 2.27 | 0.15 | 0.42 | 0.01 | 0.02 | -0.02 | -0.01 | -0.02 | 0.01 |  |
|  | 2A | 64 | *AX-109882240* | *AX-111464687* | 2.07 | 1.25 | 0.41 | 0.10 | 0.01 | 0.01 | -0.01 | 0.00 | -0.01 | 0.00 |  |
|  | 2A | 66 | *AX-111508694* | *AX-111092616* | 1.87 | 1.16 | 0.40 | 0.08 | 0.01 | 0.01 | 0.00 | 0.00 | -0.01 | 0.00 |  |
|  | 2A | 68 | *AX-109404528* | *AX-108932182* | 2.29 | 2.71 | 0.48 | 0.30 | 0.01 | 0.02 | 0.01 | -0.01 | -0.01 | -0.01 |  |
|  | 2A | 74 | *AX-109378069* | *AX-111576466* | 2.17 | 1.51 | 0.44 | 0.14 | 0.00 | 0.02 | -0.01 | -0.01 | 0.01 | 0.00 |  |
|  | 2A | 76 | *AX-109291631* | *AX-109631196* | 2.06 | 2.32 | 0.44 | 0.26 | 0.01 | 0.02 | 0.00 | -0.01 | -0.01 | -0.01 |  |
|  | 2A | 84 | *AX-111014053* | *AX-108917836* | 0.95 | 1.93 | 0.21 | 0.29 | -0.02 | 0.02 | 0.00 | 0.01 | -0.01 | 0.01 |  |
|  | 2A | 98 | *AX-111704483* | *AX-109999116* | 0.95 | 1.62 | 0.20 | 0.24 | -0.01 | 0.02 | -0.01 | -0.01 | 0.00 | 0.01 |  |
|  | 2B | 49 | *AX-110489945* | *AX-111001071* | 0.71 | 2.40 | 0.16 | 0.47 | -0.02 | 0.01 | 0.00 | 0.03 | -0.02 | 0.00 |  |
|  | 2D | 31 | *AX-109906393* | *AX-109402447* | 2.15 | 2.28 | 0.47 | 0.44 | 0.01 | 0.01 | 0.00 | 0.01 | 0.02 | -0.03 |  |
|  | 2D | 13 | *AX-110558888* | *AX-111097388* | 1.13 | 1.42 | 0.25 | 0.16 | -0.01 | 0.00 | 0.00 | 0.01 | 0.01 | -0.01 |  |
|  | 2D | 4 | *AX-110012897* | *AX-110411457* | 4.33 | 2.60 | 0.94 | 0.36 | 0.01 | 0.01 | -0.01 | 0.01 | 0.02 | -0.02 |  |
|  | 2D | 17 | *AX-111516380* | *AX-109459272* | 2.54 | 1.39 | 0.55 | 0.39 | -0.01 | 0.01 | -0.01 | 0.03 | 0.00 | -0.02 |  |
|  | 2D | 36 | *AX-111722527* | *AX-109421761* | 2.10 | 1.45 | 0.45 | 0.47 | -0.01 | 0.01 | 0.00 | 0.03 | -0.01 | -0.02 |  |
|  | 3A | 55 | *AX-110643660* | *AX-109406442* | 1.11 | 3.01 | 0.24 | 0.48 | -0.02 | -0.01 | -0.01 | 0.02 | -0.01 | 0.02 |  |
|  | 3A | 79 | *AX-108757584* | *AX-111449905* | 1.43 | 1.72 | 0.31 | 0.19 | -0.02 | 0.00 | 0.01 | 0.00 | 0.00 | 0.01 |  |
|  | 3B | 61 | *AX-111001001* | *AX-111564047* | 2.04 | 1.54 | 0.41 | 0.14 | 0.01 | 0.01 | -0.01 | 0.00 | -0.01 | -0.01 |  |
|  | 3B | 67 | *AX-108757678* | *AX-111007091* | 2.26 | 1.49 | 0.45 | 0.09 | 0.01 | 0.01 | 0.00 | 0.00 | -0.01 | -0.01 |  |
|  | 3B | 78 | *AX-108914541* | *AX-110515429* | 1.72 | 1.20 | 0.36 | 0.11 | 0.01 | 0.00 | -0.01 | 0.00 | 0.00 | 0.00 |  |
|  | 3B | 47 | *AX-110993960* | *AX-108783900* | 2.63 | 0.97 | 0.58 | 0.07 | 0.01 | 0.01 | -0.01 | 0.01 | -0.01 | 0.00 |  |
|  | 3D | 19 | *AX-111383164* | *AX-109900541* | 1.76 | 1.44 | 0.39 | 0.26 | -0.02 | 0.00 | -0.01 | 0.00 | 0.03 | 0.00 |  |
|  | 3D | 49 | *AX-89576720* | *AX-111916664* | 1.90 | 1.22 | 0.42 | 0.18 | 0.00 | 0.00 | -0.02 | 0.01 | 0.01 | 0.00 |  |
|  | 3D | 54 | *AX-89337262* | *AX-110042483* | 2.87 | 1.67 | 0.52 | 0.20 | -0.01 | 0.00 | -0.01 | 0.01 | 0.02 | 0.00 |  |
|  | 3D | 64 | *AX-109884133* | *AX-110923174* | 2.54 | 1.60 | 0.56 | 0.17 | 0.00 | 0.00 | -0.02 | 0.00 | 0.01 | 0.01 |  |
|  | 3D | 0 | *AX-109271722* | *AX-110941549* | 2.19 | 3.72 | 0.49 | 0.86 | -0.02 | 0.04 | 0.00 | 0.02 | -0.01 | -0.02 | *QKW.sicau-2SY-3D* |
|  | 4A | 83 | *AX-109935326* | *AX-109415651* | 0.88 | 1.62 | 0.19 | 0.29 | -0.01 | 0.01 | -0.01 | -0.02 | 0.01 | 0.01 |  |
|  | 4B | 6 | *AX-109110130* | *AX-111168324* | 0.88 | 3.62 | 0.20 | 0.72 | -0.03 | 0.01 | 0.01 | 0.03 | -0.01 | -0.01 |  |
|  | 4B | 76 | *AX-109287209* | *AX-111064477* | 2.01 | 2.78 | 0.44 | 0.27 | -0.02 | 0.00 | -0.01 | 0.01 | 0.00 | 0.02 |  |
|  | 4D | 12 | *AX-110937266* | *AX-110005953* | 1.91 | 0.66 | 0.40 | 0.10 | 0.00 | -0.01 | -0.01 | 0.01 | 0.00 | 0.01 |  |
|  | 4D | 65 | *AX-110572006* | *AX-109230716* | 2.67 | 2.44 | 0.57 | 0.39 | -0.02 | 0.02 | -0.01 | 0.01 | -0.01 | 0.02 |  |
|  | 4D | 90 | *AX-109583763* | *AX-169335640* | 0.35 | 2.60 | 0.08 | 0.51 | -0.02 | 0.02 | 0.01 | 0.00 | -0.03 | 0.02 |  |
|  | 4D | 127 | *AX-110380647* | *AX-108801189* | 0.68 | 2.37 | 0.15 | 0.75 | -0.01 | 0.01 | 0.00 | -0.04 | 0.00 | 0.03 |  |
|  | 4D | 131 | *AX-108934138* | *AX-111470564* | 0.83 | 3.06 | 0.18 | 0.80 | -0.01 | 0.01 | 0.00 | -0.03 | 0.00 | 0.04 |  |
|  | 5A | 3 | *AX-111070969* | *AX-109859109* | 3.22 | 2.86 | 0.71 | 0.21 | 0.01 | 0.00 | -0.01 | 0.00 | 0.01 | -0.02 |  |
|  | 5A | 14 | *AX-108948329* | *AX-94441001* | 1.68 | 1.71 | 0.37 | 0.15 | 0.01 | -0.01 | -0.01 | 0.00 | 0.01 | -0.01 |  |
|  | 5A | 102 | *AX-109466244* | *AX-109042952* | 0.53 | 2.40 | 0.12 | 0.43 | -0.03 | -0.01 | 0.00 | 0.01 | 0.00 | 0.03 |  |
|  | 5A | 110 | *AX-108848765* | *AX-111673092* | 1.18 | 2.65 | 0.26 | 0.49 | -0.02 | -0.01 | 0.00 | -0.01 | 0.00 | 0.04 |  |
|  | 5A | 4 | *AX-109537606* | *AX-108892891* | 1.13 | 2.99 | 0.23 | 0.88 | 0.00 | 0.02 | 0.00 | -0.04 | -0.01 | 0.04 |  |
|  | 5A | 19 | *AX-109328544* | *AX-111096820* | 2.97 | 1.91 | 0.64 | 0.42 | -0.01 | 0.01 | -0.01 | -0.03 | 0.01 | 0.02 |  |
|  | 5A | 38 | *AX-110366474* | *AX-108732747* | 2.33 | 1.34 | 0.48 | 0.14 | 0.00 | 0.00 | -0.01 | -0.01 | 0.00 | 0.02 |  |
|  | 5B | 61 | *AX-108938195* | *AX-108790507* | 1.47 | 1.81 | 0.32 | 0.59 | 0.01 | 0.00 | 0.02 | -0.04 | -0.01 | 0.01 |  |
|  | 5B | 101 | *AX-109648386* | *AX-111627154* | 0.04 | 2.59 | 0.01 | 0.43 | 0.01 | 0.01 | -0.03 | -0.01 | 0.00 | 0.02 |  |
|  | 5B | 0 | *AX-109584999* | *AX-108857064* | 1.91 | 1.35 | 0.42 | 0.20 | 0.00 | -0.01 | 0.01 | -0.01 | -0.01 | 0.02 |  |
|  | 5B | 6 | *AX-110094230* | *AX-111001298* | 1.53 | 1.61 | 0.31 | 0.26 | 0.00 | -0.01 | 0.01 | 0.00 | -0.01 | 0.02 |  |
|  | 5B | 26 | *AX-89718033* | *AX-110937447* | 1.37 | 1.35 | 0.30 | 0.20 | 0.01 | -0.02 | 0.01 | 0.01 | -0.02 | 0.01 |  |
|  | 5B | 31 | *AX-110016191* | *AX-110553427* | 2.32 | 1.29 | 0.50 | 0.12 | 0.00 | -0.01 | 0.01 | 0.00 | -0.01 | 0.01 |  |
|  | 5B | 35 | *AX-109313355* | *AX-109031326* | 2.73 | 1.49 | 0.60 | 0.13 | 0.00 | -0.01 | 0.01 | 0.01 | -0.01 | 0.01 |  |
|  | 5D | 33 | *AX-89489968* | *AX-89633041* | 3.03 | 1.40 | 0.65 | 0.33 | 0.00 | 0.02 | 0.00 | -0.03 | 0.00 | 0.01 |  |
|  | 5D | 151 | *AX-108971172* | *AX-94616983* | 2.00 | 0.76 | 0.43 | 0.19 | -0.02 | 0.00 | 0.00 | 0.02 | 0.00 | 0.00 |  |
|  | 5D | 6 | *AX-111460807* | *AX-110625313* | 2.24 | 0.70 | 0.50 | 0.10 | 0.00 | -0.01 | 0.01 | 0.00 | 0.00 | 0.01 |  |
|  | 5D | 113 | *AX-109351133* | *AX-110974080* | 2.83 | 1.56 | 0.63 | 0.08 | 0.00 | 0.01 | 0.00 | -0.01 | 0.01 | -0.01 |  |
|  | 5D | 129 | *AX-110709907* | *AX-109311748* | 2.14 | 1.07 | 0.47 | 0.07 | 0.01 | 0.01 | 0.00 | 0.00 | 0.00 | -0.01 |  |
|  | 5D | 146 | *AX-108881807* | *AX-109575293* | 2.04 | 1.05 | 0.45 | 0.08 | 0.00 | 0.00 | 0.00 | 0.00 | 0.01 | -0.01 |  |
|  | 6A | 15 | *AX-111358884* | *AX-109300733* | 0.95 | 2.22 | 0.21 | 0.38 | -0.02 | -0.01 | 0.01 | 0.02 | -0.02 | 0.01 |  |
|  | 6A | 76 | *AX-111724505* | *AX-110428755* | 1.07 | 1.64 | 0.24 | 0.28 | 0.00 | -0.01 | 0.01 | 0.02 | -0.01 | -0.02 |  |
|  | 6A | 149 | *AX-108876696* | *AX-109817601* | 0.97 | 1.73 | 0.21 | 0.17 | 0.01 | 0.01 | -0.02 | 0.01 | -0.01 | 0.00 |  |
|  | 6B | 135 | *AX-110427037* | *AX-109513034* | 2.20 | 1.11 | 0.48 | 0.18 | 0.00 | 0.01 | 0.02 | -0.01 | -0.01 | -0.01 |  |
|  | 6B | 15 | *AX-110565716* | *AX-111142565* | 2.89 | 2.28 | 0.63 | 0.27 | -0.01 | -0.01 | 0.00 | 0.01 | -0.01 | 0.02 |  |
|  | 6B | 35 | *AX-111112860* | *AX-111756688* | 6.21 | 2.44 | 1.36 | 0.27 | -0.01 | 0.00 | 0.00 | -0.01 | -0.02 | 0.02 |  |
|  | 6B | 85 | *AX-110515622* | *AX-110610753* | 2.95 | 1.42 | 0.64 | 0.20 | -0.01 | 0.00 | 0.00 | -0.01 | -0.02 | 0.02 |  |
|  | 6D | 65 | *AX-94618881* | *AX-110469783* | 27.96 | 8.76 | 6.76 | 1.65 | -0.01 | -0.04 | -0.01 | 0.02 | 0.06 | 0.00 | ***QKW.sicau-2SY-6D*** |
|  | 7A | 26 | *AX-109431738* | *AX-110481731* | 3.15 | 2.30 | 0.69 | 0.13 | 0.01 | -0.02 | -0.01 | 0.01 | 0.00 | 0.01 |  |
|  | 7A | 38 | *AX-111504531* | *AX-111059391* | 1.71 | 2.02 | 0.37 | 0.16 | 0.00 | -0.02 | -0.01 | 0.01 | 0.00 | 0.01 |  |
|  | 7A | 52 | *AX-108830715* | *AX-111488311* | 2.57 | 2.28 | 0.56 | 0.12 | 0.00 | -0.01 | -0.01 | 0.01 | -0.01 | 0.01 |  |
|  | 7A | 91 | *AX-110041044* | *AX-108727131* | 2.41 | 1.63 | 0.53 | 0.10 | 0.00 | -0.01 | 0.00 | 0.01 | -0.01 | 0.00 |  |
|  | 7A | 109 | *AX-109426816* | *AX-111120480* | 3.13 | 1.91 | 0.61 | 0.13 | 0.00 | -0.01 | 0.01 | 0.01 | -0.01 | 0.00 |  |
|  | 7A | 0 | *AX-111697134* | *AX-111129729* | 2.02 | 1.25 | 0.44 | 0.20 | -0.02 | 0.00 | 0.00 | 0.01 | -0.01 | 0.02 |  |
|  | 7A | 9 | *AX-109860028* | *AX-109348317* | 1.83 | 0.92 | 0.40 | 0.13 | -0.01 | -0.01 | 0.00 | 0.01 | 0.00 | 0.01 |  |
|  | 7A | 28 | *AX-109417084* | *AX-108776518* | 2.17 | 1.51 | 0.48 | 0.18 | 0.00 | 0.00 | 0.00 | 0.01 | -0.02 | 0.01 |  |
|  | 7A | 49 | *AX-108762348* | *AX-108908964* | 2.13 | 0.72 | 0.47 | 0.10 | 0.01 | 0.00 | -0.01 | 0.01 | 0.00 | 0.00 |  |
|  | 7A | 55 | *AX-111025190* | *AX-108760303* | 1.77 | 0.77 | 0.39 | 0.05 | 0.00 | 0.01 | -0.01 | 0.00 | 0.00 | 0.00 |  |
|  | 7A | 59 | *AX-111052953* | *AX-110068002* | 2.55 | 0.97 | 0.56 | 0.06 | 0.00 | 0.01 | -0.01 | 0.00 | 0.00 | 0.00 |  |
|  | 7A | 120 | *AX-109357068* | *AX-109285978* | 0.28 | 2.27 | 0.06 | 0.47 | 0.03 | 0.01 | -0.01 | 0.01 | 0.00 | -0.03 |  |
|  | 7A | 181 | *AX-110001543* | *AX-110410864* | 1.78 | 1.89 | 0.39 | 0.26 | -0.01 | 0.01 | 0.01 | 0.02 | -0.01 | -0.02 |  |
|  | 7B | 86 | *AX-109881255* | *AX-89365306* | 1.87 | 2.66 | 0.39 | 0.50 | 0.00 | 0.00 | -0.02 | -0.02 | 0.02 | 0.02 |  |
|  | 7B | 149 | *AX-109483160* | *AX-109851212* | 2.23 | 2.42 | 0.51 | 0.22 | -0.02 | 0.00 | 0.01 | -0.01 | 0.01 | 0.01 |  |
|  | 7B | 17 | *AX-109433397* | *AX-108978455* | 0.66 | 1.88 | 0.14 | 0.36 | 0.01 | -0.01 | -0.03 | 0.01 | 0.01 | 0.01 |  |
|  | 7B | 38 | *AX-89556194* | *AX-110493377* | 3.49 | 1.97 | 0.76 | 0.23 | 0.01 | 0.01 | -0.02 | 0.00 | 0.02 | 0.00 |  |
|  | 7B | 18 | *AX-108778081* | *AX-109887886* | 3.13 | 1.81 | 0.65 | 0.29 | -0.01 | 0.00 | -0.02 | 0.01 | 0.00 | 0.02 |  |
|  | 7B | 25 | *AX-109926256* | *AX-89540697* | 3.63 | 1.64 | 0.79 | 0.18 | -0.01 | 0.00 | -0.01 | 0.01 | 0.00 | 0.02 |  |
|  | 7D | 53 | *AX-108814055* | *AX-108956953* | 0.00 | 2.87 | 0.00 | 0.67 | 0.00 | 0.03 | -0.02 | -0.03 | 0.01 | 0.02 |  |
|  | 7D | 68 | *AX-111856876* | *AX-110240876* | 0.43 | 2.23 | 0.09 | 0.44 | -0.01 | 0.03 | -0.01 | -0.02 | 0.01 | 0.01 |  |
| **KT** | 1A | 33 | *AX-111015470* | *AX-111464225* | 1.95 | 1.59 | 0.84 | 0.51 | 0.01 | 0.01 | 0.00 | 0.01 | 0.00 | -0.02 |  |
|  | 1A | 41 | *AX-110998966* | *AX-109006135* | 1.98 | 1.32 | 0.85 | 0.38 | 0.01 | 0.01 | 0.00 | 0.01 | 0.00 | -0.02 |  |
|  | 1B | 101 | *AX-109509730* | *AX-109273979* | 0.40 | 4.00 | 0.17 | 1.84 | 0.01 | -0.02 | -0.01 | -0.02 | -0.01 | 0.04 | *QKT.sicau-2SY-1B* |
|  | 1B | 139 | *AX-111221126* | *AX-109359622* | 1.67 | 1.00 | 0.73 | 1.05 | 0.00 | 0.00 | 0.02 | -0.03 | 0.01 | -0.01 |  |
|  | 1B | 144 | *AX-109490843* | *AX-110065453* | 2.10 | 0.71 | 0.89 | 0.85 | 0.00 | 0.01 | 0.01 | -0.03 | 0.01 | -0.01 |  |
|  | 1B | 170 | *AX-111122733* | *AX-111183074* | 2.81 | 1.15 | 1.19 | 0.48 | 0.01 | 0.00 | -0.01 | -0.02 | 0.01 | 0.00 |  |
|  | 1B | 201 | *AX-110666954* | *AX-110415999* | 2.07 | 1.80 | 0.88 | 0.29 | 0.00 | -0.01 | -0.01 | 0.00 | 0.00 | 0.02 |  |
|  | 1B | 23 | *AX-109376365* | *AX-109299717* | 1.84 | 3.24 | 0.78 | 0.81 | 0.02 | 0.00 | 0.01 | 0.00 | -0.01 | -0.02 |  |
|  | 1B | 33 | *AX-89506553* | *AX-110384584* | 2.10 | 2.22 | 0.89 | 0.45 | 0.01 | 0.00 | 0.01 | 0.01 | -0.02 | -0.01 |  |
|  | 1B | 53 | *AX-109924351* | *AX-111454486* | 4.11 | 2.46 | 1.75 | 1.04 | -0.01 | -0.01 | 0.00 | 0.02 | -0.02 | 0.02 |  |
|  | 1D | 69 | *AX-110556510* | *AX-109349990* | 0.29 | 2.43 | 0.12 | 0.85 | 0.01 | 0.01 | 0.00 | -0.02 | -0.02 | 0.02 |  |
|  | 2A | 45 | *AX-109074831* | *AX-86179766* | 2.01 | 0.78 | 0.79 | 0.17 | 0.00 | 0.01 | 0.00 | -0.01 | 0.00 | 0.00 |  |
|  | 2A | 57 | *AX-111563490* | *AX-110943358* | 1.50 | 1.61 | 0.64 | 0.69 | 0.01 | 0.02 | -0.02 | -0.02 | 0.00 | 0.01 |  |
|  | 2A | 80 | *AX-108918456* | *AX-108769936* | 0.79 | 3.88 | 0.34 | 1.18 | -0.01 | 0.03 | 0.01 | -0.01 | -0.02 | 0.00 |  |
|  | 2A | 86 | *AX-108917836* | *AX-109950638* | 0.28 | 3.39 | 0.11 | 1.03 | -0.01 | 0.03 | 0.01 | -0.01 | -0.01 | 0.00 |  |
|  | 2A | 94 | *AX-110990867* | *AX-109311920* | 1.08 | 2.29 | 0.38 | 0.87 | -0.02 | 0.01 | 0.01 | -0.02 | 0.00 | 0.01 |  |
|  | 2A | 102 | *AX-109417714* | *AX-110949248* | 1.70 | 2.33 | 0.66 | 0.69 | -0.01 | 0.02 | 0.01 | -0.01 | 0.00 | 0.00 |  |
|  | 2B | 34 | *AX-109835884* | *AX-110406750* | 2.41 | 3.13 | 1.04 | 0.50 | 0.01 | 0.01 | -0.01 | 0.00 | -0.02 | 0.00 | *QKT.sicau-2SY-2B.1* |
|  | 2B | 44 | *AX-111500033* | *AX-111710268* | 2.24 | 1.52 | 0.96 | 0.38 | -0.01 | 0.01 | 0.00 | -0.02 | 0.01 | 0.01 |  |
|  | 2B | 130 | *AX-109915619* | *AX-110934424* | 2.87 | 3.52 | 1.19 | 1.11 | 0.00 | 0.00 | 0.00 | 0.02 | 0.01 | -0.03 | *QKT.sicau-2SY-2B.2* |
|  | 2B | 147 | *AX-94441014* | *AX-109521609* | 0.46 | 2.52 | 0.20 | 0.71 | -0.02 | 0.00 | -0.01 | 0.01 | 0.01 | 0.02 |  |
|  | 2B | 160 | *AX-109447372* | *AX-108826010* | 0.28 | 5.35 | 0.12 | 1.35 | -0.01 | 0.01 | 0.01 | 0.01 | -0.03 | 0.02 | *QKT.sicau-2SY-2B.3* |
|  | 2B | 198 | *AX-110437295* | *AX-94452908* | 0.51 | 2.34 | 0.22 | 0.90 | -0.01 | 0.02 | 0.02 | -0.02 | -0.01 | 0.00 |  |
|  | 2D | 9 | *AX-110929471* | *AX-109847853* | 34.03 | 16.59 | 16.64 | 7.78 | -0.01 | -0.04 | 0.03 | 0.05 | -0.06 | 0.04 | ***QKT.sicau-2SY-2D*** |
|  | 2D | 55 | *AX-109291628* | *AX-111093303* | 3.33 | 1.64 | 1.34 | 0.81 | -0.01 | 0.00 | 0.01 | 0.02 | -0.01 | -0.02 |  |
|  | 3A | 56 | *AX-111074009* | *AX-111122043* | 0.84 | 2.04 | 0.36 | 0.83 | 0.01 | 0.02 | 0.00 | 0.00 | -0.01 | -0.02 |  |
|  | 3A | 7 | *AX-110363308* | *AX-108853839* | 0.84 | 1.87 | 0.31 | 0.65 | 0.00 | -0.01 | 0.01 | -0.01 | -0.01 | 0.02 |  |
|  | 3B | 1 | *AX-109868743* | *AX-108758910* | 0.11 | 3.10 | 0.04 | 1.49 | 0.02 | -0.03 | 0.01 | -0.02 | 0.01 | 0.00 |  |
|  | 3B | 45 | *AX-110599784* | *AX-111135059* | 2.01 | 0.80 | 0.86 | 0.42 | 0.00 | -0.01 | 0.00 | -0.01 | 0.02 | 0.00 |  |
|  | 3D | 3 | *AX-109499958* | *AX-108907550* | 1.30 | 1.70 | 0.55 | 0.63 | 0.00 | 0.01 | 0.02 | -0.02 | 0.01 | -0.01 |  |
|  | 4A | 28 | *AX-109426161* | *AX-110626940* | 0.97 | 1.80 | 0.41 | 0.39 | 0.00 | 0.00 | 0.00 | 0.00 | -0.01 | 0.02 |  |
|  | 4A | 31 | *AX-111645255* | *AX-111543816* | 2.42 | 4.50 | 1.04 | 0.68 | -0.01 | -0.01 | 0.00 | 0.00 | 0.02 | 0.01 | *QKT.sicau-2SY-4A* |
|  | 4A | 150 | *AX-108760840* | *AX-110908322* | 0.09 | 2.66 | 0.04 | 1.33 | -0.02 | -0.02 | 0.01 | 0.03 | 0.01 | -0.01 |  |
|  | 4B | 38 | *AX-89414036* | *AX-110428871* | 1.78 | 2.25 | 0.76 | 0.58 | -0.02 | 0.00 | -0.01 | 0.00 | 0.00 | 0.02 |  |
|  | 4B | 5 | *AX-111233094* | *AX-109110130* | 0.00 | 3.52 | 0.00 | 1.31 | -0.02 | 0.02 | 0.02 | 0.00 | -0.02 | -0.01 |  |
|  | 4B | 63 | *AX-108797244* | *AX-110430517* | 2.06 | 1.64 | 0.88 | 0.46 | 0.00 | 0.00 | -0.02 | -0.01 | 0.00 | 0.02 |  |
|  | 4B | 75 | *AX-109287209* | *AX-111064477* | 2.91 | 1.45 | 1.25 | 0.34 | -0.01 | 0.00 | -0.01 | -0.01 | 0.01 | 0.01 |  |
|  | 4B | 55 | *AX-110467582* | *AX-109410422* | 0.96 | 1.76 | 0.41 | 0.58 | -0.01 | 0.01 | 0.01 | 0.01 | 0.00 | -0.02 |  |
|  | 5A | 6 | *AX-89349479* | *AX-109292423* | 0.81 | 2.00 | 0.35 | 0.83 | 0.02 | -0.02 | 0.00 | 0.01 | 0.01 | -0.02 |  |
|  | 5A | 17 | *AX-109512380* | *AX-111572088* | 0.23 | 2.41 | 0.10 | 0.63 | -0.01 | -0.01 | 0.00 | 0.01 | 0.02 | 0.00 | *QKT.sicau-2SY-5A* |
|  | 5B | 78 | *AX-95126382* | *AX-110524422* | 1.45 | 2.60 | 0.62 | 0.61 | 0.02 | -0.02 | 0.01 | -0.01 | 0.00 | 0.00 |  |
|  | 5D | 20 | *AX-89489968* | *AX-89633041* | 5.29 | 3.29 | 1.99 | 0.82 | -0.01 | 0.01 | -0.02 | -0.02 | 0.01 | 0.02 | *QKT.sicau-2SY-5D* |
|  | 5D | 44 | *AX-109431959* | *AX-109947024* | 1.79 | 1.47 | 0.77 | 0.65 | 0.01 | -0.02 | 0.01 | -0.01 | 0.00 | 0.01 |  |
|  | 5D | 43 | *AX-89451602* | *AX-110091088* | 1.61 | 1.29 | 0.69 | 0.31 | 0.00 | -0.01 | 0.01 | 0.01 | 0.00 | 0.00 |  |
|  | 29 | 16 | *AX-110470461* | *AX-109341845* | 1.82 | 0.78 | 0.77 | 0.23 | -0.01 | 0.01 | 0.00 | 0.00 | 0.00 | -0.01 |  |
|  | 6A | 34 | *AX-110423063* | *AX-109420246* | 0.57 | 3.01 | 0.25 | 1.05 | 0.00 | -0.01 | 0.03 | 0.00 | -0.01 | -0.01 | *QKT.sicau-2SY-6A* |
|  | 6B | 10 | *AX-89682312* | *AX-109499082* | 1.21 | 1.75 | 0.50 | 0.82 | -0.02 | 0.00 | -0.02 | 0.01 | 0.00 | 0.02 |  |
|  | 6B | 107 | *AX-110577201* | *AX-109469226* | 1.84 | 1.18 | 0.78 | 0.41 | 0.00 | -0.01 | 0.01 | 0.00 | 0.01 | -0.01 |  |
|  | 6B | 122 | *AX-109935603* | *AX-108954574* | 4.11 | 1.04 | 1.75 | 0.26 | 0.01 | -0.01 | 0.00 | -0.01 | 0.01 | 0.00 |  |
|  | 6B | 48 | *AX-111547303* | *AX-111472663* | 0.81 | 4.27 | 0.34 | 0.97 | -0.01 | 0.00 | 0.02 | 0.00 | -0.03 | 0.01 | *QKT.sicau-2SY-6B* |
|  | 6B | 82 | *AX-110515622* | *AX-110610753* | 1.06 | 1.58 | 0.46 | 0.42 | -0.02 | -0.01 | 0.00 | 0.01 | 0.00 | 0.01 |  |
|  | 6D | 62 | *AX-110667224* | *AX-94618881* | 3.29 | 2.13 | 1.35 | 0.15 | 0.01 | -0.01 | 0.01 | 0.00 | 0.00 | 0.00 |  |
|  | 6D | 97 | *AX-109158832* | *AX-108978672* | 3.02 | 1.42 | 1.28 | 0.39 | -0.01 | 0.01 | 0.01 | 0.01 | -0.01 | 0.00 |  |
|  | 7A | 58 | *AX-111052953* | *AX-110068002* | 1.09 | 2.17 | 0.46 | 0.71 | 0.01 | 0.02 | 0.01 | -0.01 | -0.01 | -0.01 |  |
|  | 7A | 67 | *AX-111034263* | *AX-109366562* | 0.74 | 2.59 | 0.32 | 1.00 | 0.01 | 0.03 | 0.00 | -0.01 | -0.01 | -0.01 |  |
|  | 7A | 73 | *AX-111610630* | *AX-111511322* | 0.29 | 3.06 | 0.12 | 1.24 | 0.01 | 0.03 | 0.01 | -0.01 | -0.01 | -0.02 |  |
|  | 7A | 115 | *AX-111567539* | *AX-109357068* | 0.08 | 2.47 | 0.04 | 0.94 | 0.02 | 0.00 | 0.00 | 0.00 | 0.00 | -0.03 |  |
|  | 7B | 52 | *AX-111638601* | *AX-111650139* | 0.97 | 2.04 | 0.42 | 0.67 | -0.01 | 0.01 | -0.01 | -0.01 | 0.01 | 0.01 |  |
|  | 7B | 12 | *AX-111765415* | *AX-94537587* | 0.48 | 4.41 | 0.21 | 1.05 | 0.00 | -0.01 | 0.00 | -0.02 | 0.03 | 0.00 | *QKT.sicau-2SY-7B* |
|  | 7D | 21 | *AX-109379249* | *AX-111250905* | 0.01 | 2.52 | 0.00 | 0.85 | -0.01 | 0.02 | 0.01 | 0.00 | -0.01 | -0.01 |  |
|  | 7D | 95 | *AX-111438923* | *AX-89384255* | 0.77 | 2.41 | 0.33 | 0.80 | 0.01 | 0.01 | 0.01 | -0.01 | 0.00 | -0.03 |  |
|  | 7D | 148 | *AX-110681402* | *AX-111542944* | 2.82 | 2.44 | 1.21 | 0.46 | 0.00 | 0.00 | -0.01 | 0.00 | 0.02 | -0.01 |  |
| **TKW** | 1A | 31 | *AX-108780484* | *AX-111637533* | 1.48 | 1.96 | 0.39 | 0.37 | 0.47 | 0.23 | 0.12 | 0.30 | -0.82 | -0.25 |  |
|  | 1A | 47 | *AX-110911618* | *AX-111158522* | 0.72 | 1.83 | 0.19 | 0.33 | 0.60 | -0.53 | 0.37 | 0.18 | -0.21 | -0.41 |  |
|  | 1B | 120 | *AX-109457059* | *AX-110501414* | 0.06 | 3.60 | 0.02 | 0.79 | -0.37 | -0.64 | -0.05 | 0.05 | 1.35 | -0.30 | *QTKW.sicau-2SY-1B.2* |
|  | 1B | 128 | *AX-89635557* | *AX-109478219* | 3.11 | 0.87 | 0.80 | 0.06 | 0.13 | -0.24 | -0.16 | 0.12 | 0.21 | -0.15 |  |
|  | 1B | 138 | *AX-109334796* | *AX-111221126* | 2.40 | 0.64 | 0.61 | 0.06 | 0.18 | -0.38 | 0.02 | -0.05 | 0.09 | 0.10 |  |
|  | 1B | 146 | *AX-110065453* | *AX-94433089* | 3.17 | 1.79 | 0.83 | 0.07 | 0.17 | 0.16 | 0.11 | -0.36 | -0.09 | -0.03 |  |
|  | 1B | 160 | *AX-111042449* | *AX-111688280* | 1.73 | 2.24 | 0.42 | 0.37 | -0.86 | 0.54 | 0.15 | -0.19 | 0.06 | 0.15 | *QTKW.sicau-2SY-1B.3* |
|  | 1B | 201 | *AX-110666954* | *AX-110415999* | 2.70 | 0.93 | 0.70 | 0.02 | -0.04 | 0.19 | 0.06 | -0.04 | -0.08 | -0.06 |  |
|  | 1B | 27 | *AX-108839316* | *AX-108930953* | 2.69 | 2.10 | 0.70 | 0.17 | 0.46 | 0.11 | 0.10 | -0.02 | -0.41 | -0.33 | *QTKW.sicau-2SY-1B.1* |
|  | 1B | 35 | *AX-110938295* | *AX-108765712* | 1.63 | 1.58 | 0.43 | 0.16 | 0.23 | 0.25 | 0.12 | 0.01 | -0.22 | -0.54 |  |
|  | 1B | 40 | *AX-109618461* | *AX-110000934* | 2.00 | 1.63 | 0.52 | 0.19 | 0.22 | 0.38 | 0.06 | 0.05 | -0.38 | -0.46 |  |
|  | 1B | 53 | *AX-109924351* | *AX-111454486* | 2.09 | 3.13 | 0.55 | 0.40 | -0.03 | 0.12 | -0.15 | -0.28 | 0.89 | -0.56 |  |
|  | 1D | 8 | *AX-109304203* | *AX-108764560* | 1.95 | 3.42 | 0.51 | 0.64 | 0.20 | 0.27 | -0.34 | 0.92 | -0.20 | -0.90 | *QTKW.sicau-2SY-1D* |
|  | 1D | 39 | *AX-110806579* | *AX-108863674* | 0.44 | 2.25 | 0.12 | 0.41 | 0.40 | -0.04 | 0.23 | -0.10 | 0.30 | -0.94 |  |
|  | 2A | 80 | *AX-108918456* | *AX-108769936* | 2.70 | 1.61 | 0.70 | 0.18 | 0.41 | -0.22 | 0.34 | -0.45 | -0.04 | -0.08 |  |
|  | 2A | 85 | *AX-111014053* | *AX-108917836* | 2.74 | 1.39 | 0.70 | 0.18 | 0.35 | -0.30 | 0.36 | -0.43 | 0.01 | -0.05 |  |
|  | 2B | 0 | *AX-110936280* | *AX-109918460* | 2.28 | 0.95 | 0.60 | 0.04 | -0.01 | -0.21 | 0.11 | -0.12 | 0.03 | 0.21 |  |
|  | 2B | 22 | *AX-108766267* | *AX-109912457* | 2.47 | 1.30 | 0.63 | 0.81 | 0.08 | -1.39 | 0.05 | 0.38 | 0.47 | 0.37 |  |
|  | 2B | 130 | *AX-109915619* | *AX-110934424* | 1.24 | 3.00 | 0.32 | 0.53 | 0.16 | 0.98 | -0.38 | -0.12 | -0.67 | -0.10 |  |
|  | 2B | 190 | *AX-110462784* | *AX-109817336* | 1.79 | 0.83 | 0.47 | 0.17 | -0.41 | 0.48 | 0.14 | 0.11 | -0.27 | -0.04 |  |
|  | 2D | 31 | *AX-109906393* | *AX-109402447* | 1.09 | 1.89 | 0.28 | 0.33 | 0.46 | -0.20 | -0.40 | 0.40 | -0.57 | 0.31 |  |
|  | 2D | 49 | *AX-109417243* | *AX-109294613* | 2.50 | 1.24 | 0.65 | 0.33 | 0.27 | 0.48 | -0.62 | -0.17 | -0.31 | 0.45 |  |
|  | 2D | 5 | *AX-110411457* | *AX-110899429* | 31.32 | 10.64 | 9.23 | 0.37 | 0.22 | -0.35 | -0.29 | -0.30 | 0.42 | 0.67 | ***QTKW.sicau-2SY-2D*** |
|  | 2D | 25 | *AX-108767381* | *AX-111722527* | 0.93 | 1.92 | 0.23 | 1.09 | 0.27 | 1.51 | -0.69 | -0.61 | -0.17 | -0.03 |  |
|  | 3A | 58 | *AX-111074009* | *AX-111122043* | 1.24 | 1.36 | 0.32 | 0.13 | -0.13 | -0.38 | 0.04 | 0.42 | 0.16 | -0.12 |  |
|  | 3A | 106 | *AX-109443040* | *AX-111087163* | 1.66 | 1.15 | 0.43 | 0.22 | 0.37 | 0.25 | -0.44 | 0.39 | -0.33 | -0.15 |  |
|  | 3B | 0 | *AX-111523002* | *AX-109868743* | 0.37 | 2.54 | 0.10 | 0.63 | 0.43 | -0.85 | -0.64 | 0.52 | -0.16 | 0.62 |  |
|  | 3B | 45 | *AX-110599784* | *AX-111135059* | 1.21 | 2.41 | 0.32 | 0.33 | 0.46 | 0.10 | -0.65 | 0.05 | -0.41 | 0.45 |  |
|  | 3B | 67 | *AX-108757678* | *AX-111007091* | 1.70 | 1.57 | 0.39 | 0.19 | 0.24 | 0.20 | 0.03 | 0.34 | -0.43 | -0.40 |  |
|  | 3B | 46 | *AX-110993960* | *AX-108783900* | 2.18 | 0.92 | 0.55 | 0.47 | -0.07 | 1.07 | -0.34 | -0.09 | -0.33 | -0.24 |  |
|  | 3B | 110 | *AX-110453138* | *AX-111474611* | 0.92 | 2.47 | 0.24 | 0.55 | -0.02 | 1.16 | -0.34 | -0.16 | -0.22 | -0.34 |  |
|  | 3D | 57 | *AX-89337262* | *AX-110042483* | 2.82 | 0.65 | 0.66 | 0.37 | -0.11 | 0.91 | -0.28 | -0.39 | -0.15 | 0.12 |  |
|  | 3D | 0 | *AX-109271722* | *AX-110941549* | 3.95 | 2.04 | 1.03 | 0.13 | -0.01 | 0.14 | 0.43 | 0.08 | -0.34 | -0.28 |  |
|  | 3D | 23 | *AX-108787732* | *AX-108894516* | 1.19 | 3.19 | 0.31 | 0.65 | -0.22 | -1.19 | 0.49 | 0.47 | 0.11 | 0.25 |  |
|  | 4A | 150 | *AX-108760840* | *AX-110908322* | 3.12 | 1.07 | 0.81 | 0.26 | 0.11 | 0.63 | -0.35 | -0.19 | -0.42 | 0.24 |  |
|  | 4B | 39 | *AX-89414036* | *AX-110428871* | 1.05 | 2.27 | 0.25 | 0.35 | 0.28 | 0.20 | -0.28 | -0.78 | 0.53 | 0.04 |  |
|  | 4B | 31 | *AX-110574019* | *AX-109330857* | 2.30 | 2.77 | 0.60 | 0.61 | -0.10 | -0.27 | 0.28 | -1.07 | 0.32 | 0.69 | *QTKW.sicau-2SY-4B* |
|  | 4B | 57 | *AX-108769486* | *AX-111080767* | 3.31 | 2.62 | 0.84 | 0.21 | -0.51 | 0.30 | -0.41 | 0.13 | 0.18 | 0.28 |  |
|  | 4B | 76 | *AX-109287209* | *AX-111064477* | 4.20 | 1.74 | 1.10 | 0.14 | -0.18 | -0.18 | -0.27 | 0.01 | 0.52 | 0.03 |  |
|  | 4D | 2 | *AX-109876857* | *AX-110937266* | 2.99 | 2.84 | 0.78 | 0.30 | 0.34 | -0.60 | -0.26 | 0.56 | 0.16 | -0.13 |  |
|  | 4D | 64 | *AX-110572006* | *AX-109230716* | 1.23 | 2.05 | 0.32 | 0.23 | 0.06 | -0.02 | 0.08 | -0.43 | 0.63 | -0.32 | *QTKW.sicau-2SY-4D* |
|  | 4D | 108 | *AX-110466464* | *AX-110479846* | 2.92 | 0.73 | 0.74 | 0.13 | 0.17 | 0.40 | -0.18 | -0.22 | -0.28 | 0.21 |  |
|  | 4D | 131 | *AX-108934138* | *AX-111470564* | 0.77 | 2.22 | 0.20 | 0.55 | 0.27 | -0.59 | 0.14 | -0.59 | 0.93 | -0.18 |  |
|  | 5A | 3 | *AX-111070969* | *AX-109859109* | 4.14 | 1.44 | 1.08 | 0.07 | 0.04 | -0.15 | -0.02 | 0.33 | -0.17 | -0.15 |  |
|  | 5A | 14 | *AX-108948329* | *AX-94441001* | 1.98 | 1.19 | 0.52 | 0.12 | -0.02 | -0.08 | -0.40 | 0.38 | -0.12 | 0.15 |  |
|  | 5A | 48 | *AX-109819799* | *AX-109440590* | 0.16 | 2.96 | 0.04 | 0.78 | -0.05 | -0.80 | -0.18 | 1.23 | -0.42 | 0.13 |  |
|  | 5A | 66 | *AX-108802032* | *AX-94985037* | 1.62 | 1.00 | 0.42 | 0.64 | 0.40 | -1.16 | -0.12 | 0.63 | 0.13 | -0.03 |  |
|  | 5A | 125 | *AX-110739984* | *AX-109622137* | 1.64 | 1.30 | 0.42 | 0.25 | 0.50 | -0.39 | -0.42 | -0.12 | -0.06 | 0.40 |  |
|  | 5A | 131 | *AX-108790581* | *AX-111129970* | 1.23 | 1.48 | 0.29 | 0.35 | 0.40 | 0.61 | -0.19 | 0.12 | -0.51 | -0.45 |  |
|  | 5A | 144 | *AX-110076201* | *AX-109919444* | 0.36 | 2.23 | 0.08 | 0.53 | 0.56 | -0.09 | 0.18 | 0.40 | -0.08 | -1.06 |  |
|  | 5A | 2 | *AX-110430512* | *AX-108923855* | 0.25 | 2.74 | 0.06 | 0.81 | -0.24 | -1.28 | 0.63 | 0.07 | 0.50 | 0.33 |  |
|  | 5A | 40 | *AX-110366474* | *AX-108732747* | 2.16 | 0.57 | 0.55 | 0.32 | 0.04 | -0.85 | 0.03 | 0.12 | 0.46 | 0.11 |  |
|  | 5B | 4 | *AX-110675968* | *AX-110384815* | 1.52 | 1.10 | 0.39 | 0.18 | 0.03 | 0.02 | -0.03 | -0.38 | 0.54 | -0.31 |  |
|  | 5B | 69 | *AX-110974598* | *AX-110447570* | 0.08 | 3.01 | 0.02 | 0.89 | -0.20 | -1.07 | 1.16 | 0.25 | 0.23 | -0.37 |  |
|  | 5B | 0 | *AX-109584999* | *AX-108857064* | 2.27 | 0.63 | 0.59 | 0.16 | 0.03 | -0.58 | -0.08 | 0.14 | 0.33 | 0.13 |  |
|  | 5B | 31 | *AX-110016191* | *AX-110553427* | 1.60 | 0.92 | 0.41 | 0.08 | -0.01 | -0.11 | -0.15 | 0.43 | -0.18 | -0.02 |  |
|  | 5B | 34 | *AX-108742618* | *AX-109313355* | 2.14 | 1.22 | 0.56 | 0.12 | -0.10 | -0.16 | -0.11 | 0.52 | -0.18 | -0.01 |  |
|  | 5D | 33 | *AX-89489968* | *AX-89633041* | 3.56 | 1.40 | 0.92 | 0.49 | 0.10 | -1.01 | 0.27 | -0.23 | 0.44 | 0.41 |  |
|  | 5D | 41 | *AX-108881619* | *AX-109356068* | 0.66 | 2.03 | 0.17 | 0.52 | 0.52 | 0.54 | -0.71 | 0.04 | 0.30 | -0.64 |  |
|  | 5D | 73 | *AX-108822269* | *AX-111388087* | 2.13 | 1.51 | 0.55 | 0.16 | 0.37 | 0.04 | -0.57 | -0.06 | 0.12 | -0.01 |  |
|  | 6A | 3 | *AX-110671478* | *AX-109525056* | 1.33 | 2.65 | 0.35 | 0.51 | -0.32 | -0.15 | -0.41 | 0.04 | -0.29 | 1.08 |  |
|  | 6A | 14 | *AX-111089021* | *AX-111113291* | 2.87 | 1.33 | 0.75 | 0.15 | -0.46 | -0.22 | -0.05 | 0.35 | 0.11 | 0.19 |  |
|  | 6A | 95 | *AX-111800524* | *AX-109041968* | 2.20 | 1.01 | 0.50 | 0.03 | -0.07 | 0.19 | -0.22 | 0.01 | -0.02 | -0.01 |  |
|  | 6A | 168 | *AX-111549411* | *AX-109552957* | 1.87 | 0.99 | 0.47 | 0.11 | -0.07 | 0.10 | -0.07 | -0.33 | -0.07 | 0.45 |  |
|  | 6B | 83 | *AX-89344223* | *AX-110472291* | 1.51 | 1.09 | 0.39 | 0.14 | 0.51 | 0.00 | -0.36 | -0.05 | -0.01 | -0.17 |  |
|  | 6B | 108 | *AX-110580782* | *AX-94831360* | 3.90 | 1.84 | 1.02 | 0.18 | 0.42 | -0.46 | -0.25 | 0.01 | -0.05 | 0.31 |  |
|  | 6B | 113 | *AX-110404003* | *AX-110386321* | 3.77 | 2.31 | 0.99 | 0.09 | 0.34 | 0.15 | -0.22 | -0.29 | 0.07 | -0.04 |  |
|  | 6B | 122 | *AX-109935603* | *AX-108954574* | 4.04 | 2.40 | 1.06 | 0.09 | 0.26 | 0.20 | -0.29 | -0.11 | 0.17 | -0.21 |  |
|  | 6B | 132 | *AX-111621971* | *AX-110956323* | 2.34 | 2.03 | 0.62 | 0.17 | 0.17 | 0.53 | -0.30 | -0.26 | 0.08 | -0.22 |  |
|  | 6B | 35 | *AX-111112860* | *AX-111756688* | 2.19 | 1.02 | 0.57 | 0.56 | 0.54 | -1.02 | -0.05 | -0.08 | 0.63 | -0.19 |  |
|  | 6D | 65 | *AX-94618881* | *AX-110469783* | 4.74 | 3.81 | 1.23 | 0.44 | 0.89 | -0.44 | -0.42 | 0.34 | -0.06 | -0.23 | *QTKW.sicau-2SY-6D* |
|  | 6D | 97 | *AX-109158832* | *AX-108978672* | 2.58 | 2.04 | 0.68 | 0.16 | -0.48 | -0.12 | 0.14 | 0.40 | 0.24 | -0.12 |  |
|  | 6D | 171 | *AX-110378380* | *AX-111487792* | 1.54 | 1.20 | 0.40 | 0.57 | -0.21 | 1.12 | -0.56 | -0.36 | -0.17 | 0.13 |  |
|  | 7A | 18 | *AX-111576650* | *AX-108812645* | 2.45 | 1.97 | 0.64 | 0.30 | -0.25 | -0.43 | -0.44 | 0.50 | 0.39 | 0.28 |  |
|  | 7A | 34 | *AX-86172687* | *AX-108761706* | 2.47 | 1.72 | 0.65 | 0.24 | -0.15 | -0.15 | -0.57 | 0.54 | 0.24 | 0.00 |  |
|  | 7A | 55 | *AX-110915503* | *AX-109306246* | 1.34 | 1.34 | 0.35 | 0.21 | 0.09 | 0.15 | -0.42 | 0.48 | 0.06 | -0.43 |  |
|  | 7A | 68 | *AX-111030876* | *AX-109866369* | 0.88 | 2.16 | 0.23 | 0.41 | -0.11 | 0.24 | -0.20 | 0.84 | -0.35 | -0.54 |  |
|  | 7A | 28 | *AX-109417084* | *AX-108776518* | 1.88 | 1.31 | 0.50 | 0.08 | 0.27 | -0.01 | -0.09 | -0.28 | 0.24 | -0.15 |  |
|  | 7A | 59 | *AX-111052953* | *AX-110068002* | 1.94 | 0.97 | 0.50 | 0.21 | -0.11 | 0.51 | 0.33 | -0.27 | 0.06 | -0.42 |  |
|  | 7A | 64 | *AX-111149309* | *AX-111034263* | 1.49 | 1.11 | 0.40 | 0.15 | -0.19 | 0.25 | 0.42 | -0.16 | 0.14 | -0.38 |  |
|  | 7A | 96 | *AX-111730218* | *AX-108837168* | 1.91 | 2.99 | 0.50 | 0.39 | -0.05 | 0.47 | -0.04 | 0.44 | -0.90 | -0.01 | *QTKW.sicau-2SY-7A* |
|  | 7A | 102 | *AX-110518554* | *AX-110442528* | 1.59 | 1.42 | 0.41 | 0.20 | -0.35 | -0.23 | 0.07 | 0.47 | 0.37 | -0.23 |  |
|  | 7A | 155 | *AX-108823936* | *AX-108820421* | 2.97 | 1.88 | 0.77 | 0.51 | 0.30 | 0.87 | -0.42 | -0.08 | 0.06 | -0.71 |  |
|  | 7A | 163 | *AX-108947190* | *AX-110049453* | 2.00 | 1.58 | 0.52 | 0.71 | 0.20 | 1.21 | -0.55 | -0.23 | -0.11 | -0.53 |  |
|  | 7A | 184 | *AX-110410864* | *AX-108743179* | 1.79 | 1.23 | 0.45 | 0.36 | 0.30 | 0.75 | -0.56 | -0.32 | -0.11 | -0.08 |  |
|  | 7B | 87 | *AX-89365306* | *AX-109927500* | 1.18 | 3.26 | 0.31 | 0.77 | 0.27 | -1.20 | 0.29 | -0.33 | 0.24 | 0.74 |  |
|  | 7B | 148 | *AX-110390771* | *AX-110412930* | 3.32 | 2.72 | 0.86 | 0.34 | 0.45 | -0.38 | 0.07 | -0.55 | -0.14 | 0.61 |  |
|  | 7B | 36 | *AX-108869330* | *AX-89556194* | 1.46 | 1.19 | 0.38 | 0.17 | -0.11 | 0.07 | -0.19 | -0.44 | 0.36 | 0.39 |  |
|  | 7B | 14 | *AX-109955495* | *AX-108778081* | 2.60 | 1.12 | 0.68 | 0.09 | -0.17 | 0.30 | 0.06 | -0.03 | 0.23 | -0.32 |  |
|  | 7B | 27 | *AX-89540697* | *AX-108917117* | 3.24 | 1.42 | 0.83 | 0.16 | -0.45 | 0.39 | 0.26 | -0.23 | 0.15 | -0.04 |  |
|  | 7D | 2 | *AX-108917923* | *AX-111693608* | 2.52 | 0.66 | 0.57 | 0.20 | -0.29 | 0.59 | -0.38 | 0.05 | 0.04 | 0.12 |  |
|  | 7D | 95 | *AX-111438923* | *AX-89384255* | 1.52 | 3.01 | 0.40 | 0.58 | 0.65 | 0.25 | 0.40 | -0.10 | -1.07 | -0.09 |  |
|  | 7D | 104 | *AX-108777441* | *AX-111793251* | 0.38 | 2.53 | 0.10 | 0.47 | 0.52 | -0.06 | 0.41 | 0.37 | -0.79 | -0.47 |  |
| **LWR** | 1A | 0 | *AX-111707816* | *AX-109930672* | 3.94 | 1.13 | 0.99 | 0.46 | 0.01 | 0.01 | -0.02 | -0.01 | 0.01 | -0.01 |  |
|  | 1A | 12 | *AX-94425021* | *AX-110932359* | 5.49 | 1.08 | 1.37 | 0.25 | 0.00 | 0.00 | -0.02 | 0.00 | 0.01 | 0.00 |  |
|  | 1A | 28 | *AX-94991487* | *AX-110559766* | 5.85 | 1.14 | 1.45 | 0.21 | 0.00 | 0.00 | -0.02 | 0.00 | 0.01 | 0.00 |  |
|  | 1B | 23 | *AX-109919035* | *AX-109390820* | 0.37 | 2.16 | 0.10 | 0.41 | 0.00 | 0.01 | 0.00 | 0.01 | -0.01 | -0.02 |  |
|  | 1B | 51 | *AX-110996145* | *AX-108806701* | 0.55 | 2.30 | 0.12 | 0.40 | 0.01 | 0.01 | -0.02 | 0.01 | 0.00 | -0.01 |  |
|  | 1B | 109 | *AX-108785928* | *AX-108911911* | 1.42 | 2.13 | 0.36 | 0.33 | 0.00 | 0.01 | 0.01 | -0.01 | 0.00 | -0.01 |  |
|  | 1B | 146 | *AX-110065453* | *AX-94433089* | 5.05 | 0.77 | 1.26 | 0.30 | 0.01 | 0.00 | -0.02 | 0.01 | -0.01 | 0.00 |  |
|  | 1B | 164 | *AX-111042449* | *AX-111688280* | 8.74 | 3.32 | 2.15 | 0.30 | 0.01 | 0.01 | -0.02 | 0.00 | 0.00 | 0.00 |  |
|  | 1B | 181 | *AX-111044588* | *AX-110906777* | 1.67 | 1.91 | 0.42 | 0.28 | 0.00 | 0.00 | 0.00 | 0.01 | -0.01 | -0.01 |  |
|  | 1B | 191 | *AX-110502871* | *AX-110392069* | 3.50 | 1.19 | 0.84 | 0.19 | -0.01 | 0.00 | -0.01 | 0.01 | 0.00 | 0.00 |  |
|  | 1D | 58 | *AX-111515122* | *AX-109320713* | 2.25 | 0.63 | 0.57 | 0.21 | -0.01 | 0.00 | 0.02 | 0.00 | 0.00 | 0.00 | *QLWR.sicau-2SY-1D* |
|  | 2A | 60 | *AX-109990976* | *AX-110407291* | 2.52 | 0.59 | 0.62 | 0.15 | 0.01 | 0.00 | -0.01 | 0.00 | 0.00 | 0.00 |  |
|  | 2A | 79 | *AX-109842387* | *AX-108918456* | 3.83 | 2.03 | 0.95 | 0.35 | 0.01 | -0.01 | -0.02 | 0.01 | 0.00 | 0.00 |  |
|  | 2A | 86 | *AX-108917836* | *AX-109950638* | 2.96 | 1.59 | 0.69 | 0.17 | 0.01 | 0.00 | -0.01 | 0.01 | 0.00 | 0.00 |  |
|  | 2A | 102 | *AX-109417714* | *AX-110949248* | 2.94 | 1.98 | 0.65 | 0.18 | 0.01 | 0.00 | -0.01 | 0.01 | 0.00 | -0.01 |  |
|  | 2A | 107 | *AX-108865385* | *AX-108833449* | 3.26 | 1.70 | 0.73 | 0.24 | 0.01 | 0.00 | -0.01 | 0.01 | 0.00 | -0.01 |  |
|  | 2B | 12 | *AX-111148175* | *AX-111074854* | 1.26 | 1.28 | 0.32 | 0.28 | -0.01 | 0.00 | -0.01 | 0.02 | 0.00 | -0.01 |  |
|  | 2B | 76 | *AX-94493672* | *AX-111026116* | 1.64 | 2.03 | 0.41 | 0.42 | 0.01 | -0.01 | 0.01 | -0.02 | -0.01 | 0.00 |  |
|  | 2B | 85 | *AX-111513142* | *AX-111233617* | 1.49 | 2.10 | 0.38 | 0.51 | 0.01 | 0.00 | 0.02 | -0.02 | -0.01 | 0.01 |  |
|  | 2B | 91 | *AX-110640825* | *AX-109334428* | 1.22 | 1.71 | 0.31 | 0.37 | 0.00 | 0.00 | 0.01 | -0.01 | -0.01 | 0.01 |  |
|  | 2D | 9 | *AX-86163393* | *AX-109785183* | 0.63 | 2.44 | 0.16 | 0.35 | -0.02 | -0.01 | 0.02 | 0.00 | 0.00 | 0.00 |  |
|  | 2D | 11 | *AX-111526465* | *AX-110558888* | 2.35 | 0.50 | 0.58 | 0.33 | 0.00 | 0.00 | 0.02 | 0.00 | 0.00 | -0.01 |  |
|  | 2D | 49 | *AX-109417243* | *AX-109294613* | 2.00 | 0.93 | 0.51 | 0.12 | 0.00 | 0.00 | -0.01 | 0.01 | 0.00 | 0.01 |  |
|  | 2D | 25 | *AX-108767381* | *AX-111722527* | 5.44 | 4.89 | 1.39 | 0.81 | 0.00 | -0.03 | 0.02 | 0.00 | 0.00 | 0.02 | ***QLWR.sicau-2SY-2D*** |
|  | 3A | 72 | *AX-108940763* | *AX-111552673* | 6.02 | 2.96 | 1.52 | 0.12 | 0.00 | 0.00 | -0.01 | 0.00 | 0.01 | 0.01 | *QLWR.sicau-2SY-3A* |
|  | 3A | 93 | *AX-111759901* | *AX-108950927* | 3.88 | 1.69 | 0.98 | 0.11 | -0.01 | 0.00 | 0.00 | 0.00 | 0.01 | 0.01 |  |
|  | 3A | 107 | *AX-111087163* | *AX-108854601* | 1.91 | 1.29 | 0.49 | 0.13 | -0.01 | 0.00 | 0.00 | 0.00 | 0.01 | 0.01 |  |
|  | 3A | 121 | *AX-110479868* | *AX-109342110* | 1.97 | 1.11 | 0.50 | 0.13 | -0.01 | 0.00 | -0.01 | 0.01 | 0.01 | 0.00 |  |
|  | 3A | 0 | *AX-108780807* | *AX-108737664* | 2.14 | 1.18 | 0.55 | 0.16 | -0.01 | 0.00 | 0.00 | 0.01 | 0.00 | 0.01 |  |
|  | 3A | 17 | *AX-110363308* | *AX-108853839* | 3.03 | 1.79 | 0.77 | 0.14 | -0.01 | 0.01 | -0.01 | 0.00 | 0.01 | 0.00 |  |
|  | 3B | 1 | *AX-109868743* | *AX-108758910* | 1.97 | 0.59 | 0.49 | 0.26 | -0.01 | 0.00 | 0.01 | 0.01 | -0.01 | 0.00 |  |
|  | 3B | 83 | *AX-111218273* | *AX-110493720* | 2.78 | 1.73 | 0.70 | 0.14 | -0.01 | 0.00 | -0.01 | 0.01 | 0.01 | -0.01 |  |
|  | 3B | 93 | *AX-109391294* | *AX-110453138* | 2.65 | 1.47 | 0.65 | 0.09 | 0.00 | 0.00 | -0.01 | 0.00 | 0.01 | 0.00 |  |
|  | 3D | 2 | *AX-94989783* | *AX-109499958* | 0.85 | 1.79 | 0.22 | 0.36 | 0.00 | -0.01 | 0.00 | 0.01 | -0.02 | 0.01 |  |
|  | 3D | 50 | *AX-89576720* | *AX-111916664* | 1.57 | 1.70 | 0.40 | 0.19 | 0.00 | -0.01 | 0.00 | 0.00 | 0.00 | 0.01 |  |
|  | 3D | 66 | *AX-111045512* | *AX-109303898* | 1.59 | 1.77 | 0.40 | 0.22 | 0.00 | -0.01 | 0.01 | 0.00 | 0.01 | -0.01 |  |
|  | 3D | 83 | *AX-111807575* | *AX-110987465* | 1.73 | 1.04 | 0.39 | 0.11 | 0.01 | 0.00 | 0.01 | -0.01 | 0.00 | 0.00 |  |
|  | 4A | 26 | *AX-109867908* | *AX-110580622* | 2.14 | 2.40 | 0.54 | 0.41 | 0.00 | 0.00 | 0.02 | -0.01 | 0.00 | -0.02 |  |
|  | 4A | 64 | *AX-111634994* | *AX-108765799* | 2.35 | 2.08 | 0.60 | 0.25 | 0.00 | -0.01 | 0.01 | 0.00 | 0.00 | -0.01 |  |
|  | 4A | 149 | *AX-95140127* | *AX-108760840* | 2.27 | 1.42 | 0.57 | 0.32 | 0.00 | -0.01 | 0.02 | -0.01 | 0.00 | 0.01 |  |
|  | 4A | 170 | *AX-109926421* | *AX-109384787* | 3.89 | 1.67 | 0.98 | 0.35 | 0.00 | 0.00 | 0.02 | -0.01 | 0.00 | 0.00 |  |
|  | 4A | 175 | *AX-110481242* | *AX-109286213* | 2.62 | 1.72 | 0.67 | 0.37 | 0.00 | 0.00 | 0.02 | -0.02 | -0.01 | 0.01 |  |
|  | 4B | 52 | *AX-109637078* | *AX-109861624* | 1.98 | 2.41 | 0.50 | 0.42 | 0.01 | 0.00 | 0.02 | -0.01 | 0.00 | -0.02 |  |
|  | 4D | 0 | *AX-111651065* | *AX-109529178* | 0.52 | 2.05 | 0.13 | 0.58 | 0.00 | 0.00 | 0.03 | -0.01 | -0.01 | -0.01 |  |
|  | 4D | 81 | *AX-110320914* | *AX-109606880* | 0.00 | 2.97 | 0.00 | 0.65 | 0.01 | -0.02 | -0.01 | 0.02 | 0.02 | -0.01 |  |
|  | 4D | 163 | *AX-111684216* | *AX-89567801* | 1.10 | 1.67 | 0.27 | 0.44 | 0.00 | 0.02 | -0.01 | -0.01 | 0.01 | -0.01 |  |
|  | 5A | 47 | *AX-111519092* | *AX-109819799* | 1.22 | 2.01 | 0.33 | 0.11 | 0.00 | 0.01 | 0.00 | 0.00 | -0.01 | 0.00 |  |
|  | 5A | 57 | *AX-109362376* | *AX-108878364* | 0.89 | 2.77 | 0.23 | 0.41 | 0.01 | 0.01 | -0.01 | -0.02 | 0.00 | 0.01 |  |
|  | 5B | 0 | *AX-108791526* | *AX-111490382* | 3.09 | 0.62 | 0.78 | 0.25 | 0.00 | 0.00 | -0.02 | 0.01 | 0.00 | -0.01 |  |
|  | 5B | 75 | *AX-108867784* | *AX-109952651* | 2.80 | 0.57 | 0.66 | 0.13 | 0.00 | 0.01 | -0.01 | 0.00 | 0.01 | 0.00 |  |
|  | 5B | 99 | *AX-109648386* | *AX-111627154* | 1.00 | 2.26 | 0.23 | 0.42 | 0.00 | -0.01 | 0.02 | 0.00 | 0.00 | -0.02 |  |
|  | 5B | 36 | *AX-109031326* | *AX-108788214* | 1.45 | 1.18 | 0.36 | 0.24 | -0.01 | 0.01 | 0.01 | 0.00 | 0.00 | -0.02 |  |
|  | 5D | 8 | *AX-111031068* | *AX-89489968* | 1.79 | 1.44 | 0.43 | 0.21 | 0.01 | 0.00 | -0.01 | 0.01 | 0.00 | 0.00 |  |
|  | 5D | 94 | *AX-109006940* | *AX-110405892* | 1.30 | 1.31 | 0.33 | 0.17 | 0.01 | 0.00 | -0.01 | 0.01 | 0.00 | -0.01 |  |
|  | 5D | 151 | *AX-108971172* | *AX-94616983* | 2.11 | 1.12 | 0.54 | 0.32 | 0.01 | 0.01 | -0.01 | -0.01 | 0.01 | 0.00 |  |
|  | 5D | 25 | *AX-110673944* | *AX-109464956* | 1.41 | 1.40 | 0.35 | 0.26 | 0.00 | 0.00 | 0.01 | -0.01 | 0.01 | -0.01 |  |
|  | 5D | 115 | *AX-110824131* | *AX-109034925* | 3.62 | 2.29 | 0.89 | 0.16 | 0.00 | 0.00 | 0.01 | 0.00 | 0.00 | -0.01 |  |
|  | 5D | 152 | *AX-109131548* | *AX-108793902* | 3.54 | 1.27 | 0.87 | 0.26 | 0.00 | 0.01 | -0.01 | -0.01 | 0.00 | 0.00 |  |
|  | 6A | 72 | *AX-109536927* | *AX-110038163* | 3.30 | 1.22 | 0.81 | 0.08 | -0.01 | 0.00 | 0.00 | 0.00 | 0.01 | 0.01 |  |
|  | 6A | 162 | *AX-111063393* | *AX-109984471* | 4.03 | 1.63 | 1.02 | 0.31 | -0.01 | -0.01 | 0.02 | 0.00 | 0.00 | 0.01 |  |
|  | 6A | 174 | *AX-111683356* | *AX-109828791* | 3.06 | 0.99 | 0.77 | 0.24 | 0.00 | -0.01 | 0.02 | 0.00 | 0.00 | 0.00 |  |
|  | 6B | 2 | *AX-111041733* | *AX-89682312* | 2.13 | 2.22 | 0.51 | 0.26 | 0.00 | -0.01 | 0.01 | 0.00 | 0.01 | -0.01 |  |
|  | 6B | 67 | *AX-109861055* | *AX-109034125* | 1.84 | 0.95 | 0.47 | 0.15 | 0.00 | 0.00 | 0.00 | 0.01 | 0.00 | -0.01 |  |
|  | 6B | 104 | *AX-109883185* | *AX-109449004* | 2.19 | 0.63 | 0.56 | 0.17 | 0.00 | 0.00 | -0.01 | 0.00 | 0.00 | 0.01 |  |
|  | 6B | 128 | *AX-111672676* | *AX-108811307* | 2.20 | 1.00 | 0.56 | 0.23 | -0.01 | -0.01 | 0.01 | 0.01 | -0.01 | 0.01 |  |
|  | 6B | 137 | *AX-109389027* | *AX-111113941* | 2.03 | 1.02 | 0.51 | 0.30 | -0.01 | -0.01 | 0.01 | 0.01 | -0.01 | 0.01 |  |
|  | 6B | 47 | *AX-109461489* | *AX-111547303* | 0.03 | 3.35 | 0.01 | 0.72 | 0.02 | 0.00 | -0.01 | -0.02 | 0.02 | -0.01 | *QLWR.sicau-2SY-6B* |
|  | 6D | 66 | *AX-110469783* | *AX-110066157* | 9.74 | 7.61 | 2.55 | 1.03 | 0.01 | 0.00 | 0.01 | -0.01 | -0.03 | 0.02 | ***QLWR.sicau-2SY-6D*** |
|  | 6D | 171 | *AX-110378380* | *AX-111487792* | 4.44 | 2.47 | 1.12 | 0.44 | -0.02 | 0.00 | 0.02 | -0.01 | 0.01 | 0.01 |  |
|  | 7A | 19 | *AX-111203913* | *AX-109945192* | 2.28 | 2.59 | 0.55 | 0.40 | 0.01 | -0.01 | 0.02 | -0.01 | -0.01 | -0.01 |  |
|  | 7A | 23 | *AX-109598170* | *AX-109465295* | 2.15 | 2.25 | 0.50 | 0.30 | 0.01 | -0.01 | 0.02 | 0.00 | 0.00 | -0.01 |  |
|  | 7A | 27 | *AX-109417084* | *AX-108776518* | 2.57 | 2.73 | 0.59 | 0.35 | 0.00 | -0.01 | 0.02 | 0.00 | 0.00 | -0.01 |  |
|  | 7A | 37 | *AX-110677006* | *AX-109824443* | 1.43 | 1.90 | 0.35 | 0.30 | 0.00 | -0.01 | 0.02 | 0.00 | 0.00 | -0.01 |  |
|  | 7A | 46 | *AX-108746976* | *AX-110483438* | 1.86 | 2.03 | 0.46 | 0.25 | 0.00 | -0.01 | 0.02 | 0.00 | -0.01 | 0.00 |  |
|  | 7A | 51 | *AX-110458473* | *AX-111253793* | 2.01 | 1.46 | 0.49 | 0.16 | 0.00 | -0.01 | 0.01 | 0.00 | 0.00 | 0.00 |  |
|  | 7B | 49 | *AX-109285209* | *AX-111054336* | 2.06 | 1.25 | 0.52 | 0.26 | -0.01 | -0.01 | 0.01 | 0.01 | 0.00 | -0.01 |  |
|  | 7B | 97 | *AX-108795893* | *AX-109920134* | 2.11 | 3.69 | 0.54 | 0.77 | 0.01 | 0.00 | 0.01 | 0.01 | 0.00 | -0.03 | *QLWR.sicau-2SY-7B* |
|  | 7B | 19 | *AX-108778081* | *AX-109887886* | 1.91 | 1.23 | 0.46 | 0.16 | 0.01 | 0.00 | 0.00 | 0.00 | 0.00 | -0.01 |  |
|  | 7D | 68 | *AX-111856876* | *AX-110240876* | 2.55 | 1.33 | 0.64 | 0.17 | 0.01 | -0.01 | -0.01 | 0.01 | 0.00 | 0.00 |  |
| **KS** | 1A | 2 | *AX-108848169* | *AX-111071514* | 0.20 | 3.18 | 0.06 | 1.19 | 0.62 | 1.41 | 0.90 | -1.31 | -0.54 | -1.14 |  |
|  | 1A | 31 | *AX-108780484* | *AX-111637533* | 1.58 | 1.83 | 0.51 | 0.50 | 0.10 | 0.46 | 1.05 | 0.12 | -0.48 | -1.07 |  |
|  | 1B | 65 | *AX-94432535* | *AX-94748504* | 0.09 | 3.38 | 0.03 | 0.90 | 1.06 | -1.61 | -0.57 | -0.07 | 0.89 | 0.22 |  |
|  | 1B | 128 | *AX-89635557* | *AX-109478219* | 2.20 | 3.61 | 0.69 | 1.15 | 1.33 | -0.56 | -0.83 | 1.04 | -1.48 | -0.08 | *QKS.sicau-2SY-1B.2* |
|  | 1B | 138 | *AX-109334796* | *AX-111221126* | 3.09 | 2.23 | 0.99 | 1.55 | 1.19 | -0.28 | 0.00 | -2.32 | 0.99 | -0.33 | *QKS.sicau-2SY-1B.3* |
|  | 1B | 147 | *AX-110065453* | *AX-94433089* | 5.97 | 7.95 | 1.95 | 0.69 | -0.86 | 0.05 | 0.17 | 1.23 | 0.75 | -0.64 | ***QKS.sicau-2SY-1B*** |
|  | 1B | 168 | *AX-111688280* | *AX-108898290* | 3.33 | 4.11 | 1.09 | 1.88 | 0.95 | -0.13 | -2.79 | 0.49 | 0.72 | -0.14 |  |
|  | 1B | 197 | *AX-111496561* | *AX-110011386* | 2.25 | 0.76 | 0.72 | 0.13 | 0.11 | -0.29 | 0.03 | -0.45 | 0.34 | 0.50 |  |
|  | 1B | 23 | *AX-109376365* | *AX-109299717* | 1.40 | 1.40 | 0.45 | 0.29 | -0.29 | -0.18 | 1.13 | -0.39 | -0.21 | -0.09 |  |
|  | 1B | 32 | *AX-94872837* | *AX-89506553* | 2.71 | 2.09 | 0.88 | 0.51 | 1.40 | -0.22 | 0.01 | -0.45 | -0.60 | 0.23 |  |
|  | 1B | 54 | *AX-111127329* | *AX-108819050* | 1.60 | 1.92 | 0.51 | 0.91 | -1.40 | -0.40 | 0.20 | 1.21 | -0.40 | 1.09 |  |
|  | 1D | 8 | *AX-109304203* | *AX-108764560* | 1.50 | 1.03 | 0.48 | 0.32 | 0.34 | -0.33 | 0.95 | -0.11 | -0.73 | 0.17 |  |
|  | 1D | 68 | *AX-110556510* | *AX-109349990* | 1.52 | 1.94 | 0.49 | 0.63 | -0.50 | 0.51 | 0.71 | -1.13 | -0.37 | 1.00 |  |
|  | 1D | 99 | *AX-108797722* | *AX-111504007* | 2.92 | 1.91 | 0.95 | 0.48 | -0.20 | -0.16 | -0.35 | -0.77 | 1.13 | 0.69 |  |
|  | 1D | 112 | *AX-111584875* | *AX-111090826* | 2.10 | 0.70 | 0.67 | 0.18 | 0.02 | 0.24 | 0.18 | -0.68 | 0.31 | 0.36 |  |
|  | 1D | 119 | *AX-109862278* | *AX-110163017* | 1.94 | 0.84 | 0.63 | 0.26 | -0.18 | 0.16 | -0.05 | -0.66 | 0.58 | 0.62 |  |
|  | 1D | 141 | *AX-111604536* | *AX-89510455* | 1.38 | 1.86 | 0.45 | 0.56 | 0.36 | -0.36 | 0.44 | -1.38 | 0.70 | 0.47 |  |
|  | 2A | 68 | *AX-109404528* | *AX-108932182* | 0.45 | 2.91 | 0.14 | 0.78 | 0.47 | 1.30 | 0.55 | -1.26 | -0.61 | -0.30 |  |
|  | 2A | 76 | *AX-109291631* | *AX-109631196* | 0.40 | 2.63 | 0.12 | 0.76 | 0.44 | 1.25 | 0.40 | -1.43 | -0.44 | -0.05 |  |
|  | 2A | 81 | *AX-108918456* | *AX-108769936* | 2.39 | 1.60 | 0.77 | 0.36 | -0.79 | 1.02 | -0.42 | -0.20 | 0.15 | 0.05 |  |
|  | 2A | 86 | *AX-108917836* | *AX-109950638* | 1.87 | 1.61 | 0.57 | 0.32 | -0.82 | 0.82 | -0.02 | 0.39 | -0.28 | -0.30 |  |
|  | 2A | 102 | *AX-109417714* | *AX-110949248* | 1.27 | 1.24 | 0.37 | 0.29 | -0.83 | 0.70 | 0.05 | -0.09 | 0.38 | -0.42 |  |
|  | 2A | 107 | *AX-108865385* | *AX-108833449* | 1.09 | 1.46 | 0.33 | 0.29 | -0.86 | 0.51 | -0.33 | 0.49 | 0.39 | -0.31 |  |
|  | 2B | 9 | *AX-111640258* | *AX-108972533* | 0.92 | 1.83 | 0.30 | 0.47 | -0.67 | 0.68 | 0.93 | 0.19 | -0.36 | -0.79 |  |
|  | 2B | 71 | *AX-109897993* | *AX-109855284* | 0.53 | 2.07 | 0.17 | 0.62 | -0.90 | 0.66 | 0.89 | 0.44 | -0.20 | -1.03 |  |
|  | 2B | 96 | *AX-109420202* | *AX-109893758* | 1.05 | 1.73 | 0.34 | 0.48 | -0.74 | 0.82 | -0.42 | 0.73 | 0.32 | -0.81 |  |
|  | 2B | 130 | *AX-109915619* | *AX-110934424* | 1.83 | 1.42 | 0.58 | 0.29 | 0.06 | -0.21 | 0.19 | 0.71 | 0.17 | -0.99 |  |
|  | 2B | 153 | *AX-111457622* | *AX-108792274* | 1.60 | 2.53 | 0.52 | 0.56 | -0.13 | -0.08 | 0.44 | 1.28 | -0.98 | -0.47 |  |
|  | 2D | 26 | *AX-86163393* | *AX-109785183* | 0.13 | 2.56 | 0.04 | 0.90 | 0.81 | 0.18 | 0.74 | -0.76 | 0.81 | -1.53 |  |
|  | 2D | 9 | *AX-110604633* | *AX-111526465* | 1.44 | 1.10 | 0.47 | 0.15 | 0.39 | -0.61 | 0.21 | -0.27 | 0.40 | -0.11 | *QKS.sicau-2SY-2D.1* |
|  | 2D | 5 | *AX-110411457* | *AX-110899429* | 11.13 | 16.79 | 3.71 | 2.95 | -1.42 | -0.48 | 1.93 | -1.36 | 1.97 | -1.76 | ***QKS.sicau-2SY-2D*** |
|  | 2D | 9 | *AX-110929471* | *AX-109847853* | 4.91 | 5.21 | 1.63 | 1.91 | 1.98 | -0.19 | -0.89 | -0.67 | -1.11 | 1.78 |  |
|  | 2D | 25 | *AX-108767381* | *AX-111722527* | 5.35 | 4.18 | 1.74 | 3.65 | -1.18 | -0.61 | -0.41 | 4.10 | -0.66 | -0.63 | *QKS.sicau-2SY-2D.1* |
|  | 2D | 36 | *AX-111722527* | *AX-109421761* | 1.88 | 1.63 | 0.60 | 0.16 | -0.13 | 0.22 | 0.66 | -0.06 | -0.29 | -0.53 |  |
|  | 3A | 62 | *AX-111799835* | *AX-110407102* | 2.05 | 1.42 | 0.66 | 0.15 | 0.06 | 0.75 | 0.08 | -0.37 | -0.35 | -0.07 |  |
|  | 3A | 92 | *AX-108975984* | *AX-110539312* | 1.56 | 1.13 | 0.51 | 0.20 | -0.38 | 0.43 | 0.76 | -0.23 | -0.33 | -0.17 |  |
|  | 3A | 56 | *AX-111758075* | *AX-89644172* | 1.27 | 1.46 | 0.40 | 0.34 | -1.03 | -0.12 | 0.53 | -0.24 | 0.00 | 0.59 |  |
|  | 3B | 1 | *AX-109868743* | *AX-108758910* | 0.62 | 3.96 | 0.20 | 1.36 | 0.17 | -1.06 | 0.56 | -1.89 | 1.41 | 0.81 |  |
|  | 3B | 67 | *AX-108757678* | *AX-111007091* | 1.69 | 1.80 | 0.46 | 0.30 | 0.46 | 0.25 | 0.73 | -0.78 | -0.39 | -0.15 |  |
|  | 3D | 0 | *AX-109271722* | *AX-110941549* | 2.26 | 3.33 | 0.73 | 0.85 | -0.04 | 1.37 | 0.53 | 0.40 | -0.73 | -1.40 | *QKS.sicau-2SY-3D* |
|  | 4A | 150 | *AX-108760840* | *AX-110908322* | 2.06 | 2.09 | 0.66 | 0.63 | -0.76 | -0.75 | 1.04 | 0.53 | 0.70 | -0.65 |  |
|  | 4B | 39 | *AX-89414036* | *AX-110428871* | 0.85 | 2.06 | 0.26 | 0.43 | -0.81 | 0.23 | -0.90 | 0.49 | 0.46 | 0.66 |  |
|  | 4B | 5 | *AX-111233094* | *AX-109110130* | 0.04 | 3.19 | 0.01 | 0.97 | -1.48 | 0.88 | 0.73 | 0.99 | -1.01 | -0.19 |  |
|  | 4B | 55 | *AX-108769486* | *AX-111080767* | 2.95 | 0.97 | 0.95 | 0.17 | -0.27 | 0.01 | -0.30 | 0.65 | 0.09 | -0.51 |  |
|  | 4B | 76 | *AX-109287209* | *AX-111064477* | 2.24 | 1.48 | 0.72 | 0.48 | -0.68 | 0.05 | -0.65 | -0.53 | 0.30 | 1.11 |  |
|  | 4D | 66 | *AX-110572006* | *AX-109230716* | 1.13 | 2.33 | 0.35 | 0.58 | -0.99 | 0.95 | 0.06 | -0.33 | -0.51 | 0.96 |  |
|  | 4D | 122 | *AX-89425809* | *AX-109390946* | 1.68 | 1.34 | 0.43 | 0.21 | -0.65 | 0.04 | -0.44 | -0.03 | 0.30 | 0.64 |  |
|  | 4D | 131 | *AX-108934138* | *AX-111470564* | 0.61 | 2.78 | 0.19 | 0.98 | -0.87 | 0.06 | -0.79 | -0.71 | 0.43 | 1.80 |  |
|  | 4D | 144 | *AX-109036178* | *AX-89353317* | 1.64 | 1.38 | 0.53 | 0.38 | -0.59 | 0.87 | -0.57 | -0.44 | -0.13 | 0.68 |  |
|  | 5A | 3 | *AX-111070969* | *AX-109859109* | 2.79 | 2.16 | 0.89 | 0.50 | 0.98 | -0.29 | -0.34 | -0.09 | 0.74 | -1.04 |  |
|  | 5A | 109 | *AX-110383644* | *AX-108848765* | 0.02 | 2.67 | 0.01 | 0.95 | -0.31 | -0.98 | -0.08 | -1.03 | 0.85 | 1.52 | *QKS.sicau-2SY-5A* |
|  | 5A | 131 | *AX-108790581* | *AX-111129970* | 2.30 | 0.93 | 0.70 | 0.12 | 0.16 | -0.23 | 0.38 | -0.54 | 0.11 | 0.31 |  |
|  | 5B | 34 | *AX-108742618* | *AX-109313355* | 1.55 | 0.96 | 0.50 | 0.12 | 0.29 | -0.38 | -0.13 | 0.09 | -0.41 | 0.47 |  |
|  | 5B | 94 | *AX-108776572* | *AX-111711490* | 1.73 | 1.04 | 0.56 | 0.22 | 0.14 | 0.65 | -0.54 | -0.26 | -0.49 | 0.42 |  |
|  | 5D | 33 | *AX-89489968* | *AX-89633041* | 3.13 | 0.88 | 1.00 | 0.12 | -0.17 | 0.50 | -0.13 | -0.57 | 0.22 | 0.01 |  |
|  | 6A | 31 | *AX-109333168* | *AX-110423063* | 1.21 | 1.71 | 0.32 | 0.50 | -0.70 | -0.06 | 1.29 | -0.34 | -0.50 | 0.45 | *QKS.sicau-2SY-6A* |
|  | 6B | 122 | *AX-109935603* | *AX-108954574* | 3.15 | 0.67 | 1.02 | 0.13 | 0.09 | 0.43 | 0.25 | -0.42 | -0.09 | -0.48 |  |
|  | 6B | 133 | *AX-108926385* | *AX-110427037* | 2.04 | 1.06 | 0.65 | 0.16 | 0.07 | -0.10 | 0.25 | 0.52 | -0.15 | -0.71 |  |
|  | 6B | 16 | *AX-110384871* | *AX-109469027* | 1.72 | 1.21 | 0.55 | 0.23 | 0.09 | -0.71 | -0.31 | 0.40 | -0.34 | 0.57 |  |
|  | 6B | 35 | *AX-111112860* | *AX-111756688* | 3.76 | 1.06 | 1.21 | 0.23 | 0.28 | -0.35 | -0.61 | -0.42 | 0.04 | 0.68 |  |
|  | 6B | 85 | *AX-110515622* | *AX-110610753* | 2.74 | 1.04 | 0.87 | 0.17 | -0.18 | -0.47 | -0.52 | 0.26 | 0.09 | 0.43 |  |
|  | 6D | 65 | *AX-94618881* | *AX-110469783* | 16.63 | 3.97 | 5.60 | 1.05 | -0.49 | -1.80 | 0.50 | 0.71 | 0.92 | 0.67 | ***QKS.sicau-2SY-6D*** |
|  | 6D | 171 | *AX-110378380* | *AX-111487792* | 4.41 | 0.60 | 1.40 | 0.60 | -0.44 | -0.66 | 0.42 | 0.72 | 0.11 | 0.93 |  |
|  | 7A | 26 | *AX-109431738* | *AX-110481731* | 2.68 | 1.45 | 0.86 | 0.14 | 0.15 | -0.58 | -0.37 | 0.31 | -0.04 | 0.40 |  |
|  | 7A | 39 | *AX-111504531* | *AX-111059391* | 2.27 | 1.54 | 0.71 | 0.16 | -0.32 | -0.40 | -0.39 | 0.49 | 0.06 | 0.48 |  |
|  | 7A | 60 | *AX-109306246* | *AX-109492030* | 2.73 | 1.19 | 0.87 | 0.14 | -0.07 | -0.05 | -0.21 | -0.56 | 0.25 | 0.58 |  |
|  | 7A | 112 | *AX-109426816* | *AX-111120480* | 1.61 | 1.09 | 0.48 | 0.13 | -0.08 | -0.63 | -0.11 | 0.49 | -0.02 | 0.22 |  |
|  | 7A | 160 | *AX-109271402* | *AX-111254187* | 2.03 | 0.84 | 0.64 | 0.14 | 0.41 | -0.35 | 0.46 | -0.08 | 0.22 | -0.40 |  |
|  | 7A | 180 | *AX-108850333* | *AX-108825968* | 2.22 | 0.84 | 0.71 | 0.52 | -0.14 | -0.17 | 0.53 | 1.27 | -0.28 | -0.81 |  |
|  | 7B | 52 | *AX-111638601* | *AX-111650139* | 0.67 | 2.06 | 0.22 | 0.55 | -0.54 | 0.79 | -0.93 | -0.09 | -0.24 | 1.08 |  |
|  | 7B | 87 | *AX-89365306* | *AX-109927500* | 0.83 | 2.02 | 0.26 | 0.64 | -0.19 | 0.21 | 0.04 | -1.46 | 1.03 | 0.44 |  |
|  | 7B | 148 | *AX-110390771* | *AX-110412930* | 4.27 | 1.79 | 1.37 | 0.37 | -0.44 | -0.06 | 1.00 | -0.76 | 0.32 | 0.25 |  |
|  | 7B | 8 | *AX-111451435* | *AX-109461245* | 2.40 | 0.91 | 0.78 | 0.18 | -0.35 | -0.09 | -0.16 | -0.23 | 0.77 | 0.31 |  |
|  | 7B | 36 | *AX-108869330* | *AX-89556194* | 2.62 | 1.68 | 0.84 | 0.35 | -0.19 | -0.24 | 0.09 | -0.93 | 0.64 | 0.77 |  |
|  | 7B | 1 | *AX-110935680* | *AX-108794246* | 2.65 | 2.21 | 0.84 | 0.54 | -0.45 | -0.04 | 0.72 | -0.99 | -0.27 | 1.08 |  |
|  | 7B | 4 | *AX-110470708* | *AX-109427696* | 1.78 | 1.93 | 0.57 | 0.45 | 0.06 | -0.11 | 0.45 | -1.07 | -0.35 | 0.98 |  |
|  | 7B | 14 | *AX-109955495* | *AX-108778081* | 2.46 | 1.15 | 0.80 | 0.09 | -0.40 | -0.13 | 0.55 | -0.15 | -0.03 | 0.16 |  |
|  | 7B | 25 | *AX-109926256* | *AX-89540697* | 3.15 | 1.36 | 1.02 | 0.13 | -0.36 | -0.06 | 0.36 | -0.13 | -0.35 | 0.58 |  |
|  | 7B | 42 | *AX-109995969* | *AX-109489314* | 1.44 | 1.53 | 0.46 | 0.18 | -0.13 | -0.24 | 0.62 | -0.46 | -0.38 | 0.39 |  |
|  | 7D | 41 | *AX-111902534* | *AX-110443275* | 1.98 | 1.48 | 0.63 | 0.23 | -0.01 | 0.96 | 0.09 | -0.39 | -0.23 | -0.34 |  |
|  | 7D | 56 | *AX-110146812* | *AX-111609634* | 0.36 | 2.34 | 0.12 | 0.75 | 0.13 | 1.55 | 0.22 | -1.17 | -0.56 | -0.08 |  |
|  | 7D | 61 | *AX-111011171* | *AX-111542493* | 1.41 | 1.70 | 0.45 | 0.54 | -0.21 | 1.33 | 0.32 | -0.93 | -0.21 | -0.36 |  |
|  | 7D | 82 | *AX-108912162* | *AX-108797663* | 1.95 | 2.15 | 0.59 | 0.51 | 0.32 | 1.09 | 0.26 | -0.02 | -0.90 | -0.79 |  |
| **FFD** | 1A | 2 | *AX-108848169* | *AX-111071514* | 0.20 | 3.18 | 0.06 | 1.19 | 0.62 | 1.41 | 0.90 | -1.31 | -0.54 | -1.14 |  |
|  | 1A | 31 | *AX-108780484* | *AX-111637533* | 1.58 | 1.83 | 0.51 | 0.50 | 0.10 | 0.46 | 1.05 | 0.12 | -0.48 | -1.07 |  |
|  | 1B | 65 | *AX-94432535* | *AX-94748504* | 0.09 | 3.38 | 0.03 | 0.90 | 1.06 | -1.61 | -0.57 | -0.07 | 0.89 | 0.22 |  |
|  | 1B | 128 | *AX-89635557* | *AX-109478219* | 2.20 | 3.61 | 0.69 | 1.15 | 1.33 | -0.56 | -0.83 | 1.04 | -1.48 | -0.08 |  |
|  | 1B | 138 | *AX-109334796* | *AX-111221126* | 3.09 | 2.23 | 0.99 | 1.55 | 1.19 | -0.28 | 0.00 | -2.32 | 0.99 | -0.33 |  |
|  | 1B | 147 | *AX-110065453* | *AX-94433089* | 5.97 | 7.95 | 1.95 | 0.69 | -0.86 | 0.05 | 0.17 | 1.23 | 0.75 | -0.64 |  |
|  | 1B | 168 | *AX-111688280* | *AX-108898290* | 3.33 | 4.11 | 1.09 | 1.88 | 0.95 | -0.13 | -2.79 | 0.49 | 0.72 | -0.14 |  |
|  | 1B | 197 | *AX-111496561* | *AX-110011386* | 2.25 | 0.76 | 0.72 | 0.13 | 0.11 | -0.29 | 0.03 | -0.45 | 0.34 | 0.50 |  |
|  | 1B | 23 | *AX-109376365* | *AX-109299717* | 1.40 | 1.40 | 0.45 | 0.29 | -0.29 | -0.18 | 1.13 | -0.39 | -0.21 | -0.09 |  |
|  | 1B | 32 | *AX-94872837* | *AX-89506553* | 2.71 | 2.09 | 0.88 | 0.51 | 1.40 | -0.22 | 0.01 | -0.45 | -0.60 | 0.23 |  |
|  | 1B | 54 | *AX-111127329* | *AX-108819050* | 1.60 | 1.92 | 0.51 | 0.91 | -1.40 | -0.40 | 0.20 | 1.21 | -0.40 | 1.09 |  |
|  | 1D | 8 | *AX-109304203* | *AX-108764560* | 1.50 | 1.03 | 0.48 | 0.32 | 0.34 | -0.33 | 0.95 | -0.11 | -0.73 | 0.17 |  |
|  | 1D | 68 | *AX-110556510* | *AX-109349990* | 1.52 | 1.94 | 0.49 | 0.63 | -0.50 | 0.51 | 0.71 | -1.13 | -0.37 | 1.00 |  |
|  | 1D | 99 | *AX-108797722* | *AX-111504007* | 2.92 | 1.91 | 0.95 | 0.48 | -0.20 | -0.16 | -0.35 | -0.77 | 1.13 | 0.69 |  |
|  | 1D | 112 | *AX-111584875* | *AX-111090826* | 2.10 | 0.70 | 0.67 | 0.18 | 0.02 | 0.24 | 0.18 | -0.68 | 0.31 | 0.36 |  |
|  | 1D | 119 | *AX-109862278* | *AX-110163017* | 1.94 | 0.84 | 0.63 | 0.26 | -0.18 | 0.16 | -0.05 | -0.66 | 0.58 | 0.62 |  |
|  | 1D | 141 | *AX-111604536* | *AX-89510455* | 1.38 | 1.86 | 0.45 | 0.56 | 0.36 | -0.36 | 0.44 | -1.38 | 0.70 | 0.47 |  |
|  | 2A | 68 | *AX-109404528* | *AX-108932182* | 0.45 | 2.91 | 0.14 | 0.78 | 0.47 | 1.30 | 0.55 | -1.26 | -0.61 | -0.30 |  |
|  | 2A | 76 | *AX-109291631* | *AX-109631196* | 0.40 | 2.63 | 0.12 | 0.76 | 0.44 | 1.25 | 0.40 | -1.43 | -0.44 | -0.05 |  |
|  | 2A | 81 | *AX-108918456* | *AX-108769936* | 2.39 | 1.60 | 0.77 | 0.36 | -0.79 | 1.02 | -0.42 | -0.20 | 0.15 | 0.05 |  |
|  | 2A | 86 | *AX-108917836* | *AX-109950638* | 1.87 | 1.61 | 0.57 | 0.32 | -0.82 | 0.82 | -0.02 | 0.39 | -0.28 | -0.30 |  |
|  | 2A | 102 | *AX-109417714* | *AX-110949248* | 1.27 | 1.24 | 0.37 | 0.29 | -0.83 | 0.70 | 0.05 | -0.09 | 0.38 | -0.42 |  |
|  | 2A | 107 | *AX-108865385* | *AX-108833449* | 1.09 | 1.46 | 0.33 | 0.29 | -0.86 | 0.51 | -0.33 | 0.49 | 0.39 | -0.31 |  |
|  | 2B | 9 | *AX-111640258* | *AX-108972533* | 0.92 | 1.83 | 0.30 | 0.47 | -0.67 | 0.68 | 0.93 | 0.19 | -0.36 | -0.79 |  |
|  | 2B | 71 | *AX-109897993* | *AX-109855284* | 0.53 | 2.07 | 0.17 | 0.62 | -0.90 | 0.66 | 0.89 | 0.44 | -0.20 | -1.03 |  |
|  | 2B | 96 | *AX-109420202* | *AX-109893758* | 1.05 | 1.73 | 0.34 | 0.48 | -0.74 | 0.82 | -0.42 | 0.73 | 0.32 | -0.81 |  |
|  | 2B | 130 | *AX-109915619* | *AX-110934424* | 1.83 | 1.42 | 0.58 | 0.29 | 0.06 | -0.21 | 0.19 | 0.71 | 0.17 | -0.99 |  |
|  | 2B | 153 | *AX-111457622* | *AX-108792274* | 1.60 | 2.53 | 0.52 | 0.56 | -0.13 | -0.08 | 0.44 | 1.28 | -0.98 | -0.47 |  |
|  | 2D | 26 | *AX-86163393* | *AX-109785183* | 0.13 | 2.56 | 0.04 | 0.90 | 0.81 | 0.18 | 0.74 | -0.76 | 0.81 | -1.53 |  |
|  | 2D | 9 | *AX-110604633* | *AX-111526465* | 1.44 | 1.10 | 0.47 | 0.15 | 0.39 | -0.61 | 0.21 | -0.27 | 0.40 | -0.11 |  |
|  | 2D | 5 | *AX-110411457* | *AX-110899429* | 11.13 | 16.79 | 3.71 | 2.95 | -1.42 | -0.48 | 1.93 | -1.36 | 1.97 | -1.76 | ***QFFD.sicau-2SY-2D*** |
|  | 2D | 9 | *AX-110929471* | *AX-109847853* | 4.91 | 5.21 | 1.63 | 1.91 | 1.98 | -0.19 | -0.89 | -0.67 | -1.11 | 1.78 |  |
|  | 2D | 25 | *AX-108767381* | *AX-111722527* | 5.35 | 4.18 | 1.74 | 3.65 | -1.18 | -0.61 | -0.41 | 4.10 | -0.66 | -0.63 | ***QFFD.sicau-2SY-2D.1*** |
|  | 2D | 36 | *AX-111722527* | *AX-109421761* | 1.88 | 1.63 | 0.60 | 0.16 | -0.13 | 0.22 | 0.66 | -0.06 | -0.29 | -0.53 |  |
|  | 3A | 62 | *AX-111799835* | *AX-110407102* | 2.05 | 1.42 | 0.66 | 0.15 | 0.06 | 0.75 | 0.08 | -0.37 | -0.35 | -0.07 |  |
|  | 3A | 92 | *AX-108975984* | *AX-110539312* | 1.56 | 1.13 | 0.51 | 0.20 | -0.38 | 0.43 | 0.76 | -0.23 | -0.33 | -0.17 |  |
|  | 3A | 56 | *AX-111758075* | *AX-89644172* | 1.27 | 1.46 | 0.40 | 0.34 | -1.03 | -0.12 | 0.53 | -0.24 | 0.00 | 0.59 |  |
|  | 3B | 1 | *AX-109868743* | *AX-108758910* | 0.62 | 3.96 | 0.20 | 1.36 | 0.17 | -1.06 | 0.56 | -1.89 | 1.41 | 0.81 |  |
|  | 3B | 67 | *AX-108757678* | *AX-111007091* | 1.69 | 1.80 | 0.46 | 0.30 | 0.46 | 0.25 | 0.73 | -0.78 | -0.39 | -0.15 |  |
|  | 3D | 0 | *AX-109271722* | *AX-110941549* | 2.26 | 3.33 | 0.73 | 0.85 | -0.04 | 1.37 | 0.53 | 0.40 | -0.73 | -1.40 |  |
|  | 4A | 150 | *AX-108760840* | *AX-110908322* | 2.06 | 2.09 | 0.66 | 0.63 | -0.76 | -0.75 | 1.04 | 0.53 | 0.70 | -0.65 |  |
|  | 4B | 39 | *AX-89414036* | *AX-110428871* | 0.85 | 2.06 | 0.26 | 0.43 | -0.81 | 0.23 | -0.90 | 0.49 | 0.46 | 0.66 |  |
|  | 4B | 5 | *AX-111233094* | *AX-109110130* | 0.04 | 3.19 | 0.01 | 0.97 | -1.48 | 0.88 | 0.73 | 0.99 | -1.01 | -0.19 |  |
|  | 4B | 55 | *AX-108769486* | *AX-111080767* | 2.95 | 0.97 | 0.95 | 0.17 | -0.27 | 0.01 | -0.30 | 0.65 | 0.09 | -0.51 |  |
|  | 4B | 76 | *AX-109287209* | *AX-111064477* | 2.24 | 1.48 | 0.72 | 0.48 | -0.68 | 0.05 | -0.65 | -0.53 | 0.30 | 1.11 |  |
|  | 4D | 66 | *AX-110572006* | *AX-109230716* | 1.13 | 2.33 | 0.35 | 0.58 | -0.99 | 0.95 | 0.06 | -0.33 | -0.51 | 0.96 |  |
|  | 4D | 122 | *AX-89425809* | *AX-109390946* | 1.68 | 1.34 | 0.43 | 0.21 | -0.65 | 0.04 | -0.44 | -0.03 | 0.30 | 0.64 |  |
|  | 4D | 131 | *AX-108934138* | *AX-111470564* | 0.61 | 2.78 | 0.19 | 0.98 | -0.87 | 0.06 | -0.79 | -0.71 | 0.43 | 1.80 |  |
|  | 4D | 144 | *AX-109036178* | *AX-89353317* | 1.64 | 1.38 | 0.53 | 0.38 | -0.59 | 0.87 | -0.57 | -0.44 | -0.13 | 0.68 |  |
|  | 5A | 3 | *AX-111070969* | *AX-109859109* | 2.79 | 2.16 | 0.89 | 0.50 | 0.98 | -0.29 | -0.34 | -0.09 | 0.74 | -1.04 |  |
|  | 5A | 109 | *AX-110383644* | *AX-108848765* | 0.02 | 2.67 | 0.01 | 0.95 | -0.31 | -0.98 | -0.08 | -1.03 | 0.85 | 1.52 |  |
|  | 5A | 131 | *AX-108790581* | *AX-111129970* | 2.30 | 0.93 | 0.70 | 0.12 | 0.16 | -0.23 | 0.38 | -0.54 | 0.11 | 0.31 |  |
|  | 5B | 34 | *AX-108742618* | *AX-109313355* | 1.55 | 0.96 | 0.50 | 0.12 | 0.29 | -0.38 | -0.13 | 0.09 | -0.41 | 0.47 |  |
|  | 5B | 94 | *AX-108776572* | *AX-111711490* | 1.73 | 1.04 | 0.56 | 0.22 | 0.14 | 0.65 | -0.54 | -0.26 | -0.49 | 0.42 |  |
|  | 5D | 33 | *AX-89489968* | *AX-89633041* | 3.13 | 0.88 | 1.00 | 0.12 | -0.17 | 0.50 | -0.13 | -0.57 | 0.22 | 0.01 |  |
|  | 6A | 31 | *AX-109333168* | *AX-110423063* | 1.21 | 1.71 | 0.32 | 0.50 | -0.70 | -0.06 | 1.29 | -0.34 | -0.50 | 0.45 |  |
|  | 6B | 122 | *AX-109935603* | *AX-108954574* | 3.15 | 0.67 | 1.02 | 0.13 | 0.09 | 0.43 | 0.25 | -0.42 | -0.09 | -0.48 |  |
|  | 6B | 133 | *AX-108926385* | *AX-110427037* | 2.04 | 1.06 | 0.65 | 0.16 | 0.07 | -0.10 | 0.25 | 0.52 | -0.15 | -0.71 |  |
|  | 6B | 16 | *AX-110384871* | *AX-109469027* | 1.72 | 1.21 | 0.55 | 0.23 | 0.09 | -0.71 | -0.31 | 0.40 | -0.34 | 0.57 |  |
|  | 6B | 35 | *AX-111112860* | *AX-111756688* | 3.76 | 1.06 | 1.21 | 0.23 | 0.28 | -0.35 | -0.61 | -0.42 | 0.04 | 0.68 |  |
|  | 6B | 85 | *AX-110515622* | *AX-110610753* | 2.74 | 1.04 | 0.87 | 0.17 | -0.18 | -0.47 | -0.52 | 0.26 | 0.09 | 0.43 |  |
|  | 6D | 65 | *AX-94618881* | *AX-110469783* | 16.63 | 3.97 | 5.60 | 1.05 | -0.49 | -1.80 | 0.50 | 0.71 | 0.92 | 0.67 |  |
|  | 6D | 171 | *AX-110378380* | *AX-111487792* | 4.41 | 0.60 | 1.40 | 0.60 | -0.44 | -0.66 | 0.42 | 0.72 | 0.11 | 0.93 |  |
|  | 7A | 26 | *AX-109431738* | *AX-110481731* | 2.68 | 1.45 | 0.86 | 0.14 | 0.15 | -0.58 | -0.37 | 0.31 | -0.04 | 0.40 |  |
|  | 7A | 39 | *AX-111504531* | *AX-111059391* | 2.27 | 1.54 | 0.71 | 0.16 | -0.32 | -0.40 | -0.39 | 0.49 | 0.06 | 0.48 |  |
|  | 7A | 60 | *AX-109306246* | *AX-109492030* | 2.73 | 1.19 | 0.87 | 0.14 | -0.07 | -0.05 | -0.21 | -0.56 | 0.25 | 0.58 |  |
|  | 7A | 112 | *AX-109426816* | *AX-111120480* | 1.61 | 1.09 | 0.48 | 0.13 | -0.08 | -0.63 | -0.11 | 0.49 | -0.02 | 0.22 |  |
|  | 7A | 160 | *AX-109271402* | *AX-111254187* | 2.03 | 0.84 | 0.64 | 0.14 | 0.41 | -0.35 | 0.46 | -0.08 | 0.22 | -0.40 |  |
|  | 7A | 180 | *AX-108850333* | *AX-108825968* | 2.22 | 0.84 | 0.71 | 0.52 | -0.14 | -0.17 | 0.53 | 1.27 | -0.28 | -0.81 |  |
|  | 7A | 52 | *AX-111638601* | *AX-111650139* | 0.67 | 2.06 | 0.22 | 0.55 | -0.54 | 0.79 | -0.93 | -0.09 | -0.24 | 1.08 |  |
|  | 7B | 87 | *AX-89365306* | *AX-109927500* | 0.83 | 2.02 | 0.26 | 0.64 | -0.19 | 0.21 | 0.04 | -1.46 | 1.03 | 0.44 |  |
|  | 7B | 148 | *AX-110390771* | *AX-110412930* | 4.27 | 1.79 | 1.37 | 0.37 | -0.44 | -0.06 | 1.00 | -0.76 | 0.32 | 0.25 |  |
|  | 7B | 8 | *AX-111451435* | *AX-109461245* | 2.40 | 0.91 | 0.78 | 0.18 | -0.35 | -0.09 | -0.16 | -0.23 | 0.77 | 0.31 |  |
|  | 7B | 36 | *AX-108869330* | *AX-89556194* | 2.62 | 1.68 | 0.84 | 0.35 | -0.19 | -0.24 | 0.09 | -0.93 | 0.64 | 0.77 |  |
|  | 7B | 1 | *AX-110935680* | *AX-108794246* | 2.65 | 2.21 | 0.84 | 0.54 | -0.45 | -0.04 | 0.72 | -0.99 | -0.27 | 1.08 |  |
|  | 7B | 4 | *AX-110470708* | *AX-109427696* | 1.78 | 1.93 | 0.57 | 0.45 | 0.06 | -0.11 | 0.45 | -1.07 | -0.35 | 0.98 |  |
|  | 7B | 14 | *AX-109955495* | *AX-108778081* | 2.46 | 1.15 | 0.80 | 0.09 | -0.40 | -0.13 | 0.55 | -0.15 | -0.03 | 0.16 |  |
|  | 7B | 25 | *AX-109926256* | *AX-89540697* | 3.15 | 1.36 | 1.02 | 0.13 | -0.36 | -0.06 | 0.36 | -0.13 | -0.35 | 0.58 |  |
|  | 7B | 42 | *AX-109995969* | *AX-109489314* | 1.44 | 1.53 | 0.46 | 0.18 | -0.13 | -0.24 | 0.62 | -0.46 | -0.38 | 0.39 |  |
|  | 7D | 41 | *AX-111902534* | *AX-110443275* | 1.98 | 1.48 | 0.63 | 0.23 | -0.01 | 0.96 | 0.09 | -0.39 | -0.23 | -0.34 |  |
|  | 7D | 56 | *AX-110146812* | *AX-111609634* | 0.36 | 2.34 | 0.12 | 0.75 | 0.13 | 1.55 | 0.22 | -1.17 | -0.56 | -0.08 |  |
|  | 7D | 61 | *AX-111011171* | *AX-111542493* | 1.41 | 1.70 | 0.45 | 0.54 | -0.21 | 1.33 | 0.32 | -0.93 | -0.21 | -0.36 |  |
|  | 7D | 82 | *AX-108912162* | *AX-108797663* | 1.95 | 2.15 | 0.59 | 0.51 | 0.32 | 1.09 | 0.26 | -0.02 | -0.90 | -0.79 |  |

Note: LOD (A): LOD score for additive and dominance effects.

LOD (A by E): LOD score for additive and dominance by environment effects.

PVE (A): Phenotypic variation explained by additive and dominance effect at the current scanning position.

PVE (A by E): Phenotypic variation explained by additive and dominance by environment effect at the current scanning position.
