# Supplementary material for: Genetic Mapping and Validation of Loci for Kernel-Related Traits in Wheat (Triticum aestivum L.)
Source: Front Plant Sci. 2021 Jun 7;12:667493. doi: 10.3389/fpls.2021.667493 (PMC8215603; doi:10.3389/fpls.2021.667493)
Supplement: Supplementary Table 9 — Predicted genes in the interval of the major QTL (S9.1–9.3). [file Table_9.DOCX]

**Table S9** Predicted genes in the interval of the major QTL (TableS9.1-9.3)

**Table S9.1** Predicted genes in the interval of the major QTL *QKL.sicau-2SY-1B* and *QKS.sicau-2SY-1B*

| **Gene ID in CS** | **Annotation** | **Orthologs in Wild emmer** | **References** |
| --- | --- | --- | --- |
| *TraesCS1B01G349900.1* | **Glycosyltransferase** | *TRIDC1BG056750.1* | [1] |
| *TraesCS1B01G351200.1* | **VQ motif-containing protein** | *TRIDC1BG056890.1* | [2] |
| *TraesCS1B01G338700.1* | **B3 domain-containing protein family** | *TRIDC1BG054930.1* | [3] |
| *TraesCS1B01G349800.1* | **NHL domain-containing protein** | *TRIDC1BG056740.1* | [4] |
| *TraesCS1B01G345000.1* | **Carboxypeptidase** | *TRIDC1BG055900.1* | [5] |
| *TraesCS1B01G344600.1* | **F-box family protein** | *TRIDC1BG055870.1* | [6] |
| *TraesCS1B01G342700.1* | **F-box protein** | *TRIDC1BG055580.1* | [7] |
| *TraesCS1B01G345900.1* | Clavata3/ESR (CLE) gene family member | *TRIDC1BG056090.1* | [8] |
| *TraesCS1B01G347700.1* | Cytochrome P450 | *TRIDC1BG056370.1* | [9] |
| *TraesCS1B01G342500.1* | Ethylene-responsive transcription factor | *TRIDC1BG055500.1* | [10] |
| *TraesCS1B01G350800.1* | Non-symbiotic hemoglobin | *TRIDC1BG056850.1* | [11] |
| *TraesCS1B01G348200.1* | O-methyltransferase | *TRIDC1BG056420.1* | [12] |
| *TraesCS1B01G348400.1* | O-methyltransferase-like protein | *TRIDC1BG056460.1* | [12] |
| *TraesCS1B01G348700.1* | O-methyltransferase-like protein | *TRIDC1BG056540.1* | [12] |
| *TraesCS1B01G344000.1* | Phosphate transporter | *TRIDC1BG055760.1* | [13] |
| *TraesCS1B01G338900.1* | rapid alkalinization factor 23 | *TRIDC1BG054960.1* | [14] |
| *TraesCS1B01G351000.1* | RNA ligase/cyclic nucleotide phosphodiesterase family protein | *TRIDC1BG056860.1* | [15] |
| *TraesCS1B01G349600.1* | rRNA N-glycosidase | *TRIDC1BG056730.1* | [16] |
| *TraesCS1B01G351400.1* | senescence regulator (Protein of unknown function, DUF584) | *TRIDC1BG056910.1* | [17] |
| *TraesCS1B01G340600.1* | Signal recognition particle 54 kDa protein | *TRIDC1BG056380.1* | [18] |
| *TraesCS1B01G338800.1* | Thioredoxin | *TRIDC1BG054950.1* | [19] |
| *TraesCS1B01G339000.1* | Thioredoxin | *TRIDC1BG055020.1* | [19] |
| *TraesCS1B01G343700.1* | Thioredoxin | *TRIDC1BG055690.1* | [19] |
| *TraesCS1B01G350600.1* | Trehalose-6-phosphate synthase 6 | *TRIDC1BG056830.1* | [20] |
| *TraesCS1B01G347200.1* | Tubby-like F-box protein | *TRIDC1BG056320.1* | [21] |
| *TraesCS1B01G343000.1* | Tyrosine decarboxylase | *TRIDC1BG055600.1* | [22] |
| *TraesCS1B01G345200.1* | 2-oxoglutarate (2OG) and Fe(II)-dependent oxygenase superfamily protein | *TRIDC1BG055940.1* | N |
| *TraesCS1B01G339400.1* | 30S ribosomal protein S19 | *TRIDC1BG055080.1* | N |
| *TraesCS1B01G346800.1* | 50S ribosomal protein L33 | *TRIDC1BG056240.1* | N |
| *TraesCS1B01G349400.1* | Anaerobic nitric oxide reductase flavorubredoxin | *TRIDC1BG056700.1* | N |
| *TraesCS1B01G352400.1* | ATP-dependent chaperone ClpB | *TRIDC1BG057070.1* | N |
| *TraesCS1B01G346000.1* | BAG family molecular chaperone regulator 8, chloroplastic | *TRIDC1BG056100.1* | N |
| *TraesCS1B01G343500.1* | Basic-leucine zipper (BZIP) transcription factor family | *TRIDC1BG055660.1* | N |
| *TraesCS1B01G345500.1* | BSD domain containing protein | *TRIDC1BG056040.1* | N |
| *TraesCS1B01G346400.1* | Calcium-transporting ATPase | *TRIDC1BG056190.1* | N |
| *TraesCS1B01G340100.1* | Citrate-binding protein | *TRIDC1BG055200.1* | N |
| *TraesCS1B01G346600.1* | Cleavage stimulation factor subunit | *TRIDC1BG056210.1* | N |
| *TraesCS1B01G340900.1* | DUF1677 family protein | *TRIDC1BG055290.1* | N |
| *TraesCS1B01G352000.1* | DUF506 family protein | *TRIDC1BG057010.1* | N |
| *TraesCS1B01G352100.1* | DUF506 family protein | *TRIDC1BG057030.1* | N |
| *TraesCS1B01G350300.1* | Gamma-glutamyl phosphate reductase | *TRIDC1BG056790.1* | N |
| *TraesCS1B01G345800.1* | Lysine--trna ligase | *TRIDC1BG056070.1* | N |
| *TraesCS1B01G339500.1* | Photosystem II reaction center W protein | *TRIDC1BG055090.1* | N |
| *TraesCS1B01G339700.1* | Photosystem II reaction center W protein, chloroplastic | *TRIDC1BG055160.1* | N |
| *TraesCS1B01G339100.1* | p-loop containing nucleoside triphosphate hydrolases superfamily protein | *TRIDC1BG055050.1* | N |
| *TraesCS1B01G341700.1* | Poly [ADP-ribose] polymerase | *TRIDC1BG055410.1* | N |
| *TraesCS1B01G350200.1* | Ras-like protein | *TRIDC1BG056780.1* | N |
| *TraesCS1B01G352500.1* | Transcriptional regulatory plant protein, putative | *TRIDC1BG057090.1* | N |
| *TraesCS1B01G339300.1* | Ubiquitin carboxyl-terminal hydrolase 2 | *TRIDC1BG055070.1* | N |
| *TraesCS1B01G351500.1* | UPF0502 protein YceH | *TRIDC1BG056920.1* | N |
| *TraesCS1B01G350400.1* | Very long-chain-fatty-acid--CoA ligase bubblegum | *TRIDC1BG056800.1* | N |
| *TraesCS1B01G338600.1* |  | N |  |
| *TraesCS1B01G339200.1* |  | N |  |
| *TraesCS1B01G339600.1* |  | N |  |
| *TraesCS1B01G339800.1* |  | N |  |
| *TraesCS1B01G339900.1* |  | N |  |
| *TraesCS1B01G340000.1* |  | N |  |
| *TraesCS1B01G340200.1* |  | N |  |
| *TraesCS1B01G338600.1* |  | N |  |
| *TraesCS1B01G339200.1* |  | N |  |
| *TraesCS1B01G340300.1* |  | N |  |
| *TraesCS1B01G340400.1* |  | N |  |
| *TraesCS1B01G340500.1* |  | N |  |
| *TraesCS1B01G340700.1* |  | N |  |
| *TraesCS1B01G340800.1* |  | N |  |
| *TraesCS1B01G341000.1* |  | N |  |
| *TraesCS1B01G341100.1* |  | N |  |
| *TraesCS1B01G341200.1* |  | N |  |
| *TraesCS1B01G341300.1* |  | N |  |
| *TraesCS1B01G341400.1* |  | N |  |
| *TraesCS1B01G341500.1* |  |  |  |
| *TraesCS1B01G341600.1* |  | N |  |
| *TraesCS1B01G341800.1* |  | N |  |
| *TraesCS1B01G341900.1* |  | N |  |
| *TraesCS1B01G342000.1* |  | N |  |
| *TraesCS1B01G342100.1* |  | N |  |
| *TraesCS1B01G342200.1* |  | N |  |
| *TraesCS1B01G342300.1* |  | N |  |
| *TraesCS1B01G342400.1* |  | N |  |
| *TraesCS1B01G342600.1* |  | N |  |
| *TraesCS1B01G342800.1* |  | N |  |
| *TraesCS1B01G342900.1* |  | N |  |
| *TraesCS1B01G343100.1* |  | N |  |
| *TraesCS1B01G343200.1* |  | N |  |
| *TraesCS1B01G343300.1* |  | N |  |
| *TraesCS1B01G343400.1* |  | N |  |
| *TraesCS1B01G343600.1* |  | N |  |
| *TraesCS1B01G343800.1* |  | N |  |
| *TraesCS1B01G343900.1* |  | N |  |
| *TraesCS1B01G344100.1* |  | N |  |
| *TraesCS1B01G344200.1* |  | N |  |
| *TraesCS1B01G344300.1* |  | N |  |
| *TraesCS1B01G344400.1* |  | N |  |
| *TraesCS1B01G344500.1* |  | N |  |
| *TraesCS1B01G344700.1* |  | N |  |
| *TraesCS1B01G344800.1* |  | N |  |
| *TraesCS1B01G344900.1* |  | N |  |
| *TraesCS1B01G345100.1* |  | N |  |
| *TraesCS1B01G345300.1* |  | N |  |
| *TraesCS1B01G345400.1* |  | N |  |
| *TraesCS1B01G345600.1* |  | N |  |
| *TraesCS1B01G345700.1* |  | N |  |
| *TraesCS1B01G346100.1* |  | N |  |
| *TraesCS1B01G346200.1* |  | N |  |
| *TraesCS1B01G346300.1* |  | N |  |
| *TraesCS1B01G346500.1* |  | N |  |
| *TraesCS1B01G346700.1* |  | N |  |
| *TraesCS1B01G346900.1* |  | N |  |
| *TraesCS1B01G347000.1* |  | N |  |
| *TraesCS1B01G345100.1* |  | N |  |
| *TraesCS1B01G345300.1* |  | N |  |
| *TraesCS1B01G345400.1* |  | N |  |
| *TraesCS1B01G345600.1* |  | N |  |
| *TraesCS1B01G345700.1* |  | N |  |
| *TraesCS1B01G346100.1* |  | N |  |
| *TraesCS1B01G346200.1* |  | N |  |
| *TraesCS1B01G346300.1* |  | N |  |
| *TraesCS1B01G346500.1* |  | N |  |
| *TraesCS1B01G346700.1* |  | N |  |
| *TraesCS1B01G346900.1* |  | N |  |
| *TraesCS1B01G347000.1* |  | N |  |
| *TraesCS1B01G347100.1* |  | N |  |
| *TraesCS1B01G347300.1* |  | N |  |
| *TraesCS1B01G347400.1* |  | N |  |
| *TraesCS1B01G347500.1* |  | N |  |
| *TraesCS1B01G347600.1* |  | N |  |
| *TraesCS1B01G347800.1* |  | N |  |
| *TraesCS1B01G347900.1* |  | N |  |
| *TraesCS1B01G348000.1* |  | N |  |
| *TraesCS1B01G348100.1* |  | N |  |
| *TraesCS1B01G348300.1* |  | N |  |
| *TraesCS1B01G348500.1* |  | N |  |
| *TraesCS1B01G348600.1* |  | N |  |
| *TraesCS1B01G348800.1* |  | N |  |
| *TraesCS1B01G348900.1* |  | N |  |
| *TraesCS1B01G349000.1* |  | N |  |
| *TraesCS1B01G349100.1* |  | N |  |
| *TraesCS1B01G349200.1* |  | N |  |
| *TraesCS1B01G349300.1* |  | N |  |
| *TraesCS1B01G349500.1* |  | N |  |
| *TraesCS1B01G349700.1* |  | N |  |
| *TraesCS1B01G350000.1* |  | N |  |
| *TraesCS1B01G350100.1* |  | N |  |
| *TraesCS1B01G350500.1* |  | N |  |
| *TraesCS1B01G350700.1* |  | N |  |
| *TraesCS1B01G350900.1* |  | N |  |
| *TraesCS1B01G351100.1* |  | N |  |
| *TraesCS1B01G351300.1* |  | N |  |
| *TraesCS1B01G351600.1* |  | N |  |
| *TraesCS1B01G351700.1* |  | N |  |
| *TraesCS1B01G351800.1* |  | N |  |
| *TraesCS1B01G351900.1* |  | N |  |
| *TraesCS1B01G352200.1* |  | N |  |
| *TraesCS1B01G352300.1* |  | N |  |
| *TraesCS1B01G352600.1* |  | N |  |
| *TraesCS1B01G352700.1* |  | N |  |
| *TraesCS1B01G352800.1* |  | N |  |
| *TraesCS1B01G352900.1* |  | N |  |
| *TraesCS1B01G353000.1* |  | N |  |
| *TraesCS1B01G353100.1* |  | N |  |
| *TraesCS1B01G353200.1* |  | N |  |
| *TraesCS1B01G353300.1* |  | N |  |
| N |  | *TRIDC1BG054910.1* |  |
| N |  | *TRIDC1BG054920.1* |  |
| N |  | *TRIDC1BG054940.1* |  |
| N |  | *TRIDC1BG054970.1* |  |
| N |  | *TRIDC1BG054980.1* |  |
| N |  | *TRIDC1BG054990.1* |  |
| N |  | *TRIDC1BG055000.1* |  |
| N |  | *TRIDC1BG055010.1* |  |
| N |  | *TRIDC1BG055030.1* |  |
| N |  | *TRIDC1BG055040.1* |  |
| N |  | *TRIDC1BG055060.1* |  |
| N |  | *TRIDC1BG055100.1* |  |
| N |  | *TRIDC1BG055110.1* |  |
| N |  | *TRIDC1BG055120.1* |  |
| N |  | *TRIDC1BG055130.1* |  |
| N |  | *TRIDC1BG055140.1* |  |
| N |  | *TRIDC1BG055150.1* |  |
| N |  | *TRIDC1BG055170.1* |  |
| N |  | *TRIDC1BG055180.1* |  |
| N |  | *TRIDC1BG055190.1* |  |
| N |  | *TRIDC1BG055210.1* |  |
| N |  | *TRIDC1BG055220.1* |  |
| N |  | *TRIDC1BG055230.1* |  |
| N |  | *TRIDC1BG055240.1* |  |
| N |  | *TRIDC1BG055250.1* |  |
| N |  | *TRIDC1BG055260.1* |  |
| N |  | *TRIDC1BG055270.1* |  |
| N |  | *TRIDC1BG055280.1* |  |
| N |  | *TRIDC1BG055300.1* |  |
| N |  | *TRIDC1BG055310.1* |  |
| N |  | *TRIDC1BG055320.1* |  |
| N |  | *TRIDC1BG055330.1* |  |
| N |  | *TRIDC1BG055340.1* |  |
| N |  | *TRIDC1BG055350.1* |  |
| N |  | *TRIDC1BG055360.1* |  |
| N |  | *TRIDC1BG055370.1* |  |
| N |  | *TRIDC1BG055380.1* |  |
| N |  | *TRIDC1BG055390.1* |  |
| N |  | *TRIDC1BG055400.1* |  |
| N |  | *TRIDC1BG055420.1* |  |
| N |  | *TRIDC1BG055430.1* |  |
| N |  | *TRIDC1BG055440.1* |  |
| N |  | *TRIDC1BG055450.1* |  |
| N |  | *TRIDC1BG055460.1* |  |
| N |  | *TRIDC1BG055470.1* |  |
| N |  | *TRIDC1BG055480.1* |  |
| N |  | *TRIDC1BG055490.1* |  |
| N |  | *TRIDC1BG055510.1* |  |
| N |  | *TRIDC1BG055520.1* |  |
| N |  | *TRIDC1BG055530.1* |  |
| N |  | *TRIDC1BG055540.1* |  |
| N |  | *TRIDC1BG055550.1* |  |
| N |  | *TRIDC1BG055560.1* |  |
| N |  | *TRIDC1BG055570.1* |  |
| N |  | *TRIDC1BG055590.1* |  |
| N |  | *TRIDC1BG055610.1* |  |
| N |  | *TRIDC1BG055620.1* |  |
| N |  | *TRIDC1BG055630.1* |  |
| N |  | *TRIDC1BG055640.1* |  |
| N |  | *TRIDC1BG055650.1* |  |
| N |  | *TRIDC1BG055670.1* |  |
| N |  | *TRIDC1BG055680.1* |  |
| N |  | *TRIDC1BG055700.1* |  |
| N |  | *TRIDC1BG055710.1* |  |
| N |  | *TRIDC1BG055720.1* |  |
| N |  | *TRIDC1BG055730.1* |  |
| N |  | *TRIDC1BG055740.1* |  |
| N |  | *TRIDC1BG055750.1* |  |
| N |  | *TRIDC1BG055770.1* |  |
| N |  | *TRIDC1BG055780.1* |  |
| N |  | *TRIDC1BG055790.1* |  |
| N |  | *TRIDC1BG055800.1* |  |
| N |  | *TRIDC1BG055810.1* |  |
| N |  | *TRIDC1BG055820.1* |  |
| N |  | *TRIDC1BG055830.1* |  |
| N |  | *TRIDC1BG055840.1* |  |
| N |  | *TRIDC1BG055850.1* |  |
| N |  | *TRIDC1BG055860.1* |  |
| N |  | *TRIDC1BG055880.1* |  |
| N |  | *TRIDC1BG055890.1* |  |
| N |  | *TRIDC1BG055910.1* |  |
| N |  | *TRIDC1BG055920.1* |  |
| N |  | *TRIDC1BG055930.1* |  |
| N |  | *TRIDC1BG055950.1* |  |
| N |  | *TRIDC1BG055960.1* |  |
| N |  | *TRIDC1BG055970.1* |  |
| N |  | *TRIDC1BG055980.1* |  |
| N |  | *TRIDC1BG055990.1* |  |
| N |  | *TRIDC1BG056000.1* |  |
| N |  | *TRIDC1BG056010.1* |  |
| N |  | *TRIDC1BG056020.1* |  |
| N |  | *TRIDC1BG056030.1* |  |
| N |  | *TRIDC1BG056050.1* |  |
| N |  | *TRIDC1BG056060.1* |  |
| N |  | *TRIDC1BG056080.1* |  |
| N |  | *TRIDC1BG056110.1* |  |
| N |  | *TRIDC1BG056120.1* |  |
| N |  | *TRIDC1BG056130.1* |  |
| N |  | *TRIDC1BG056140.1* |  |
| N |  | *TRIDC1BG056150.1* |  |
| N |  | *TRIDC1BG056160.1* |  |
| N |  | *TRIDC1BG056170.1* |  |
| N |  | *TRIDC1BG056180.1* |  |
| N |  | *TRIDC1BG056200.1* |  |
| N |  | *TRIDC1BG056220.1* |  |
| N |  | *TRIDC1BG056230.1* |  |
| N |  | *TRIDC1BG056250.1* |  |
| N |  | *TRIDC1BG056260.1* |  |
| N |  | *TRIDC1BG056270.1* |  |
| N |  | *TRIDC1BG056280.1* |  |
| N |  | *TRIDC1BG056290.1* |  |
| N |  | *TRIDC1BG056300.1* |  |
| N |  | *TRIDC1BG056310.1* |  |
| N |  | *TRIDC1BG056330.1* |  |
| N |  | *TRIDC1BG056340.1* |  |
| N |  | *TRIDC1BG056350.1* |  |
| N |  | *TRIDC1BG056360.1* |  |
| N |  | *TRIDC1BG056390.1* |  |
| N |  | *TRIDC1BG056400.1* |  |
| N |  | *TRIDC1BG056410.1* |  |
| N |  | *TRIDC1BG056430.1* |  |
| N |  | *TRIDC1BG056440.1* |  |
| N |  | *TRIDC1BG056450.1* |  |
| N |  | *TRIDC1BG056470.1* |  |
| N |  | *TRIDC1BG056480.1* |  |
| N |  | *TRIDC1BG056490.1* |  |
| N |  | *TRIDC1BG056500.1* |  |
| N |  | *TRIDC1BG056510.1* |  |
| N |  | *TRIDC1BG056530.1* |  |
| N |  | *TRIDC1BG056520.1* |  |
| N |  | *TRIDC1BG056550.1* |  |
| N |  | *TRIDC1BG056560.1* |  |
| N |  | *TRIDC1BG056570.1* |  |
| N |  | *TRIDC1BG056580.1* |  |
| N |  | *TRIDC1BG056590.1* |  |
| N |  | *TRIDC1BG056600.1* |  |
| N |  | *TRIDC1BG056610.1* |  |
| N |  | *TRIDC1BG056620.1* |  |
| N |  | *TRIDC1BG056630.1* |  |
| N |  | *TRIDC1BG056640.1* |  |
| N |  | *TRIDC1BG056650.1* |  |
| N |  | *TRIDC1BG056660.1* |  |
| N |  | *TRIDC1BG056670.1* |  |
| N |  | *TRIDC1BG056690.1* |  |
| N |  | *TRIDC1BG056680.1* |  |
| N |  | *TRIDC1BG056710.1* |  |
| N |  | *TRIDC1BG056720.1* |  |
| N |  | *TRIDC1BG056760.1* |  |
| N |  | *TRIDC1BG056770.1* |  |
| N |  | *TRIDC1BG056810.1* |  |
| N |  | *TRIDC1BG056820.1* |  |
| N |  | *TRIDC1BG056840.1* |  |
| N |  | *TRIDC1BG056870.1* |  |
| N |  | *TRIDC1BG056880.1* |  |
| N |  | *TRIDC1BG056900.1* |  |
| N |  | *TRIDC1BG056930.1* |  |
| N |  | *TRIDC1BG056940.1* |  |
| N |  | *TRIDC1BG056950.1* |  |
| N |  | *TRIDC1BG056960.1* |  |
| N |  | *TRIDC1BG056970.1* |  |
| N |  | *TRIDC1BG056980.1* |  |
| N |  | *TRIDC1BG056990.1* |  |
| N |  | *TRIDC1BG057000.1* |  |
| N |  | *TRIDC1BG057020.1* |  |
| N |  | *TRIDC1BG057040.1* |  |
| N |  | *TRIDC1BG057050.1* |  |
| N |  | *TRIDC1BG057060.1* |  |
| N |  | *TRIDC1BG057080.1* |  |
| N |  | *TRIDC1BG057100.1* |  |
| N |  | *TRIDC1BG057110.1* |  |
| N |  | *TRIDC1BG057120.1* |  |
| N |  | *TRIDC1BG057130.1* |  |
| N |  | *TRIDC1BG057140.1* |  |
| N |  | *TRIDC1BG057150.1* |  |
| N |  | *TRIDC1BG057160.1* |  |
| N |  | *TRIDC1BG055810.1* |  |

Note: N represents no orthologs were identified after reciprocal blasting analysis.

**References**

[1] Goubet, F. AtCSLA7, a cellulose synthase-like putative glycosyltransferase, is important for pollen tube growth and embryogenesis in Arabidopsis. Plant Physiology, 2003, 131(2):547-557.

[2] Wang A , Garcia D , Zhang H , et al. The VQ motif protein IKU1 regulates endosperm growth and seed size in Arabidopsis. Plant Journal, 2010, 63(4):670-679.

[3] Grimault, Aurélie, Gendrot G , Chaignon S , et al. Role of B3 domain transcription factors of the AFL family in maize kernel filling. Plant ence, 2015, 236:116-125.

[4] Chen J , Gao H , Zheng X M , et al. An evolutionarily conserved gene, FUWA, plays a role in determining panicle architecture, grain shape and grain weight in rice. Plant Journal, 2015, 83(3):427-438.

[5] Ma L , Li T , Hao C , et al. TaGS5‐3A, a grain size gene selected during wheat improvement for larger kernel and yield. Plant Biotechnology Journal, 2016, 14(5):1269-1280.

[6] Jain M, Nijhawan A, Arora R, et al. F-box proteins in rice. Genome-wide analysis, classification, temporal and spatial gene expression during panicle and seed development, and regulation by light and abiotic stress. Plant Physiology, 2007, 143(4): 1467-1483.

[7] Zhang Y , Yang S , Song Y , et al. Genome-wide characterization, expression and functional analysis of CLV3/ESR gene family in tomato[J]. Bmc Genomics, 2014, 15(1):827.

[8] Yang X , Luo Z , Liang W , et al. Rice CYP703A3, a cytochrome P450 hydroxylase, is essential for development of anther cuticle and pollen exine. Journal of Integrative Plant Biology, 2015, 56(010):979-994.

[9] Hu Y , Zhao L , Chong K , et al. Overexpression of OsERF1, a novel rice ERF gene, up-regulates ethylene-responsive genes expression besides affects growth and development in Arabidopsis. Journal of Plant Physiology, 2008, 165(16):1717-1725.

[10] Verónica Lira-Ruan, Ruiz-Kubli M , Raúl Arredondo-Peter. Expression of non-symbiotic hemoglobin 1 and 2 genes in rice (Oryza sativa) embryonic organs. Communicative & Integrative Biology, 2011, 4(4):457-8.

[11] Kim B G , Lee Y , Hur H G , et al. Flavonoid 3'-O-methyltransferase from rice: cDNA cloning, characterization and functional expression. Phytochemistry, 2006, 67(4):387-394.

[12] Rausch C , Daram P , Brunner S , et al. A phosphate transporter expressed in arbuscule-containing cells in potato. Nature, 2001, 414(6862):462-470.

[13] Zhang G Y , Wu J , Wang X W . Cloning and expression analysis of a pollen preferential rapid alkalinization factor gene, BoRALF1, from broccoli flowers. Molecular Biology Reports, 2010, 37(7):3273-3281.

[14] Vandepeute J , Alvarez H R . Cyclic nucleotide phosphodiesterase activity in barley seeds[J]. Plant Physiology, 1973, 52(3):278-282.

[15] JoséMiguel, Ferreras, and, et al. Distribution and properties of major ribosome-inactivating proteins (28 S rRNA N-glycosidases) of the plant Saponaria officinalis L. (Caryophyllaceae). Biochimica Et Biophysica Acta Gene Structure & Expression, 1993.

[16] Fischer-Kilbienski I , Miao Y , Roitsch T , et al. Nuclear targeted AtS40 modulates senescence associated gene expression in Arabidopsis thaliana during natural development and in darkness. Plant Molecular Biology, 2010, 73(s4-5):379-390.

[17] Zhang F , Luo X , Hu B , et al. YGL138(t), encoding a putative signal recognition particle 54 kDa protein, is involved in chloroplast development of rice. Rice, 2013, 6(1):7.

[18] Chi Y H , Moon J C , Park J H , et al. Abnormal chloroplast development and growth inhibition in rice thioredoxin m knock-down plants.[J]. Plant Physiology, 2008, 148(2):808-817.

[19] Jang, I.-C. Expression of a Bifunctional Fusion of the Escherichia coli Genes for Trehalose-6-Phosphate Synthase and Trehalose-6-Phosphate Phosphatase in Transgenic Rice Plants Increases Trehalose Accumulation and Abiotic Stress Tolerance without Stunting Growth. Plant Physiology, 2003, 131(2):516.

[20] Yang Z , Zhou Y , Wang X , et al. Genomewide comparative phylogenetic and molecular evolutionary analysis of tubby-like protein family in Arabidopsis, rice, and poplar. Genomics, 2008, 92( 4):246-253.

[21] El-Ahmady S , Nessler C . Cellular localization of tyrosine decarboxylase expression in transgenic opium poppy and tobacco. Plant Cell Reports, 2001, 20(4):313-317.

**Table S9.2** Predicted genes in the interval of the major QTL *QKW.sicau-2SY-6D*, *QLWR.sicau-2SY-6D* and *QKS.sicau-2SY-6D*

| **Gene ID in CS** | **Annotation** | **Orthologs in Aegilops tauschii** | **References** |
| --- | --- | --- | --- |
| *TraesCS6D01G081900.1* | **BURP domain protein RD22** | *AET6Gv20259300.1* | [1] |
| *TraesCS6D01G082000.1* | **BURP domain protein RD22** | *AET6Gv20258800.1* | [1] |
| *TraesCS6D01G088500.1* | **cyclin-dependent kinase inhibitor** | *AET6Gv20246600.1* | [2] |
| *TraesCS6D01G088200.1* | **F-box family protein** | *AET6Gv20247500.1* | [3] |
| *TraesCS6D01G090100.1* | **F-box family protein** | *AET6Gv20266200.1* | [3] |
| *TraesCS6D01G090300.1* | **F-box family protein** | *AET6Gv20266600.1* | [3] |
| *TraesCS6D01G091400.1* | **F-box family protein** | *AET6Gv20269000.1* | [3] |
| *TraesCS6D01G093500.1* | **F-box family protein** | *AET6Gv20273300.1* | [3] |
| *TraesCS6D01G093600.1* | **F-box family protein** | *AET6Gv20274100.1* | [3] |
| *TraesCS6D01G097200.1* | **F-box family protein** | *AET6Gv20282700.1* | [3] |
| *TraesCS6D01G107300.1* | **F-box family protein** | *AET6Gv20303900.1* | [3] |
| *TraesCS6D01G080600.1* | **F-box domain containing protein** | *AET6Gv20240000.1* | [4] |
| *TraesCS6D01G080900.1* | **F-box domain containing protein** | *AET6Gv20240800.1* | [4] |
| *TraesCS6D01G091500.1* | **F-box domain containing protein** | *AET6Gv20269100.1* | [4] |
| *TraesCS6D01G092900.1* | **F-box domain containing protein** | *AET6Gv20272000.1* | [4] |
| *TraesCS6D01G093100.1* | **F-box domain containing protein** | *AET6Gv20272500.1* | [4] |
| *TraesCS6D01G102900.1* | **F-box domain containing protein** | *AET6Gv20292600.1* | [4] |
| *TraesCS6D01G103200.1* | **F-box domain containing protein** | *AET6Gv20294200.1* | [4] |
| *TraesCS6D01G090700.1* | **Glycosyltransferase** | *AET6Gv20267600.1* | [5] |
| *TraesCS6D01G094700.1* | **Glycosyltransferase** | *AET6Gv20276900.1* | [5] |
| *TraesCS6D01G105400.1* | **Glycosyltransferase** | *AET6Gv20299500.1* | [5] |
| *TraesCS6D01G104900.1* | FBD-associated F-box protein | *AET6Gv20297100.1* | [6] |
| *TraesCS6D01G107200.1* | FBD-associated F-box protein | *AET6Gv20303500.1* | [6] |
| *TraesCS6D01G100800.1* | Auxin response factor | *AET6Gv20287800.1* | [7] |
| *TraesCS6D01G102300.1* | Auxin response factor | *AET6Gv20290900.1* | [7] |
| *TraesCS6D01G088600.1* | Chlorophyll a-b binding protein, chloroplastic | *AET6Gv20246500.1* | [8] |
| *TraesCS6D01G088800.1* | Chlorophyll a-b binding protein, chloroplastic | *AET6Gv20245800.1* | [8] |
| *TraesCS6D01G095000.1* | Splicing factor 3B subunit-like protein | *AET6Gv20277400.1* | [9] |
| *TraesCS6D01G104700.1* | Splicing factor 3B subunit 1 | *AET6Gv20296700.1* | [9] |
| *TraesCS6D01G086200.1* | Sequence-specific DNA binding transcription factor | *AET6Gv20251000.1* | [10] |
| *TraesCS6D01G086500.1* | Sequence-specific DNA binding transcription factor | *AET6Gv20250300.1* | [10] |
| *TraesCS6D01G087900.1* | Sequence-specific DNA binding transcription factor | *AET6Gv20248200.1* | [10] |
| *TraesCS6D01G104400.1* | SAUR-like auxin-responsive protein family, putative | *AET6Gv20296300.1* | [11] |
| *TraesCS6D01G104500.1* | SAUR-like auxin-responsive protein family, putative | *AET6Gv20296400.1* | [11] |
| *TraesCS6D01G086300.1* | RING/U-box superfamily protein | *AET6Gv20250900.1* | [12] |
| *TraesCS6D01G087000.1* | RING/U-box superfamily protein | *AET6Gv20249200.1* | [12] |
| *TraesCS6D01G086600.1* | P-loop containing nucleoside triphosphate hydrolases superfamily protein | *AET6Gv20250000.1* | [13] |
| *TraesCS6D01G088400.1* | P-loop containing nucleoside triphosphate hydrolases superfamily protein | *AET6Gv20246700.1* | [13] |
| *TraesCS6D01G083100.1* | Pentatricopeptide repeat-containing protein | *AET6Gv20256200.1* | [14] |
| *TraesCS6D01G083400.1* | Pentatricopeptide repeat-containing protein | *AET6Gv20255400.1* | [14] |
| *TraesCS6D01G101000.1* | Mitochondrial import inner membrane translocase subunit TIM22 | *AET6Gv20288100.1* | [15] |
| *TraesCS6D01G096100.1* | Mitochondrial import inner membrane translocase subunit tim-10 isoform 1 | *AET6Gv20280800.1* | [15] |
| *TraesCS6D01G082700.1* | Lipid transfer protein | *AET6Gv20257800.1* | [16] |
| *TraesCS6D01G084600.1* | Lipid transfer protein | *AET6Gv20253700.1* | [16] |
| *TraesCS6D01G081500.1* | Leucine-rich repeat receptor-like protein kinase family protein | *AET6Gv20262600.1* | [17] |
| *TraesCS6D01G096600.1* | Leucine-rich repeat receptor-like protein kinase family protein | *AET6Gv20281900.1* | [17] |
| *TraesCS6D01G106800.1* | Histone H2A | *AET6Gv20302800.1* | [18] |
| *TraesCS6D01G107000.1* | Histone H2A | *AET6Gv20303400.1* | [18] |
| *TraesCS6D01G098400.1* | Glucan endo-1,3-beta-glucosidase-like protein | *AET6Gv20284600.1* | [19] |
| *TraesCS6D01G099100.1* | Glucan endo-1,3-beta-glucosidase 3 | *AET6Gv20285400.1* | [19] |
| *TraesCS6D01G085800.1* | F-box/LRR-repeat protein | *AET6Gv20252000.1* | [20] |
| *TraesCS6D01G087800.1* | F-box/LRR-repeat protein | *AET6Gv20248300.1* | [20] |
| *TraesCS6D01G084200.1* | Ethylene-responsive transcription factor | *AET6Gv20254600.1* | [21] |
| *TraesCS6D01G084900.1* | Ethylene-responsive transcription factor | *AET6Gv20253100.1* | [21] |
| *TraesCS6D01G085000.1* | Ethylene-responsive transcription factor | *AET6Gv20252800.1* | [21] |
| *TraesCS6D01G092800.1* | Disease resistance protein (NBS-LRR class) family | *AET6Gv20271800.1* | [22] |
| *TraesCS6D01G105100.1* | Disease resistance protein (NBS-LRR class) family | *AET6Gv20297700.1* | [22] |
| *TraesCS6D01G085100.1* | Cytochrome P450 family protein, expressed | *AET6Gv20252600.1* | [23] |
| *TraesCS6D01G103300.1* | Cytochrome P450 family protein, expressed | *AET6Gv20294400.1* | [23] |
| *TraesCS6D01G098600.1* | Terpene cyclase/mutase family member | *AET6Gv20284800.1* | [24] |
| *TraesCS6D01G100000.1* | Terpene cyclase/mutase family member | *AET6Gv20286800.1* | [24] |
| *TraesCS6D01G106100.1* | Harpin-induced protein | *AET6Gv20301600.1* | [25] |
| *TraesCS6D01G106200.1* | Harpin-induced protein | *AET6Gv20301700.1* | [25] |
| *TraesCS6D01G080300.1* | Late embryogenesis abundant protein | *AET6Gv20239000.1* | [26] |
| *TraesCS6D01G082600.1* | Zinc transporter, putative | *AET6Gv20257900.1* | [27] |
| *TraesCS6D01G103100.1* | Zinc finger protein VAR3, chloroplastic | *AET6Gv20292700.1* | [28] |
| *TraesCS6D01G108000.1* | Zinc finger protein CONSTANS-LIKE 2 | *AET6Gv20305800.1* | [29] |
| *TraesCS6D01G103600.1* | Zinc finger BED domain-containing protein DAYSLEEPER | *AET6Gv20294800.1* | [30] |
| *TraesCS6D01G080400.1* | UBX domain-containing protein 1 | *AET6Gv20239100.1* | [31] |
| *TraesCS6D01G104300.1* | U3 small nucleolar RNA-associated protein 18-like protein | *AET6Gv20295600.1* | [32] |
| *TraesCS6D01G092700.1* | transmembrane protein, putative (DUF1218) | *AET6Gv20271600.1* | [33] |
| *TraesCS6D01G084700.1* | Transmembrane protein 53 | *AET6Gv20253300.1* | [34] |
| *TraesCS6D01G105900.1* | transmembrane protein | *AET6Gv20301300.1* | [35] |
| *TraesCS6D01G093700.1* | Transducin family protein / WD-40 repeat family protein | *AET6Gv20274200.1* | [36] |
| *TraesCS6D01G097100.1* | Transcription factor GTE8 | *AET6Gv20282400.1* | [37] |
| *TraesCS6D01G083200.1* | Succinate dehydrogenase [ubiquinone] flavoprotein subunit, mitochondrial | *AET6Gv20255600.1* | [38] |
| *TraesCS6D01G101200.1* | Subtilisin-like protease | *AET6Gv20288200.1* | [39] |
| *TraesCS6D01G098500.1* | Squamosa promoter-binding-like protein | *AET6Gv20284700.1* | [40] |
| *TraesCS6D01G086400.1* | Spindle pole body component SPC42 | *AET6Gv20250600.1* | [41] |
| *TraesCS6D01G088300.1* | Sphingoid base hydroxylase 2 | *AET6Gv20246900.1* | [42] |
| *TraesCS6D01G108200.1* | Sn1-specific diacylglycerol lipase alpha | *AET6Gv20306300.1* | [43] |
| *TraesCS6D01G089600.1* | Signal recognition particle protein | *AET6Gv20265000.1* | [44] |
| *TraesCS6D01G103700.1* | Serine/threonine-protein kinase TAO1 | *AET6Gv20294900.1* | [45] |
| *TraesCS6D01G081100.1* | Serine/threonine-protein kinase ATM | *AET6Gv20241400.1* | [46] |
| *TraesCS6D01G085200.1* | Sec14p-like phosphatidylinositol transfer family protein | *AET6Gv20252400.1* | [47] |
| *TraesCS6D01G086800.1* | RNA binding protein, putative | *AET6Gv20249500.1* | [48] |
| *TraesCS6D01G096800.1* | RNA binding protein | *AET6Gv20282000.1* | [49] |
| *TraesCS6D01G095500.1* | Ribosomal RNA small subunit methyltransferase H | *AET6Gv20278100.1* | [50] |
| *TraesCS6D01G101400.1* | Ribosomal RNA apurinic site specific lyase | *AET6Gv20288500.1* | [51] |
| *TraesCS6D01G092500.1* | Ribosomal protein S4 | *AET6Gv20271200.1* | [52] |
| *TraesCS6D01G096200.1* | Rhamnogalacturonate lyase | *AET6Gv20281100.1* | [53] |
| *TraesCS6D01G085400.1* | Protoheme IX farnesyltransferase | *AET6Gv20252100.1* | [54] |
| *TraesCS6D01G105700.1* | Protein STIP1-like protein | *AET6Gv20301000.1* | N |
| *TraesCS6D01G099700.1* | Protein phosphatase 2c, putative | *AET6Gv20286200.1* | [55] |
| *TraesCS6D01G097500.1* | Protein arginine N-methyltransferase | *AET6Gv20283300.1* | [56] |
| *TraesCS6D01G102200.1* | Protein AIG2 | *AET6Gv20290800.1* | [57] |
| *TraesCS6D01G084800.1* | Proteasome subunit alpha type | *AET6Gv20253200.1* | [58] |
| *TraesCS6D01G092200.1* | Proline-rich protein | *AET6Gv20270900.1* | [59] |
| *TraesCS6D01G105800.1* | Polynucleotide 5'-hydroxyl-kinase NOL9 | *AET6Gv20301200.1* | [60] |
| *TraesCS6D01G094800.1* | Pleiotropic drug resistance ABC transporter | *AET6Gv20277300.1* | [61] |
| *TraesCS6D01G091000.1* | Plant regulator RWP-RK family protein | *AET6Gv20268100.1* | [62] |
| *TraesCS6D01G093800.1* | Plant protein 1589 of unknown function | *AET6Gv20274400.1* | N |
| *TraesCS6D01G095600.1* | Photosystem I assembly protein Ycf3 | *AET6Gv20279400.1* | [63] |
| *TraesCS6D01G084500.1* | Phosphopantetheine adenylyltransferase | *AET6Gv20254200.1* | [64] |
| *TraesCS6D01G108400.1* | Peroxidase | *AET6Gv20306700.1* | [65] |
| *TraesCS6D01G087600.1* | Pentatricopeptide repeat-containing protein | *AET6Gv20248600.1* | [66] |
| *TraesCS6D01G104100.1* | ORMDL family protein-like | *AET6Gv20295300.1* | [67] |
| *TraesCS6D01G089800.1* | Oligopeptide transporter | *AET6Gv20265800.1* | [68] |
| *TraesCS6D01G081400.1* | Nucleotide-sugar transporter family protein | *AET6Gv20241600.1* | [69] |
| *TraesCS6D01G102600.1* | Nuclear pore complex protein Nup160 | *AET6Gv20291700.1* | [70] |
| *TraesCS6D01G089100.1* | Nucellin-like aspartic protease | *AET6Gv20244800.1* | [71] |
| *TraesCS6D01G094600.1* | Nodulin-like / Major Facilitator Superfamily protein | *AET6Gv20276600.1* | [72] |
| *TraesCS6D01G100300.1* | NBS-LRR-like resistance protein | *AET6Gv20287100.1* | [73] |
| *TraesCS6D01G084400.1* | NAD-dependent glyceraldehyde-3-phosphate dehydrogenase | *AET6Gv20254300.1* | [74] |
| *TraesCS6D01G096300.1* | NAC domain-containing protein, putative | *AET6Gv20281500.1* | [75] |
| *TraesCS6D01G098200.1* | Myb family transcription factor-like | *AET6Gv20284300.1* | [76] |
| *TraesCS6D01G108100.1* | Mitogen-activated protein kinase | *AET6Gv20306100.1* | [77] |
| *TraesCS6D01G081200.1* | MALE GAMETOPHYTE DEFECTIVE 1 | *AET6Gv20241500.1* | [78] |
| *TraesCS6D01G097900.1* | low-molecular-weight cysteine-rich 48 | *AET6Gv20283700.1* | [79] |
| *TraesCS6D01G096000.1* | Leucine-rich repeat protein kinase family protein | *AET6Gv20279900.1* | [80] |
| *TraesCS6D01G083900.1* | Kinetochore protein nuf2, putative | *AET6Gv20254800.1* | [81] |
| *TraesCS6D01G091600.1* | Kinesin-like protein | *AET6Gv20269300.1* | [82] |
| *TraesCS6D01G082800.1* | Histone H3 | *AET6Gv20257700.1* | [83] |
| *TraesCS6D01G095400.1* | Histone acetyltransferase | *AET6Gv20278000.1* | [84] |
| *TraesCS6D01G083800.1* | Heat shock transcription factor | *AET6Gv20255000.1* | [85] |
| *TraesCS6D01G095700.1* | guanine nucleotide-binding protein subunit gamma | *AET6Gv20279500.1* | [86] |
| *TraesCS6D01G089700.1* | GRF zinc finger family protein, expressed | *AET6Gv20270800.1* | [87] |
| *TraesCS6D01G091800.1* | Glycine rich protein | *AET6Gv20269600.1* | [88] |
| *TraesCS6D01G090500.1* | Geranylgeranyl pyrophosphate synthase | *AET6Gv20266800.1* | [89] |
| *TraesCS6D01G093000.1* | GDSL esterase/lipase | *AET6Gv20272100.1* | [90] |
| *TraesCS6D01G100700.1* | Gamma-tubulin complex component | *AET6Gv20287500.1* | [91] |
| *TraesCS6D01G102400.1* | Galactokinase | *AET6Gv20291200.1* | [92] |
| *TraesCS6D01G099200.1* | F-box plant-like protein, putative | *AET6Gv20285600.1* | [93] |
| *TraesCS6D01G100200.1* | Fatty acid desaturase 4, chloroplastic | *AET6Gv20287000.1* | [94] |
| *TraesCS6D01G103500.1* | Fasciclin-like arabinogalactan protein | *AET6Gv20294700.1* | [95] |
| *TraesCS6D01G102100.1* | Eukaryotic translation initiation factor | *AET6Gv20290500.1* | [96] |
| *TraesCS6D01G082300.1* | Esterase/lipase/thioesterase-like protein | *AET6Gv20258200.1* | [97] |
| *TraesCS6D01G089000.1* | Endoglucanase 11 | *AET6Gv20245000.1* | [98] |
| *TraesCS6D01G089200.1* | Endoglucanase | *AET6Gv20244600.1* | [99] |
| *TraesCS6D01G108300.1* | E3 ubiquitin-protein ligase SINA-like 10 | *AET6Gv20306400.1* | [100] |
| *TraesCS6D01G087300.1* | E3 ubiquitin-protein ligase MARCH8 | *AET6Gv20248900.1* | [101] |
| *TraesCS6D01G083500.1* | DUF1645 family protein | *AET6Gv20255300.1* | [102] |
| *TraesCS6D01G103900.1* | DTW domain containing protein, expressed | *AET6Gv20295100.1* | N |
| *TraesCS6D01G104800.1* | Dolichol kinase | *AET6Gv20296900.1* | [103] |
| *TraesCS6D01G106700.1* | DNA-binding protein BIN4 | *AET6Gv20302700.1* | [104] |
| *TraesCS6D01G107900.1* | DNA/RNA helicase protein | *AET6Gv20305100.1* | [105] |
| *TraesCS6D01G107800.1* | DNA repair helicase ERCC6-like | *AET6Gv20304400.1* | [106] |
| *TraesCS6D01G089900.1* | DNA polymerase V | *AET6Gv20265900.1* | [107] |
| *TraesCS6D01G106000.1* | DNA mismatch repair protein MutS | *AET6Gv20301400.1* | [108] |
| *TraesCS6D01G106600.1* | disease resistance protein (TIR-NBS-LRR class) | *AET6Gv20302200.1* | [109] |
| *TraesCS6D01G081600.1* | Disease resistance family protein | *AET6Gv20260900.1* | [110] |
| *TraesCS6D01G098000.1* | Dirigent protein | *AET6Gv20284000.1* | [111] |
| *TraesCS6D01G095800.1* | Delta-aminolevulinic acid dehydratase | *AET6Gv20279600.1* | [112] |
| *TraesCS6D01G094000.1* | cytomegalovirus UL139 protein | *AET6Gv20275200.1* | [113] |
| *TraesCS6D01G080800.1* | Cytochrome P450 family protein | *AET6Gv20240500.1* | [114] |
| *TraesCS6D01G090800.1* | CTC-interacting domain 7 | *AET6Gv20267800.1* | [115] |
| *TraesCS6D01G101300.1* | Cortactin-binding protein 2 | *AET6Gv20288400.1* | [116] |
| *TraesCS6D01G102700.1* | Chorismate synthase | *AET6Gv20291900.1* | [117] |
| *TraesCS6D01G084300.1* | Chloride channel protein | *AET6Gv20254500.1* | [118] |
| *TraesCS6D01G106500.1* | Chitinase-like protein 2 | *AET6Gv20302300.1* | [119] |
| *TraesCS6D01G094300.1* | Cellulose synthase-like protein | *AET6Gv20276100.1* | [120] |
| *TraesCS6D01G088000.1* | Cation-transporting ATPase 4 | *AET6Gv20247700.1* | [121] |
| *TraesCS6D01G083000.1* | CASP-like protein | *AET6Gv20256600.1* | [122] |
| *TraesCS6D01G090200.1* | Calreticulin/calnexin | *AET6Gv20266400.1* | [123] |
| *TraesCS6D01G080500.1* | Calmodulin-binding protein-like | *AET6Gv20239300.1* | [124] |
| *TraesCS6D01G093400.1* | Calmodulin-binding family protein, putative, expressed | *AET6Gv20273500.1* | [125] |
| *TraesCS6D01G104000.1* | Calcium-dependent lipid-binding domain protein | *AET6Gv20295200.1* | [126] |
| *TraesCS6D01G102500.1* | CAAX amino terminal protease | *AET6Gv20291600.1* | [127] |
| *TraesCS6D01G087400.1* | BZIP transcription factor | *AET6Gv20248700.1* | [128] |
| *TraesCS6D01G094100.1* | BTB/POZ domain containing protein, expressed | *AET6Gv20275300.1* | [129] |
| *TraesCS6D01G087200.1* | BRCT domain-containing protein | *AET6Gv20249000.1* | [130] |
| *TraesCS6D01G102000.1* | B3 domain-containing protein | *AET6Gv20290200.1* | [131] |
| *TraesCS6D01G103400.1* | ATP-dependent RNA helicase DDX47 | *AET6Gv20294600.1* | [132] |
| *TraesCS6D01G105000.1* | ATP-dependent RNA helicase | *AET6Gv20297200.1* | [133] |
| *TraesCS6D01G090000.1* | AT5G11810-like protein | *AET6Gv20266100.1* |  |
| *TraesCS6D01G091100.1* | Aspartyl/glutamyl-tRNA(Asn/Gln) amidotransferase subunit B | *AET6Gv20268200.1* | [134] |
| *TraesCS6D01G101600.1* | AP-1 complex subunit sigma-like protein | *AET6Gv20289600.1* | [135] |
| *TraesCS6D01G089300.1* | Anthranilate phosphoribosyltransferase | *AET6Gv20244500.1* | [136] |
| *TraesCS6D01G094500.1* | Alpha-amylase/subtilisin inhibitor | *AET6Gv20276400.1* | [137] |
| *TraesCS6D01G082500.1* | Adenylate cyclase | *AET6Gv20258000.1* | [138] |
| *TraesCS6D01G090900.1* | 5'-AMP-activated protein kinase subunit beta-1 | *AET6Gv20268000.1* | [139] |
| *TraesCS6D01G094200.1* | 50S ribosomal protein L3 | *AET6Gv20275400.1* | [140] |
| *TraesCS6D01G086900.1* | 3-isopropylmalate dehydratase large subunit | *AET6Gv20249400.1* | [141] |
| *TraesCS6D01G107100.1* | 2-oxoglutarate (2OG) and Fe(II)-dependent oxygenase superfamily protein | *AET6Gv20303700.1* | [142] |
| *TraesCS6D01G105500.1* | 26S proteasome non-ATPase regulatory subunit-like protein | *AET6Gv20299700.1* | [143] |
| *TraesCS6D01G080700.1* |  | N |  |
| *TraesCS6D01G081000.1* |  | N |  |
| *TraesCS6D01G081300.1* |  | N |  |
| *TraesCS6D01G081700.1* |  | N |  |
| *TraesCS6D01G081800.1* |  | N |  |
| *TraesCS6D01G082100.1* |  | N |  |
| *TraesCS6D01G082200.1* |  | N |  |
| *TraesCS6D01G082400.1* |  | N |  |
| *TraesCS6D01G082900.1* |  | N |  |
| *TraesCS6D01G083300.1* |  | N |  |
| *TraesCS6D01G083600.1* |  | N |  |
| *TraesCS6D01G083700.1* |  | N |  |
| *TraesCS6D01G084000.1* |  | N |  |
| *TraesCS6D01G084100.1* |  | N |  |
| *TraesCS6D01G085300.1* |  | N |  |
| *TraesCS6D01G085500.1* |  | N |  |
| *TraesCS6D01G085600.1* |  | N |  |
| *TraesCS6D01G085700.1* |  | N |  |
| *TraesCS6D01G085900.1* |  | N |  |
| *TraesCS6D01G086000.1* |  | N |  |
| *TraesCS6D01G086100.1* |  | N |  |
| *TraesCS6D01G086700.1* |  | N |  |
| *TraesCS6D01G087100.1* |  | N |  |
| *TraesCS6D01G087500.1* |  | N |  |
| *TraesCS6D01G087700.1* |  | N |  |
| *TraesCS6D01G088100.1* |  | N |  |
| *TraesCS6D01G088700.1* |  | N |  |
| *TraesCS6D01G088900.1* |  | N |  |
| *TraesCS6D01G089400.1* |  | N |  |
| *TraesCS6D01G089500.1* |  | N |  |
| *TraesCS6D01G090400.1* |  | N |  |
| *TraesCS6D01G090600.1* |  | N |  |
| *TraesCS6D01G091300.1* |  | N |  |
| *TraesCS6D01G091700.1* |  | N |  |
| *TraesCS6D01G091900.1* |  | N |  |
| *TraesCS6D01G092000.1* |  | N |  |
| *TraesCS6D01G092100.1* |  | N |  |
| *TraesCS6D01G092300.1* |  | N |  |
| *TraesCS6D01G092400.1* |  | N |  |
| *TraesCS6D01G092600.1* |  | N |  |
| *TraesCS6D01G093200.1* |  | N |  |
| *TraesCS6D01G093300.1* |  | N |  |
| *TraesCS6D01G093900.1* |  | N |  |
| *TraesCS6D01G094400.1* |  | N |  |
| *TraesCS6D01G094900.1* |  | N |  |
| *TraesCS6D01G095100.1* |  | N |  |
| *TraesCS6D01G095200.1* |  | N |  |
| *TraesCS6D01G095300.1* |  | N |  |
| *TraesCS6D01G095900.1* |  | N |  |
| *TraesCS6D01G096400.1* |  | N |  |
| *TraesCS6D01G096500.1* |  | N |  |
| *TraesCS6D01G096700.1* |  | N |  |
| *TraesCS6D01G096900.1* |  | N |  |
| *TraesCS6D01G097000.1* |  | N |  |
| *TraesCS6D01G097300.1* |  | N |  |
| *TraesCS6D01G097400.1* |  | N |  |
| *TraesCS6D01G097600.1* |  | N |  |
| *TraesCS6D01G097700.1* |  | N |  |
| *TraesCS6D01G097800.1* |  | N |  |
| *TraesCS6D01G098100.1* |  | N |  |
| *TraesCS6D01G098300.1* |  | N |  |
| *TraesCS6D01G098700.1* |  | N |  |
| *TraesCS6D01G098800.1* |  | N |  |
| *TraesCS6D01G098900.1* |  | N |  |
| *TraesCS6D01G099000.1* |  | N |  |
| *TraesCS6D01G099300.1* |  | N |  |
| *TraesCS6D01G099400.1* |  | N |  |
| *TraesCS6D01G099500.1* |  | N |  |
| *TraesCS6D01G099600.1* |  | N |  |
| *TraesCS6D01G099800.1* |  | N |  |
| *TraesCS6D01G099900.1* |  | N |  |
| *TraesCS6D01G100100.1* |  | N |  |
| *TraesCS6D01G100400.1* |  | N |  |
| *TraesCS6D01G100500.1* |  | N |  |
| *TraesCS6D01G100600.1* |  | N |  |
| *TraesCS6D01G100900.1* |  | N |  |
| *TraesCS6D01G101100.1* |  | N |  |
| *TraesCS6D01G101500.1* |  | N |  |
| *TraesCS6D01G101700.1* |  | N |  |
| *TraesCS6D01G101800.1* |  | N |  |
| *TraesCS6D01G101900.1* |  | N |  |
| *TraesCS6D01G102800.1* |  | N |  |
| *TraesCS6D01G103000.1* |  | N |  |
| *TraesCS6D01G103800.1* |  | N |  |
| *TraesCS6D01G104200.1* |  | N |  |
| *TraesCS6D01G104600.1* |  | N |  |
| *TraesCS6D01G105200.1* |  | N |  |
| *TraesCS6D01G105300.1* |  | N |  |
| *TraesCS6D01G105600.1* |  | N |  |
| *TraesCS6D01G106300.1* |  | N |  |
| *TraesCS6D01G106400.1* |  | N |  |
| *TraesCS6D01G106900.1* |  | N |  |
| *TraesCS6D01G107400.1* |  | N |  |
| *TraesCS6D01G107500.1* |  | N |  |
| *TraesCS6D01G107600.1* |  | N |  |
| *TraesCS6D01G107700.1* |  | N |  |
| *TraesCS6D01G108500.1* |  | N |  |
| *TraesCS6D01G108600.1* |  | N |  |
| N |  | *AET6Gv20240100.1* |  |
| N |  | *AET6Gv20240200.1* |  |
| N |  | *AET6Gv20240700.1* |  |
| N |  | *AET6Gv20242400.1* |  |
| N |  | *AET6Gv20242600.1* |  |
| N |  | *AET6Gv20242800.1* |  |
| N |  | *AET6Gv20243500.1* |  |
| N |  | *AET6Gv20243700.1* |  |
| N |  | *AET6Gv20243900.1* |  |
| N |  | *AET6Gv20244300.1* |  |
| N |  | *AET6Gv20244700.1* |  |
| N |  | *AET6Gv20245900.1* |  |
| N |  | *AET6Gv20246000.1* |  |
| N |  | *AET6Gv20246300.1* |  |
| N |  | *AET6Gv20246800.1* |  |
| N |  | *AET6Gv20248500.1* |  |
| N |  | *AET6Gv20251200.1* |  |
| N |  | *AET6Gv20251300.1* |  |
| N |  | *AET6Gv20251900.1* |  |
| N |  | *AET6Gv20252300.1* |  |
| N |  | *AET6Gv20252500.1* |  |
| N |  | *AET6Gv20253400.1* |  |
| N |  | *AET6Gv20253800.1* |  |
| N |  | *AET6Gv20254700.1* |  |
| N |  | *AET6Gv20255100.1* |  |
| N |  | *AET6Gv20255800.1* |  |
| N |  | *AET6Gv20255900.1* |  |
| N |  | *AET6Gv20256900.1* |  |
| N |  | *AET6Gv20258300.1* |  |
| N |  | *AET6Gv20258500.1* |  |
| N |  | *AET6Gv20258600.1* |  |
| N |  | *AET6Gv20258900.1* |  |
| N |  | *AET6Gv20259100.1* |  |
| N |  | *AET6Gv20259600.1* |  |
| N |  | *AET6Gv20259700.1* |  |
| N |  | *AET6Gv20259900.1* |  |
| N |  | *AET6Gv20260400.1* |  |
| N |  | *AET6Gv20260500.1* |  |
| N |  | *AET6Gv20262700.1* |  |
| N |  | *AET6Gv20263500.1* |  |
| N |  | *AET6Gv20263600.1* |  |
| N |  | *AET6Gv20263900.1* |  |
| N |  | *AET6Gv20264400.1* |  |
| N |  | *AET6Gv20264600.1* |  |
| N |  | *AET6Gv20264900.1* |  |
| N |  | *AET6Gv20265600.1* |  |
| N |  | *AET6Gv20266300.1* |  |
| N |  | *AET6Gv20268500.1* |  |
| N |  | *AET6Gv20268600.1* |  |
| N |  | *AET6Gv20268800.1* |  |
| N |  | *AET6Gv20268900.1* |  |
| N |  | *AET6Gv20269700.1* |  |
| N |  | *AET6Gv20269900.1* |  |
| N |  | *AET6Gv20270400.1* |  |
| N |  | *AET6Gv20270600.1* |  |
| N |  | *AET6Gv20271000.1* |  |
| N |  | *AET6Gv20271100.1* |  |
| N |  | *AET6Gv20271300.1* |  |
| N |  | *AET6Gv20271400.1* |  |
| N |  | *AET6Gv20271500.1* |  |
| N |  | *AET6Gv20272200.1* |  |
| N |  | *AET6Gv20273800.1* |  |
| N |  | *AET6Gv20274700.1* |  |
| N |  | *AET6Gv20275000.1* |  |
| N |  | *AET6Gv20275600.1* |  |
| N |  | *AET6Gv20275700.1* |  |
| N |  | *AET6Gv20276200.1* |  |
| N |  | *AET6Gv20276500.1* |  |
| N |  | *AET6Gv20277500.1* |  |
| N |  | *AET6Gv20277800.1* |  |
| N |  | *AET6Gv20278300.1* |  |
| N |  | *AET6Gv20278500.1* |  |
| N |  | *AET6Gv20278700.1* |  |
| N |  | *AET6Gv20278800.1* |  |
| N |  | *AET6Gv20279000.1* |  |
| N |  | *AET6Gv20279100.1* |  |
| N |  | *AET6Gv20279200.1* |  |
| N |  | *AET6Gv20280300.1* |  |
| N |  | *AET6Gv20280400.1* |  |
| N |  | *AET6Gv20280600.1* |  |
| N |  | *AET6Gv20281000.1* |  |
| N |  | *AET6Gv20281700.1* |  |
| N |  | *AET6Gv20281800.1* |  |
| N |  | *AET6Gv20282100.1* |  |
| N |  | *AET6Gv20282200.1* |  |
| N |  | *AET6Gv20282800.1* |  |
| N |  | *AET6Gv20282900.1* |  |
| N |  | *AET6Gv20284200.1* |  |
| N |  | *AET6Gv20284400.1* |  |
| N |  | *AET6Gv20284500.1* |  |
| N |  | *AET6Gv20285200.1* |  |
| N |  | *AET6Gv20285800.1* |  |
| N |  | *AET6Gv20286000.1* |  |
| N |  | *AET6Gv20286500.1* |  |
| N |  | *AET6Gv20286600.1* |  |
| N |  | *AET6Gv20286700.1* |  |
| N |  | *AET6Gv20286900.1* |  |
| N |  | *AET6Gv20287200.1* |  |
| N |  | *AET6Gv20287900.1* |  |
| N |  | *AET6Gv20288300.1* |  |
| N |  | *AET6Gv20288600.1* |  |
| N |  | *AET6Gv20288700.1* |  |
| N |  | *AET6Gv20289100.1* |  |
| N |  | *AET6Gv20289400.1* |  |
| N |  | *AET6Gv20289500.1* |  |
| N |  | *AET6Gv20289700.1* |  |
| N |  | *AET6Gv20290400.1* |  |
| N |  | *AET6Gv20290600.1* |  |
| N |  | *AET6Gv20292300.1* |  |
| N |  | *AET6Gv20292900.1* |  |
| N |  | *AET6Gv20291400.1* |  |
| N |  | *AET6Gv20293000.1* |  |
| N |  | *AET6Gv20293200.1* |  |
| N |  | *AET6Gv20293400.1* |  |
| N |  | *AET6Gv20293900.1* |  |
| N |  | *AET6Gv20295000.1* |  |
| N |  | *AET6Gv20295800.1* |  |
| N |  | *AET6Gv20298700.1* |  |
| N |  | *AET6Gv20298800.1* |  |
| N |  | *AET6Gv20299000.1* |  |
| N |  | *AET6Gv20300100.1* |  |
| N |  | *AET6Gv20300500.1* |  |
| N |  | *AET6Gv20304000.1* |  |
| N |  | *AET6Gv20304100.1* |  |
| N |  | *AET6Gv20304300.1* |  |
| N |  | *AET6Gv20304500.1* |  |
| N |  | *AET6Gv20304700.1* |  |
| N |  | *AET6Gv20301800.1* |  |
| N |  | *AET6Gv20301900.1* |  |
| N |  | *AET6Gv20302400.1* |  |
| N |  | *AET6Gv20302900.1* |  |
| N |  | *AET6Gv20303200.1* |  |
| N |  | *AET6Gv20303300.1* |  |
| N |  | *AET6Gv20305600.1* |  |
| N |  | *AET6Gv20305700.1* |  |
| N |  | *AET6Gv20305900.1* |  |
| N |  | *AET6Gv20306000.1* |  |
| N |  | *AET6Gv20306900.1* |  |
| N |  | *AET6Gv20307000.1* |  |
| N |  | *AET6Gv20307100.1* |  |

Note: N represents no orthologs were identified after reciprocal blasting analysis.

**References**

[1] Tiedemann J, Rutten T, Hillmer S, et al. The BURP domain protein AtUSPL1 of Arabidopsis thaliana is destined to the protein storage vacuoles and overexpression of the cognate gene distorts seed development. Plant Molecular Biology, 2009, 71(4-5): 319.

[2] Barrôco R M, Peres A, Droual A M, et al. The cyclin-dependent kinase inhibitor Orysa; KRP1 plays an important role in seed development of rice. Plant Physiology, 2006, 142(3): 1053-1064.

[3] Jain M, Nijhawan A, Arora R, et al. F-box proteins in rice. Genome-wide analysis, classification, temporal and spatial gene expression during panicle and seed development, and regulation by light and abiotic stress. Plant Physiology, 2007, 143(4): 1467-1483.

[4] Hong M J, Kim D Y, Seo Y W. SKP1-like-related genes interact with various F-box proteins and may form SCF complexes with Cullin–F-box proteins in wheat. Molecular Biology Reports, 2013, 40(2): 969-981.

[5] Goubet, F. AtCSLA7, a cellulose synthase-like putative glycosyltransferase, is important for pollen tube growth and embryogenesis in Arabidopsis. Plant Physiology, 2003, 131(2):547-557.

[6] Kuroda H, Takahashi N, Shimada H, et al. Classification and expression analysis of Arabidopsis F-box-containing protein genes. Plant and Cell Physiology, 2002, 43(10): 1073-1085.

[7] Schruff M C, Spielman M, Tiwari S, et al. The AUXIN RESPONSE FACTOR 2 gene of Arabidopsis links auxin signalling, cell division, and the size of seeds and other organs. Development, 2006, 133(2): 251-261.

[8] Matsuoka M. Classification and characterization of cDNA that encodes the light-harvesting chlorophyll a/b binding protein of photosystem II from rice. Plant and Cell Physiology, 1990, 31(4): 519-526.

[9] Chen X, Hao L, Pan J, et al. SPL5, a cell death and defense-related gene, encodes a putative splicing factor 3b subunit 3 (SF3b3) in rice. Molecular Breeding, 2012, 30(2): 939-949.

[10] Gendron J M, Pruneda-Paz J L, Doherty C J, et al. Arabidopsis circadian clock protein, TOC1, is a DNA-binding transcription factor. Proceedings of the National Academy of Sciences, 2012, 109(8): 3167-3172.

[11] Markakis M N, Boron A K, Van Loock B, et al. Characterization of a small auxin-up RNA (SAUR)-like gene involved in Arabidopsis thaliana development. PloS one, 2013, 8(11).

[12] Zeng L R, Park C H, Venu R C, et al. Classification, expression pattern, and E3 ligase activity assay of rice U-box-containing proteins. Molecular Plant, 2008, 1(5): 800-815.

[13] Matte A, Goldie H, Sweet R M, et al. Crystal Structure ofEscherichia coliPhosphoenolpyruvate Carboxykinase: a new structural family with the P-loop nucleoside triphosphate hydrolase fold. Journal of Molecular Biology, 1996, 256(1): 126-143.

[14] Bentolila S, Alfonso A A, Hanson M R. A pentatricopeptide repeat-containing gene restores fertility to cytoplasmic male-sterile plants. Proceedings of the National Academy of Sciences, 2002, 99(16): 10887-10892.

[15] Hu Y, Zou W, Wang Z, et al. Translocase of the outer mitochondrial membrane 40 is required for mitochondrial biogenesis and embryo development in Arabidopsis. Frontiers in Plant Science, 2019, 10: 389.

[16] Zhang D, Liang W, Yin C, et al. OsC6, encoding a lipid transfer protein, is required for postmeiotic anther development in rice. Plant Physiology, 2010, 154(1): 149-162.

[17] Li J, Chory J. A putative leucine-rich repeat receptor kinase involved in brassinosteroid signal transduction. Cell, 1997, 90(5): 929-938.

[18] Koning A J, Tanimoto E Y, Kiehne K, et al. Cell-specific expression of plant histone H2A genes. The Plant Cell, 1991, 3(7): 657-665.

[19] Linthorst H J, Melchers L S, Mayer A, et al. Analysis of gene families encoding acidic and basic beta-1, 3-glucanases of tobacco. Proceedings of the National Academy of Sciences, 1990, 87(22): 8756-8760.

[20] Tornero P, Mayda E, Gómez M D, et al. Characterization of LRP, a leucine‐rich repeat (LRR) protein from tomato plants that is processed during pathogenesis. The Plant Journal, 1996, 10(2): 315-330.

[21] Büttner M, Singh K B. Arabidopsis thaliana ethylene-responsive element binding protein (AtEBP), an ethylene-inducible, GCC box DNA-binding protein interacts with an ocs element binding protein. Proceedings of the National Academy of Sciences, 1997, 94(11): 5961-5966.

[22] Gassmann W, Hinsch M E, Staskawicz B J. The Arabidopsis RPS4 bacterial‐resistance gene is a member of the TIR‐NBS‐LRR family of disease‐resistance genes. The Plant Journal, 1999, 20(3): 265-277.

[23] Nomura T, Ishihara A, Imaishi H, et al. Molecular characterization and chromosomal localization of cytochrome P450 genes involved in the biosynthesis of cyclic hydroxamic acids in hexaploid wheat. Molecular Genetics and Genomics, 2002, 267(2): 210-217.

[24] Lin H C, Chooi Y H, Dhingra S, et al. The fumagillin biosynthetic gene cluster in Aspergillus fumigatus encodes a cryptic terpene cyclase involved in the formation of β-trans-bergamotene. Journal of the American Chemical Society, 2013, 135(12): 4616-4619.

[25] Xie Z, Chen Z. Harpin-induced hypersensitive cell death is associated with altered mitochondrial functions in tobacco cells. Molecular plant-microbe interactions, 2000, 13(2): 183-190.

[26] Xu D, Duan X, Wang B, et al. Expression of a late embryogenesis abundant protein gene, HVA1, from barley confers tolerance to water deficit and salt stress in transgenic rice. Plant Physiology, 1996, 110(1): 249-257.

[27] Ishimaru Y, Suzuki M, Kobayashi T, et al. OsZIP4, a novel zinc-regulated zinc transporter in rice. Journal of Experimental Botany, 2005, 56(422): 3207-3214.

[28] Næsted H, Holm A, Jenkins T, et al. Arabidopsis VARIEGATED 3 encodes a chloroplast-targeted, zinc-finger protein required for chloroplast and palisade cell development. Journal of Cell Science, 2004, 117(20): 4807-4818.

[29] Griffiths S, Dunford R P, Coupland G, et al. The evolution of CONSTANS-like gene families in barley, rice, and Arabidopsis. Plant Physiology, 2003, 131(4): 1855-1867.

[30] Knip M, Hiemstra S, Sietsma A, et al. DAYSLEEPER: a nuclear and vesicular-localized protein that is expressed in proliferating tissues. BMC Plant Biology, 2013, 13(1): 211.

[31] Rancour D M, Park S, Knight S D, et al. Plant UBX domain-containing protein 1, PUX1, regulates the oligomeric structure and activity of Arabidopsis CDC48. Journal of Biological Chemistry, 2004, 279(52): 54264-54274.

[32] Hughes J M X. Functional base-pairing interaction between highly conserved elements of U3 small nucleolar RNA and the small ribosomal subunit RNA. Journal of Molecular Biology, 1996, 259(4): 645-654.

[33] Mewalal R, Mizrachi E, Coetzee B, et al. The Arabidopsis domain of unknown function 1218 (DUF1218) containing proteins, MODIFYING WALL LIGNIN-1 and 2 (At1g31720/MWL-1 and At4g19370/MWL-2) function redundantly to alter secondary cell wall lignin content. PloS one, 2016, 11(3).

[34] Tamura T, Hara K, Yamaguchi Y, et al. Osmotic stress tolerance of transgenic tobacco expressing a gene encoding a membrane-located receptor-like protein from tobacco plants. Plant Physiology, 2003, 131(2): 454-462.

[35] Mewalal R, Mizrachi E, Coetzee B, et al. The Arabidopsis domain of unknown function 1218 (DUF1218) containing proteins, MODIFYING WALL LIGNIN-1 and 2 (At1g31720/MWL-1 and At4g19370/MWL-2) function redundantly to alter secondary cell wall lignin content. PloS one, 2016, 11(3).

[36] Iwasaki Y, Komano M, Ishikawa A, et al. Molecular cloning and characterization of cDNA for a rice protein that contains seven repetitive segments of the Trp-Asp forty-amino-acid repeat (WD-40 repeat). Plant and Cell Physiology, 1995, 36(3): 505-510.

[37] Bahieldin A, Atef A, Edris S, et al. Ethylene responsive transcription factor ERF109 retards PCD and improves salt tolerance in plant. BMC Plant Biology, 2016, 16(1): 216.

[38] Scalliet G, Bowler J, Luksch T, et al. Mutagenesis and functional studies with succinate dehydrogenase inhibitors in the wheat pathogen Mycosphaerella graminicola. PLoS One, 2012, 7(4).

[39] Yoshida K T, Kuboyama T. A subtilisin-like serine protease specifically expressed in reproductive organs in rice. Sexual Plant Reproduction, 2001, 13(4): 193-199.

[40] Yu S, Galvão V C, Zhang Y C, et al. Gibberellin regulates the Arabidopsis floral transition through miR156-targeted SQUAMOSA PROMOTER BINDING–LIKE transcription factors. The Plant Cell, 2012, 24(8): 3320-3332.

[41] Knop M, Strasser K. Role of the spindle pole body of yeast in mediating assembly of the prospore membrane during meiosis. The EMBO Journal, 2000, 19(14): 3657-3667.

[42] Chen M, Markham J E, Dietrich C R, et al. Sphingolipid long-chain base hydroxylation is important for growth and regulation of sphingolipid content and composition in Arabidopsis. The Plant Cell, 2008, 20(7): 1862-1878.

[43] Yuan D, Wu Z, Wang Y. Evolution of the diacylglycerol lipases. Progress in Lipid Rresearch, 2016, 64: 85-97.

[44] Zhang F, Luo X, Hu B, et al. YGL138 (t), encoding a putative signal recognition particle 54 kDa protein, is involved in chloroplast development of rice. Rice, 2013, 6(1): 7.

[45] Chen Z, Hutchison M, Cobb M H. Isolation of the protein kinase TAO2 and identification of its mitogen-activated protein kinase/extracellular signal-regulated kinase kinase binding domain. Journal of Biological Chemistry, 1999, 274(40): 28803-28807.

[46] Mao X, Zhang H, Tian S, et al. TaSnRK2. 4, an SNF1-type serine/threonine protein kinase of wheat (Triticum aestivum L.), confers enhanced multistress tolerance in Arabidopsis. Journal of Experimental Botany, 2009, 61(3): 683-696.

[47] Huang J, Ghosh R, Bankaitis V A. Sec14-like phosphatidylinositol transfer proteins and the biological landscape of phosphoinositide signaling in plants. Biochimica et Biophysica Acta (BBA)-Molecular and Cell Biology of Lipids, 2016, 1861(9): 1352-1364.

[48] Yang D H, Kwak K J, Kim M K, et al. Expression of Arabidopsis glycine-rich RNA-binding protein AtGRP2 or AtGRP7 improves grain yield of rice (Oryza sativa) under drought stress conditions. Plant Science, 2014, 214: 106-112.

[49] Sami-Subbu R, Choi S B, Wu Y, et al. Identification of a cytoskeleton-associated 120 kDa RNA-binding protein in developing rice seeds. Plant Molecular Biology, 2001, 46(1): 79-88.

[50] Zhang F, Zhang Y C, Liao J Y, et al. The subunit of RNA N6-methyladenosine methyltransferase OsFIP regulates early degeneration of microspores in rice. PLoS Genetics, 2019, 15(5): e1008120.

[51] Ogasawara T, Sawasaki T, Morishita R, et al. A new class of enzyme acting on damaged ribosomes: ribosomal RNA apurinic site specific lyase found in wheat germ. The EMBO Journal, 1999, 18(22): 6522-6531.

[52] Van Lijsebettens M, Vanderhaeghen R, De Block M, et al. An S18 ribosomal protein gene copy at the Arabidopsis PFL locus affects plant development by its specific expression in meristems. The EMBO Journal, 1994, 13(14): 3378-3388.

[53] Molina-Hidalgo F J, Franco A R, Villatoro C, et al. The strawberry (Fragaria× ananassa) fruit-specific rhamnogalacturonate lyase 1 (FaRGLyase1) gene encodes an enzyme involved in the degradation of cell-wall middle lamellae. Journal of Experimental Botany, 2013, 64(6): 1471-1483.

[54] Alawady A E, Grimm B. Tobacco Mg protoporphyrin IX methyltransferase is involved in inverse activation of Mg porphyrin and protoheme synthesis. The Plant Journal, 2005, 41(2): 282-290.

[55] Rodriguez P L, Leube M P, Grill E. Molecular cloning in Arabidopsis thaliana of a new protein phosphatase 2C (PP2C) with homology to ABI1 and ABI2. Plant Molecular Biology, 1998, 38(5): 879-883.

[56] Ahmad A, Dong Y, Cao X. Characterization of the PRMT gene family in rice reveals conservation of arginine methylation. PLoS One, 2011, 6(8).

[57] Lytle B L, Peterson F C, Tyler E M, et al. Solution structure of Arabidopsis thaliana protein At5g39720. 1, a member of the AIG2-like protein family. Acta Crystallographica Section F: Structural Biology and Crystallization Communications, 2006, 62(6): 490-493.

[58] Genschik P, Jamet E, Phillips G, et al. Molecular characterization of a beta-type proteasome subunit from Arabidopsis thaliana co-expressed at a high level with an alpha-type proteasome subunit early in the cell cycle. Plant Journal, 2010, 6(4):537-546.

[59] Stein H, Honig A, Miller G, et al. Elevation of free proline and proline-rich protein levels by simultaneous manipulations of proline biosynthesis and degradation in plants. Plant Science, 2011, 181(2): 140-150.

[60] Heindl K, Martinez J. Nol9 is a novel polynucleotide 5′‐kinase involved in ribosomal RNA processing. The EMBO Journal, 2010, 29(24): 4161-4171.

[61] Bessire M, Borel S, Fabre G, et al. A member of the PLEIOTROPIC DRUG RESISTANCE family of ATP binding cassette transporters is required for the formation of a functional cuticle in Arabidopsis. The Plant Cell, 2011, 23(5): 1958-1970.

[62] Waki T, Hiki T, Watanabe R, et al. The Arabidopsis RWP-RK protein RKD4 triggers gene expression and pattern formation in early embryogenesis. Current Biology, 2011, 21(15): 1277-1281.

[63] Albus C A, Ruf S, Schöttler M A, et al. Y3IP1, a nucleus-encoded thylakoid protein, cooperates with the plastid-encoded Ycf3 protein in photosystem I assembly of tobacco and Arabidopsis. The Plant Cell, 2010, 22(8): 2838-2855.

[64] Rubio S, Whitehead L, Larson T R, et al. The coenzyme a biosynthetic enzyme phosphopantetheine adenylyltransferase plays a crucial role in plant growth, salt/osmotic stress resistance, and seed lipid storage. Plant Physiology, 2008, 148(1): 546-556.

[65] Kar M, Mishra D. Catalase, peroxidase, and polyphenoloxidase activities during rice leaf senescence. Plant Physiology, 1976, 57(2): 315-319.

[66] Bentolila S, Alfonso A A, Hanson M R. A pentatricopeptide repeat-containing gene restores fertility to cytoplasmic male-sterile plants. Proceedings of the National Academy of Sciences, 2002, 99(16): 10887-10892.

[67] Hjelmqvist L, Tuson M, Marfany G, et al. ORMDL proteins are a conserved new family of endoplasmic reticulum membrane proteins. Genome Biology, 2002, 3(6): research0027. 1.

[68] Rentsch D, Laloi M, Rouhara I, et al. NTR1 encodes a high affinity oligopeptide transporter in Arabidopsis. FEBS letters, 1995, 370(3): 264-268.

[69] Zhang B, Liu X, Qian Q, et al. Golgi nucleotide sugar transporter modulates cell wall biosynthesis and plant growth in rice. Proceedings of the National Academy of Sciences, 2011, 108(12): 5110-5115.

[70] Parry G. Assessing the function of the plant nuclear pore complex and the search for specificity. Journal of Experimental Botany, 2013, 64(4): 833-845.

[71] Bi X, Khush G S, Bennett J. The rice nucellin gene ortholog OsAsp1 encodes an active aspartic protease without a plant-specific insert and is strongly expressed in early embryo. Plant and Cell Physiology, 2005, 46(1): 87-98.

[72] Haydon M J, Cobbett C S. A novel major facilitator superfamily protein at the tonoplast influences zinc tolerance and accumulation in Arabidopsis. Plant Physiology, 2007, 143(4): 1705-1719.

[73] Gassmann W, Hinsch M E, Staskawicz B J. The Arabidopsis RPS4 bacterial‐resistance gene is a member of the TIR‐NBS‐LRR family of disease‐resistance genes. The Plant Journal, 1999, 20(3): 265-277.

[74] Martínez I, Zhu J, Lin H, et al. Replacing Escherichia coli NAD-dependent glyceraldehyde 3-phosphate dehydrogenase (GAPDH) with a NADP-dependent enzyme from Clostridium acetobutylicum facilitates NADPH dependent pathways. Metabolic Engineering, 2008, 10(6): 352-359.

[75] Oh S K, Lee S, Yu S H, et al. Expression of a novel NAC domain-containing transcription factor (CaNAC1) is preferentially associated with incompatible interactions between chili pepper and pathogens. Planta, 2005, 222(5): 876-887.

[76] Yanhui C, Xiaoyuan Y, Kun H, et al. The MYB transcription factor superfamily of Arabidopsis: expression analysis and phylogenetic comparison with the rice MYB family. Plant Molecular Biology, 2006, 60(1): 107-124.

[77] Kovtun Y, Chiu W L, Tena G, et al. Functional analysis of oxidative stress-activated mitogen-activated protein kinase cascade in plants. Proceedings of the National Academy of Sciences, 2000, 97(6): 2940-2945.

[78] Li W Q, Zhang X Q, Xia C, et al. MALE GAMETOPHYTE DEFECTIVE 1, encoding the FAd subunit of mitochondrial F1F0-ATP synthase, is essential for pollen formation in Arabidopsis thaliana. Plant and Cell Physiology, 2010, 51(6): 923-935.

[79] Shinshi H, Neuhaus J M, Ryals J, et al. Structure of a tobacco endochitinase gene: evidence that different chitinase genes can arise by transposition of sequences encoding a cysteine-rich domain. Plant Molecular Biology, 1990, 14(3): 357-368.

[80] Li J, Chory J. A putative leucine-rich repeat receptor kinase involved in brassinosteroid signal transduction. Cell, 1997, 90(5): 929-938.

[81] Sundin L J R, Guimaraes G J, DeLuca J G. The NDC80 complex proteins Nuf2 and Hec1 make distinct contributions to kinetochore–microtubule attachment in mitosis. Molecular Biology of the Cell, 2011, 22(6): 759-768.

[82] Liu B, Cyr R J, Palevitz B A. A kinesin-like protein, KatAp, in the cells of Arabidopsis and other plants. The Plant Cell, 1996, 8(1): 119-132.

[83] Terada R, Nakayama T, Iwabuchi M, et al. A wheat histone H3 promoter confers cell division‐dependent and‐independent expression of the gus A gene in transgenic rice plants. The Plant Journal, 1993, 3(2): 241-252.

[84] Song X J, Kuroha T, Ayano M, et al. Rare allele of a previously unidentified histone H4 acetyltransferase enhances grain weight, yield, and plant biomass in rice. Proceedings of the National Academy of Sciences, 2015, 112(1): 76-81.

[85] Guo J, Wu J, Ji Q, et al. Genome-wide analysis of heat shock transcription factor families in rice and Arabidopsis. Journal of Genetics and Genomics, 2008, 35(2): 105-118.

[86] Yamaguchi K, Imai K, Akamatsu A, et al. SWAP70 functions as a Rac/Rop guanine nucleotide‐exchange factor in rice. The Plant Journal, 2012, 70(3): 389-397.

[87] Cao Y, Han Y, Jin Q, et al. Comparative genomic analysis of the GRF genes in Chinese pear (Pyrus bretschneideri Rehd), poplar (Populous), grape (Vitis vinifera), Arabidopsis and rice (Oryza sativa). Frontiers in plant science, 2016, 7: 1750.

[88] Keller B, Templeton M D, Lamb C J. Specific localization of a plant cell wall glycine-rich protein in protoxylem cells of the vascular system. Proceedings of the National Academy of Sciences, 1989, 86(5): 1529-1533.

[89] Zhu X F, Suzuki K, Saito T, et al. Geranylgeranyl pyrophosphate synthase encoded by the newly isolated gene GGPS6 from Arabidopsis thaliana is localized in mitochondria. Plant molecular biology, 1997, 35(3): 331-341.

[90] Dong X, Yi H, Han C T, et al. GDSL esterase/lipase genes in Brassica rapa L.: genome-wide identification and expression analysis. Molecular Genetics and Genomics, 2016, 291(2): 531-542.

[91] Ambrose C, Wasteneys G O. Cell edges accumulate gamma tubulin complex components and nucleate microtubules following cytokinesis in Arabidopsis thaliana. PLoS One, 2011, 6(11).

[92] Kaplan C P, Tugal H B, Baker A. Isolation of a cDNA encoding an Arabidopsis galactokinase by functional expression in yeast. Plant molecular biology, 1997, 34(3): 497-506.

[93] Angot A, Peeters N, Lechner E, et al. Ralstonia solanacearum requires F-box-like domain-containing type III effectors to promote disease on several host plants. Proceedings of the National Academy of Sciences, 2006, 103(39): 14620-14625.

[94] Iba K, Gibson S, Nishiuchi T, et al. A gene encoding a chloroplast omega-3 fatty acid desaturase complements alterations in fatty acid desaturation and chloroplast copy number of the fad7 mutant of Arabidopsis thaliana. Journal of Biological Chemistry, 1993, 268(32): 24099-24105.

[95] Johnson K L, Jones B J, Bacic A, et al. The fasciclin-like arabinogalactan proteins of Arabidopsis. A multigene family of putative cell adhesion molecules. Plant physiology, 2003, 133(4): 1911-1925.

[96] Fingar D C, Richardson C J, Tee A R, et al. mTOR controls cell cycle progression through its cell growth effectors S6K1 and 4E-BP1/eukaryotic translation initiation factor 4E. Molecular and cellular biology, 2004, 24(1): 200-216.

[97] Liu W, Wu C, Fu Y, et al. Identification and characterization of HTD2: a novel gene negatively regulating tiller bud outgrowth in rice. Planta, 2009, 230(4): 649-658.

[98] Zhang Q, Zhang W, Lin C, et al. Expression of an Acidothermus cellulolyticus endoglucanase in transgenic rice seeds. Protein expression and purification, 2012, 82(2): 279-283.

[99] Zhang Q, Zhang W, Lin C, et al. Expression of an Acidothermus cellulolyticus endoglucanase in transgenic rice seeds. Protein expression and purification, 2012, 82(2): 279-283.

[100] Song X J, Huang W, Shi M, et al. A QTL for rice grain width and weight encodes a previously unknown RING-type E3 ubiquitin ligase. Nature genetics, 2007, 39(5): 623-630.

[101] Song X J, Huang W, Shi M, et al. A QTL for rice grain width and weight encodes a previously unknown RING-type E3 ubiquitin ligase. Nature genetics, 2007, 39(5): 623-630.

[102] Cui Y, Wang M, Zhou H, et al. OsSGL, a novel DUF1645 domain-containing protein, confers enhanced drought tolerance in transgenic rice and Arabidopsis. Frontiers in plant science, 2016, 7: 2001.

[103] Kanehara K, Cho Y, Lin Y C, et al. Arabidopsis DOK 1 encodes a functional dolichol kinase involved in reproduction. The Plant Journal, 2015, 81(2): 292-303.

[104] Breuer C, Stacey N J, West C E, et al. BIN4, a novel component of the plant DNA topoisomerase VI complex, is required for endoreduplication in Arabidopsis. The Plant Cell, 2007, 19(11): 3655-3668.

[105] Ursic D, Chinchilla K, Finkel J S, et al. Multiple protein/protein and protein/RNA interactions suggest roles for yeast DNA/RNA helicase Sen1p in transcription, transcription‐coupled DNA repair and RNA processing. Nucleic acids research, 2004, 32(8): 2441-2452.

[106] Troelstra C, van Gool A, de Wit J, et al. ERCC6, a member of a subfamily of putative helicases, is involved in Cockayne's syndrome and preferential repair of active genes. Cell, 1992, 71(6): 939-953.

[107] Balmukhanov T S, Erekenov A M, Ajtkhozhina N A. Isolation of DNA polymerase alpha from germinated wheat embryos. Molecular biology reports, 1992, 16(1): 11-16.

[108] Culligan K M, Hays J B. DNA mismatch repair in plants (an Arabidopsis thaliana gene that predicts a protein belonging to the MSH2 subfamily of eukaryotic MutS homologs). Plant Physiology, 1997, 115(2): 833-839.

[109] Gassmann W, Hinsch M E, Staskawicz B J. The Arabidopsis RPS4 bacterial‐resistance gene is a member of the TIR‐NBS‐LRR family of disease‐resistance genes. The Plant Journal, 1999, 20(3): 265-277.

[110] Meyers B C, Dickerman A W, Michelmore R W, et al. Plant disease resistance genes encode members of an ancient and diverse protein family within the nucleotide‐binding superfamily. The Plant Journal, 1999, 20(3): 317-332.

[111] Ma Q H, Liu Y C. TaDIR13, a dirigent protein from wheat, promotes lignan biosynthesis and enhances pathogen resistance. Plant Molecular Biology Reporter, 2015, 33(1): 143-152.

[112] Kaczor C M, Smith M W, Sangwan I, et al. Plant [delta]-Aminolevulinic Acid Dehydratase (Expression in Soybean Root Nodules and Evidence for a Bacterial Lineage of the Alad Gene). Plant Physiology, 1994, 104(4): 1411-1417.

[113] Taylor D L, Fellows L E, Farrar G H, et al. Loss of cytomegalovirus infectivity after treatment with castanospermine or related plant alkaloids correlates with aberrant glycoprotein synthesis. Antiviral Research, 1988, 10(1-3): 11-26.

[114] Nomura T, Ishihara A, Imaishi H, et al. Molecular characterization and chromosomal localization of cytochrome P450 genes involved in the biosynthesis of cyclic hydroxamic acids in hexaploid wheat. Molecular Genetics and Genomics, 2002, 267(2): 210-217.

[115] Bravo J, Aguilar-Henonin L, Olmedo G, et al. Four distinct classes of proteins as interaction partners of the PABC domain of Arabidopsis thaliana Poly (A)-binding proteins. Molecular Genetics and Genomics, 2005, 272(6): 651-665.

[116] Shih P Y, Lee S P, Chen Y K, et al. Cortactin-binding protein 2 increases microtubule stability and regulates dendritic arborization. J Cell Sci, 2014, 127(16): 3521-3534.

[117] Mousdale D M, Coggins J R. Detection and subcellular localization of a higher plant chorismate synthase. FEBS Letters, 1986, 205(2): 328-332.

[118] Nakamura A, Fukuda A, Sakai S, et al. Molecular cloning, functional expression and subcellular localization of two putative vacuolar voltage-gated chloride channels in rice (Oryza sativa L.). Plant and Cell Physiology, 2006, 47(1): 32-42.

[119] Hermans C, Porco S, Verbruggen N, et al. Chitinase-like protein CTL1 plays a role in altering root system architecture in response to multiple environmental conditions. Plant Physiology, 2010, 152(2): 904-917.

[120] Favery B, Ryan E, Foreman J, et al. KOJAK encodes a cellulose synthase-like protein required for root hair cell morphogenesis in Arabidopsis. Genes & Development, 2001, 15(1): 79-89.

[121] TAYLOR W R, GREEN N M. The predicted secondary structures of the nucleotide‐binding sites of six cation‐transporting ATPases lead to a probable tertiary fold. European Journal of Biochemistry, 1989, 179(1): 241-248.

[122] Roppolo D, De Rybel B, Tendon V D, et al. A novel protein family mediates Casparian strip formation in the endodermis. Nature, 2011, 473(7347): 380-383.

[123] Komatsu S, Yang G, Khan M, et al. Over-expression of calcium-dependent protein kinase 13 and calreticulin interacting protein 1 confers cold tolerance on rice plants. Molecular Genetics and Genomics, 2007, 277(6): 713.

[124] Yamamoto M, Shitsukawa N, Yamada M, et al. Identification of a novel homolog for a calmodulin-binding protein that is upregulated in alloplasmic wheat showing pistillody. Planta, 2013, 237(4): 1001-1013.

[125] Yamamoto M, Shitsukawa N, Yamada M, et al. Identification of a novel homolog for a calmodulin-binding protein that is upregulated in alloplasmic wheat showing pistillody. Planta, 2013, 237(4): 1001-1013.

[126] Welters P, Takegawa K, Emr S D, et al. AtVPS34, a phosphatidylinositol 3-kinase of Arabidopsis thaliana, is an essential protein with homology to a calcium-dependent lipid binding domain. Proceedings of the National Academy of Sciences, 1994, 91(24): 11398-11402.

[127] Schmidt W K, Tam A, Fujimura-Kamada K, et al. Endoplasmic reticulum membrane localization of Rce1p and Ste24p, yeast proteases involved in carboxyl-terminal CAAX protein processing and amino-terminal a-factor cleavage. Proceedings of the National Academy of Sciences, 1998, 95(19): 11175-11180.

[128] Jakoby M, Weisshaar B, Dröge-Laser W, et al. bZIP transcription factors in Arabidopsis. Trends in Plant Science, 2002, 7(3): 106-111.

[129] Ha C M, Jun J H, Nam H G, et al. BLADE-ON-PETIOLE1 encodes a BTB/POZ domain protein required for leaf morphogenesis in Arabidopsis thaliana. Plant and Cell Physiology, 2004, 45(10): 1361-1370.

[130] Yang M, McCormick S. The Arabidopsis MEI1 gene likely encodes a protein with BRCT domains. Sexual Plant Reproduction, 2002, 14(6): 355-357.

[131] Luerßen H, Kirik V, Herrmann P, et al. FUSCA3 encodes a protein with a conserved VP1/ABI3‐like B3 domain which is of functional importance for the regulation of seed maturation in Arabidopsis thaliana. The Plant Journal, 1998, 15(6): 755-764.

[132] Liu Y, Imai R. Function of plant DExD/H-box RNA helicases associated with ribosomal RNA biogenesis. Frontiers in Plant Science, 2018, 9: 125.

[133] Nakamura T, Muramoto Y, Yokota S, et al. Structural and transcriptional characterization of a salt-responsive gene encoding putative ATP-dependent RNA helicase in barley. Plant Science, 2004, 167(1): 63-70.

[134] Curnow A W, Hong K, Yuan R, et al. Glu-tRNAGln amidotransferase: a novel heterotrimeric enzyme required for correct decoding of glutamine codons during translation. Proceedings of the National Academy of Sciences, 1997, 94(22): 11819-11826.

[135] Park M, Song K, Reichardt I, et al. Arabidopsis μ-adaptin subunit AP1M of adaptor protein complex 1 mediates late secretory and vacuolar traffic and is required for growth. Proceedings of the National Academy of Sciences, 2013, 110(25): 10318-10323.

[136] Sikdar M S I, Kim J S. Functional analysis of a gene encoding Anthranilate phosphoribosyltransferase from rice[C]//Proceedings of International Conference on Biology, Environment and Chemistry (ICBEC 2010). 2010.

[137] Jarrett S J, Marschke R J, Symons M H, et al. Alpha-amylase/subtilisin inhibitor levels in Australian barleys. Journal of Cereal Science, 1997, 25(3): 261-266.

[138] Kulkarni R D, Dean R A. Identification of proteins that interact with two regulators of appressorium development, adenylate cyclase and cAMP-dependent protein kinase A, in the rice blast fungus Magnaporthe grisea. Molecular Genetics and Genomics, 2004, 270(6): 497-508.

[139] Lu C A, Lin C C, Lee K W, et al. The SnRK1A protein kinase plays a key role in sugar signaling during germination and seedling growth of rice. The Plant Cell, 2007, 19(8): 2484-2499.

[140] Di R, Blechl A, Dill-Macky R, et al. Expression of a truncated form of yeast ribosomal protein L3 in transgenic wheat improves resistance to Fusarium head blight. Plant science, 2010, 178(4): 374-380.

[141] Xie S, Wu H, Chen L, et al. Transcriptome profiling of Bacillus subtilis OKB105 in response to rice seedlings. BMC Microbiology, 2015, 15(1): 21.

[142] Kawai Y, Ono E, Mizutani M. Evolution and diversity of the 2–oxoglutarate‐dependent dioxygenase superfamily in plants. The Plant Journal, 2014, 78(2): 328-343.

[143] Lee H, Choi A J, Kang G Y, et al. Increased 26S proteasome non-ATPase regulatory subunit 1 in the aqueous humor of patients with age-related macular degeneration. BMB Reports, 2014, 47(5): 292.

**Table S9.3** Predicted genes in the interval of the major QTL *QKT.sicau-2SY-2D*, *QTKW.sicau-2SY-2D*, *QKS.sicau-2SY-2D* and *QFFD.sicau-2SY-2D*

| **Gene ID in CS** | **Annotation** | **Orthologs in Aegilops tauschii** | **References** |
| --- | --- | --- | --- |
| *TraesCS2D01G391600.1* | **Expansin** | *AET2Gv20878000.1* | [1] |
| *TraesCS2D01G385000.1* | **Kinesin-like protein** | *AET2Gv20863300.1* | [2] |
| *TraesCS2D01G388300.1* | **F-box family protein** | *AET2Gv20866700.1* | [3] |
| *TraesCS2D01G378100.1* | Kinase family protein | *AET2Gv20852100.1* | [4] |
| *TraesCS2D01G386900.1* | Kinase family protein | *AET2Gv20870900.1* | [4] |
| *TraesCS2D01G388000.1* | Kinase family protein | *AET2Gv20867500.1* | [4] |
| *TraesCS2D01G389200.1* | Kinase family protein | *AET2Gv20873800.1* | [4] |
| *TraesCS2D01G391000.1* | Kinase family protein | *AET2Gv20877000.1* | [4] |
| *TraesCS2D01G396100.1* | Xyloglucan galactosyltransferase KATAMARI1-like protein | *AET2Gv20886900.1* | [5] |
| *TraesCS2D01G396200.1* | Xyloglucan galactosyltransferase KATAMARI1-like protein | *AET2Gv20887000.1* | [5] |
| *TraesCS2D01G377800.1* | SAUR-like auxin-responsive family protein | *AET2Gv20851400.1* | [6] |
| *TraesCS2D01G383200.1* | SAUR-like auxin-responsive family protein | *AET2Gv20860700.1* | [6] |
| *TraesCS2D01G385600.1* | Required to maintain repression 2 | *AET2Gv20864200.1* | [8] |
| *TraesCS2D01G385800.1* | Required to maintain repression 2 | *AET2Gv20864500.1* | [8] |
| *TraesCS2D01G385900.1* | Required to maintain repression 2 | *AET2Gv20865100.1* | [8] |
| *TraesCS2D01G377900.1* | Protein kinase family protein | *AET2Gv20851600.1* | [9] |
| *TraesCS2D01G378000.1* | Protein kinase family protein | *AET2Gv20851900.1* | [9] |
| *TraesCS2D01G389900.1* | Pentatricopeptide repeat-containing protein | *AET2Gv20874700.1* | [10] |
| *TraesCS2D01G391800.1* | Pentatricopeptide repeat-containing protein | *AET2Gv20878800.1* | [10] |
| *TraesCS2D01G388900.1* | Histone H1 | *AET2Gv20873000.1* | [11] |
| *TraesCS2D01G395200.1* | Histone H1 | *AET2Gv20886200.1* | [11] |
| *TraesCS2D01G397000.1* | Dehydration-responsive element binding factor | *AET2Gv20888800.1* | [12] |
| *TraesCS2D01G397100.1* | Dehydration-responsive element binding factor | *AET2Gv20889100.1* | [12] |
| *TraesCS2D01G392800.1* | 2-oxoglutarate (2OG) and Fe(II)-dependent oxygenase superfamily protein | *AET2Gv20881200.1* | [13] |
| *TraesCS2D01G392900.1* | 2-oxoglutarate (2OG) and Fe(II)-dependent oxygenase superfamily protein | *AET2Gv20881400.1* | [13] |
| *TraesCS2D01G377200.1* | Phenylalanine ammonia-lyase | *AET2Gv20850300.1* | [14] |
| *TraesCS2D01G377300.1* | Protein FAR1-RELATED SEQUENCE 3 | *AET2Gv20850700.1* | [15] |
| *TraesCS2D01G386300.1* | Zinc finger, B-box | *AET2Gv20866200.1* | [16] |
| *TraesCS2D01G382700.1* | YABBY protein | *AET2Gv20860100.1* | [17] |
| *TraesCS2D01G390200.1* | WRKY transcription factor | *AET2Gv20875200.1* | [18] |
| *TraesCS2D01G383400.1* | Vacuolar-processing enzyme | *AET2Gv20861200.1* | [19] |
| *TraesCS2D01G396900.1* | Vacuolar protein sorting-associated protein 53 A | *AET2Gv20888700.1* | [20] |
| *TraesCS2D01G383900.1* | Vacuolar iron transporter | *AET2Gv20861700.1* | [21] |
| *TraesCS2D01G387400.1* | Ubiquitin-specific protease family C19-related protein | *AET2Gv20868400.1* | [22] |
| *TraesCS2D01G394900.1* | Ubiquitin fusion degradation 1 protein | *AET2Gv20885800.1* | [23] |
| *TraesCS2D01G387100.1* | Trihelix transcription factor GT-2 | *AET2Gv20869300.1* | [24] |
| *TraesCS2D01G388500.1* | Trihelix transcription factor | *AET2Gv20872600.1* | [24] |
| *TraesCS2D01G382200.1* | Transporter | *AET2Gv20859000.1* | [25] |
| *TraesCS2D01G394300.1* | Transmembrane protein 14C | *AET2Gv20884700.1* | [26] |
| *TraesCS2D01G390300.1* | transcription repressor | *AET2Gv20875900.1* | [27] |
| *TraesCS2D01G394500.1* | TatD family | *AET2Gv20885100.1* | [28] |
| *TraesCS2D01G396500.1* | Superoxide dismutase [Cu-Zn] | *AET2Gv20888100.1* | [29] |
| *TraesCS2D01G396400.1* | Subtilisin-like protease | *AET2Gv20888000.1* | [30] |
| *TraesCS2D01G395700.1* | S-type anion channel | *AET2Gv20886500.1* | [31] |
| *TraesCS2D01G396700.1* | SPX domain-containing family protein | *AET2Gv20888400.1* | [32] |
| *TraesCS2D01G387200.1* | Sphingoid long-chain bases kinase 1 | *AET2Gv20868800.1* | [33] |
| *TraesCS2D01G379200.1* | Sister chromatid cohesion protein PDS5-like protein B | *AET2Gv20853400.1* | N |
| *TraesCS2D01G386200.1* | SH3 domain-containing protein 2 | *AET2Gv20866000.1* | [34] |
| *TraesCS2D01G387500.1* | senescence regulator (Protein of unknown function, DUF584) | *AET2Gv20868000.1* | [35] |
| *TraesCS2D01G397900.1* | S-adenosyl-L-methionine-dependent methyltransferases superfamily protein | *AET2Gv20891100.1* | [36] |
| *TraesCS2D01G388400.1* | RNA binding protein, putative | *AET2Gv20872400.1* | [37] |
| *TraesCS2D01G392600.1* | RING/U-box superfamily protein, putative | *AET2Gv20880900.1* | [38] |
| *TraesCS2D01G397200.1* | Ring finger protein, putative | *AET2Gv20889200.1* | [39] |
| *TraesCS2D01G393400.1* | RING finger family protein | *AET2Gv20882700.1* | [40] |
| *TraesCS2D01G394800.1* | Rho GTPase-activating protein | *AET2Gv20885400.1* | [41] |
| *TraesCS2D01G390600.1* | responsive to abscisic acid 28 | *AET2Gv20876400.1* | [42] |
| *TraesCS2D01G378700.1* | Protodermal factor 1 | *AET2Gv20852800.1* | [43] |
| *TraesCS2D01G394000.1* | Protein SPIRAL1 | *AET2Gv20883300.1* | [44] |
| *TraesCS2D01G378300.1* | Protein root UVB sensitive 2, chloroplastic | *AET2Gv20852300.1* | [45] |
| *TraesCS2D01G390900.1* | Protein ROOT PRIMORDIUM DEFECTIVE 1 | *AET2Gv20876900.1* | [46] |
| *TraesCS2D01G397500.1* | Protein DETOXIFICATION | *AET2Gv20890600.1* | [47] |
| *TraesCS2D01G386400.1* | Protein COBRA | *AET2Gv20866400.1* | [48] |
| *TraesCS2D01G381600.1* | Protein BREAST CANCER SUSCEPTIBILITY 1-like protein | *AET2Gv20857900.1* | [49] |
| *TraesCS2D01G392100.1* | p-loop containing nucleoside triphosphate hydrolases superfamily protein, putative | *AET2Gv20879100.1* | [50] |
| *TraesCS2D01G391900.1* | p-loop containing nucleoside triphosphate hydrolases superfamily protein | *AET2Gv20879000.1* | [50] |
| *TraesCS2D01G394600.1* | pleckstrin homology (PH) domain-containing protein | *AET2Gv20885200.1* | [51] |
| *TraesCS2D01G380900.1* | plastid transcriptionally active 5 | *AET2Gv20856400.1* | [52] |
| *TraesCS2D01G384800.1* | Phosphoglucosamine mutase | *AET2Gv20863200.1* | N |
| *TraesCS2D01G380300.1* | Phosducin-like protein | *AET2Gv20855200.1* | N |
| *TraesCS2D01G377400.1* | Phenylalanine ammonia-lyase | *AET2Gv20850500.1* | [53] |
| *TraesCS2D01G396800.1* | Peptidoglycan-binding LysM domain-containing protein | *AET2Gv20888600.1* | [54] |
| *TraesCS2D01G382900.1* | Pathogenesis-related protein 1 | *AET2Gv20860300.1* | [55] |
| *TraesCS2D01G396600.1* | Oxygen-dependent choline dehydrogenase | *AET2Gv20888300.1* | [56] |
| *TraesCS2D01G391700.1* | Oxidative stress 3, putative isoform 2 | *AET2Gv20878300.1* | [57] |
| *TraesCS2D01G394100.1* | Nuclear transport factor 2 (NTF2) family protein | *AET2Gv20884400.1* | [58] |
| *TraesCS2D01G395000.1* | Non-specific serine/threonine protein kinase | *AET2Gv20886000.1* | [59] |
| *TraesCS2D01G385300.1* | NAD(P)H-quinone oxidoreductase subunit M | *AET2Gv20863700.1* | [60] |
| *TraesCS2D01G382800.1* | NAC domain protein, | *AET2Gv20860200.1* | [61] |
| *TraesCS2D01G378800.1* | NAC domain protein | *AET2Gv20853000.1* | [61] |
| *TraesCS2D01G386800.1* | Multiprotein-bridging factor, putative | *AET2Gv20871000.1* | [62] |
| *TraesCS2D01G378600.1* | Mitochondrial import receptor subunit TOM22 | *AET2Gv20852700.1* | [63] |
| *TraesCS2D01G385200.1* | Metallothiol transferase fosB | *AET2Gv20863600.1* | N |
| *TraesCS2D01G383700.1* | Light-independent protochlorophyllide reductase iron-sulfur ATP-binding protein | *AET2Gv20861500.1* | [64] |
| *TraesCS2D01G378900.1* | LIGHT-DEPENDENT SHORT HYPOCOTYLS-like protein (DUF640) | *AET2Gv20853200.1* | [65] |
| *TraesCS2D01G379000.1* | L-allo-threonine aldolase | *AET2Gv20853300.1* | [66] |
| *TraesCS2D01G384400.1* | Hydroxyproline-rich glycoprotein family protein | *AET2Gv20862400.1* | [67] |
| *TraesCS2D01G390800.1* | HXXXD-type acyl-transferase family protein, putative | *AET2Gv20876800.1* | [68] |
| *TraesCS2D01G383600.1* | Hsp70-Hsp90 organizing protein 1 | *AET2Gv20861400.1* | [69] |
| *TraesCS2D01G387300.1* | Homeobox protein, putative | *AET2Gv20868600.1* | [70] |
| *TraesCS2D01G385500.1* | Homeobox leucine zipper protein | *AET2Gv20863900.1* | [71] |
| *TraesCS2D01G389100.1* | Histone-lysine N-methyltransferase, H3 lysine-9 specific | *AET2Gv20873200.1* | [72] |
| *TraesCS2D01G391500.1* | Histone H4 | *AET2Gv20877900.1* | [73] |
| *TraesCS2D01G382500.1* | Histidine triad nucleotide binding protein | *AET2Gv20859700.1* | [74] |
| *TraesCS2D01G378200.1* | Hexosyltransferase | *AET2Gv20852200.1* | N |
| *TraesCS2D01G392200.1* | Heavy metal transport/detoxification superfamily protein | *AET2Gv20879800.1* | [75] |
| *TraesCS2D01G389700.1* | HAUS augmin-like complex subunit 6 | *AET2Gv20874200.1* | N |
| *TraesCS2D01G395900.1* | Growth-regulating factor | *AET2Gv20886700.1* | [76] |
| *TraesCS2D01G393200.1* | GRAS transcription factor | *AET2Gv20882500.1* | [77] |
| *TraesCS2D01G388800.1* | Glutamate dehydrogenase | *AET2Gv20872900.1* | [78] |
| *TraesCS2D01G377700.1* | Glucuronoxylan 4-O-methyltransferase | *AET2Gv20851000.1* | N |
| *TraesCS2D01G390000.1* | GATA transcription factor, putative | *AET2Gv20874800.1* | [79] |
| *TraesCS2D01G386100.1* | GATA transcription factor | *AET2Gv20865800.1* | [79] |
| *TraesCS2D01G386600.1* | Formin-like protein | *AET2Gv20871600.1* | [80] |
| *TraesCS2D01G393700.1* | Ferric reduction oxidase 2 | *AET2Gv20883700.1* | [81] |
| *TraesCS2D01G397700.1* | F-box protein PP2-A13 | *AET2Gv20890900.1* | [82] |
| *TraesCS2D01G381800.1* | Fatty acid hydroxylase superfamily protein | *AET2Gv20858100.1* | [83] |
| *TraesCS2D01G396000.1* | Fasciclin-like arabinogalactan protein | *AET2Gv20886800.1* | [84] |
| *TraesCS2D01G388100.1* | Eukaryotic translation initiation factor 4G | *AET2Gv20867400.1* | [85] |
| *TraesCS2D01G391400.1* | Ethylene-responsive transcription factor | *AET2Gv20877800.1* | [86] |
| *TraesCS2D01G381400.1* | Enolase | *AET2Gv20857400.1* | [87] |
| *TraesCS2D01G381500.1* | Embryo sac development arrest 6, putative | *AET2Gv20857700.1* | [88] |
| *TraesCS2D01G383800.1* | Elongation factor G | *AET2Gv20861600.1* | [89] |
| *TraesCS2D01G390400.1* | ELMO domain-containing protein, putative | *AET2Gv20876000.1* | [90] |
| *TraesCS2D01G390500.1* | ELMO domain-containing protein A | *AET2Gv20876200.1* | [90] |
| *TraesCS2D01G393000.1* | DUF1191 superfamily protein | *AET2Gv20882000.1* | [91] |
| *TraesCS2D01G380400.1* | Disease resistance protein (NBS-LRR class) family | *AET2Gv20856000.1* | [92] |
| *TraesCS2D01G380200.1* | Dihydroxy-acid dehydratase | *AET2Gv20855700.1* | N |
| *TraesCS2D01G379600.1* | dCTP pyrophosphatase 1 | *AET2Gv20854100.1* | [93] |
| *TraesCS2D01G386000.1* | Cytochrome P450 family protein | *AET2Gv20865200.1* | [94] |
| *TraesCS2D01G391200.1* | Cysteine-rich receptor-kinase-like protein | *AET2Gv20877200.1* | [95] |
| *TraesCS2D01G384500.1* | Cysteine desulfurase | *AET2Gv20862700.1* | [96] |
| *TraesCS2D01G389800.1* | Cyclin-like | *AET2Gv20874400.1* | [97] |
| *TraesCS2D01G380500.1* | CC-NBS-LRR family disease resistance protein | *AET2Gv20856200.1* | [98] |
| *TraesCS2D01G379300.1* | CASP-like protein | *AET2Gv20853500.1* | N |
| *TraesCS2D01G388200.1* | Casein kinase II subunit beta | *AET2Gv20867200.1* | [99] |
| *TraesCS2D01G382300.1* | Caleosin | *AET2Gv20859200.1* | [100] |
| *TraesCS2D01G385100.1* | BRCT domain-containing protein | *AET2Gv20863500.1* | [101] |
| *TraesCS2D01G390700.1* | Blue copper protein | *AET2Gv20876600.1* | [102] |
| *TraesCS2D01G381300.1* | Beta-glucosidase, putative | *AET2Gv20857000.1* | [103] |
| *TraesCS2D01G381000.1* | Beta-glucosidase | *AET2Gv20856500.1* | [103] |
| *TraesCS2D01G393900.1* | Beta-carotene hydroxylase | *AET2Gv20883500.1* | [104] |
| *TraesCS2D01G393500.1* | Beta-1,3-N-acetylglucosaminyltransferase lunatic fringe | *AET2Gv20884100.1* | N |
| *TraesCS2D01G392400.1* | B3 domain-containing protein | *AET2Gv20880200.1* | [105] |
| *TraesCS2D01G395500.1* | Ankyrin repeat protein-like | *AET2Gv20886300.1* | [106] |
| *TraesCS2D01G395800.1* | Ankyrin repeat and zinc finger domain-containing protein 1 | *AET2Gv20886600.1* | [106] |
| *TraesCS2D01G389600.1* | Alpha/beta-Hydrolases superfamily protein, putative | *AET2Gv20874100.1* | [107] |
| *TraesCS2D01G392300.1* | Alpha/beta-Hydrolases superfamily protein | *AET2Gv20880000.1* | [107] |
| *TraesCS2D01G386700.1* | Aldehyde dehydrogenase | *AET2Gv20871400.1* | [108] |
| *TraesCS2D01G380000.1* | Actin family protein | *AET2Gv20854500.1* | [109] |
| *TraesCS2D01G383100.1* | Acidic leucine-rich nuclear phosphoprotein 32 family B protein | *AET2Gv20860500.1* | [110] |
| *TraesCS2D01G393800.1* | ABC2 homolog 4 | *AET2Gv20883600.1* | [111] |
| *TraesCS2D01G395600.1* | 4-hydroxy-tetrahydrodipicolinate synthase | *AET2Gv20886400.1* | N |
| *TraesCS2D01G379400.1* | 3-hydroxyacyl-[acyl-carrier-protein] dehydratase FabZ | *AET2Gv20853700.1* | N |
| *TraesCS2D01G394200.1* | 1-aminocyclopropane-1-carboxylate synthase | *AET2Gv20884600.1* | [112] |
| *TraesCS2D01G376700.1* |  | N |  |
| *TraesCS2D01G376800.1* |  | N |  |
| *TraesCS2D01G376900.1* |  | N |  |
| *TraesCS2D01G377000.1* |  | N |  |
| *TraesCS2D01G377100.1* |  | N |  |
| *TraesCS2D01G377500.1* |  | N |  |
| *TraesCS2D01G377600.1* |  | N |  |
| *TraesCS2D01G378400.1* |  | N |  |
| *TraesCS2D01G378500.1* |  | N |  |
| *TraesCS2D01G379100.1* |  | N |  |
| *TraesCS2D01G379500.1* |  | N |  |
| *TraesCS2D01G379700.1* |  | N |  |
| *TraesCS2D01G379800.1* |  | N |  |
| *TraesCS2D01G379900.1* |  | N |  |
| *TraesCS2D01G380100.1* |  | N |  |
| *TraesCS2D01G380600.1* |  | N |  |
| *TraesCS2D01G380700.1* |  | N |  |
| *TraesCS2D01G380800.1* |  | N |  |
| *TraesCS2D01G381100.1* |  | N |  |
| *TraesCS2D01G381200.1* |  | N |  |
| *TraesCS2D01G381700.1* |  | N |  |
| *TraesCS2D01G381900.1* |  | N |  |
| *TraesCS2D01G382000.1* |  | N |  |
| *TraesCS2D01G382100.1* |  | N |  |
| *TraesCS2D01G382400.1* |  | N |  |
| *TraesCS2D01G382600.1* |  | N |  |
| *TraesCS2D01G383000.1* |  | N |  |
| *TraesCS2D01G383300.1* |  | N |  |
| *TraesCS2D01G383500.1* |  | N |  |
| *TraesCS2D01G384000.1* |  | N |  |
| *TraesCS2D01G384100.1* |  | N |  |
| *TraesCS2D01G384200.1* |  | N |  |
| *TraesCS2D01G384300.1* |  | N |  |
| *TraesCS2D01G384600.1* |  | N |  |
| *TraesCS2D01G384700.1* |  | N |  |
| *TraesCS2D01G384900.1* |  | N |  |
| *TraesCS2D01G385400.1* |  | N |  |
| *TraesCS2D01G385700.1* |  | N |  |
| *TraesCS2D01G386500.1* |  | N |  |
| *TraesCS2D01G387000.1* |  | N |  |
| *TraesCS2D01G387600.1* |  | N |  |
| *TraesCS2D01G387700.1* |  | N |  |
| *TraesCS2D01G387800.1* |  | N |  |
| *TraesCS2D01G387900.1* |  | N |  |
| *TraesCS2D01G388600.1* |  | N |  |
| *TraesCS2D01G388700.1* |  | N |  |
| *TraesCS2D01G389000.1* |  | N |  |
| *TraesCS2D01G389300.1* |  | N |  |
| *TraesCS2D01G389400.1* |  | N |  |
| *TraesCS2D01G389500.1* |  | N |  |
| *TraesCS2D01G390100.1* |  | N |  |
| *TraesCS2D01G391100.1* |  | N |  |
| *TraesCS2D01G391300.1* |  | N |  |
| *TraesCS2D01G392000.1* |  | N |  |
| *TraesCS2D01G392700.1* |  | N |  |
| *TraesCS2D01G393100.1* |  | N |  |
| *TraesCS2D01G393300.1* |  | N |  |
| *TraesCS2D01G393600.1* |  | N |  |
| *TraesCS2D01G394400.1* |  | N |  |
| *TraesCS2D01G394700.1* |  | N |  |
| *TraesCS2D01G395100.1* |  | N |  |
| *TraesCS2D01G395300.1* |  | N |  |
| *TraesCS2D01G395400.1* |  | N |  |
| *TraesCS2D01G396300.1* |  | N |  |
| *TraesCS2D01G397300.1* |  | N |  |
| *TraesCS2D01G397400.1* |  | N |  |
| *TraesCS2D01G398000.1* |  | N |  |
| *TraesCS2D01G398100.1* |  | N |  |
| *TraesCS2D01G398200.1* |  | N |  |
| *TraesCS2D01G398300.1* |  | N |  |
| *TraesCS2D01G398400.1* |  | N |  |
| *TraesCS2D01G398500.1* |  | N |  |
| N |  | *AET2Gv20850800.1* |  |
| N |  | *AET2Gv20850900.1* |  |
| N |  | *AET2Gv20852000.1* |  |
| N |  | *AET2Gv20852400.1* |  |
| N |  | *AET2Gv20852500.1* |  |
| N |  | *AET2Gv20852600.1* |  |
| N |  | *AET2Gv20854000.1* |  |
| N |  | *AET2Gv20854800.1* |  |
| N |  | *AET2Gv20857200.1* |  |
| N |  | *AET2Gv20864100.1* |  |
| N |  | *AET2Gv20867600.1* |  |
| N |  | *AET2Gv20867700.1* |  |
| N |  | *AET2Gv20867900.1* |  |
| N |  | *AET2Gv20868700.1* |  |
| N |  | *AET2Gv20869100.1* |  |
| N |  | *AET2Gv20870800.1* |  |
| N |  | *AET2Gv20871800.1* |  |
| N |  | *AET2Gv20872700.1* |  |
| N |  | *AET2Gv20872800.1* |  |
| N |  | *AET2Gv20873400.1* |  |
| N |  | *AET2Gv20875000.1* |  |
| N |  | *AET2Gv20875300.1* |  |
| N |  | *AET2Gv20875800.1* |  |
| N |  | *AET2Gv20877300.1* |  |
| N |  | *AET2Gv20877500.1* |  |
| N |  | *AET2Gv20877600.1* |  |
| N |  | *AET2Gv20878900.1* |  |
| N |  | *AET2Gv20879400.1* |  |
| N |  | *AET2Gv20879600.1* |  |
| N |  | *AET2Gv20879700.1* |  |
| N |  | *AET2Gv20881000.1* |  |
| N |  | *AET2Gv20881100.1* |  |
| N |  | *AET2Gv20881700.1* |  |
| N |  | *AET2Gv20882100.1* |  |
| N |  | *AET2Gv20882600.1* |  |
| N |  | *AET2Gv20883800.1* |  |
| N |  | *AET2Gv20883900.1* |  |
| N |  | *AET2Gv20884500.1* |  |
| N |  | *AET2Gv20884900.1* |  |
| N |  | *AET2Gv20886100.1* |  |
| N |  | *AET2Gv20887300.1* |  |
| N |  | *AET2Gv20887900.1* |  |
| N |  | *AET2Gv20889000.1* |  |
| N |  | *AET2Gv20890700.1* |  |
| N |  | *AET2Gv20891300.1* |  |
| N |  | *AET2Gv20891400.1* |  |
| N |  | *AET2Gv20891500.1* |  |
| N |  | *AET2Gv20891700.1* |  |
| N |  | *AET2Gv20891900.1* |  |
| N |  | *AET2Gv20892000.1* |  |
| N |  | *AET2Gv20892200.1* |  |
| N |  | *AET2Gv20892300.1* |  |
| N |  | *AET2Gv20892400.1* |  |
| N |  | *AET2Gv20892600.1* |  |
| N |  | *AET2Gv20892800.1* |  |
| N |  | *AET2Gv20858500.1* |  |
| N |  | *AET2Gv20859500.1* |  |
| N |  | *AET2Gv20859600.1* |  |
| N |  | *AET2Gv20859900.1* |  |
| N |  | *AET2Gv20860000.1* |  |
| N |  | *AET2Gv20860800.1* |  |
| N |  | *AET2Gv20860900.1* |  |
| N |  | *AET2Gv20861100.1* |  |
| N |  | *AET2Gv20861800.1* |  |
| N |  | *AET2Gv20862100.1* |  |
| N |  | *AET2Gv20862900.1* |  |
| N |  | *AET2Gv20863100.1* |  |
| N |  | *AET2Gv20893200.1* |  |
| N |  | *AET2Gv20893300.1* |  |
| N |  | *AET2Gv20893400.1* |  |
| N |  | *AET2Gv20893500.1* |  |
| N |  | *AET2Gv20893600.1* |  |
| N |  | *AET2Gv20893800.1* |  |
| N |  | *AET2Gv20893900.1* |  |
| N |  | *AET2Gv20858200.1* |  |
| N |  | *AET2Gv20858400.1* |  |

Note: N represents no orthologs were identified after reciprocal blasting analysis.

**References**

[1] Beom C, Yeon K, Kesavan M, et al. GW2 Functions as an E3 Ubiquitin Ligase for Rice Expansin-Like 1. International Journal of Molecular ences, 2018, 19(7):1904.

[2] Li J, Jiang J, Qian Q, Xu Y, Zhang C, Xiao J, Du C, Luo W, Zou G, Chen M, Huang Y, Feng Y, Cheng Z, Yuan M, Chong K. Mutation of rice BC12/GDD1, which encodes a kinesin-like protein that binds to a GA biosynthesis gene promoter, leads to dwarfism with impaired cell elongation. Plant Cell. 2011 Feb;23(2):628-40.

[3] Jain M, Nijhawan A, Arora R, et al. F-box proteins in rice. Genome-wide analysis, classification, temporal and spatial gene expression during panicle and seed development, and regulation by light and abiotic stress. Plant Physiology, 2007, 143(4): 1467-1483.

[4] Cristina M S, Petersen M, Mundy J. Mitogen-activated protein kinase signaling in plants. Annual Review of Plant Biology, 2010, 61: 621-649.

[5] Tamura K, Shimada T, Kondo M, et al. KATAMARI1/MURUS3 is a novel Golgi membrane protein that is required for endomembrane organization in Arabidopsis. The Plant Cell, 2005, 17(6): 1764-1776.

[6] Jain M, Tyagi A K, Khurana J P. Genome-wide analysis, evolutionary expansion, and expression of early auxin-responsive SAUR gene family in rice (Oryza sativa). Genomics, 2006, 88(3): 360-371.

[7] Andersen P, Kragelund B B, Olsen A N, et al. Structure and biochemical function of a prototypical Arabidopsis U-box domain. Journal of Biological Chemistry, 2004, 279(38): 40053-40061.

[8] Hollick J B, Chandler V L. Genetic factors required to maintain repression of a paramutagenic maize pl1 allele. Genetics, 2001, 157(1): 369-378.

[9] Wang, Zafian, Choudhary, et al. The PR5K receptor protein kinase from Arabidopsis thaliana is structurally related to a family of plant defense proteins. Proceedings of the National Academy of Sciences of the United States of America, 1996.

[10] Bentolila S, Alfonso A A, Hanson M R. A pentatricopeptide repeat-containing gene restores fertility to cytoplasmic male-sterile plants. Proceedings of the National Academy of Sciences, 2002, 99(16): 10887-10892.

[11] Feiler H S, Jacobs T W. Cell division in higher plants: a cdc2 gene, its 34-kDa product, and histone H1 kinase activity in pea. Proceedings of the National Academy of Sciences, 1990, 87(14): 5397-5401.

[12] Jaglo K R, Kleff S, Amundsen K L, et al. Components of the Arabidopsis C-repeat/dehydration-responsive element binding factor cold-response pathway are conserved inbrassica napus and other plant species. Plant Physiology, 2001, 127(3): 910-917.

[13] Farrow S C, Facchini P J. Functional diversity of 2-oxoglutarate/Fe (II)-dependent dioxygenases in plant metabolism. Frontiers in Plant Science, 2014, 5: 524.

[14] Zhu Q , Dabi T , Beeche A , et al. Cloning and properties of a rice gene encoding phenylalanine ammonia-lyase. Plant Molecular Biology, 1995, 29(3):535-550.

[15] Ma L, Li G. Far1-related sequence (FRS) and Frs-related factor (FRF) family proteins in arabidopsis growth and development. Frontiers in Plant Science, 2018, 9: 692.

[16] Khanna R, Kronmiller B, Maszle D R, et al. The Arabidopsis B-box zinc finger family. The Plant Cell, 2009, 21(11): 3416-3420.

[17] Simon M K, Skinner D J, Gallagher T L, et al. Integument development in Arabidopsis depends on interaction of YABBY protein INNER NO OUTER with coactivators and corepressors. Genetics, 2017, 207(4): 1489-1500.

[18] Pandey S P, Somssich I E. The role of WRKY transcription factors in plant immunity. Plant Physiology, 2009, 150(4): 1648-1655.

[19] Hara-Nishimura I, Hatsugai N, Nakaune S, et al. Vacuolar processing enzyme: an executor of plant cell death. Current Opinion in Plant Biology, 2005, 8(4): 404-408.

[20] Neuhaus J M, Rogers J C. Sorting of proteins to vacuoles in plant cells[M]//Protein Trafficking in Plant Cells. Springer, Dordrecht, 1998: 127-144.

[21] Zhang Y, Xu Y H, Yi H Y, et al. Vacuolar membrane transporters OsVIT1 and OsVIT2 modulate iron translocation between flag leaves and seeds in rice. The Plant Journal, 2012, 72(3): 400-410.

[22] Wang D H, Song W, Wei S W, et al. Characterization of the ubiquitin C-terminal hydrolase and ubiquitin-specific protease families in rice (Oryza sativa). Frontiers in Plant Science, 2018, 9: 1636.

[23] Huang Xianzhong, Wei Lingzhu, Ma Zhengqiang. Cloning and Characterization of a Ubiquitin Fusion Degradation Protein Gene in Wheat. British Medical Journal, 2004, 294(6574):744-746.

[24] Wang X H, Li Q T, Chen H W, et al. Trihelix transcription factor GT-4 mediates salt tolerance via interaction with TEM2 in Arabidopsis. BMC Plant Biology, 2014, 14(1): 339.

[25] Vert G, Grotz N, Dédaldéchamp F, et al. IRT1, an Arabidopsis transporter essential for iron uptake from the soil and for plant growth. The Plant Cell, 2002, 14(6): 1223-1233.

[26] Dibb-Fuller J E, Morris D A. Studies on the evolution of auxin carriers and phytotropin receptors: transmembrane auxin transport in unicellular and multicellular Chlorophyta. Planta, 1992, 186(2): 219-226.

[27] Zhang Z L, Xie Z, Zou X, et al. A rice WRKY gene encodes a transcriptional repressor of the gibberellin signaling pathway in aleurone cells. Plant Physiology, 2004, 134(4): 1500-1513.

[28] Chen L, Shen D, Sun N, et al. Phytophthora sojae TatD nuclease positively regulates sporulation and negatively regulates pathogenesis. Molecular Plant-Microbe Interactions, 2014, 27(10): 1070-1080.

[29] Gupta A S, Heinen J L, Holaday A S, et al. Increased resistance to oxidative stress in transgenic plants that overexpress chloroplastic Cu/Zn superoxide dismutase. Proceedings of the National Academy of Sciences, 1993, 90(4): 1629-1633.

[30] Tanaka H, Onouchi H, Kondo M, et al. A subtilisin-like serine protease is required for epidermal surface formation in Arabidopsis embryos and juvenile plants. Development, 2001, 128(23): 4681-4689.

[31] Vahisalu T, Kollist H, Wang Y F, et al. SLAC1 is required for plant guard cell S-type anion channel function in stomatal signalling. Nature, 2008, 452(7186): 487-491.

[32] Duan K, Yi K, Dang L, et al. Characterization of a sub‐family of Arabidopsis genes with the SPX domain reveals their diverse functions in plant tolerance to phosphorus starvation. The Plant Journal, 2008, 54(6): 965-975.

[33] Imai H, Nishiura H. Phosphorylation of sphingoid long-chain bases in Arabidopsis: functional characterization and expression of the first sphingoid long-chain base kinase gene in plants. Plant and Cell Physiology, 2005, 46(2): 375-380.

[34] Kim H H, Tharayil M, Rudd C E. Growth factor receptor-bound protein 2 SH2/SH3 domain binding to CD28 and its role in co-signaling. Journal of Biological Chemistry, 1998, 273(1): 296-301.

[35] Liang C, Wang Y, Zhu Y, et al. OsNAP connects abscisic acid and leaf senescence by fine-tuning abscisic acid biosynthesis and directly targeting senescence-associated genes in rice. Proceedings of the National Academy of Sciences, 2014, 111(27): 10013-10018.

[36] Joshi C P, Chiang V L. Conserved sequence motifs in plant S-adenosyl-L-methionine-dependent methyltransferases. Plant Molecular Biology, 1998, 37(4): 663-674.

[37] Sami-Subbu R, Choi S B, Wu Y, et al. Identification of a cytoskeleton-associated 120 kDa RNA-binding protein in developing rice seeds. Plant Molecular Biology, 2001, 46(1): 79-88.

[38] Andersen P, Kragelund B B, Olsen A N, et al. Structure and biochemical function of a prototypical Arabidopsis U-box domain. Journal of Biological Chemistry, 2004, 279(38): 40053-40061.

[39] Lee H, Xiong L, Gong Z, et al. The Arabidopsis HOS1 gene negatively regulates cold signal transduction and encodes a RING finger protein that displays cold-regulated nucleo–cytoplasmic partitioning. Genes & development, 2001, 15(7): 912-924.

[40] Lim S D, Yim W C, Moon J C, et al. A gene family encoding RING finger proteins in rice: their expansion, expression diversity, and co-expressed genes. Plant Molecular Biology, 2010, 72(4-5): 369-380.

[41] Wu G, Li H, Yang Z. Arabidopsis RopGAPs are a novel family of rho GTPase-activating proteins that require the Cdc42/Rac-interactive binding motif for rop-specific GTPase stimulation. Plant Physiology, 2000, 124(4): 1625-1636.

[42] Pla M, Gómez J, Goday A, et al. Regulation of the abscisic acid-responsive gene rab28 in maize viviparous mutants. Molecular and General Genetics MGG, 1991, 230(3): 394-400.

[43] Ingouff M, Farbos I, Lagercrantz U, et al. PaHB1 is an evolutionary conserved HD‐GL2 homeobox gene expressed in the protoderm during Norway spruce embryo development. Genesis, 2001, 30(4): 220-230.

[44] Nakajima K, Furutani I, Tachimoto H, et al. SPIRAL1 encodes a plant-specific microtubule-localized protein required for directional control of rapidly expanding Arabidopsis cells. The Plant Cell, 2004, 16(5): 1178-1190.

[45] Ge L , Peer W , Robert S , et al. Arabidopsis ROOT UVB SENSITIVE2/WEAK AUXIN RESPONSE1Is Required for Polar Auxin Transport. Plant Cell, 2010, 22(6):1749-1761.

[46] Konishi M, Sugiyama M. A novel plant-specific family gene, ROOT PRIMORDIUM DEFECTIVE 1, is required for the maintenance of active cell proliferation. Plant Physiology, 2006, 140(2): 591-602.

[47] Li L, He Z, Pandey G K, et al. Functional cloning and characterization of a plant efflux carrier for multidrug and heavy metal detoxification. Journal of Biological Chemistry, 2002, 277(7): 5360-5368.

[48] Li Y, Qian Q, Zhou Y, et al. BRITTLE CULM1, which encodes a COBRA-like protein, affects the mechanical properties of rice plants. The Plant Cell, 2003, 15(9): 2020-2031.

[49] Lafarge S, Montané M H. Characterization of Arabidopsis thaliana ortholog of the human breast cancer susceptibility gene 1: AtBRCA1, strongly induced by gamma rays. Nucleic Acids Research, 2003, 31(4): 1148-1155.

[50] Kawamura Y, Asai K, Ishii S, et al. Systematic analyses of P-loop containing nucleotide triphosphate hydrolase superfamily based on sequence, structure and function. Genome Informatics, 2003, 14: 581-582.

[51] Tang D, Ade J, Frye C A, et al. Regulation of plant defense responses in Arabidopsis by EDR2, a PH and START domain‐containing protein. The Plant Journal, 2005, 44(2): 245-257.

[52] Gao Z P, Yu Q B, Zhao T T, et al. A functional component of the transcriptionally active chromosome complex, Arabidopsis pTAC14, interacts with pTAC12/HEMERA and regulates plastid gene expression. Plant Physiology, 2011, 157(4): 1733-1745.

[53] Zhu Q , Dabi T , Beeche A , et al. Cloning and properties of a rice gene encoding phenylalanine ammonia-lyase. Plant Molecular Biology, 1995, 29(3):535-550.

[54] Petutschnig E K, Jones A M E, Serazetdinova L, et al. The lysin motif receptor-like kinase (LysM-RLK) CERK1 is a major chitin-binding protein in Arabidopsis thaliana and subject to chitin-induced phosphorylation. Journal of Biological Chemistry, 2010, 285(37): 28902-28911.

[55] Sarowar S, Kim Y J, Kim E N, et al. Overexpression of a pepper basic pathogenesis-related protein 1 gene in tobacco plants enhances resistance to heavy metal and pathogen stresses. Plant Cell Reports, 2005, 24(4): 216-224.

[56] Yilmaz J L, Bülow L. Enhanced stress tolerance in Escherichia coli and Nicotiana tabacum expressing a betaine aldehyde dehydrogenase/choline dehydrogenase fusion protein. Biotechnology Progress, 2002, 18(6): 1176-1182.

[57] Shri M, Kumar S, Chakrabarty D, et al. Effect of arsenic on growth, oxidative stress, and antioxidant system in rice seedlings. Ecotoxicology and Environmental Safety, 2009, 72(4): 1102-1110.

[58] Zhao Q, Leung S, Corbett A H, et al. Identification and characterization of the Arabidopsis orthologs of nuclear transport factor 2, the nuclear import factor of ran. Plant Physiology, 2006, 140(3): 869-878.

[59] Diédhiou C J, Popova O V, Dietz K J, et al. The SNF1-type serine-threonine protein kinase SAPK4 regulates stress-responsive gene expression in rice. BMC Plant Biology, 2008, 8(1): 49.

[60] Jaiswal A K. Regulation of genes encoding NAD (P) H: quinone oxidoreductases. Free Radical Biology and Medicine, 2000, 29(3-4): 254-262.

[61] Yoo S Y, Kim Y, Kim S Y, et al. Control of flowering time and cold response by a NAC-domain protein in Arabidopsis. PloS one, 2007, 2(7).

[62] Suzuki N, Rizhsky L, Liang H, et al. Enhanced tolerance to environmental stress in transgenic plants expressing the transcriptional coactivator multiprotein bridging factor 1c. Plant Physiology, 2005, 139(3): 1313-1322.

[63] Chew O, Lister R, Qbadou S, et al. A plant outer mitochondrial membrane protein with high amino acid sequence identity to a chloroplast protein import receptor. FEBS Letters, 2004, 557(1-3): 109-114.

[64] Sarma R, Barney B M, Hamilton T L, et al. Crystal structure of the L protein of Rhodobacter sphaeroides light-independent protochlorophyllide reductase with MgADP bound: a homologue of the nitrogenase Fe protein. Biochemistry, 2008, 47(49): 13004-13015.

[65] Li X, Sun L, Tan L, et al. TH1, a DUF640 domain-like gene controls lemma and palea development in rice. Plant Molecular Biology, 2012, 78(4-5): 351-359.

[66] Monschau N, Sahm H, Stahmann K P. Threonine aldolase overexpression plus threonine supplementation enhanced riboflavin production inAshbya gossypii. Appl. Environ. Microbiol., 1998, 64(11): 4283-4290.

[67] Corbin D R, Sauer N, Lamb C J. Differential regulation of a hydroxyproline-rich glycoprotein gene family in wounded and infected plants. Molecular and Cellular Biology, 1987, 7(12): 4337-4344.

[68] Xu D, Shi J, Rautengarten C, et al. Defective Pollen Wall 2 (DPW2) encodes an acyl transferase required for rice pollen development. Plant Physiology, 2017, 173(1): 240-255.

[69] Prasad B D, Goel S, Krishna P. In silico identification of carboxylate clamp type tetratricopeptide repeat proteins in Arabidopsis and rice as putative co-chaperones of Hsp90/Hsp70. PloS one, 2010, 5(9).

[70] Matsuoka M, Ichikawa H, Saito A, et al. Expression of a rice homeobox gene causes altered morphology of transgenic plants. The Plant Cell, 1993, 5(9): 1039-1048.

[71] Zhong R, Ye Z H. IFL1, a gene regulating interfascicular fiber differentiation in Arabidopsis, encodes a homeodomain-leucine zipper protein. The Plant Cell, 1999, 11(11): 2139-2152.

[72] Ding Y, Wang X, Su L, et al. SDG714, a histone H3K9 methyltransferase, is involved in Tos17 DNA methylation and transposition in rice. The Plant Cell, 2007, 19(1): 9-22.

[73] Song X J, Kuroha T, Ayano M, et al. Rare allele of a previously unidentified histone H4 acetyltransferase enhances grain weight, yield, and plant biomass in rice. Proceedings of the National Academy of Sciences, 2015, 112(1): 76-81.

[74] Brenner C, Bieganowski P, Pace H C, et al. The histidine triad superfamily of nucleotide‐binding proteins. Journal of Cellular Physiology, 1999, 181(2): 179-187.

[75] Cohen C K, Fox T C, Garvin D F, et al. The role of iron-deficiency stress responses in stimulating heavy-metal transport in plants. Plant Physiology, 1998, 116(3): 1063-1072.

[76] Kim J H, Tsukaya H. Regulation of plant growth and development by the GROWTH-REGULATING FACTOR and GRF-INTERACTING FACTOR duo. Journal of Experimental Botany, 2015, 66(20): 6093-6107.

[77] Xu K, Chen S, Li T, et al. OsGRAS23, a rice GRAS transcription factor gene, is involved in drought stress response through regulating expression of stress-responsive genes. BMC Plant Biology, 2015, 15(1): 141.

[78] Kumar R G, Shah K, Dubey R S. Salinity induced behavioural changes in malate dehydrogenase and glutamate dehydrogenase activities in rice seedlings of differing salt tolerance. Plant Science, 2000, 156(1): 23-34.

[79] Luo X M, Lin W H, Zhu S, et al. Integration of light-and brassinosteroid-signaling pathways by a GATA transcription factor in Arabidopsis. Developmental Cell, 2010, 19(6): 872-883.

[80] Banno H, Chua N H. Characterization of the Arabidopsis formin-like protein AFH1 and its interacting protein. Plant and Cell Physiology, 2000, 41(5): 617-626.

[81] CAKmAK I, van de Wetering D A M, Marschner H, et al. Involvement of superoxide radical in extracellular ferric reduction by iron-deficient bean roots. Plant Physiology, 1987, 85(1): 310-314.

[82] Stefanowicz K, Lannoo N, Van Damme E J M. Plant F-box proteins–judges between life and death. Critical Reviews in Plant Sciences, 2015, 34(6): 523-552.

[83] Kandel S, Sauveplane V, Olry A, et al. Cytochrome P450-dependent fatty acid hydroxylases in plants. Phytochemistry Reviews, 2006, 5(2-3): 359-372.

[84] Li J, Yu M, Geng L L, et al. The fasciclin‐like arabinogalactan protein gene, FLA3, is involved in microspore development of Arabidopsis. The Plant Journal, 2010, 64(3): 482-497.

[85] Albar L, Bangratz‐Reyser M, Hébrard E, et al. Mutations in the eIF (iso) 4G translation initiation factor confer high resistance of rice to Rice yellow mottle virus. The Plant Journal, 2006, 47(3): 417-426.

[86] Ohta M, Ohme‐Takagi M, Shinshi H. Three ethylene‐responsive transcription factors in tobacco with distinct transactivation functions. The Plant Journal, 2000, 22(1): 29-38.

[87] Fukayama H, Masumoto C, Taniguchi Y, et al. Characterization and expression analyses of two plastidic enolase genes in rice. Bioscience, Biotechnology, and Biochemistry, 2015, 79(3): 402-409.

[88] Niewiadomski P, Knappe S, Geimer S, et al. The Arabidopsis plastidic glucose 6-phosphate/phosphate translocator GPT1 is essential for pollen maturation and embryo sac development. The Plant Cell, 2005, 17(3): 760-775.

[89] Albrecht V, Ingenfeld A, Apel K. Characterization of the snowy cotyledon 1 mutant of Arabidopsis thaliana: the impact of chloroplast elongation factor G on chloroplast development and plant vitality. Plant Molecular Biology, 2006, 60(4): 507-518.

[90] Hoefle C, Hückelhoven R. A barley Engulfment and Motility domain containing protein modulates Rho GTPase activating protein HvMAGAP1 function in the barley powdery mildew interaction. Plant Molecular Biology, 2014, 84(4-5): 469-478.

[91] Lv M, Hou D, Zhang L, et al. Molecular characterization and function analysis of the rice OsDUF1191 family. Biotechnology & Biotechnological Equipment, 2019, 33(1): 1608-1615.

[92] Gassmann W, Hinsch M E, Staskawicz B J. The Arabidopsis RPS4 bacterial‐resistance gene is a member of the TIR‐NBS‐LRR family of disease‐resistance genes. The Plant Journal, 1999, 20(3): 265-277.

[93] Sakakibara Y, Kobayashi H, Kasamo K. Isolation and characterization of cDNAs encoding vacuolar H+-pyrophosphatase isoforms from rice (Oryza sativa L.). Plant Molecular Biology, 1996, 31(5): 1029-1038.

[94] Tian L, Musetti V, Kim J, et al. The Arabidopsis LUT1 locus encodes a member of the cytochrome P450 family that is required for carotenoid ε-ring hydroxylation activity. Proceedings of the National Academy of Sciences, 2004, 101(1): 402-407.

[95] Ederli L, Madeo L, Calderini O, et al. The Arabidopsis thaliana cysteine-rich receptor-like kinase CRK20 modulates host responses to Pseudomonas syringae pv. tomato DC3000 infection. Journal of Plant Physiology, 2011, 168(15): 1784-1794.

[96] Turowski V R, Busi M V, Gomez-Casati D F. Structural and functional studies of the mitochondrial cysteine desulfurase from Arabidopsis thaliana. Molecular Plant, 2012, 5(5): 1001-1010.

[97] Wang G, Kong H, Sun Y, et al. Genome-wide analysis of the cyclin family in Arabidopsis and comparative phylogenetic analysis of plant cyclin-like proteins. Plant Physiology, 2004, 135(2): 1084-1099.

[98] Gassmann W, Hinsch M E, Staskawicz B J. The Arabidopsis RPS4 bacterial‐resistance gene is a member of the TIR‐NBS‐LRR family of disease‐resistance genes. The Plant Journal, 1999, 20(3): 265-277.

[99] Lu Q, Ding S, Reiland S, et al. Identification and characterization of chloroplast casein kinase II from Oryza sativa (rice). Journal of Experimental Botany, 2015, 66(1): 175-187.

[100] Chen J C F, Tsai C C Y, Tzen J T C. Cloning and secondary structure analysis of caleosin, a unique calcium-binding protein in oil bodies of plant seeds. Plant and Cell Physiology, 1999, 40(10): 1079-1086.

[101] Cho E A, Prindle M J, Dressler G R. BRCT domain-containing protein PTIP is essential for progression through mitosis. Molecular and Cellular Biology, 2003, 23(5): 1666-1673.

[102] Nersissian A M, Valentine J S, Immoos C, et al. Uclacyanins, stellacyanins, and plantacyanins are distinct subfamilies of phytocyanins: Plant‐specific mononuclear blue copper proteins. Protein Science, 1998, 7(9): 1915-1929.

[103] G Simos, C A Panagiotidis, A Skoumbas, et, al. Barley beta-glucosidase: expression during seed germination and maturation and partial amino acid sequences.. Biochimica Et Biophysica Acta, 1994, 1199(1):52.

[104] Rissler H M, Pogson B J. Antisense inhibition of the beta-carotene hydroxylase enzyme in Arabidopsis and the implications for carotenoid accumulation, photoprotection and antenna assembly. Photosynthesis Research, 2001, 67(1-2): 127-137.

[105] Luerßen H, Kirik V, Herrmann P, et al. FUSCA3 encodes a protein with a conserved VP1/ABI3‐like B3 domain which is of functional importance for the regulation of seed maturation in Arabidopsis thaliana. The Plant Journal, 1998, 15(6): 755-764.

[106] Becerra C, Jahrmann T, Puigdomènech P, et al. Ankyrin repeat-containing proteins in Arabidopsis: characterization of a novel and abundant group of genes coding ankyrin-transmembrane proteins. Gene, 2004, 340(1): 111-121.

[107] Dimitriou P S, Denesyuk A, Takahashi S, et al. Alpha/beta‐hydrolases: a unique structural motif coordinates catalytic acid residue in 40 protein fold families. Proteins: Structure, Function, and Bioinformatics, 2017, 85(10): 1845-1855.

[108] Sunkar R, Bartels D, Kirch H H. Overexpression of a stress‐inducible aldehyde dehydrogenase gene from Arabidopsis thaliana in transgenic plants improves stress tolerance. The Plant Journal, 2003, 35(4): 452-464.

[109] McElroy D, Rothenberg M, Reece K S, et al. Characterization of the rice (Oryza sativa) actin gene family. Plant Molecular Biology, 1990, 15(2): 257-268.

[110] Macovei A, Vaid N, Tula S, et al. A new DEAD-box helicase ATP-binding protein (OsABP) from rice is responsive to abiotic stress. Plant Signaling & Behavior, 2012, 7(9): 1138-1143.

[111] Sánchez-Fernández R, Davies T G E, Coleman J O D, et al. The Arabidopsis thaliana ABC protein superfamily, a complete inventory. Journal of Biological Chemistry, 2001, 276(32): 30231-30244.

[111] Liang X, Abel S, Keller J A, et al. The 1-aminocyclopropane-1-carboxylate synthase gene family of Arabidopsis thaliana. Proceedings of the National Academy of Sciences, 1992, 89(22): 11046-11050.
