# Supplementary material for: Genetic Mapping and Validation of Loci for Kernel-Related Traits in Wheat (Triticum aestivum L.)
Source: Front Plant Sci. 2021 Jun 7;12:667493. doi: 10.3389/fpls.2021.667493 (PMC8215603; doi:10.3389/fpls.2021.667493)
Supplement: Supplementary Table 10 — Comparison of the major quantitative trait loci (QTL) for kernel-related traits identified in this study with previous studies. [file Table_10.DOCX]

**Table S10** Comparison of the major quantitative trait loci (QTL) for kernel-related traits identified in this study with previous studies

| **Triat** | **Gene/QTL** | **chromosome arm** | **Left marker information** | | **Right marker information** | | **Reference** |
| --- | --- | --- | --- | --- | --- | --- | --- |
|  |  |  | **Physical position** | **ID** | **Physical position** | **ID** |  |
| **KL** |  | 1BL | 94832296 | *IWA3092* | 409496782 | *BS00010625_51* | [1] |
|  | *QKl-1B* | 1BL | N | *wPt-2315* | 637368430 | *Xwmc766* | [2] |
|  | *QKl.sdau-1B.1* | 1B | N | *Xswes15* | N | *Xubc811b* | [3] |
|  | *QKl.sdau-1B.2* | 1B | N | *Xubc856a* | N | *Xswesa69b* | [3] |
|  | *QKl-1BL.1* | 1BL | *19040394* | *Xmwg68.2* | 111026591 | *wPt-9857 wPt-0260* | [4] |
|  |  | 1BS | 73135128 | *Xgwm413* |  |  | [5] |
|  |  | 1BL | 459137504 | IWA3092 | 551177487 | BS00010625_51 | [6] |
|  | *QGl.ccsu-1B.1* | 1BL | 431653786 | *Xgwm413* | 454599095 | *Xwmc419* | [7] |
|  |  | 1BS | 101590746 | *gpw7383* | N | *wPt7273* | [8] |
|  | ***QKL.sicau-2SY-1B*** | **1BL** | **566604700** | ***AX-94433089*** | **583631139** | ***AX-109379070*** | **This study** |
| **KW** | *QKw.nwafu-6D.1* | 6DL | 464846150 | *barc21* | 456455996 | *GDM98* | [9] |
|  | *QKw.nwafu-6D.2* | 6D | N | *cfa5* | 14221399 | *GDM132* | [9] |
|  |  | 6DL | N | *wPt-3127* | 459178794 | *Xcfd45* | [5] |
|  | *QGwid.ccsu-6D.1* | 6DS | 62078025 | *Xcfd19c* | 79956573 | *Xgwm325b* | [7] |
|  | *qKW-6D* | 6DL | 418280403 | *Xbarc96* |  |  | [10] |
|  | ***QKW.sicau-2SY-6D*** | **6DS** | **45905000** | ***AX-94618881*** | **73372140** | ***AX-110066157*** | **This study** |
| **KT** |  | 2DL | 142336663 | *Xgwm30* | 267082590 | *Xwmc144* | [10] |
|  | ***QKT.sicau-2SY-2D*** | 2DL | **481592235** | ***AX-110012897*** | **512881951** | ***AX-110720701*** | **This study** |
| **TKW** | *QTkw‑2D.2* | 2DL | 608198902 | *Xmag3596* | N | *wPt-665317* | [4] |
|  |  | 2DL | N | *UBC815* |  |  | [11] |
|  | *QTgw.ipk-2D* | 2DL | 599941936 | *Xgdm6* |  |  | [12] |
|  | *QTgw.crc-2D.1* | 2DL | 332519419 | *wmc601* |  |  | [13] |
|  | *QTgw.crc-2D.2* | 2DL | 647509942 | *gwm382* |  |  | [13] |
|  | *QTgw.nfcri-2D* | 2DS | 115879168 | *xbarc228* | 580011701 | *xcfd168* | [14] |
|  | *QTGW* | 2DL | 577440659 | *wmc41* | 593738612 | *wmc181* | [15] |
|  |  | 2DS | 59311162 | *cfd62* | N | *mag4355b* | [15] |
|  |  | 2DS | 130832831 | *Xwmc18* |  |  | [16] |
|  | *QTkw-2D* | 2DL | 481601603 | *IWA4789* | 523150715 | *IWB53594* | [6] |
|  | *QGw.nau-2D* | 2DS | 19623155 | *GWM261* | 23024714 | *WMC112* | [17] |
|  | *QTkw-2D* | 2DL | 561157733 | *Xcfd233* | 574603546 | *Xgpw5215.1* | [2] |
|  |  | 2DS | 27981473 | *wPt-666223* | 38250344 | *gpw4473* | [18] |
|  | *QTgw.cau-2D* | 2DL | 18490000 | *SSR-2013* | 23760000 | *Xbarc11* | [19] |
|  | *QTgw.crc-2D* | 2DS | 17609164 | *Xgwm296* | 23024735 | *Xwmc112* | [12] |
|  | *qTGW2D* | 2D | N | *Me8Em11b* | N | *Me8Em11a* | [18] |
|  | *QTKW.sicau-2D* | 2DS | 32970798 | *AX-111096297* | 33735919 | *KASP-AX-94721936* | [19] |
|  |  | 2DS | 23024744 | *Xcfd53* |  | *Xbarc168* | [20] |
|  | *Qtkw2D-1* | 2DL | 513098578 | Xgwm539 |  | *Xcfd168* | [21] |
|  | **QTKW.sicau-2SY-2D** | 2DL | **481592235** | ***AX-110012897*** | **512881951** | ***AX-110720701*** | **This study** |

N: information could not be found.

**References**

[1] Mohler V , Albrecht T , Castell A , et al. Considering causal genes in the genetic dissection of kernel traits in common wheat. Journal of Applied Genetics, 2016, 57(4):1-10.

[2] Cui F, Fan X, Chen M, Zhang N, Zhao C, Zhang W, Han J, Ji J, Zhao X, Yang L, Zhao Z, Tong Y, Wang T, Li J. QTL detection for wheat kernel size and quality and the responses of these traits to low nitrogen stress. Theoretical and Applied Genetics, 129(3):469-84.

[3] Sun XY, Wu K, Zhao Y, Kong FM, Han GZ, Jiang HM, Huang XJ, Li R-J, Wang HG, Li SS. QTL analysis of kernel shape and weight using recombinant inbred lines in wheat. Euphytica, 165:615–624.

[4] Cui F, Zhao C, Ding A, et al. Construction of an integrative linkage map and QTL mapping of grain yield-related traits using three related wheat RIL populations. Theoretical and Applied Genetics, 2014, 127(3): 659-675.

[5] Williams K, Sorrells M E. Three-dimensional seed size and shape QTL in hexaploid wheat (Triticum aestivum L.) populations. Crop Science, 2014, 54(1): 98-110.

[6] Mohler V, Albrecht T, Castell A, et al. Considering causal genes in the genetic dissection of kernel traits in common wheat. Journal of Applied Genetics, 2016, 57(4): 467-476.

[7] Tyagi S, Mir R R, Balyan H S, et al. Interval mapping and meta-QTL analysis of grain traits in common wheat (Triticum aestivum L.). Euphytica, 2015, 201(3): 367-380.

[8] Zhou X, Ma J, Luo W, Jiang Y, Sun M, Yang Y, Jiang Q, Liu Y, Chen G, Wei Y, Zheng Y, Lan X. QTL Maping for Kernel Morphology Traits of Tibetan Semi-wild Wheat. Journal of Triticeae Crops 36:27-35.

[9] Li, Meixia, et al. "Quantitative trait loci analysis for kernel‐related characteristics in common wheat (Triticum aestivum L.)." Crop Science 55.4 (2015): 1485-1493.

[10] Chen W, Sun D, Yan X, et al. QTL analysis of wheat kernel traits, and genetic effects of qKW-6A on kernel width. Euphytica, 2019, 215(2): 11.

[11] FAmmiraju J S S, Dholakia B B, Santra D K, et al. Identification of inter simple sequence repeat (ISSR) markers associated with seed size in wheat. Theoretical and Applied Genetics, 2001, 102(5): 726-732.

[12] Huang X Q, Cöster H, Ganal M W, et al. Advanced backcross QTL analysis for the identification of quantitative trait loci alleles from wild relatives of wheat (Triticum aestivum L.). Theoretical and Applied Genetics, 2003, 106(8): 1379-1389.

[13] Cuthbert J L, Somers D J, Brûlé-Babel A L, et al. Molecular mapping of quantitative trait loci for yield and yield components in spring wheat (Triticum aestivum L.). Theoretical and Applied Genetics, 2008, 117(4): 595-608.

[14] Wang R X, Hai L, Zhang X Y, et al. QTL mapping for grain filling rate and yield-related traits in RILs of the Chinese winter wheat population Heshangmai× Yu8679. Theoretical and Applied Genetics, 2009, 118(2): 313-325.

[15] Zhang G, Wang Y, Guo Y, et al. Characterization and mapping of QTLs on chromosome 2D for grain size and yield traits using a mutant line induced by EMS in wheat. The Crop Journal, 2015, 3(2): 135-144.

[16] Breseghello F, Sorrells M E. QTL analysis of kernel size and shape in two hexaploid wheat mapping populations. Field Crops Research, 2007, 101(2): 172-179.

[17] Huang Y , Kong Z , Wu X , et al. Characterization of three wheat grain weight QTLs that differentially affect kernel dimensions. Theoretical and Applied Genetics, 2015, 128(12):2437-2445.

[18] Yu M, Mao S, Hou D, et al. Analysis of contributors to grain yield in wheat at the individual quantitative trait locus level. Plant Breeding, 2018, 137(1): 35-49.

[19] Ma J, Zhang H, Li S, et al. Identification of quantitative trait loci for kernel traits in a wheat cultivar Chuannong16. BMC Genetics, 2019, 20(1): 77.

[20] Heidari B, Sayed-Tabatabaei B E, Saeidi G, et al. Mapping QTL for grain yield, yield components, and spike features in a doubled haploid population of bread wheat. Genome, 2011, 54(6): 517-527.

[21] Zhang X, Deng Z, Wang Y, et al. Unconditional and conditional QTL analysis of kernel weight related traits in wheat (Triticum aestivum L.) in multiple genetic backgrounds. Genetica, 2014, 142(4): 371-379.
