# Supplementary material for: Genetic Mapping and Validation of Loci for Kernel-Related Traits in Wheat (Triticum aestivum L.)
Source: Front Plant Sci. 2021 Jun 7;12:667493. doi: 10.3389/fpls.2021.667493 (PMC8215603; doi:10.3389/fpls.2021.667493)
Supplement: Supplementary Table 11 — The physical location information of the detected major QTL. [file Table_11.DOCX]

**Table S11**. The physical location information of the detected major QTL.

| Chromosome | Physical location(Mbp) | QTL | Trait | Environment |  |
| --- | --- | --- | --- | --- | --- |
| 1BL | 566.6-583.6 | *QKL.sicau-2SY-1B* | KL | E1-E7 |  |
|  | 566.6-583.6 | *QKS.sicau-2SY-1B* | KS | E1,E3,E6,E7 |  |
| 2DL | 481.5-502.1 | *QTKW.sicau-2SY-2D* | TKW | E1,E2,E3,E5,E6,E7 |  |
|  | 481.5-512.8 | *QKS.sicau-2SY-2D* | KS | E1,E3,E5,E6,E7 |  |
|  | 487.3-512.8 | *QKT.sicau-2SY-2D* | KT | E1,E3,E4,E5,E6,E7 |  |
|  | 489.7-502.1 | *QFFD.sicau-2SY-2D* | FFD | E1,E2,E4,E6,E7 |  |
| 6DS | 45.9-73.3 | *QKW.sicau-2SY-6D* | KW | E1,E3,E4,E5,E6,E7 |  |
|  | 45.9-73.3 | *QLWR.sicau-2SY-6D* | LWR | E1,E4,E6,E7 |  |
|  | 45.9-73.3 | *QKS.sicau-2SY-6D* | KS | E4,E5,E6,E7 |  |

Note: E1-E7 represent 2017CZ, 2017YA, 2018CZ, 2018YA, 2019CZ, 2019WJ and BLUP dataset, respectively.
